# Molecular characterization of strawberry vein banding virus from China and the development of loop‑mediated isothermal amplification assays for their detection

**DOI:** 10.1038/s41598-022-08981-9

**Published:** 2022-03-22

**Authors:** Junda Ren, Jiaxing Zhang, Qiushi Wang, Yu Zhou, Jingxuan Wang, Ce Ran, Qiaoxia Shang

**Affiliations:** Key Laboratory for Northern Urban Agriculture of Ministry of Agriculture and Rural Affairs, Department of Plant Protection, Beijing University of Agriculture, Beijing, 102206 China

**Keywords:** Microbiology, Plant sciences

## Abstract

Strawberry vein banding virus (SVBV) is one of the serious viral pathogens infecting strawberry worldwide. To understand the molecular characterization of SVBV from China, complete genome sequences of sixteen SVBV isolates were cloned and sequenced. Sequence comparison showed they shared high nucleotide sequence identity (93.6–99.5%) with isolates from China and Japan (96.6–98.4%), while relatively low identity with the isolates from Canada (91.9–93.7%) and USA (85.5–85.9%). Phylogenetic analyses based on the complete genome sequence or coat protein (CP) gene showed the SVBV isolates clustered into three clades correlated with geographic distribution. Recombination analyses identified 13 recombinants and 21 recombinant events, indicating frequent and multiple recombinations in SVBV evolution. Furthermore, a sensitive loop-mediated isothermal amplification (LAMP) method was developed for rapid detection of SVBV isolates, which could be especially suitable for seedling propagation, virus-free culture and routine diagnostics in field investigation. This study offers new understanding of the molecular evolution and may help to improve the management of SVBV.

## Introduction

Strawberry vein banding virus (SVBV), a member of the genus *Caulimovirus* in the *Caulimoviridae*, has a double-stranded DNA genome^1,2^ of approximately 8 kb encapsidated in icosahedral particles of approximately 45 nm diameter^3,4^. SVBV is transmitted in a semi-persistent manner by several *Chaetosiphon* species (*C. fragaefolii, C. thomasiand, C. jacobi*)^5,6^. Its presence has been reported in many countries worldwide: Australia, America, Asia, Africa, Europe (Czech Republic, Slovak Republic, Hungary and Serbia) and China, causing huge economic and production losses^7–11^. The SVBV symptoms range from almost latent infections to necrosis and severe stunting of whole plants. Symptoms of infected strawberry include vein yellowing, greatly reduced stolons, low seed setting rate and growth retardation, and eventually this leads to significant decrease in yield and quality^12^. The most pronounced symptoms of SVBV are often found in mixed infections with other strawberry viruses^13^.

The strawberry plant is a vegetatively propagated perennial and, therefore, the health of the propagation material is important for its cultivation. Routine detection of SVBV for certification purposes relies on time-consuming leaf-graft bioassays on indicator plants^14^. Knowledge of the complete nucleotide sequence of SVBV has enabled the development of less laborious and more sensitive hybridization and polymerase chain reaction (PCR) based detection methods^15–18^. With these methods, strawberry leaf samples infected with SVBV have been successfully screened^10^. However, they often depend on high-precision thermal cyclers and require purified DNA from plant tissue samples before analysis. Thus, user-friendly and field-deployed methods that facilitate early detection would be very helpful for controlling the disease. The discovery of loop-mediated isothermal amplification (LAMP) is a milestone development as it is sensitive, quick, simple and cost-effective^19^. Its main principle is recognizing six to eight distinct regions of a target gene by employing four to six specially designed primers. Utilizing the catalysis of Bst DNA polymerase and incubated at constant temperature (60–65 °C) for 30–60 min, the batch amplification of the target sequence can be realized. The additional advantage of LAMP technique is that final results are directly visible to naked eyes or by using gel electrophoresis. Although LAMP has been successfully applied for the detection of various plant pathogens, no attempt has yet been made to detect SVBV to our knowledge.

As strawberry production expands, China now has the largest acreage planted in the world and some of the strawberry viruses and diseases have also emerged^20^. SVBV is widely distributed in the strawberry producing area of China, but the molecular characterization of SVBV in China remains poorly understood. In this study, we cloned and sequenced the complete genome of sixteen SVBV isolates from China and analyzed its genomic characterization. Moreover, we developed and optimized a very sensitive LAMP assay for SVBV diagnostics. This research will be helpful to the investigation and study of virus disease, thus providing theoretical guidance for sustainable strawberry production.

## Results

### SVBV was prevalent in strawberry plants

**Figure 1:**
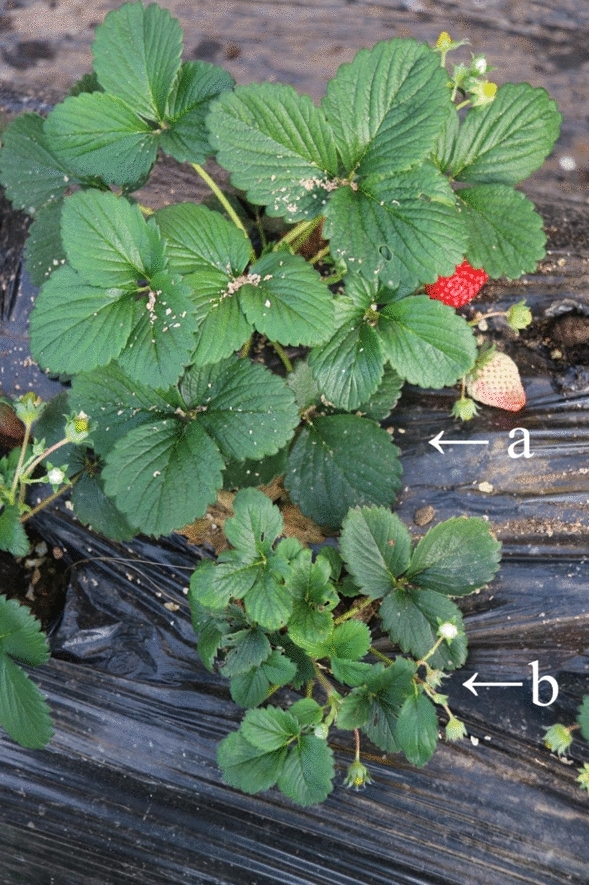
Symptoms of SVBV-infected strawberry plants in the field. (**a**) No obvious symptom; (**b**) Leaf mottle, stunted and clustered plants.

In the field survey, strawberry plants showed the typical symptoms of viral disease, such as appearing stunted, clustered, deformed, and suffering from mosaic, but some plants were symptomless (Fig. 1). To clarify the occurrence of SVBV in strawberry plants, 259 strawberry samples from different regions of China were collected and detected by PCR and sequenced using specific primers. The types of fields that were surveyed including organic production, soil cultivation, substrate culture, elevated cultivation and integrated production. The results showed that 71 samples (27.4%) were SVBV-positive and this virus disease happened very commonly on strawberry in China.

### Complete genome sequence characterization and phylogenetic analyses of SVBV

The complete genome sequences of sixteen SVBV isolates from China were cloned and submitted to the GenBank database (Accession Nos: MN956520, MT012732, MT012734, MT027006, MT027007, MT036053, MT036054, MT036055, MT036056, MT036057, KX249738, KX249737, KX249736, KX249735, MF197916 and KT250632). The length of these isolates varied from 7846 to 7942 nts and coding capacity analyses showed the double-stranded DNA genome contained seven putative open reading frames (ORFs), which was consistent with other reported SVBV isolates and other members in the genus *Caulimovirus*^21^ (Supplementary Fig. 1). ORF I encoded the putative viral movement protein involved in cell-to-cell movement of 329 aa with a predicted molecular mass of 37.8 kDa. The conserved DXR motif which may be functionally important was also present. ORF II encoded a putative aphid-transmission-associated protein of 162 aa and 18.5 kDa. ORF III encoded a putative virion-associated protein of 116 aa or 117 aa in some isolates from Beijing with the acc. no. MT036053-MT036055, KT250632 and MT027007. ORF IV encoded a coat protein of 471 aa and 55.0 kDa which contains the conserved zinc-finger domain with the arrangement Cx2Cx4Hx4C typical of all the caulimoviruses. ORF V encoded a putative reverse transcriptase of 704 aa and 80.6 kDa with the motifs of this multifunctional proteins in SVBV: a Leu-zip motif near the N-terminus, an Asp-proteinase domain, a reverse transcriptase domain and an RNase-H domain. ORF VI encoded a putative inclusion body matrix protein of 520 aa and 5.9 kDa. ORF VII encoded a putative protein of 107 aa. The non-coding region (NCR) was between the ORF VI and ORF VII, containing a CAT-like element (GGCCAT), an eukaryotic promoter TATA box (TATATAA) and a poly (A) signal (AATAAA).

### Sequence comparison and phylogenetic analyses

**Figure 2:**
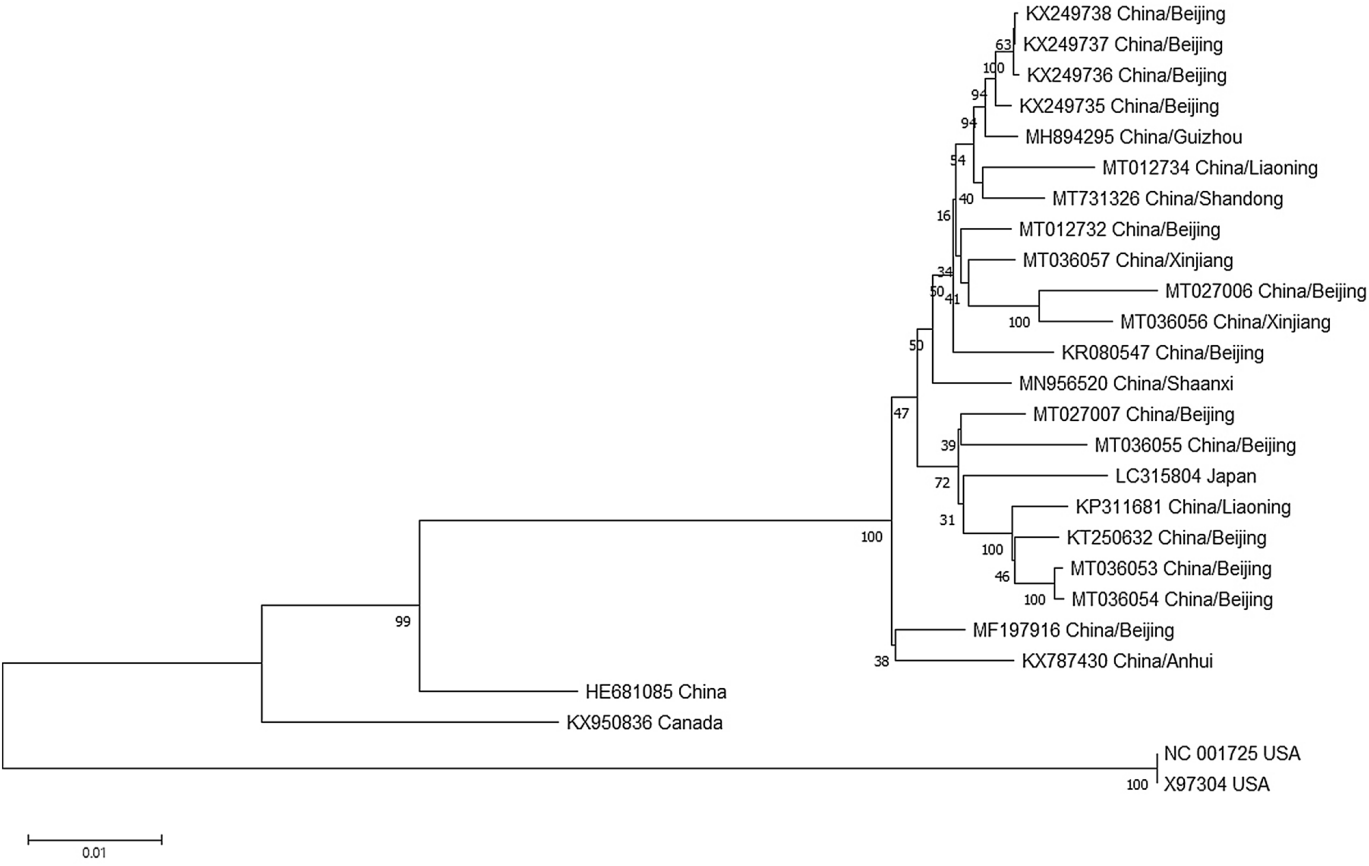
Phylogenetic relationship of the complete genomic sequences of SVBV isolates. All complete genomic sequences of SVBV isolates currently available were used to construct the NJ phylogenetic tree with 1000 bootstrap value by the software of MEGA 7.

**Figure 3:**
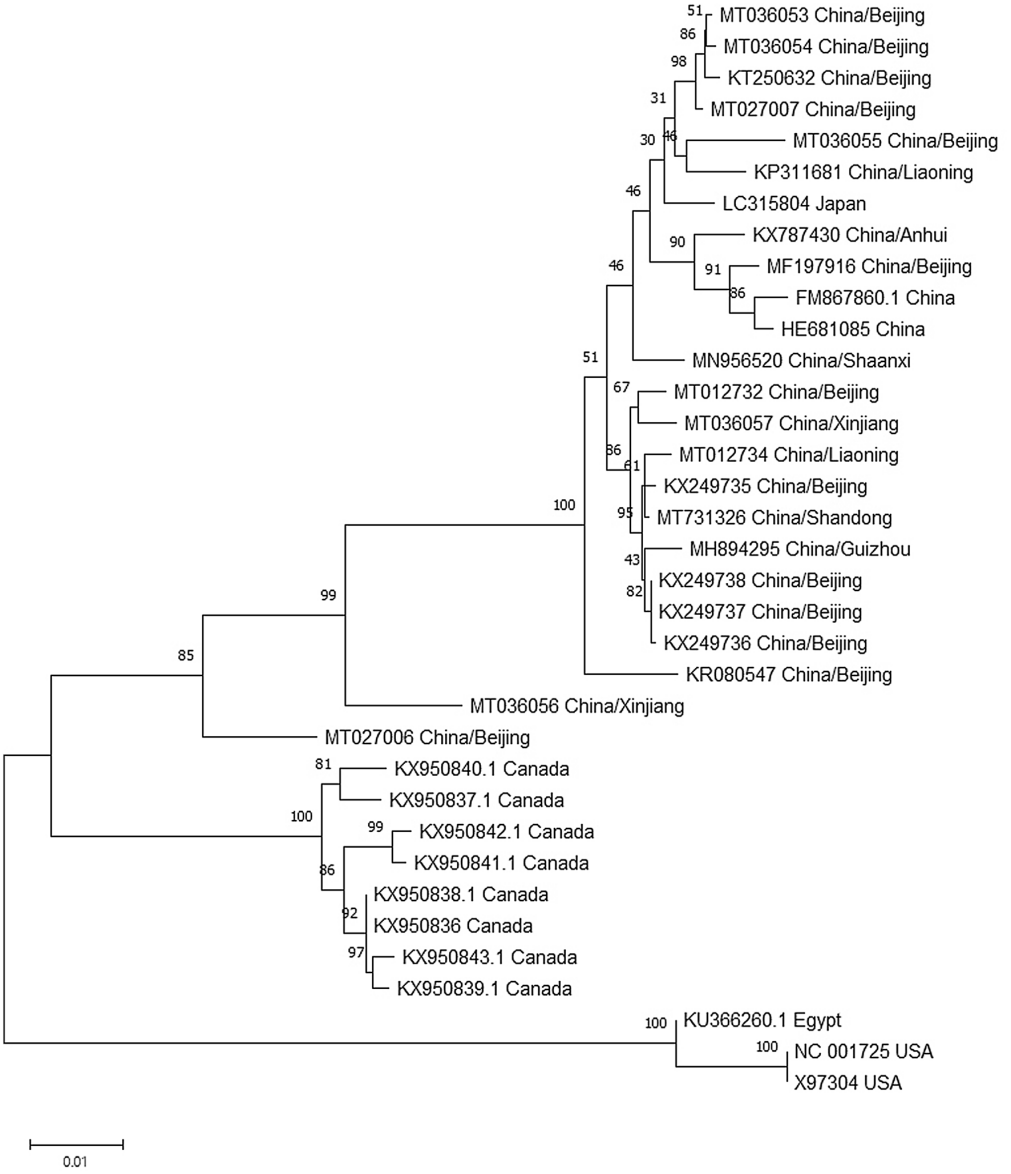
Phylogenetic relationship of the coat protein (CP) gene sequences of SVBV isolates. All coat protein (CP) gene sequences of SVBV isolates currently available were used to construct the NJ phylogenetic tree with 1000 bootstrap value by the software of MEGA 7.

Sequence comparison showed the cloned sequences in this study shared high nucleotide sequence identities with each other and other reported isolates from China (93.6–99.5%) and Japan (96.6–98.4%) while relative low identities of 91.9–93.7% with the Canada isolate and 85.5–85.9% with the USA isolate (Supplementary Fig. 2). At the ORF level, all the isolates from China shared 95.4–99.8% and 95.7–99.8% sequence identity at nt and aa level respectively except for the isolate (HE681085). To elucidate the relationship of different SVBV isolates, a phylogenetic tree was constructed with the available SVBV complete genomic sequences. The phylogenetic tree illustrated that the isolates from USA, the isolates from Canada and the isolates from China and Japan clustered separately into three clades (Fig. 2), which was also in accordance with the sequence comparison. To further confirm the phylogenetic relationship, the coat protein (CP) gene nucleotide sequence based phylogenetic tree was also constructed (Fig. 3). Many Canadian isolates (shown in Fig. 3) were only sequenced for the CP gene and the complete genomic sequences were still unknown. So there are so many variants from Canada compared to Fig. 2. The same topology was observed, indicating that the SVBV evolution was strongly associated with geographical distribution.

### High recombinant frequency and multiple recombinant events in SVBV

A total of thirteen isolates were identified to be potential recombinants with 21 recombination events detected, indicating a relative high recombinant frequency in SVBV. One recombination event was detected in the isolates from China (MT036056, MT731326, KR080547, MT012734 and KP311681) and Japan (LC315804). Two recombination events were detected in the isolates HE681085, MT027006, KX950836, KT250632, MT036055 and MT027007 in different regions while in the isolate MT036055 the two recombination regions overlapped. Three recombination events were detected in the isolate KX787430. The recombination regions were distributed nearly along the whole genome, indicating no recombination hotspot (Supplementary Table S1). Recombination is an important mechanism in virus evolution that can lead to increased or decreased variation and is a major player in virus speciation events leading to emerging viruses. This phenomenon has been reported in single-stranded DNA viruses involving those of begomoviruses and mastreviruses in the family *Geminiviridae* including geminiviruses and double-stranded DNA virus including cauliflower mosaic virus in the genus *Caulimovirus*^21^.

### Development and optimization of the LAMP assay

**Figure 4:**
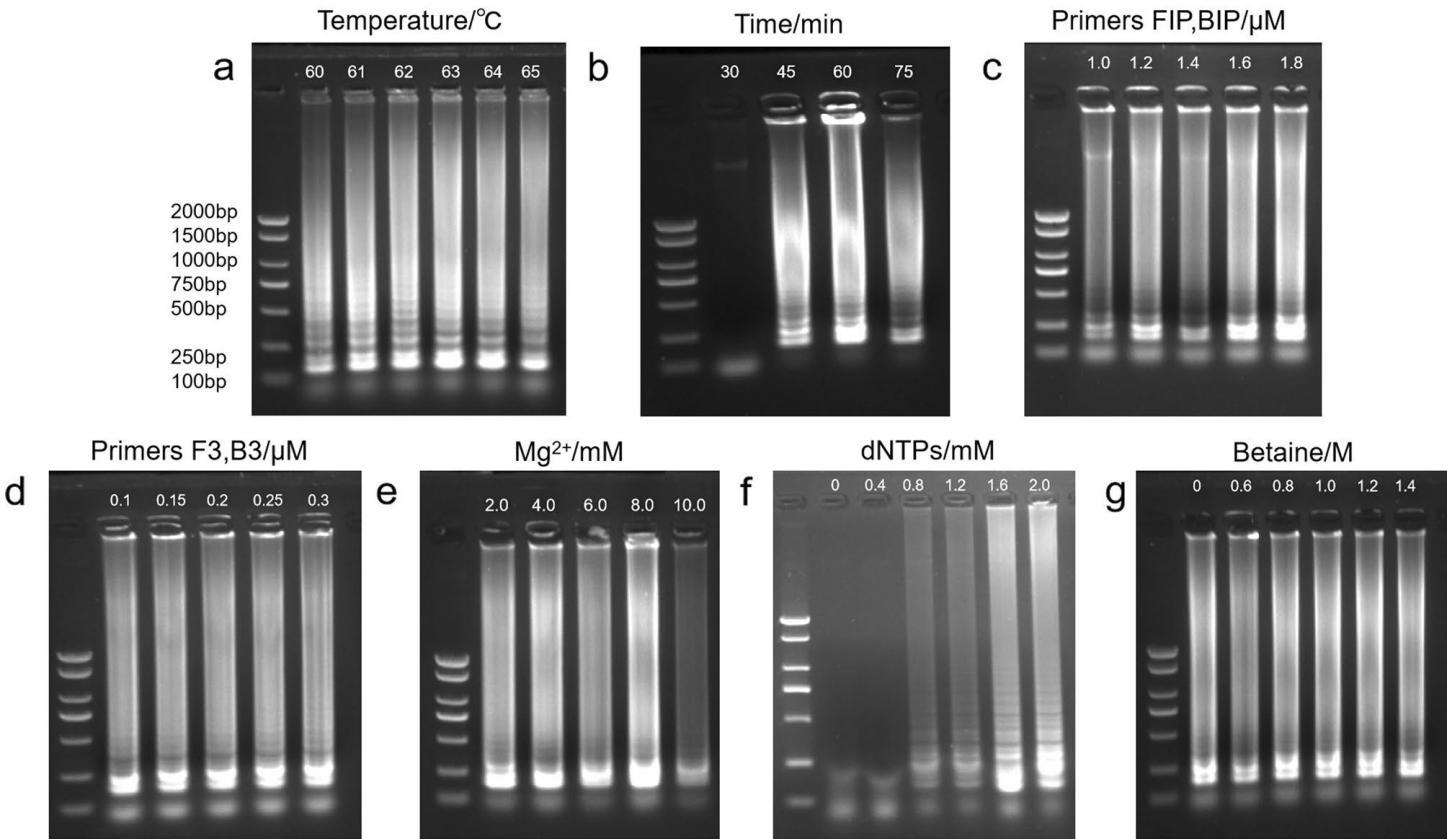
Optimization of the LAMP system to detect SVBV. (**a**) temperature: 60, 61, 62, 63, 64, 65 °C; (**b**) time: 30, 45, 60, 75 min; (**c**) primers FIP/BIP: 1.0, 1.2, 1.4, 1.6, 1.8 μM; (**d**) primers F3/B3: 0.1, 0.15, 0.2, 0.25, 0.3 μM; (**e**) Mg^2+^: 2.0, 4.0, 6.0, 8.0, 10.0 mM; (**f**) dNTPs: 0, 0.4, 0.8, 1.2, 1.6, 2.0 mM; (**g**) Betaine: 0, 0.6, 0.8, 1.0, 1.2, 1.4 M; lane M, DL 2000 DNA marker (100–2000 bp). The grouping of gels cropped from different gels were divided with white space. The full-length gels are presented in Supplementary Fig. S4.

LAMP assay conditions were optimized in a stepwise manner, with one parameter modified at a time following the order of gels presented in Fig. 4a–g. As shown in the Fig. 4, different temperatures and times had some impact on the formation of waterfall-type bands, and then directly affected the observation of the detection results. The bands were clear and bright when incubated at 62 °C for 45 min. When the concentration of primers FIP/ BIP was 1.0 μM no clear cascading bands were formed, while a clear waterfall strip could be formed with higher concentrations. The primer F3/B3 had little effect on the formation of waterfall bands, the same is true for Mg^2+^ and Betaine. The optimized amplification was achieved by applying incubation for 45 min at 62 °C. The finally optimized reaction system was 1.2 μM SVBV-FIP/BIP, 0.1 μM SVBV-F3/B3, 2 mM Mg^2+^, 1.6 mM dNTPs and 1.0 M Betaine (Fig. 4). The full-length gels are presented in Supplementary Fig. S4.

### Specificity, sensitivity and field applicability of the LAMP assay

**Figure 5:**
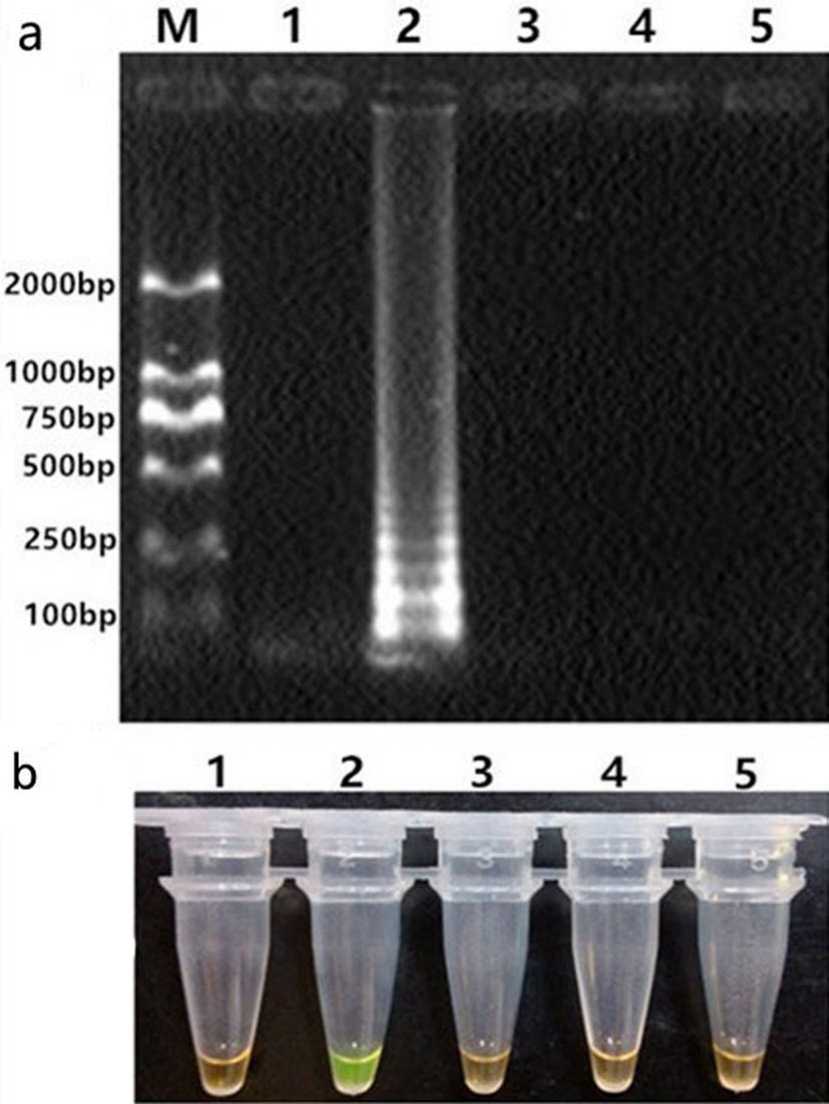
Specificity of the LAMP assay for SVBV. Detection of LAMP products by (**a**) agarose gel electrophoresis and (**b**) visual inspection by SYBR green I. Lane M, DL2000 DNA Marker; lane 1, Negative control; lane 2–5, Samples with DNAs/cDNAs of Strawberry vein banding virus (SVBV), Strawberry mottle virus (SMoV), Strawberry mild yellow edge virus (SMYEV) and Strawberry crinkle virus (SCV). The full-length gels are presented in Supplementary Fig. S5.

**Figure 6:**
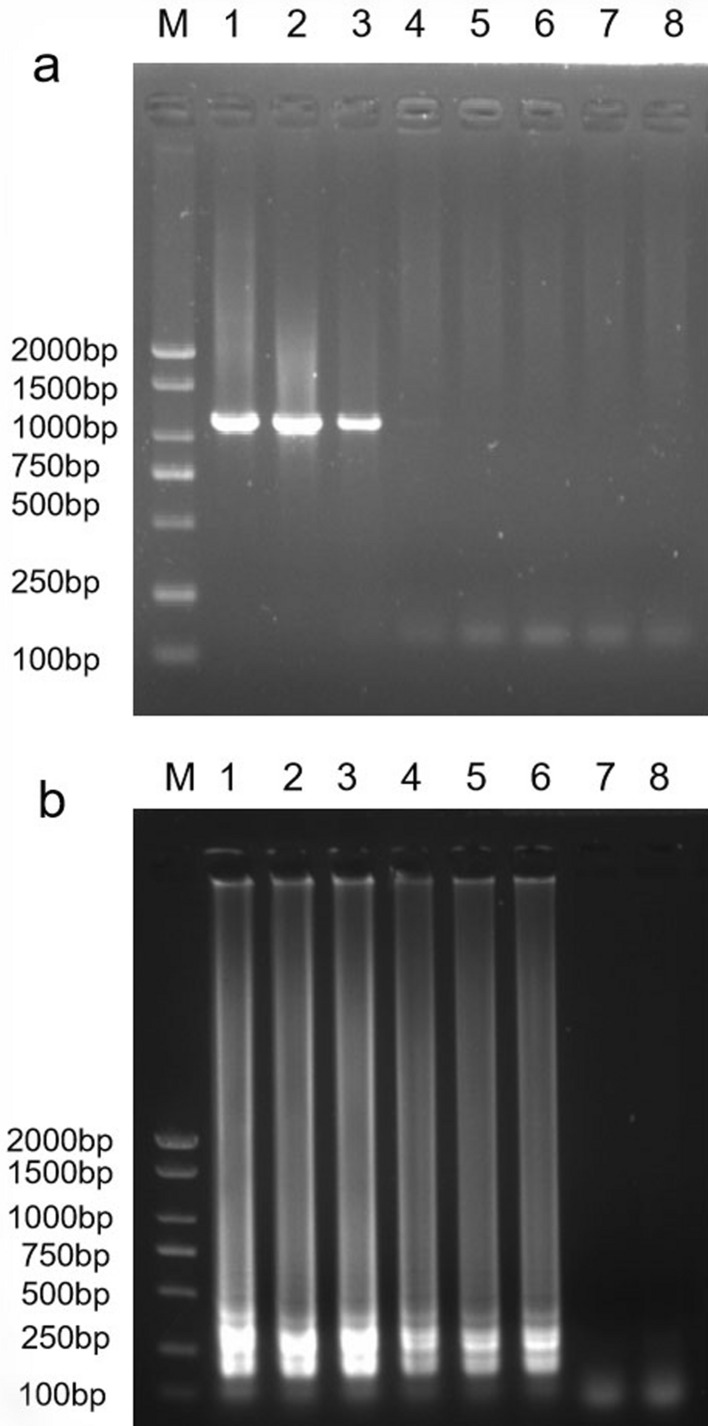
Sensitivity comparison of the PCR and LAMP methods for the detection of SVBV. (**a**) Detection of PCR products by agarose gel electrophoresis; (**b**) detection of LAMP products by agarose gel electrophoresis; Lane M, DL2000 DNA Marker; lane 1, DNA of SVBV; lanes 2–7, 10-folds serial dilutions of DNA of SVBV by 10^–1^, 10^–2^, 10^–3^, 10^–4^, 10^–5^, 10^–6^; lane 8, negative control. The full-length gels are presented in Supplementary Fig. S6.

The specificity of the LAMP detecting SVBV were confirmed by both the gel electrophoresis and visualized analysis to amplify only DNA from SVBV, with no amplification of the negative control and other viruses (Fig. 5). Obvious bands were not achieved when the dilution multiple exceeded 100 for traditional PCR method, while the LAMP method was 1000 times more sensitive than PCR with the dilution limit of 10^–5^ in comparison (Fig. 6).

**Figure 7:**
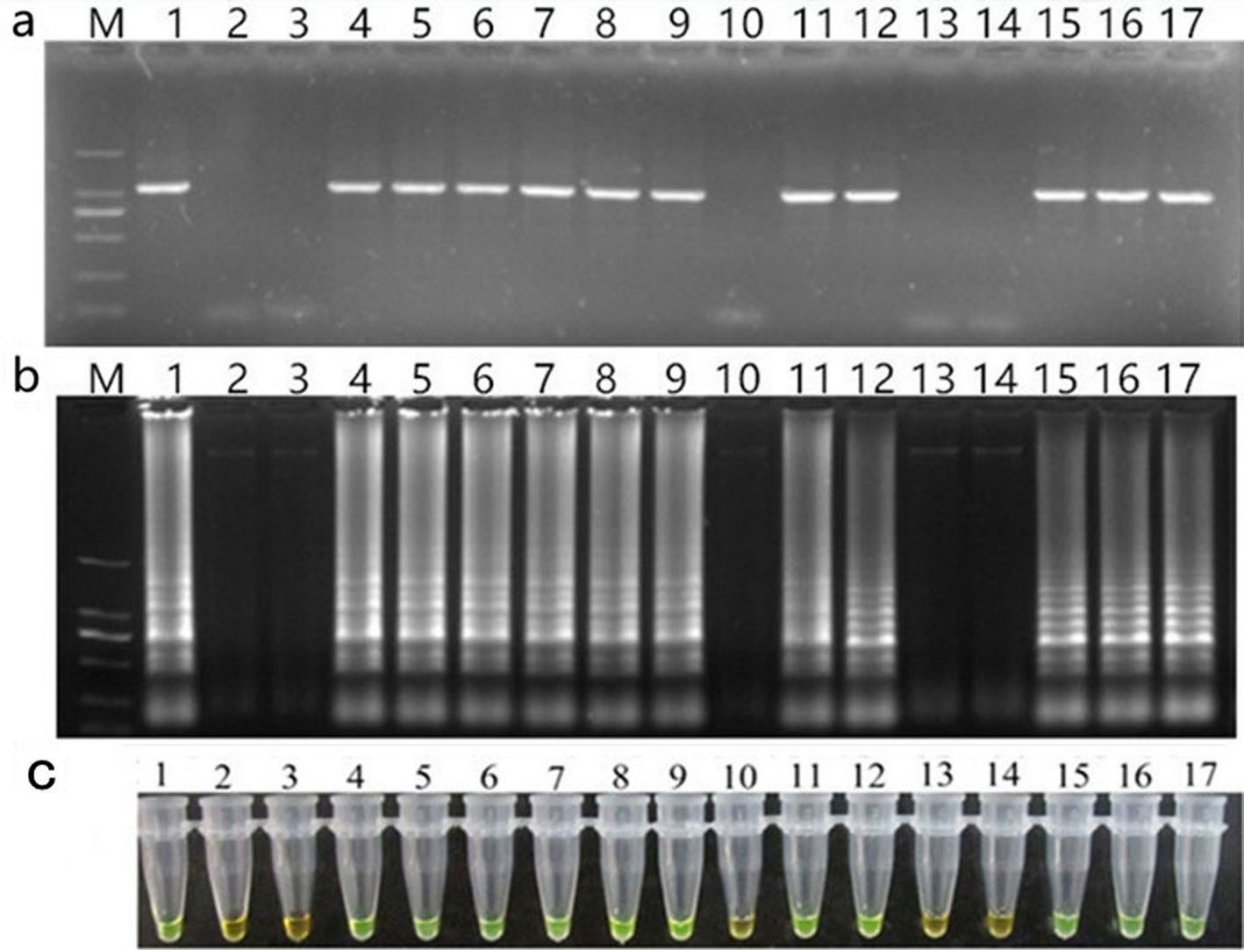
Field applications of PCR and LAMP detection of SVBV. (**a**) Electrophoretic results of the PCR products of 15 strawberry leaf samples in the field; (**b**) agarose gel illustrating the LAMP products of the samples; (**c**) visualization results of the LAMP assay for the samples. Lane M, DL 2000 DNA marker (100–2000 bp); lane 1, positive control; lane 2, negative control; lanes 3–17, strawberry leaf samples in the field. The full-length gels are presented in Supplementary Fig. S7.

Fifteen strawberry leaf samples were collected in various commercial production fields. Eleven of the samples were positive for SVBV, the others were negative. Color changes were noted after addition of SYBR green I, with positive samples turned green and negative samples remained orange. Those observations were consistent with the gel electrophoresis results. These results proved that the PCR and LAMP methods shared high degree of consistency (Fig. 7). In conclusion, the LAMP method developed and optimized in this study is highly specific and much more sensitive than the traditional PCR method, which is completely suitable for field detection of SVBV.

## Discussion

At present, there are more than 20 viral diseases that associated with plant decline and yield loss infecting strawberries worldwide^14,22^. Of which the most widespread, prevalent and serious viruses are four aphid-transmitted viruses, including SVBV, SMoV, SMYEV and SCV. SVBV is widely distributed in strawberry growing areas, occurring in China, Japan, America, Brazil, Australia, the Czech Republic, Italy and many other countries^10,23,24^. We have been engaged in strawberry virus detection and disease investigation for more than ten years in China^20,25,26^. An interesting finding is that the SVBV have higher prevalence and dispersion compared to other aphid-transmitted viruses of strawberry and there is a tendency of gradual aggravation in recent years (unpublished data). Cultivated strawberry plants are often symptomless when infected with SVBV alone, while co-infection with other strawberry viruses has the potential to cause serious symptoms in strawberry plants^27^.

The primers we used for the PCR assay were designed according to the highly conserved regions (coat protein gene) in the SVBV genomes, which was first used for the American and European sources of SVBV in 1996. After more than 20 years of use these primers are still suitable for most isolates all over the world. We have designed and compared different pairs of primers and found that the SV5508F/SV6606R worked best (data not shown). The brightness of all specific bands are basically consistent with no weak bands appearing, which was in high accordance with other reports^28^. The whole genome of 16 SVBV isolates were sequenced and multiple sequence alignments were performed in this study (Supplementary materials S3). There might be some other highly conserved regions in the SVBV genomes having potential to develop PCR primers that would capture the diversity of all SVBV sequences in the database. Our results will provide the basis for different studies to design primers for different purposes.

The LAMP assays have been reported for the detection of numerous plant pathogens recently. This method is more reliable, rapid, simple, economical and sensitive than standard PCR on the whole. In the present study, a LAMP assay for the detection of SVBV was developed and found to be more sensitive than conventional PCR technique, as was reported earlier for other pathogens^19,29–35^. This method has also been verified to be practical for screening large numbers of field samples, having relevance for field surveying in terms of decisions to keep /manage/ destroy plants.

Strawberry production mainly relies on stolons for vegetative propagation, which provides opportunities for the accumulation and spread of the virus. Once a strawberry plant carries the virus, it will last through the whole growth period. Worse still is that there are no effective measures to prevent strawberry virus disease completely for the moment. Therefore, our recommendation is to eliminate virus-bearing plants as soon as they are found in the field survey if conditions permit. There was evidence that LAMP assay could work with a crude extract^36^. For example, in plant virus diagnosis, it could be possible to use direct crude plant extracts in order to avoid total RNA or DNA extraction, shortening the processing time, allowing the simultaneous analysis of multiple samples, and drastically reducing the total cost for single analysis^37^. Crude extracts were not used in this research, but more attempts will be made in future studies.

When it comes to the cost, labor efficiency, etc., LAMP has great advantages over conventional PCR^25^. Firstly, the amplification reaction can be achieved with a cheap water bath or heater and the results can be interpreted visually without any specific instruments. Secondly, the reagents used in LAMP are also cheaper or equivalent to the standard PCR, including the extraction and testing processes^25^. Furthermore, the LAMP takes less time than PCR method, which is more appropriate in terms of ‘time cost’. It has the characteristics of easier operation and the LAMP assay is more labor-saving.

## Materials and methods

### Plant materials and DNA extraction

Experimental research and field studies on plants including the collection of plant material, complied strictly with relevant institutional, national, and international guidelines and legislation such as the IUCN Policy Statement on Research Involving Species at Risk of Extinction and the Convention on the Trade in Endangered Species of Wild Fauna and Flora. 259 samples of different strawberry plants were surveyed from field in some regions of China including Beijing, Shaanxi, Xinjiang, Liaoning and numbered in sequence. The fields were chosen randomly from different regions of China and they showed signs of a mixture of healthy, moderately declining, and significant decline. Some of them have a long history of cultivation while others are new nurseries only planted 1–2 years. The production system and planting patterns also varied from each other, encompassing organic production, integrated production, soil cultivation, hydroponics, substrate culture and elevated cultivation. To obtain more isolates of SVBV and reflect as much genetic diversity as possible, we collected popular cultivars planted in China like Hongyan, Zhangji, Suizhu, Danmei, Hexiang, Jingchengxiang1, Jingchengxiang2, Jingyixiang1, Jingyixiang2 etc. from 2015 to 2021. A majority of the collected strawberry plants showed typical symptoms of viral disease, such as stunted, clustered, deformity, chlorisis, leaf curling, vein banding and necrosis, but other individual plants were symptomless. The tissues selected for the LAMP assay were young leaves from each strawberry plant. Washing the leaves with nuclease-free water, blotter dried, and subsequently wiping with 70% ethanol was done to minimize surface contamination. Total genomic DNA was extracted from 0.2 g fresh leaf tissue collected in the field. We used the Aidlab Genomic DNA Extraction Kit (Aidlab Co., Beijing, China) according to the manufacturer's instructions. The DNA and redundant leaves were stored at − 80 °C. The DNA was extracted and stored for all of the 259 samples individually. DNA was examined on 1% agarose gel and NanoDrop 2000 Spectrophotometers (Thermo Scientific, USA).

### Cloning of SVBV genome sequence

Sixteen isolates in total of SVBV were chosen for sequencing. These isolates were obtained from a subset of the 259 strawberry samples described above. All of the samples were obtained from individual plants. Overlapping primers (Table 1) used for PCR amplification were designed based on the published SVBV genome sequences (accession numbers KP311681.1, KR080547.1, HE681085.1 and X97304.1). The extraction of DNA was described above and the PCR reactions were performed in a 25 μL volume with reaction mixtures containing 2.5 μL of 10 × PCR buffer, 1μL of DNA, 2 mM of each dNTP, 0.5 mM of each primer, one unit of LATaq DNA polymerase (TaKaRa, Dalian, China) and brought to volume with ddH_2_O. The amplified genome segments were subsequently cloned. The target fragments were purified according to the instructions of agarose gel purification kit (Aidlab Biotechnologies Co., Ltd). Then the PCR products were cloned into the pBM23 cloning vector using the BMMach1-T1 competent cells and sequenced at Biomed Gene Technology Co., LTD. The kind of sequencing was Sanger's method-Dideoxynucleotide chain termination and the primary instrument was 3730 XL DNA Analyzer. The obtained sequences were aligned with those available in GenBank using the BLAST algorithm (http://ncbi.nlm.nih.gov/BLAST/). The complete genome sequences were assembled and analyzed with DNAMAN 7.0 (LynnonBiosoft, Quebec, Canada) and DNASTAR 6.0 (DNASTAR Inc., Madison, WI, USA). Default parameters were used and there was no modification to software default.

**Table 1:** Primers used for complete genome sequence of SVBV.

No.	Primers	Sequence	Position	Annealing temperature (°C)
1	SV7618F	TGAGCCATTTCATGAGCAAGG	7618–1648	54
	SV1648R	TGCCTGATCAATCTTCTGTGAG		
2	SV1649F	AGTGTTCAAATCCCCTAGCCT	1648–2310	54
	SV2130R	AG(A)TCTCATCTCATTGTCCCATTC		
3	SV5950F	GACCCCAAGCTCCATTATCM	5950–7630	53
	SV7630R	CTTGCTCATGAAATGGCTCA		
4	SV3808F	ACCAACCATGTACCAAGCAAC	3808–5172	55
	SV5172R	CCCAATGGTCATCTGATGCG		
5	SV4777F	GCAAAGCCCTAGGAATAGTGC	4777–6430	55
	SV6430R	CGGCTCCTTCAATGAAACCATAA		
6	SV3790F	TARTGCAGGTACAAATTGCA	3790–5439	55
	SV5439R	AGCCATTTGCCATCTCAC		
7	SV5950F	GACCCCAAGCTCCATTATCM	5950–7638	58
	SV7638R	CTTGCTCATGAAATGGCTCA		
8	SV7272F	CAGAACCTCCCTGCTTAC	7272–996	56
	SV996R	GGGCCTTAAACCTAGCATCC		
9	72100F	CAGAACCTCCCTGCTTAC	7272–1124	53
	7-11R	TTGATGGTAGAGAGCTAGGT		
10	7-11F	AGTGTTCAAATCCCCTAGCCT	222–2310	54
	2300R	AG(A)TCTCATCTCATTGTCCCATTC		
11	2030F	M(C)ACCTATCGTCCGAACCG	2125–3834	52
	3040R	TTGCY(A)TGR(G)TACAW(T)GGTTGGT		
12	3040F	TGTAAT(C)GAR(A)ATCGGACAY(T)	3104–5435	52
	4555R	AGCCATTTGCCATCTCAC		
13	4957F	CCTTC(T)CCAGACCAGTTAGCA	4906–7463	56
	6574R	CTCCTGACTCTCGGGATTCACGCTA		

### SVBV sequence analyses

All the available genome sequences of SVBV strains in the GenBank database were downloaded and aligned with Clustal X program. Phylogenetic tree based on the genomic nucleotide sequence was performed by neighbor-joining (NJ) method using MEGA7^38^ with the best model tested in this software and the confidence was estimated by 1000 bootstrap replicates. Recombination was detected with various recombination detection methods implemented in the software RDP5^39^ including programs RDP, GENECONV, BOOTSCAN, MAXCHI, CHIMAERA, SISCAN and 3SEQ, performed with the default configuration, except that options of circular sequence was selected. Only recombination events detected by at least five different methods were accepted.

### Development and optimization of LAMP for detection of SVBV

According to the sequences of SVBV (AY605663, AY955374, FM867860, JN542480, NC001725) released by NCBI GenBank, the highly conserved region of coat protein gene analyzed by DNAMAN 7.0 was chosen as the target sequence. The primers of LAMP were designed by online software Primer3 Input (http://bioinfo.ut.ee/primer3-0.4.0/primer3/), including four specific primers covering six regions of the CP gene (Table 2). The LAMP assay was slightly modified according to the method described previously^25,26^. The basic reaction system consisted of 1.6 μM each of the primers SVBV-FIP and SVBV-BIP, 0.2 μM each of SVBV-F3 and SVBV-B3, 1.6 mM dNTPs, 1 M Betaine, 2 mM MgSO_4_, 1 μl *Bst* DNA Polymerase (8 units), 2.5 μl 10 × Bst buffer (20 mM Tris–HCl, pH8.8, 10 mM KCl, 10 mM (NH_4_)_2_SO_4_, 0.1% Triton X-100 and 2 mM MgSO_4_), 2 μl DNA template, adding DEPC H_2_O to a total volume of 25 μl. Samples were incubated in a 65 °C water bath for 60 min and finally 80 °C heat shock for 10 min. The primers used for traditional PCR detection of SVBV were SV5508F (5ʹ-TCGGGAAYTTGCAGGWAAAACATAG-3ʹ) and SV6606R (5ʹ-TACTCGTGATTCTCAGGTAGATTGG-3ʹ)^28,40^, referring to the PCR conditions under the cloning section only with the annealing temperature (55 °C) specific and the length of the target fragment was 1098 bp.

**Table 2:** Primers designed for LAMP assay of SVBV.

No.	Name	Sequence (from 5ʹ to 3ʹ)
1	SVBV-FIP	CAGTGTGAAGTGATTCCAACAATGATCTTATCCTTACTCTCGCAAAG
2	SVBV-BIP	CAAACAAGCTTCTTCAACAGGACGAATTTGTCAGAGTTGTCA
3	SVBV-F_3_	CAGAGAAGGCTCTTACAAATGA
4	SVBV-B_3_	CGAGTTCCCTGTGTAAGATAGTTAG

In order to obtain the best reaction conditions, the control variable method was used to adjust the LAMP system. The variables involved were temperature (60, 61, 62, 63, 64, 65  °C), incubated time (30, 45, 60, 75 min), SVBV-FIP/BIP primers (1.0, 1.2, 1.4, 1.6, 1.8 μM), SVBV-F3/B3 primers (0.1, 0.15, 0.2, 0.25, 0.3 µM), Mg^2+^ (2.0, 4.0, 6.0, 8.0, 10.0 mM), dNTPs (0, 0.4, 0.8, 1.2, 1.6, 2.0 mM) and Betaine (0, 0.6, 0.8, 1.0, 1.2, 1.4 M). When certain LAMP parameter was being optimized, the settings for the fixed parameters were the basic reaction system, which consisted of 1.6 μM each of the primers SVBV-FIP and SVBV-BIP, 0.2 μM each of SVBV-F3 and SVBV-B3, 1.6 mM dNTPs, 1 M Betaine, 2 mM MgSO4, 1 μl Bst DNA Polymerase (8 units), 2.5 μl 10 × Bst buffer (20 mM Tris–HCl, pH8.8, 10 mM KCl, 10 mM (NH4)2SO4, 0.1% Triton X-100 and 2 mM MgSO4), 2 μl DNA template, adding DEPC H2O to a total volume of 25 μl. Samples were incubated in a 65 °C water bath for 60 min and finally 80 °C heat shock for 10 min.

Results of PCR and LAMP assay were analyzed by gel electrophoresis with 1% agarose in Tris acetate-EDTA buffer (TAE: 0.04 M Tris acetate, 1 mM EDTA) and visualized on a UV transilluminator. Additionally, the LAMP outcome could be observed through naked eyes by adding 1 μl SYBR green I nucleic acid dye (Beijing Solarbio Science & Technology Co., Ltd.) to the starting reaction volume of 25 µl, of which the color changed indicating a positive reaction.

### Specificity, sensitivity and field applicability of the LAMP assay

The specificity of the LAMP assay was tested using the DNA obtained from SVBV and cDNA from three other important strawberry virus diseases: strawberry mottle virus (SMoV), strawberry mild yellow edge virus (SMYEV) and strawberry crinkle virus (SCV). All of the cDNA controls of the SMoV, SMYEV and SCV had been confirmed accurately to be positive by both RT-PCR and RT-LAMP methods^25,26,41^. Selecting the DNA from healthy plants as the negative control. The positive PCR/LAMP controls were the strawberry leaves infected with SVBV, which had been detected and confirmed before^25,26,41^. To compare the relative sensitivity of LAMP and PCR methods, tenfold serial dilutions (different diluents from 10^0^ to 10^–6^) of SVBV genomic DNA and negative control were prepared as the template of amplification. According to the established detection system, 15 strawberry samples selected randomly from the 259 field samples were tested to evaluate the stability and practicality of this method for field application.

## Supplementary Information

Supplementary Figure 1.

Supplementary Figure 2.

Supplementary Figures.

Supplementary Tables.
