# Supplementary figures and images for: Molecular characterization of strawberry vein banding virus from China and the development of loop‑mediated isothermal amplification assays for their detection
Source: Sci Rep. 2022 Mar 22;12:4912. doi: 10.1038/s41598-022-08981-9 (PMC8940885; doi:10.1038/s41598-022-08981-9)

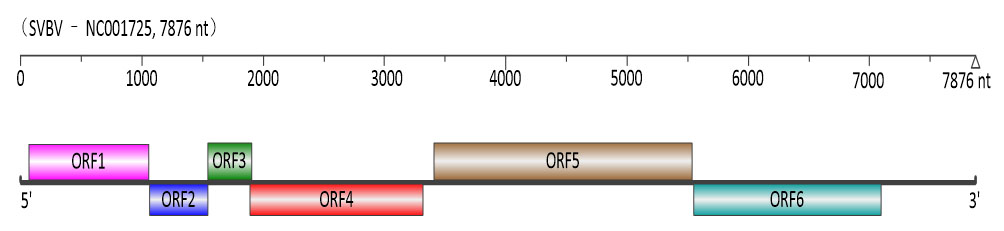

Supplement: Supplementary file 1 — Supplementary Figure 1. [file 41598_2022_8981_MOESM1_ESM.jpg]
