# Supplementary material for: Molecular characterization of strawberry vein banding virus from China and the development of loop‑mediated isothermal amplification assays for their detection
Source: Sci Rep. 2022 Mar 22;12:4912. doi: 10.1038/s41598-022-08981-9 (PMC8940885; doi:10.1038/s41598-022-08981-9)
Supplement: Supplementary file 2 — Supplementary Figure 2. [file 41598_2022_8981_MOESM2_ESM.htm]

|  |  |  |  |  |  |  |  |  |  |  |  |  |  |  |  |  |  |  |  |  |  |  |  |  |  |  |  |  |  |  |  |  |  |  |  |  |  |  |  |  |  |  |  |  |  |  |  |  |  |  |  |  |  |  |  |  |  |  |  |  |  |  |  |  |  |  |  |  |  |  |  |  |  |  |  |  |  |  |  |  |  |  |  |  |  |  |  |  |  |  |  |  |  |  |  |  |  |  |  |  |  |  |  |  |  |  |  |  |  |  |  |  |  |  |  |  |  |  |  |  |  |  |  |  |  |  |  |  |  |  |  |  |  |  |  |  |  |  |  |  |  |  |  |  |  |  |  |  |  |  |  |  |  |  |  |  |  |  |  |  |  |  |  |  |  |  |  |  |  |  |  |  |  |  |  |  |  |  |  |  |  |  |  |  |  |  |  |  |
| --- | --- | --- | --- | --- | --- | --- | --- | --- | --- | --- | --- | --- | --- | --- | --- | --- | --- | --- | --- | --- | --- | --- | --- | --- | --- | --- | --- | --- | --- | --- | --- | --- | --- | --- | --- | --- | --- | --- | --- | --- | --- | --- | --- | --- | --- | --- | --- | --- | --- | --- | --- | --- | --- | --- | --- | --- | --- | --- | --- | --- | --- | --- | --- | --- | --- | --- | --- | --- | --- | --- | --- | --- | --- | --- | --- | --- | --- | --- | --- | --- | --- | --- | --- | --- | --- | --- | --- | --- | --- | --- | --- | --- | --- | --- | --- | --- | --- | --- | --- | --- | --- | --- | --- | --- | --- | --- | --- | --- | --- | --- | --- | --- | --- | --- | --- | --- | --- | --- | --- | --- | --- | --- | --- | --- | --- | --- | --- | --- | --- | --- | --- | --- | --- | --- | --- | --- | --- | --- | --- | --- | --- | --- | --- | --- | --- | --- | --- | --- | --- | --- | --- | --- | --- | --- | --- | --- | --- | --- | --- | --- | --- | --- | --- | --- | --- | --- | --- | --- | --- | --- | --- | --- | --- | --- | --- | --- | --- | --- | --- | --- | --- | --- | --- | --- | --- | --- | --- | --- |
|  |  |  |  |  |  |  |  |  |  |  |  |  |  |  |  |  |  |  |  |  |  |  |  |  |  |  |  |  |  |  |  |  |  |  |  |  |  |  |  |  |  |  |  |  |  |  |  |  |  |  |  |  |  |  |  |  |  |  |  |  |  |  |  |  |  |  |  |  |  |  |  |  |  |  |  |  |  |  |  |  |  |  |  |  |  |  |  |  |  |  |  |  |  |  |  |  |  |  |  |  |  |  |  |  |  |  |  |  |  |  |  |  |  |  |  |  |  |  |  |  |  |  |  |  |  |  |  |  |  |  |  |  |  |  |  |  |  |  |  |  |  |  |  |  |  |  |  |  |  |  |  |  |  |  |  |  |  |  |  |  |  |  |  |  |  |  |  |  |  |  |  |  |  |  |  |  |  |  |  |  |  |  |  |  |  |  |  |  |
|  |  |  |  |  |  |  |  |  |  |  |  |  |  |  |  |  |  |  |  | \* |  |  |  |  |  |  |  |  | 2 | 0 |  |  |  |  |  |  |  |  |  | \* |  |  |  |  |  |  |  |  | 4 | 0 |  |  |  |  |  |  |  |  |  | \* |  |  |  |  |  |  |  |  | 6 | 0 |  |  |  |  |  |  |  |  |  | \* |  |  |  |  |  |  |  |  | 8 | 0 |  |  |  |  |  |  |  |  |  | \* |  |  |  |  |  |  |  | 1 | 0 | 0 |  |  |  |  |  |  |  |  |  | \* |  |  |  |  |  |  |  | 1 | 2 | 0 |  |  |  |  |  |  |  |  |  | \* |  |  |  |  |  |  |  | 1 | 4 | 0 |  |  |  |  |  |  |  |  |  | \* |  |  |  |  |  |  |  | 1 | 6 | 0 |  |  |  |  |  |  |  |  |  | \* |  |  |  |  |  |  |  |  |
| M | N | 9 | 5 | 6 | 5 | 2 | 0 |  | : |  | T | G | G | T | A | T | C | A | G | A | G | C | C | T | G | C | T | A | C | T | C | T | C | A | C | C | A | G | T | A | G | G | A | G | A | T | C | C | T | C | A | G | A | G | A | C | C | A | C | C | T | C | T | T | C | A | A | C | A | A | C | A | T | C | A | T | A | G | A | A | T | G | T | C | T | G | A | A | G | A | A | G | A | G | A | T | C | A | G | A | A | T | G | G | A | C | C | A | A | C | C | A | C | A | G | G | A | A | G | G | C | C | A | A | G | A | C | G | A | G | T | A | C | A | T | C | T | T | C | G | A | A | G | A | A | G | A | A | G | G | T | A | C | A | T | A | T | G | C | A | C | A | T | G | A | C | G | T | T | G | C | A | A | T | A | G | A | C | T | C | T |  | : |  |  | 1 | 7 | 1 |
| M | T | 0 | 1 | 2 | 7 | 3 | 2 |  | : |  | T | G | G | T | A | T | C | A | G | A | G | C | C | T | G | C | T | A | C | T | C | T | C | A | C | C | A | G | T | A | G | G | A | G | A | C | C | C | A | T | G | G | A | G | A | C | C | A | C | C | T | C | T | T | C | A | A | C | A | A | C | A | T | C | T | C | A | G | A | A | T | G | T | C | T | G | A | A | G | A | A | G | A | G | A | T | C | A | G | A | A | T | G | G | A | C | C | A | A | C | C | A | C | A | G | G | A | A | G | G | C | C | A | A | G | A | C | G | A | G | C | A | C | A | T | C | T | T | C | G | A | A | G | A | A | G | A | A | G | G | T | A | C | A | T | A | T | G | C | A | C | A | T | G | A | C | G | T | T | G | C | A | A | T | A | G | A | C | T | C | T |  | : |  |  | 1 | 7 | 1 |
| M | T | 0 | 1 | 2 | 7 | 3 | 4 |  | : |  | T | G | G | T | A | T | C | A | G | A | G | C | C | T | G | C | T | A | C | T | C | T | C | A | C | C | A | G | T | A | G | G | A | G | A | T | C | C | T | C | A | G | A | G | A | C | C | A | C | C | T | C | T | T | C | A | A | C | A | A | C | A | T | C | T | C | A | G | A | A | T | G | T | C | T | G | A | A | G | A | G | G | A | G | A | T | C | A | G | A | A | T | G | G | A | C | C | A | A | C | C | A | C | A | G | G | A | A | G | G | C | C | A | A | G | A | C | G | A | G | T | A | C | A | T | C | T | T | C | G | A | A | G | A | A | G | A | A | G | G | T | A | C | A | T | A | T | G | C | A | C | A | T | G | A | C | G | T | T | G | C | A | A | T | A | G | A | C | T | C | T |  | : |  |  | 1 | 7 | 1 |
| M | T | 0 | 2 | 7 | 0 | 0 | 6 |  | : |  | T | G | G | T | A | T | C | A | G | A | G | C | C | T | G | C | T | A | C | T | C | T | C | A | C | C | A | G | T | A | G | G | A | G | A | C | C | C | A | T | G | G | A | G | A | C | C | A | C | C | T | T | T | T | C | A | A | C | A | A | C | A | T | C | T | C | A | G | A | A | T | G | T | C | T | G | A | A | G | A | A | G | A | G | A | T | C | A | G | A | A | T | G | G | A | C | C | A | A | C | C | A | C | A | A | G | A | A | G | G | C | C | A | A | G | A | C | G | A | G | T | A | C | A | T | C | T | T | C | G | A | A | G | A | A | G | A | A | G | G | T | A | C | A | T | A | T | G | C | G | C | A | T | G | A | C | G | T | T | G | C | A | A | T | A | G | A | C | T | C | T |  | : |  |  | 1 | 7 | 1 |
| M | T | 0 | 2 | 7 | 0 | 0 | 7 |  | : |  | T | G | G | T | A | T | C | A | G | A | G | C | C | T | G | C | T | A | C | T | C | T | C | A | C | C | A | G | T | A | G | G | A | G | A | T | C | C | T | C | A | G | A | G | G | C | C | A | C | C | T | C | T | T | C | A | A | C | A | A | C | A | T | C | A | C | A | G | A | A | T | G | T | C | T | G | A | A | G | A | A | G | A | G | A | T | C | A | G | A | A | T | G | G | A | C | C | A | A | C | C | A | C | A | G | G | A | A | G | G | C | C | A | A | G | A | C | G | A | G | T | A | C | A | T | C | T | T | C | G | A | A | G | A | A | G | A | A | G | G | T | A | C | A | T | A | T | G | C | A | C | A | T | G | A | C | G | T | T | G | C | A | A | T | A | G | A | C | T | C | T |  | : |  |  | 1 | 7 | 1 |
| M | T | 0 | 3 | 6 | 0 | 5 | 3 |  | : |  | T | G | G | T | A | T | C | A | G | A | G | C | C | T | G | C | T | A | C | T | C | T | C | A | C | C | A | G | T | A | G | G | A | G | A | T | C | C | T | C | A | G | A | G | A | C | C | A | C | T | T | C | T | T | C | A | A | C | A | A | C | A | T | C | A | C | A | G | A | A | T | G | T | C | T | G | A | A | G | A | A | G | A | G | A | T | C | A | G | A | A | T | G | G | A | C | C | A | A | C | C | A | C | A | G | G | A | A | G | G | C | C | A | A | G | A | C | G | A | G | T | A | C | A | T | C | T | T | C | G | A | A | G | A | A | G | A | A | G | G | T | A | C | A | T | A | T | G | C | A | C | A | T | G | A | C | G | T | T | G | C | A | A | T | A | G | A | C | T | C | T |  | : |  |  | 1 | 7 | 1 |
| M | T | 0 | 3 | 6 | 0 | 5 | 4 |  | : |  | T | G | G | T | A | T | C | A | G | A | G | C | C | T | G | C | T | A | C | T | C | T | C | A | C | C | A | G | T | A | G | G | A | G | A | T | C | C | T | C | A | G | A | G | A | C | C | A | C | C | T | C | T | T | C | A | A | C | A | A | C | A | T | C | A | C | A | G | A | A | T | G | T | C | T | G | A | A | G | A | A | G | A | G | A | T | C | A | G | A | A | T | G | G | A | C | C | A | A | C | C | A | C | A | G | G | A | A | G | G | C | C | A | A | G | A | C | G | A | G | T | A | C | A | T | C | T | T | C | G | A | A | G | A | A | G | A | A | G | G | T | A | C | A | T | A | T | G | C | A | C | A | T | G | A | C | G | T | T | G | C | A | A | T | A | G | A | C | T | C | T |  | : |  |  | 1 | 7 | 1 |
| M | T | 0 | 3 | 6 | 0 | 5 | 5 |  | : |  | T | G | G | T | A | T | C | A | G | A | G | C | C | T | G | C | T | A | C | T | C | T | C | A | C | C | A | G | T | A | G | G | A | G | A | T | C | C | T | C | A | G | A | G | A | C | C | A | C | C | T | C | T | T | C | A | A | C | A | A | C | A | T | C | A | C | A | G | C | A | T | G | T | C | T | G | A | A | G | A | A | G | A | G | A | T | C | A | G | A | A | T | G | G | A | C | C | A | A | C | C | A | C | A | G | G | A | A | G | G | C | C | A | A | G | A | C | G | A | G | T | A | C | A | T | C | T | T | C | G | A | A | G | A | A | G | A | A | G | G | T | A | C | A | T | A | T | G | C | A | C | A | T | G | A | C | G | T | T | G | C | A | A | T | A | G | A | C | T | C | T |  | : |  |  | 1 | 7 | 1 |
| M | T | 0 | 3 | 6 | 0 | 5 | 6 |  | : |  | T | G | G | T | A | T | C | A | G | A | G | C | C | T | G | C | T | A | C | T | C | T | C | A | C | C | A | G | T | A | G | G | A | G | A | C | C | C | A | T | G | G | A | G | A | C | C | A | C | C | T | T | T | T | C | A | A | C | A | A | C | A | T | C | T | C | A | G | A | A | T | G | T | C | T | G | A | A | G | A | A | G | A | G | A | T | C | A | G | A | A | T | G | G | A | C | C | A | A | C | C | A | C | A | G | G | A | A | G | G | C | C | A | A | G | A | C | G | A | G | T | A | C | A | T | C | T | T | C | G | A | A | G | A | A | G | A | A | G | G | T | A | C | A | T | A | T | G | C | A | C | A | T | G | A | C | G | T | T | G | C | A | A | T | A | G | A | C | T | C | T |  | : |  |  | 1 | 7 | 1 |
| M | T | 0 | 3 | 6 | 0 | 5 | 7 |  | : |  | T | G | G | T | A | T | C | A | G | A | G | C | C | T | G | C | T | A | C | T | C | T | C | A | C | C | A | G | T | A | G | G | A | G | A | C | C | C | A | T | G | G | A | G | A | C | C | A | C | C | T | C | T | T | C | A | A | C | A | A | C | A | T | C | T | C | A | G | A | A | T | G | T | C | T | G | A | A | G | A | A | G | A | G | A | T | C | A | G | A | A | T | G | G | A | C | C | A | A | C | C | A | C | A | G | G | A | A | G | G | C | C | A | A | G | A | C | G | A | G | T | A | C | A | T | C | T | T | C | G | A | A | G | A | A | G | A | A | G | G | T | A | C | A | T | A | T | G | C | A | C | A | T | G | A | C | G | T | T | G | C | A | A | T | A | G | A | C | T | C | T |  | : |  |  | 1 | 7 | 1 |
| K | X | 2 | 4 | 9 | 7 | 3 | 8 |  | : |  | T | G | G | T | A | T | C | A | G | A | G | C | C | T | G | C | T | A | C | T | C | T | C | A | C | C | A | G | T | A | G | G | A | G | A | C | C | C | A | T | G | G | A | G | A | C | C | A | C | C | T | C | T | T | C | A | A | C | A | A | C | A | T | C | T | C | A | G | A | A | T | G | T | C | T | G | A | A | G | A | A | G | A | G | A | T | C | A | G | A | A | T | G | G | A | C | C | A | A | C | C | A | C | A | G | G | A | A | G | G | C | C | A | A | G | A | C | G | A | G | T | A | C | A | T | C | T | T | C | G | A | A | G | A | A | G | A | A | G | G | T | A | C | A | T | A | T | G | C | A | C | A | T | G | A | C | G | T | T | G | C | A | A | T | A | G | A | C | T | C | T |  | : |  |  | 1 | 7 | 1 |
| K | X | 2 | 4 | 9 | 7 | 3 | 7 |  | : |  | T | G | G | T | A | T | C | A | G | A | G | C | C | T | G | C | T | A | C | T | C | T | C | A | C | C | A | G | T | A | G | G | A | G | A | C | C | C | A | T | G | G | A | G | A | C | C | A | C | C | T | C | T | T | C | A | A | C | A | A | C | A | T | C | T | C | A | G | A | A | T | G | T | C | T | G | A | A | G | A | A | G | A | G | A | T | C | A | G | A | A | T | G | G | A | C | C | A | A | C | C | A | C | A | G | G | A | A | G | G | C | C | A | A | G | A | C | G | A | G | T | A | C | A | T | C | T | T | C | G | A | A | G | A | A | G | A | A | G | G | T | A | C | A | T | A | T | G | C | A | C | A | T | G | A | C | G | T | T | G | C | A | A | T | A | G | A | C | T | C | T |  | : |  |  | 1 | 7 | 1 |
| K | X | 2 | 4 | 9 | 7 | 3 | 6 |  | : |  | T | G | G | T | A | T | C | A | G | A | G | C | C | T | G | C | T | A | C | T | C | T | C | A | C | C | A | G | T | A | G | G | A | G | A | C | C | C | A | T | G | G | A | G | A | C | C | A | C | C | T | C | T | T | C | A | A | C | A | A | C | A | T | C | T | C | A | G | A | A | T | G | T | C | T | G | A | A | G | A | A | G | A | G | A | T | C | A | G | A | A | T | G | G | A | C | C | A | A | C | C | A | C | A | G | G | A | A | G | G | C | C | A | A | G | A | C | G | A | G | T | A | C | A | T | C | T | T | C | G | A | A | G | A | A | G | A | A | G | G | T | A | C | A | T | A | T | G | C | A | C | A | T | G | A | C | G | T | T | G | C | A | A | T | A | G | A | C | T | C | T |  | : |  |  | 1 | 7 | 1 |
| K | X | 2 | 4 | 9 | 7 | 3 | 5 |  | : |  | T | G | G | T | A | T | C | A | G | A | G | C | C | T | G | C | T | A | C | T | C | T | C | A | C | C | A | G | T | A | G | G | A | G | A | C | C | C | A | T | G | G | A | G | A | C | C | A | C | C | T | C | T | T | C | A | A | C | A | A | C | A | T | C | T | C | A | G | A | A | T | G | T | C | T | G | A | A | G | A | A | G | A | G | A | T | C | A | G | A | A | T | G | G | A | C | C | A | A | C | C | A | C | A | G | G | A | A | G | G | C | C | A | A | G | A | C | G | A | G | T | A | C | A | T | C | T | T | C | G | A | A | G | A | A | G | A | A | G | G | T | A | C | A | T | A | T | G | C | A | C | A | T | G | A | C | G | T | T | G | C | A | A | T | A | G | A | C | T | C | T |  | : |  |  | 1 | 7 | 1 |
| M | F | 1 | 9 | 7 | 9 | 1 | 6 |  | : |  | T | G | G | T | A | T | C | A | G | A | G | C | C | T | G | C | T | A | C | T | C | T | C | A | C | C | A | G | T | A | G | G | A | G | A | C | C | C | A | T | G | G | A | G | A | C | C | A | C | C | T | C | T | T | C | A | A | C | A | A | C | A | T | C | T | C | A | G | A | A | T | G | T | C | T | G | A | A | G | A | A | G | A | G | A | T | C | A | G | A | A | T | G | G | A | C | C | A | A | C | C | A | C | A | G | G | A | A | G | G | C | C | A | A | G | A | C | G | A | G | T | A | C | A | T | C | T | T | C | G | A | A | G | A | A | G | A | A | G | G | T | A | C | A | T | A | T | G | C | A | C | A | T | G | A | C | G | T | T | G | C | A | A | T | A | G | A | C | T | C | T |  | : |  |  | 1 | 7 | 1 |
| K | T | 2 | 5 | 0 | 6 | 3 | 2 |  | : |  | T | G | G | T | A | T | C | A | G | A | G | C | C | T | G | C | T | A | C | T | C | T | C | A | C | C | A | G | T | A | G | G | A | G | A | T | C | C | T | C | A | G | A | G | A | C | C | A | C | C | T | C | T | T | C | A | A | C | A | A | C | A | T | C | A | C | A | G | A | A | T | G | T | C | T | G | A | A | G | A | A | G | A | G | A | T | C | A | G | A | A | T | G | G | A | C | C | A | A | C | C | A | C | A | A | G | A | A | G | G | C | C | A | A | G | A | C | G | A | G | T | A | C | A | T | C | T | T | C | G | A | A | G | A | A | G | A | A | G | G | T | A | C | A | T | A | T | G | C | A | C | A | T | G | A | C | G | T | T | G | C | A | A | T | A | G | A | C | T | C | T |  | : |  |  | 1 | 7 | 1 |
|  |  |  |  |  |  |  |  |  |  |  | T | G | G | T | A | T | C | A | G | A | G | C | C | T | G | C | T | A | C | T | C | T | C | A | C | C | A | G | T | A | G | G | A | G | A |  | C | C |  |  |  | G | A | G | a | C | C | A | C | c | T | c | T | T | C | A | A | C | A | A | C | A | T | C |  | c | A | G | a | A | T | G | T | C | T | G | A | A | G | A | a | G | A | G | A | T | C | A | G | A | A | T | G | G | A | C | C | A | A | C | C | A | C | A | g | G | A | A | G | G | C | C | A | A | G | A | C | G | A | G | t | A | C | A | T | C | T | T | C | G | A | A | G | A | A | G | A | A | G | G | T | A | C | A | T | A | T | G | C | a | C | A | T | G | A | C | G | T | T | G | C | A | A | T | A | G | A | C | T | C | T |  |  |  |  |  |  |  |

|  |  |  |  |  |  |  |  |  |  |  |  |  |  |  |  |  |  |  |  |  |  |  |  |  |  |  |  |  |  |  |  |  |  |  |  |  |  |  |  |  |  |  |  |  |  |  |  |  |  |  |  |  |  |  |  |  |  |  |  |  |  |  |  |  |  |  |  |  |  |  |  |  |  |  |  |  |  |  |  |  |  |  |  |  |  |  |  |  |  |  |  |  |  |  |  |  |  |  |  |  |  |  |  |  |  |  |  |  |  |  |  |  |  |  |  |  |  |  |  |  |  |  |  |  |  |  |  |  |  |  |  |  |  |  |  |  |  |  |  |  |  |  |  |  |  |  |  |  |  |  |  |  |  |  |  |  |  |  |  |  |  |  |  |  |  |  |  |  |  |  |  |  |  |  |  |  |  |  |  |  |  |  |  |  |  |  |  |  |
| --- | --- | --- | --- | --- | --- | --- | --- | --- | --- | --- | --- | --- | --- | --- | --- | --- | --- | --- | --- | --- | --- | --- | --- | --- | --- | --- | --- | --- | --- | --- | --- | --- | --- | --- | --- | --- | --- | --- | --- | --- | --- | --- | --- | --- | --- | --- | --- | --- | --- | --- | --- | --- | --- | --- | --- | --- | --- | --- | --- | --- | --- | --- | --- | --- | --- | --- | --- | --- | --- | --- | --- | --- | --- | --- | --- | --- | --- | --- | --- | --- | --- | --- | --- | --- | --- | --- | --- | --- | --- | --- | --- | --- | --- | --- | --- | --- | --- | --- | --- | --- | --- | --- | --- | --- | --- | --- | --- | --- | --- | --- | --- | --- | --- | --- | --- | --- | --- | --- | --- | --- | --- | --- | --- | --- | --- | --- | --- | --- | --- | --- | --- | --- | --- | --- | --- | --- | --- | --- | --- | --- | --- | --- | --- | --- | --- | --- | --- | --- | --- | --- | --- | --- | --- | --- | --- | --- | --- | --- | --- | --- | --- | --- | --- | --- | --- | --- | --- | --- | --- | --- | --- | --- | --- | --- | --- | --- | --- | --- | --- | --- | --- | --- | --- | --- | --- | --- | --- | --- |
|  |  |  |  |  |  |  |  |  |  |  |  |  |  |  |  |  |  |  |  |  |  |  |  |  |  |  |  |  |  |  |  |  |  |  |  |  |  |  |  |  |  |  |  |  |  |  |  |  |  |  |  |  |  |  |  |  |  |  |  |  |  |  |  |  |  |  |  |  |  |  |  |  |  |  |  |  |  |  |  |  |  |  |  |  |  |  |  |  |  |  |  |  |  |  |  |  |  |  |  |  |  |  |  |  |  |  |  |  |  |  |  |  |  |  |  |  |  |  |  |  |  |  |  |  |  |  |  |  |  |  |  |  |  |  |  |  |  |  |  |  |  |  |  |  |  |  |  |  |  |  |  |  |  |  |  |  |  |  |  |  |  |  |  |  |  |  |  |  |  |  |  |  |  |  |  |  |  |  |  |  |  |  |  |  |  |  |  |  |
|  |  |  |  |  |  |  |  |  |  |  |  |  |  |  |  |  | 1 | 8 | 0 |  |  |  |  |  |  |  |  |  | \* |  |  |  |  |  |  |  | 2 | 0 | 0 |  |  |  |  |  |  |  |  |  | \* |  |  |  |  |  |  |  | 2 | 2 | 0 |  |  |  |  |  |  |  |  |  | \* |  |  |  |  |  |  |  | 2 | 4 | 0 |  |  |  |  |  |  |  |  |  | \* |  |  |  |  |  |  |  | 2 | 6 | 0 |  |  |  |  |  |  |  |  |  | \* |  |  |  |  |  |  |  | 2 | 8 | 0 |  |  |  |  |  |  |  |  |  | \* |  |  |  |  |  |  |  | 3 | 0 | 0 |  |  |  |  |  |  |  |  |  | \* |  |  |  |  |  |  |  | 3 | 2 | 0 |  |  |  |  |  |  |  |  |  | \* |  |  |  |  |  |  |  | 3 | 4 | 0 |  |  |  |  |  |  |  |  |  |
| M | N | 9 | 5 | 6 | 5 | 2 | 0 |  | : |  | T | C | T | C | T | C | T | T | A | G | G | A | C | A | G | A | T | A | G | A | G | A | A | A | A | A | G | G | A | T | C | T | T | G | A | A | C | T | T | T | C | T | A | C | A | G | A | A | G | A | A | G | T | G | T | T | C | A | A | A | T | C | C | C | C | T | A | G | C | C | T | A | T | G | G | A | A | G | A | A | A | T | T | C | C | T | C | A | A | A | G | C | T | A | G | A | A | A | A | A | A | T | A | T | A | T | G | T | G | T | T | G | C | T | T | G | T | G | T | T | A | G | C | A | C | T | A | G | G | G | A | A | T | A | C | C | C | C | A | T | A | G | A | A | A | T | A | A | C | C | C | A | A | G | C | C | A | A | C | G | G | A | C | T | T | A | C | T | G | A | G |  | : |  |  | 3 | 4 | 2 |
| M | T | 0 | 1 | 2 | 7 | 3 | 2 |  | : |  | T | C | T | C | T | C | T | T | A | G | G | A | C | A | G | A | T | A | G | A | G | A | A | A | A | A | G | G | A | T | C | T | T | G | A | A | C | T | T | T | C | T | A | C | A | G | A | A | G | A | A | G | T | G | T | T | C | A | A | A | T | C | C | C | C | T | A | G | C | C | T | A | T | G | G | A | A | G | A | A | A | T | T | C | C | T | C | A | A | A | G | C | T | A | G | A | A | A | A | A | A | T | A | T | A | T | G | T | G | T | T | G | C | T | T | G | T | G | T | T | A | G | C | A | C | T | A | G | G | G | A | A | T | A | C | C | C | C | A | T | A | G | A | A | A | T | A | A | C | C | C | A | A | G | C | C | A | A | C | G | G | A | C | T | T | A | C | T | G | A | G |  | : |  |  | 3 | 4 | 2 |
| M | T | 0 | 1 | 2 | 7 | 3 | 4 |  | : |  | T | C | T | C | T | C | T | T | A | G | G | A | C | A | G | A | T | A | G | A | G | A | A | A | A | A | G | G | A | T | C | T | T | G | A | A | C | T | T | T | C | T | A | C | A | G | A | A | G | A | A | G | T | G | T | T | C | A | A | A | T | C | C | C | C | T | A | G | C | C | T | A | T | G | G | A | A | G | A | A | A | T | T | C | C | T | C | A | A | A | G | C | T | A | G | A | A | A | A | A | A | T | A | T | A | T | G | T | G | T | T | G | C | T | T | G | T | G | T | T | A | G | C | A | C | T | A | G | G | G | A | A | T | A | C | C | C | C | A | T | A | G | A | A | A | T | A | A | C | C | C | A | A | G | C | C | A | A | C | G | G | A | C | T | T | A | C | T | G | A | G |  | : |  |  | 3 | 4 | 2 |
| M | T | 0 | 2 | 7 | 0 | 0 | 6 |  | : |  | T | C | T | C | T | C | T | T | A | G | G | A | C | A | G | A | T | A | G | A | G | A | A | A | A | A | G | G | A | T | C | T | T | G | A | A | C | T | T | T | C | T | A | C | A | G | A | A | G | A | A | G | T | G | T | T | C | A | A | A | T | C | C | C | C | T | A | G | C | C | T | A | T | G | G | A | A | G | A | A | A | T | T | C | C | T | C | A | A | A | G | C | T | A | G | A | A | A | A | A | A | T | A | T | A | T | G | T | G | T | T | G | C | T | T | G | T | G | T | T | A | G | C | A | C | T | A | G | G | G | A | A | T | A | C | C | C | C | A | T | A | G | A | A | A | T | A | A | C | C | C | A | A | G | C | C | A | A | C | G | G | A | C | T | T | A | C | T | G | A | G |  | : |  |  | 3 | 4 | 2 |
| M | T | 0 | 2 | 7 | 0 | 0 | 7 |  | : |  | T | C | T | C | T | C | T | T | A | G | G | A | C | A | G | A | T | A | G | A | G | A | A | A | A | A | G | G | A | T | C | T | T | G | A | A | C | T | T | T | C | T | A | C | A | G | A | A | G | A | A | G | T | G | T | T | C | A | A | A | T | C | C | C | C | T | A | G | C | C | T | A | T | G | G | A | A | G | A | A | A | T | T | C | C | T | C | A | A | A | G | C | T | A | G | A | A | A | A | A | A | T | A | T | A | T | G | T | G | T | T | G | C | T | T | G | T | G | T | T | A | G | C | A | C | T | A | G | G | G | A | A | T | A | C | C | C | C | A | T | A | G | A | A | A | T | A | A | C | C | C | A | A | G | C | C | A | A | C | G | G | A | C | T | T | A | C | T | G | A | G |  | : |  |  | 3 | 4 | 2 |
| M | T | 0 | 3 | 6 | 0 | 5 | 3 |  | : |  | T | C | T | C | T | C | T | T | A | G | G | A | C | A | G | A | T | A | G | A | G | A | A | A | A | A | G | G | A | T | C | T | T | G | A | A | C | T | T | T | C | T | A | C | A | G | A | A | G | A | A | G | T | G | T | T | C | A | A | A | T | C | C | C | C | T | A | G | C | C | T | A | T | G | G | A | A | G | A | A | A | T | T | C | C | T | C | A | A | A | G | C | T | A | G | A | A | A | A | A | A | T | A | T | A | T | G | T | G | T | T | G | C | T | T | G | T | G | T | T | A | G | C | A | C | T | A | G | G | G | A | A | T | A | C | C | C | C | A | T | A | G | A | A | A | T | A | A | C | C | C | A | A | G | C | C | A | A | C | G | G | A | C | T | T | A | C | T | G | A | G |  | : |  |  | 3 | 4 | 2 |
| M | T | 0 | 3 | 6 | 0 | 5 | 4 |  | : |  | T | C | T | C | T | C | T | T | A | G | G | A | C | A | G | A | T | A | G | A | G | A | A | A | A | A | G | G | A | T | C | T | T | G | A | A | C | T | T | T | C | T | A | C | A | G | A | A | G | A | A | G | T | G | T | T | C | A | A | A | T | C | C | C | C | T | A | G | C | C | T | A | T | G | G | A | A | G | A | A | A | T | T | C | C | T | C | A | A | A | G | C | T | A | G | A | A | A | A | A | A | T | A | T | A | T | G | T | G | T | T | G | C | T | T | G | T | G | T | T | A | G | C | A | C | T | A | G | G | G | A | A | T | A | C | C | C | C | A | T | A | G | A | A | A | T | A | A | C | C | C | A | A | G | C | C | A | A | C | G | G | A | C | T | T | A | C | T | G | A | G |  | : |  |  | 3 | 4 | 2 |
| M | T | 0 | 3 | 6 | 0 | 5 | 5 |  | : |  | T | C | T | C | T | C | T | T | A | G | G | A | C | A | G | A | T | A | G | A | G | A | A | A | A | A | G | G | A | T | C | T | T | G | A | A | C | T | T | T | C | T | A | C | A | G | A | A | G | A | A | G | T | G | T | T | C | A | A | A | T | C | C | C | C | T | A | G | C | C | T | A | T | G | G | A | A | G | A | A | A | T | T | C | C | T | C | A | A | A | G | C | T | A | G | A | A | A | A | A | A | T | A | T | A | T | G | T | G | T | T | G | C | T | T | G | T | G | T | T | A | G | C | A | C | T | A | G | G | G | A | A | T | A | C | C | C | C | A | T | A | G | A | A | A | T | A | A | C | C | C | A | A | G | C | C | A | A | C | G | G | A | C | T | T | A | C | T | G | A | G |  | : |  |  | 3 | 4 | 2 |
| M | T | 0 | 3 | 6 | 0 | 5 | 6 |  | : |  | T | C | T | C | T | C | T | T | A | G | G | A | C | A | G | A | T | A | G | A | G | A | A | A | A | A | G | G | A | T | C | T | T | G | A | A | C | T | T | T | C | T | A | C | A | G | A | A | G | A | A | G | T | G | T | T | C | A | A | A | T | C | C | C | C | T | A | G | C | C | T | A | T | G | G | A | A | G | A | A | A | T | T | C | C | T | C | A | A | A | G | C | T | A | G | A | A | A | A | A | A | T | A | T | A | T | G | T | G | T | T | G | C | T | T | G | T | G | T | T | A | G | C | A | C | T | A | G | G | G | A | A | T | A | C | C | C | C | A | T | A | G | A | A | A | T | A | A | C | C | C | A | A | G | C | C | A | A | C | G | G | A | C | T | T | A | C | T | G | A | G |  | : |  |  | 3 | 4 | 2 |
| M | T | 0 | 3 | 6 | 0 | 5 | 7 |  | : |  | T | C | T | C | T | C | T | T | A | G | G | A | C | A | G | A | T | A | G | A | G | A | A | A | A | A | G | G | A | T | C | T | T | G | A | A | C | T | T | T | C | T | A | C | A | G | A | A | G | A | A | G | T | G | T | T | C | A | A | A | T | C | C | C | C | T | A | G | C | C | T | A | T | G | G | A | A | G | A | A | A | T | T | C | C | T | C | A | A | A | G | C | T | A | G | A | A | A | A | A | A | T | A | T | A | T | G | T | G | T | T | G | C | T | T | G | T | G | T | T | A | G | C | A | C | T | A | G | G | G | A | A | T | A | C | C | C | C | A | T | A | G | A | A | A | T | A | A | C | C | C | A | A | G | C | C | A | A | C | G | G | A | C | T | T | A | C | T | G | A | G |  | : |  |  | 3 | 4 | 2 |
| K | X | 2 | 4 | 9 | 7 | 3 | 8 |  | : |  | T | C | T | C | T | C | T | T | A | G | G | A | C | A | G | A | T | A | G | A | G | A | A | A | A | A | G | G | A | T | C | T | T | G | A | A | C | T | T | T | C | T | A | C | A | G | A | A | G | A | A | G | T | G | T | T | C | A | A | A | T | C | C | C | C | T | A | G | C | A | T | A | T | G | G | A | A | G | A | A | A | T | T | C | C | T | C | A | A | A | G | C | T | A | G | A | A | A | A | A | A | T | A | T | A | T | G | T | G | T | T | G | C | T | T | G | T | G | T | T | A | G | C | A | C | T | A | G | G | G | A | A | T | A | C | C | C | C | A | T | A | G | A | A | A | T | A | A | C | C | C | A | A | G | C | C | A | A | C | G | G | A | C | T | T | A | C | T | G | A | G |  | : |  |  | 3 | 4 | 2 |
| K | X | 2 | 4 | 9 | 7 | 3 | 7 |  | : |  | T | C | T | C | T | C | T | T | A | G | G | A | C | A | G | A | T | A | G | A | G | A | A | A | A | A | G | G | A | T | C | T | T | G | A | A | C | T | T | T | C | T | A | C | A | G | A | A | G | A | A | G | T | G | T | T | C | A | A | A | T | C | C | C | C | T | A | G | C | A | T | A | T | G | G | A | A | G | A | A | A | T | T | C | C | T | C | A | A | A | G | C | T | A | G | A | A | A | A | A | A | T | A | T | A | T | G | T | G | T | T | G | C | T | T | G | T | G | T | T | A | G | C | A | C | T | A | G | G | G | A | A | T | A | C | C | C | C | A | T | A | G | A | A | A | T | A | A | C | C | C | A | A | G | C | C | A | A | C | G | G | A | C | T | T | A | C | T | G | A | G |  | : |  |  | 3 | 4 | 2 |
| K | X | 2 | 4 | 9 | 7 | 3 | 6 |  | : |  | T | C | T | C | T | C | T | T | A | G | G | A | C | A | G | A | T | A | G | A | G | A | A | A | A | A | G | G | A | T | C | T | T | G | A | A | C | T | T | T | C | T | A | C | A | G | G | A | G | A | A | G | T | G | T | T | C | A | A | A | T | C | C | C | C | T | A | G | C | C | T | A | T | G | G | A | A | G | A | A | A | T | T | C | C | T | C | A | A | A | G | C | T | A | G | A | A | A | A | A | A | T | A | T | A | T | G | T | G | T | T | G | C | T | T | G | T | G | T | T | A | G | C | A | C | T | A | G | G | G | A | A | T | A | C | C | C | C | A | T | A | G | A | A | A | T | A | A | C | C | C | A | A | G | C | C | A | A | C | G | G | A | C | T | T | A | C | T | G | A | G |  | : |  |  | 3 | 4 | 2 |
| K | X | 2 | 4 | 9 | 7 | 3 | 5 |  | : |  | T | C | T | C | T | C | T | T | A | G | G | A | C | A | G | A | T | A | G | A | G | A | A | A | A | A | G | G | A | T | C | T | T | G | A | A | C | T | T | T | C | T | A | C | A | G | A | A | G | A | A | G | T | G | T | T | C | A | A | A | T | C | C | C | C | T | A | G | C | C | T | A | T | G | G | A | A | G | A | A | A | T | T | C | C | T | C | A | A | A | G | C | T | A | G | A | A | A | A | A | A | T | A | T | A | T | G | T | G | T | T | G | C | T | T | G | T | G | T | T | A | G | C | A | C | T | A | G | G | G | A | A | T | A | C | C | C | C | A | T | A | G | A | A | A | T | A | A | C | C | C | A | A | G | C | C | A | A | C | G | G | A | C | T | T | A | C | T | G | A | G |  | : |  |  | 3 | 4 | 2 |
| M | F | 1 | 9 | 7 | 9 | 1 | 6 |  | : |  | T | C | T | C | T | C | T | T | A | G | G | A | C | A | G | A | T | A | G | A | G | A | A | A | A | A | G | G | A | T | C | T | T | G | A | A | C | T | T | T | C | T | A | C | A | G | A | A | G | A | A | G | T | G | T | T | C | A | A | A | T | C | C | C | C | T | A | G | C | C | T | A | T | G | G | A | A | G | A | A | A | T | T | C | C | T | C | A | A | A | G | C | T | A | G | A | A | A | A | A | A | T | A | T | A | T | G | T | G | T | T | G | C | T | T | G | T | G | T | T | A | G | C | A | C | T | A | G | G | G | A | A | T | A | C | C | C | C | A | T | A | G | A | A | A | T | A | A | C | C | C | A | A | G | C | C | A | A | C | G | G | A | C | T | T | A | C | T | G | A | G |  | : |  |  | 3 | 4 | 2 |
| K | T | 2 | 5 | 0 | 6 | 3 | 2 |  | : |  | T | C | T | C | T | C | T | T | A | G | G | A | C | A | G | A | T | A | G | A | G | A | A | A | A | A | G | G | A | T | C | T | T | G | A | A | C | T | T | T | C | T | A | C | A | G | A | A | G | A | A | G | T | G | T | T | C | A | A | A | T | C | C | C | C | T | A | G | C | C | T | A | T | G | G | A | A | G | A | A | A | T | T | C | C | T | C | A | A | A | G | C | T | A | G | A | A | A | A | A | A | T | A | T | A | T | G | T | G | T | T | G | C | T | T | G | T | G | T | T | A | G | C | A | C | T | A | G | G | G | A | A | T | A | C | C | C | C | A | T | A | G | A | A | A | T | A | A | C | C | C | A | A | G | C | C | A | A | C | G | G | A | C | T | T | A | C | T | G | A | G |  | : |  |  | 3 | 4 | 2 |
|  |  |  |  |  |  |  |  |  |  |  | T | C | T | C | T | C | T | T | A | G | G | A | C | A | G | A | T | A | G | A | G | A | A | A | A | A | G | G | A | T | C | T | T | G | A | A | C | T | T | T | C | T | A | C | A | G | a | A | G | A | A | G | T | G | T | T | C | A | A | A | T | C | C | C | C | T | A | G | C | c | T | A | T | G | G | A | A | G | A | A | A | T | T | C | C | T | C | A | A | A | G | C | T | A | G | A | A | A | A | A | A | T | A | T | A | T | G | T | G | T | T | G | C | T | T | G | T | G | T | T | A | G | C | A | C | T | A | G | G | G | A | A | T | A | C | C | C | C | A | T | A | G | A | A | A | T | A | A | C | C | C | A | A | G | C | C | A | A | C | G | G | A | C | T | T | A | C | T | G | A | G |  |  |  |  |  |  |  |

|  |  |  |  |  |  |  |  |  |  |  |  |  |  |  |  |  |  |  |  |  |  |  |  |  |  |  |  |  |  |  |  |  |  |  |  |  |  |  |  |  |  |  |  |  |  |  |  |  |  |  |  |  |  |  |  |  |  |  |  |  |  |  |  |  |  |  |  |  |  |  |  |  |  |  |  |  |  |  |  |  |  |  |  |  |  |  |  |  |  |  |  |  |  |  |  |  |  |  |  |  |  |  |  |  |  |  |  |  |  |  |  |  |  |  |  |  |  |  |  |  |  |  |  |  |  |  |  |  |  |  |  |  |  |  |  |  |  |  |  |  |  |  |  |  |  |  |  |  |  |  |  |  |  |  |  |  |  |  |  |  |  |  |  |  |  |  |  |  |  |  |  |  |  |  |  |  |  |  |  |  |  |  |  |  |  |  |  |  |
| --- | --- | --- | --- | --- | --- | --- | --- | --- | --- | --- | --- | --- | --- | --- | --- | --- | --- | --- | --- | --- | --- | --- | --- | --- | --- | --- | --- | --- | --- | --- | --- | --- | --- | --- | --- | --- | --- | --- | --- | --- | --- | --- | --- | --- | --- | --- | --- | --- | --- | --- | --- | --- | --- | --- | --- | --- | --- | --- | --- | --- | --- | --- | --- | --- | --- | --- | --- | --- | --- | --- | --- | --- | --- | --- | --- | --- | --- | --- | --- | --- | --- | --- | --- | --- | --- | --- | --- | --- | --- | --- | --- | --- | --- | --- | --- | --- | --- | --- | --- | --- | --- | --- | --- | --- | --- | --- | --- | --- | --- | --- | --- | --- | --- | --- | --- | --- | --- | --- | --- | --- | --- | --- | --- | --- | --- | --- | --- | --- | --- | --- | --- | --- | --- | --- | --- | --- | --- | --- | --- | --- | --- | --- | --- | --- | --- | --- | --- | --- | --- | --- | --- | --- | --- | --- | --- | --- | --- | --- | --- | --- | --- | --- | --- | --- | --- | --- | --- | --- | --- | --- | --- | --- | --- | --- | --- | --- | --- | --- | --- | --- | --- | --- | --- | --- | --- | --- | --- | --- |
|  |  |  |  |  |  |  |  |  |  |  |  |  |  |  |  |  |  |  |  |  |  |  |  |  |  |  |  |  |  |  |  |  |  |  |  |  |  |  |  |  |  |  |  |  |  |  |  |  |  |  |  |  |  |  |  |  |  |  |  |  |  |  |  |  |  |  |  |  |  |  |  |  |  |  |  |  |  |  |  |  |  |  |  |  |  |  |  |  |  |  |  |  |  |  |  |  |  |  |  |  |  |  |  |  |  |  |  |  |  |  |  |  |  |  |  |  |  |  |  |  |  |  |  |  |  |  |  |  |  |  |  |  |  |  |  |  |  |  |  |  |  |  |  |  |  |  |  |  |  |  |  |  |  |  |  |  |  |  |  |  |  |  |  |  |  |  |  |  |  |  |  |  |  |  |  |  |  |  |  |  |  |  |  |  |  |  |  |  |
|  |  |  |  |  |  |  |  |  |  |  |  |  |  |  |  |  |  | \* |  |  |  |  |  |  |  | 3 | 6 | 0 |  |  |  |  |  |  |  |  |  | \* |  |  |  |  |  |  |  | 3 | 8 | 0 |  |  |  |  |  |  |  |  |  | \* |  |  |  |  |  |  |  | 4 | 0 | 0 |  |  |  |  |  |  |  |  |  | \* |  |  |  |  |  |  |  | 4 | 2 | 0 |  |  |  |  |  |  |  |  |  | \* |  |  |  |  |  |  |  | 4 | 4 | 0 |  |  |  |  |  |  |  |  |  | \* |  |  |  |  |  |  |  | 4 | 6 | 0 |  |  |  |  |  |  |  |  |  | \* |  |  |  |  |  |  |  | 4 | 8 | 0 |  |  |  |  |  |  |  |  |  | \* |  |  |  |  |  |  |  | 5 | 0 | 0 |  |  |  |  |  |  |  |  |  | \* |  |  |  |  |  |  |  |  |  |  |
| M | N | 9 | 5 | 6 | 5 | 2 | 0 |  | : |  | A | T | T | C | C | T | T | T | C | T | T | T | A | A | C | C | G | A | G | A | A | G | A | A | A | T | C | G | A | G | A | G | T | A | A | G | A | A | A | A | G | G | G | T | G | T | T | A | A | A | A | C | C | C | G | A | A | G | A | T | C | G | T | A | A | G | A | A | G | A | T | A | G | A | T | T | T | T | A | T | C | C | A | T | A | T | A | G | G | A | T | C | T | A | T | T | A | G | G | A | T | T | A | T | G | A | T | C | A | A | A | A | G | C | A | C | C | T | T | T | A | G | G | A | C | C | G | G | G | A | T | A | G | A | C | G | C | T | C | C | T | A | T | A | A | G | T | G | T | A | G | C | C | C | T | C | C | T | T | G | A | C | C | G | G | A | G | A | A | T | G |  | : |  |  | 5 | 1 | 3 |
| M | T | 0 | 1 | 2 | 7 | 3 | 2 |  | : |  | A | T | T | C | C | T | T | T | C | T | T | T | A | A | C | C | G | A | G | A | A | G | A | A | A | T | C | G | A | G | A | G | T | A | A | G | A | A | A | A | G | G | G | T | G | T | T | A | A | A | A | C | C | C | G | A | A | G | A | T | C | G | T | A | A | G | A | A | G | A | T | A | G | A | T | T | T | T | A | T | C | C | A | T | A | T | A | G | G | A | T | C | T | A | T | T | A | G | G | A | T | T | A | T | G | A | T | C | A | A | A | A | G | C | A | C | C | T | T | T | A | G | G | A | C | C | G | G | G | A | T | A | G | A | C | G | C | T | C | C | T | A | T | A | A | G | T | G | T | A | G | C | C | C | T | C | C | T | T | G | A | C | C | G | G | A | G | A | A | T | G |  | : |  |  | 5 | 1 | 3 |
| M | T | 0 | 1 | 2 | 7 | 3 | 4 |  | : |  | A | T | T | C | C | T | T | T | C | T | T | T | A | A | C | C | G | A | G | A | A | G | A | A | A | T | C | G | A | G | A | G | T | A | A | G | A | A | A | A | G | G | G | T | G | T | T | A | A | A | A | C | C | C | G | A | A | G | A | C | C | G | T | A | A | A | A | A | G | A | T | A | G | A | T | T | T | T | A | T | C | C | A | T | A | T | A | G | G | A | T | C | T | A | T | T | A | G | G | A | T | T | A | T | G | A | T | C | A | A | A | A | G | C | A | C | C | T | T | T | A | G | G | A | C | C | G | G | G | A | T | A | G | A | C | G | C | T | C | C | T | A | T | A | A | G | T | G | T | A | G | C | C | C | T | C | C | T | T | G | A | C | C | G | G | A | G | A | A | T | G |  | : |  |  | 5 | 1 | 3 |
| M | T | 0 | 2 | 7 | 0 | 0 | 6 |  | : |  | A | T | T | C | C | T | T | T | C | T | T | T | A | A | C | C | G | A | G | A | A | G | A | A | A | T | C | G | A | G | A | G | T | A | A | G | A | A | A | A | G | G | G | T | G | T | T | A | A | A | A | C | C | C | G | A | A | G | A | T | C | G | T | A | A | G | A | A | G | A | T | A | G | A | T | T | T | T | A | T | C | C | A | T | A | T | A | G | G | A | T | C | T | A | T | T | A | G | G | A | T | T | A | T | G | A | T | C | A | A | A | A | G | C | A | C | C | T | T | T | A | G | G | A | C | C | G | G | G | A | T | A | G | A | C | G | C | T | C | C | T | A | T | A | A | G | T | G | T | A | G | C | C | C | T | C | C | T | T | G | A | C | C | G | G | A | G | A | A | T | G |  | : |  |  | 5 | 1 | 3 |
| M | T | 0 | 2 | 7 | 0 | 0 | 7 |  | : |  | A | T | T | C | C | T | T | T | C | T | T | C | A | A | C | C | G | A | G | A | A | G | A | A | A | T | T | G | A | G | A | G | T | A | A | G | A | A | A | A | G | G | G | T | G | T | T | A | A | A | A | C | C | C | G | A | A | G | A | T | C | G | T | A | A | A | A | A | G | A | T | A | G | A | T | T | T | T | A | T | C | C | A | T | A | T | A | G | G | A | T | C | T | A | T | T | A | G | G | A | T | T | A | T | G | A | T | C | A | A | A | A | G | C | A | C | C | T | T | T | A | G | G | A | C | C | G | G | G | A | T | A | G | A | C | G | C | T | C | C | T | A | T | A | A | G | T | G | T | A | G | C | C | C | T | C | C | T | T | G | A | C | C | G | G | A | G | A | A | T | G |  | : |  |  | 5 | 1 | 3 |
| M | T | 0 | 3 | 6 | 0 | 5 | 3 |  | : |  | A | T | T | C | C | T | T | T | C | T | T | C | A | A | C | C | G | A | G | A | A | G | A | A | A | T | C | G | A | G | A | G | T | A | A | G | A | A | A | A | G | G | G | T | G | T | T | A | A | A | A | C | C | C | G | A | A | G | A | T | C | G | T | A | A | A | A | A | G | A | T | A | G | A | T | T | T | T | A | T | C | C | A | T | A | T | A | G | G | A | T | C | T | A | T | T | A | G | G | A | T | T | A | T | G | A | T | C | A | A | A | A | G | C | A | C | C | T | T | T | A | G | G | A | C | C | G | G | G | A | T | A | G | A | C | G | C | T | C | C | T | A | T | A | A | G | T | G | T | A | G | C | C | C | T | C | C | T | T | G | A | C | C | G | G | A | G | A | A | T | G |  | : |  |  | 5 | 1 | 3 |
| M | T | 0 | 3 | 6 | 0 | 5 | 4 |  | : |  | A | T | T | C | C | T | T | T | C | T | T | C | A | A | C | C | G | A | G | A | A | G | A | A | A | T | C | G | A | G | A | G | T | A | A | G | A | A | A | A | G | G | G | T | G | T | T | A | A | A | A | C | C | C | G | A | A | G | A | T | C | G | T | A | A | A | A | A | G | A | T | A | G | A | T | T | T | T | A | T | C | C | A | T | A | T | A | G | G | A | T | C | T | A | T | T | A | G | G | A | T | T | A | T | G | A | T | C | A | A | A | A | G | C | A | C | C | T | T | T | A | G | G | A | C | C | G | G | G | A | T | A | G | A | C | G | C | T | C | C | T | A | T | A | A | G | T | G | T | A | G | C | C | C | T | C | C | T | T | G | A | C | C | G | G | A | G | A | A | T | G |  | : |  |  | 5 | 1 | 3 |
| M | T | 0 | 3 | 6 | 0 | 5 | 5 |  | : |  | A | T | T | C | C | T | T | T | C | T | T | T | A | A | C | C | G | A | G | A | A | G | A | A | A | T | C | G | A | A | A | G | T | A | A | G | A | A | A | A | G | G | G | T | G | T | T | A | A | A | A | C | C | C | G | A | A | G | A | T | C | G | T | A | A | G | A | A | G | A | T | A | G | A | T | T | T | T | A | T | C | C | A | T | A | T | A | G | G | A | T | C | T | A | T | T | A | G | G | A | T | T | A | T | G | A | T | C | A | A | A | A | G | C | A | C | C | T | T | T | A | G | G | A | C | C | G | G | G | A | T | A | G | A | C | G | C | T | C | C | T | A | T | A | A | G | T | G | T | A | G | C | C | C | T | C | C | T | T | G | A | C | C | G | G | A | G | A | A | T | G |  | : |  |  | 5 | 1 | 3 |
| M | T | 0 | 3 | 6 | 0 | 5 | 6 |  | : |  | A | T | T | C | C | T | T | T | C | T | T | T | A | A | C | C | G | A | G | A | A | G | A | A | A | T | C | G | A | G | A | G | T | A | A | G | A | A | A | A | G | G | G | T | G | T | T | A | A | A | A | C | C | C | G | A | A | G | A | T | C | G | T | A | A | G | A | A | G | A | T | A | G | A | T | T | T | T | A | T | C | C | A | T | A | T | A | G | G | A | T | C | T | A | T | T | A | G | G | A | T | T | A | T | G | A | T | C | A | A | A | A | G | C | A | C | C | T | T | T | A | G | G | A | C | C | G | G | G | A | T | A | G | A | C | G | C | T | C | C | T | A | T | A | A | G | T | G | T | A | G | C | C | C | T | C | C | T | T | G | A | C | C | G | G | A | G | A | A | T | G |  | : |  |  | 5 | 1 | 3 |
| M | T | 0 | 3 | 6 | 0 | 5 | 7 |  | : |  | A | T | T | C | C | T | T | T | C | T | T | T | A | A | C | C | G | A | G | A | A | G | A | A | A | T | C | G | A | G | A | G | T | A | A | G | A | A | A | A | G | G | G | T | G | T | T | A | A | A | A | C | C | C | G | A | A | G | A | T | C | G | T | A | A | G | A | A | G | A | T | A | G | A | T | T | T | T | A | T | C | C | A | T | A | T | A | G | G | A | T | C | T | A | T | T | A | G | G | A | T | T | A | T | G | A | T | C | A | A | A | A | G | C | A | C | C | T | T | T | A | G | G | A | C | C | G | G | G | A | T | A | G | A | C | G | C | T | C | C | T | A | T | A | A | G | T | G | T | A | G | C | C | C | T | C | C | T | T | G | A | C | C | G | G | A | G | A | A | T | G |  | : |  |  | 5 | 1 | 3 |
| K | X | 2 | 4 | 9 | 7 | 3 | 8 |  | : |  | A | T | T | C | C | T | T | T | C | T | T | T | A | A | C | C | G | A | G | A | A | G | A | A | A | T | C | G | A | G | A | G | T | A | A | G | A | A | A | A | G | G | G | T | G | T | T | A | A | A | A | C | C | C | G | A | A | G | A | T | C | G | T | A | A | G | A | A | G | A | T | A | G | A | T | T | T | T | A | T | C | C | A | T | A | T | A | G | G | A | T | C | T | A | T | T | A | G | G | A | T | T | A | T | G | A | T | C | A | A | A | A | G | C | A | C | C | T | T | T | A | G | G | A | C | C | G | G | G | A | T | A | G | A | C | G | C | T | C | C | T | A | T | A | A | G | T | G | T | A | G | C | C | C | T | C | C | T | T | G | A | C | C | G | G | A | G | A | A | T | G |  | : |  |  | 5 | 1 | 3 |
| K | X | 2 | 4 | 9 | 7 | 3 | 7 |  | : |  | A | T | T | C | C | T | T | T | C | T | T | T | A | A | C | C | G | A | G | A | A | G | A | A | A | T | C | G | A | G | A | G | T | A | A | G | A | A | A | A | G | G | G | T | G | T | T | A | A | A | A | C | C | C | G | A | A | G | A | T | C | G | T | A | A | G | A | A | G | A | T | A | G | A | T | T | T | T | A | T | C | C | A | T | A | T | A | G | G | A | T | C | T | A | T | T | A | G | G | A | T | T | A | T | G | A | T | C | A | A | A | A | G | C | A | C | C | T | T | T | A | G | G | A | C | C | G | G | G | A | T | A | G | A | C | G | C | T | C | C | T | A | T | A | A | G | T | G | T | A | G | C | C | C | T | C | C | T | T | G | A | C | C | G | G | A | G | A | A | T | G |  | : |  |  | 5 | 1 | 3 |
| K | X | 2 | 4 | 9 | 7 | 3 | 6 |  | : |  | A | T | T | C | C | T | T | T | C | T | T | T | A | A | C | C | G | A | G | A | A | G | A | A | A | T | C | G | A | G | A | G | T | A | A | G | A | A | A | A | G | G | G | T | G | T | T | A | A | A | A | C | C | C | G | A | A | G | A | T | C | G | T | A | A | G | A | A | G | A | T | A | G | A | T | T | T | T | A | T | C | C | A | T | A | T | A | G | G | A | T | C | T | A | T | T | A | G | G | A | T | T | A | T | G | A | T | C | A | A | A | A | G | C | A | C | C | T | T | T | A | G | G | A | C | C | G | G | G | A | T | A | G | A | C | G | C | T | C | C | T | A | T | A | A | G | T | G | T | A | G | C | C | C | T | C | C | T | T | G | A | C | C | G | G | A | G | A | A | T | G |  | : |  |  | 5 | 1 | 3 |
| K | X | 2 | 4 | 9 | 7 | 3 | 5 |  | : |  | A | T | T | C | C | T | T | T | C | T | T | T | A | A | C | C | G | A | G | A | A | G | A | A | A | T | C | G | A | G | A | G | T | A | A | G | A | A | A | A | G | G | G | T | G | T | T | A | A | A | A | C | C | C | G | A | A | G | A | T | C | G | T | A | A | G | A | A | G | A | T | A | G | A | T | T | T | T | A | T | C | C | A | T | A | T | A | G | G | A | T | C | T | A | T | T | A | G | G | A | T | T | A | T | G | A | T | C | A | A | A | A | G | C | A | C | C | T | T | T | A | G | G | A | C | C | G | G | G | A | T | A | G | A | C | G | C | T | C | C | T | A | T | A | A | G | T | G | T | A | G | C | C | C | T | C | C | T | T | G | A | C | C | G | G | A | G | A | A | T | G |  | : |  |  | 5 | 1 | 3 |
| M | F | 1 | 9 | 7 | 9 | 1 | 6 |  | : |  | A | T | T | C | C | T | T | T | C | T | T | T | A | A | C | C | G | A | G | A | A | G | A | A | A | T | C | G | A | G | A | G | T | A | A | G | A | A | A | A | G | G | G | T | G | T | T | A | A | A | A | C | C | C | G | A | A | G | A | T | C | G | T | A | A | G | A | A | G | A | T | A | G | A | T | T | T | T | A | T | C | C | A | T | A | T | A | G | G | A | T | C | T | A | T | T | A | G | G | A | T | T | A | T | G | A | T | C | A | A | A | A | G | C | A | C | C | T | T | T | A | G | G | A | C | C | G | G | G | A | T | A | G | A | C | G | C | T | C | C | T | A | T | A | A | G | T | G | T | A | G | C | C | C | T | C | C | T | T | G | A | C | C | G | G | A | G | A | A | T | G |  | : |  |  | 5 | 1 | 3 |
| K | T | 2 | 5 | 0 | 6 | 3 | 2 |  | : |  | A | T | T | C | C | T | T | T | C | T | T | C | A | A | C | C | G | A | G | A | A | G | A | A | A | T | C | G | A | G | A | G | T | A | A | G | A | A | A | A | G | G | G | T | G | T | T | A | A | A | A | C | C | C | G | A | A | G | A | T | C | G | T | A | A | A | A | A | G | A | T | A | G | A | T | T | T | T | A | T | C | C | A | T | A | T | A | G | G | A | T | C | T | A | T | T | A | G | G | A | T | T | A | T | G | A | T | C | A | A | A | A | G | C | A | C | C | T | T | T | A | G | G | A | C | C | G | G | G | A | T | A | G | A | C | G | C | T | C | C | T | A | T | A | A | G | T | G | T | A | G | C | C | C | T | C | C | T | T | G | A | C | C | G | G | A | G | A | A | T | G |  | : |  |  | 5 | 1 | 3 |
|  |  |  |  |  |  |  |  |  |  |  | A | T | T | C | C | T | T | T | C | T | T |  | A | A | C | C | G | A | G | A | A | G | A | A | A | T | c | G | A | g | A | G | T | A | A | G | A | A | A | A | G | G | G | T | G | T | T | A | A | A | A | C | C | C | G | A | A | G | A | t | C | G | T | A | A |  | A | A | G | A | T | A | G | A | T | T | T | T | A | T | C | C | A | T | A | T | A | G | G | A | T | C | T | A | T | T | A | G | G | A | T | T | A | T | G | A | T | C | A | A | A | A | G | C | A | C | C | T | T | T | A | G | G | A | C | C | G | G | G | A | T | A | G | A | C | G | C | T | C | C | T | A | T | A | A | G | T | G | T | A | G | C | C | C | T | C | C | T | T | G | A | C | C | G | G | A | G | A | A | T | G |  |  |  |  |  |  |  |

|  |  |  |  |  |  |  |  |  |  |  |  |  |  |  |  |  |  |  |  |  |  |  |  |  |  |  |  |  |  |  |  |  |  |  |  |  |  |  |  |  |  |  |  |  |  |  |  |  |  |  |  |  |  |  |  |  |  |  |  |  |  |  |  |  |  |  |  |  |  |  |  |  |  |  |  |  |  |  |  |  |  |  |  |  |  |  |  |  |  |  |  |  |  |  |  |  |  |  |  |  |  |  |  |  |  |  |  |  |  |  |  |  |  |  |  |  |  |  |  |  |  |  |  |  |  |  |  |  |  |  |  |  |  |  |  |  |  |  |  |  |  |  |  |  |  |  |  |  |  |  |  |  |  |  |  |  |  |  |  |  |  |  |  |  |  |  |  |  |  |  |  |  |  |  |  |  |  |  |  |  |  |  |  |  |  |  |  |  |
| --- | --- | --- | --- | --- | --- | --- | --- | --- | --- | --- | --- | --- | --- | --- | --- | --- | --- | --- | --- | --- | --- | --- | --- | --- | --- | --- | --- | --- | --- | --- | --- | --- | --- | --- | --- | --- | --- | --- | --- | --- | --- | --- | --- | --- | --- | --- | --- | --- | --- | --- | --- | --- | --- | --- | --- | --- | --- | --- | --- | --- | --- | --- | --- | --- | --- | --- | --- | --- | --- | --- | --- | --- | --- | --- | --- | --- | --- | --- | --- | --- | --- | --- | --- | --- | --- | --- | --- | --- | --- | --- | --- | --- | --- | --- | --- | --- | --- | --- | --- | --- | --- | --- | --- | --- | --- | --- | --- | --- | --- | --- | --- | --- | --- | --- | --- | --- | --- | --- | --- | --- | --- | --- | --- | --- | --- | --- | --- | --- | --- | --- | --- | --- | --- | --- | --- | --- | --- | --- | --- | --- | --- | --- | --- | --- | --- | --- | --- | --- | --- | --- | --- | --- | --- | --- | --- | --- | --- | --- | --- | --- | --- | --- | --- | --- | --- | --- | --- | --- | --- | --- | --- | --- | --- | --- | --- | --- | --- | --- | --- | --- | --- | --- | --- | --- | --- | --- | --- | --- |
|  |  |  |  |  |  |  |  |  |  |  |  |  |  |  |  |  |  |  |  |  |  |  |  |  |  |  |  |  |  |  |  |  |  |  |  |  |  |  |  |  |  |  |  |  |  |  |  |  |  |  |  |  |  |  |  |  |  |  |  |  |  |  |  |  |  |  |  |  |  |  |  |  |  |  |  |  |  |  |  |  |  |  |  |  |  |  |  |  |  |  |  |  |  |  |  |  |  |  |  |  |  |  |  |  |  |  |  |  |  |  |  |  |  |  |  |  |  |  |  |  |  |  |  |  |  |  |  |  |  |  |  |  |  |  |  |  |  |  |  |  |  |  |  |  |  |  |  |  |  |  |  |  |  |  |  |  |  |  |  |  |  |  |  |  |  |  |  |  |  |  |  |  |  |  |  |  |  |  |  |  |  |  |  |  |  |  |  |  |
|  |  |  |  |  |  |  |  |  |  |  |  |  |  |  | 5 | 2 | 0 |  |  |  |  |  |  |  |  |  | \* |  |  |  |  |  |  |  | 5 | 4 | 0 |  |  |  |  |  |  |  |  |  | \* |  |  |  |  |  |  |  | 5 | 6 | 0 |  |  |  |  |  |  |  |  |  | \* |  |  |  |  |  |  |  | 5 | 8 | 0 |  |  |  |  |  |  |  |  |  | \* |  |  |  |  |  |  |  | 6 | 0 | 0 |  |  |  |  |  |  |  |  |  | \* |  |  |  |  |  |  |  | 6 | 2 | 0 |  |  |  |  |  |  |  |  |  | \* |  |  |  |  |  |  |  | 6 | 4 | 0 |  |  |  |  |  |  |  |  |  | \* |  |  |  |  |  |  |  | 6 | 6 | 0 |  |  |  |  |  |  |  |  |  | \* |  |  |  |  |  |  |  | 6 | 8 | 0 |  |  |  |  |  |  |  |  |  |  |  |
| M | N | 9 | 5 | 6 | 5 | 2 | 0 |  | : |  | A | C | A | A | C | C | G | C | T | A | G | A | G | A | C | G | C | A | G | T | T | T | T | C | G | G | C | G | G | C | A | T | G | A | A | A | G | G | T | A | A | C | C | T | T | T | C | C | T | A | T | G | G | A | A | A | A | C | T | T | A | T | C | T | T | T | A | C | A | T | G | T | A | A | T | C | C | T | A | A | G | A | T | A | G | G | A | G | T | C | A | G | C | C | T | T | A | G | A | G | A | T | C | C | T | A | A | G | A | T | T | G | A | C | A | A | A | A | T | C | T | T | A | A | C | A | T | T | A | G | C | T | C | A | C | T | T | T | T | T | C | G | A | G | A | G | A | G | A | A | A | G | C | C | T | A | A | T | G | T | A | C | C | A | A | G | G | G | A | A | C |  | : |  |  | 6 | 8 | 4 |
| M | T | 0 | 1 | 2 | 7 | 3 | 2 |  | : |  | A | C | A | A | C | C | G | C | T | A | G | A | G | A | C | G | C | A | G | T | T | T | T | C | G | G | C | G | G | C | A | T | G | A | A | A | G | G | T | A | A | C | C | T | T | T | C | C | T | A | T | G | G | A | A | A | A | C | T | T | A | T | C | T | T | T | A | C | A | T | G | T | A | A | T | C | C | T | A | A | G | A | T | A | G | G | A | G | T | C | A | G | C | C | T | T | A | G | A | G | A | T | C | C | T | A | A | G | A | T | T | G | A | C | A | A | A | A | T | C | T | T | A | A | C | A | T | T | A | G | C | C | C | A | C | T | T | T | T | T | C | G | A | G | A | G | A | G | A | A | A | G | C | C | T | A | A | T | G | T | A | C | C | A | A | G | G | G | A | A | C |  | : |  |  | 6 | 8 | 4 |
| M | T | 0 | 1 | 2 | 7 | 3 | 4 |  | : |  | A | C | A | A | C | C | G | C | G | A | G | A | G | A | C | G | C | A | G | T | C | T | T | C | G | G | C | G | G | C | A | T | G | A | A | A | G | G | T | A | A | T | C | T | T | T | C | C | T | A | C | G | G | G | A | A | A | C | T | T | A | T | C | T | T | T | A | C | A | T | G | T | A | A | T | C | C | T | A | A | G | A | T | A | G | G | A | G | T | C | A | G | C | C | T | T | A | G | A | G | A | T | C | C | T | A | A | G | A | T | T | G | A | C | A | A | A | A | T | C | T | T | A | A | C | A | T | T | A | G | C | T | C | A | C | T | T | T | T | T | C | G | A | A | A | G | A | G | A | A | A | G | C | C | T | A | A | T | G | C | A | C | C | T | A | G | G | G | A | A | C |  | : |  |  | 6 | 8 | 4 |
| M | T | 0 | 2 | 7 | 0 | 0 | 6 |  | : |  | A | C | A | A | C | C | G | C | T | A | G | A | G | A | C | G | C | A | G | T | T | T | T | C | G | G | C | G | G | C | A | T | G | A | A | A | G | G | T | A | A | C | C | T | T | T | C | C | T | A | T | G | G | A | A | A | A | C | T | T | A | T | C | T | T | T | A | C | A | T | G | T | A | A | T | C | C | T | A | A | G | A | T | A | G | G | A | G | T | C | A | G | C | C | T | T | A | G | A | G | A | T | C | C | T | A | A | G | A | T | T | G | A | C | A | A | A | A | T | C | T | T | A | A | C | A | T | T | A | G | C | T | C | A | T | T | T | T | T | T | C | G | A | G | A | G | A | G | A | A | A | G | C | C | T | A | A | T | G | T | A | C | C | A | A | G | G | G | A | A | C |  | : |  |  | 6 | 8 | 4 |
| M | T | 0 | 2 | 7 | 0 | 0 | 7 |  | : |  | A | C | A | A | C | C | A | C | G | A | G | A | G | A | C | G | C | A | G | T | C | T | T | C | G | G | C | G | G | C | A | T | G | A | A | A | G | G | T | A | A | T | C | T | T | T | C | C | T | A | C | G | G | G | A | A | A | C | T | T | A | T | C | T | T | T | A | C | A | T | G | T | A | A | T | C | C | T | A | A | G | A | T | A | G | G | A | G | T | C | A | G | C | C | T | T | A | G | A | G | A | T | C | C | T | A | G | G | A | T | T | G | A | C | A | A | A | A | T | C | T | T | A | A | C | A | T | T | A | G | C | T | C | A | C | T | T | T | T | T | C | G | A | G | A | G | A | G | A | A | A | G | C | C | T | A | A | T | G | C | A | C | C | T | A | G | G | G | A | A | C |  | : |  |  | 6 | 8 | 4 |
| M | T | 0 | 3 | 6 | 0 | 5 | 3 |  | : |  | A | C | A | A | C | C | G | C | G | A | G | A | G | A | C | G | C | A | G | T | C | T | T | C | G | G | C | G | G | C | A | T | G | A | A | A | G | G | T | A | A | T | C | T | T | T | C | C | T | A | C | G | G | G | A | A | A | C | T | T | A | T | C | T | T | T | A | C | A | T | G | T | A | A | T | C | C | T | A | A | G | A | T | A | G | G | A | G | T | C | A | G | C | C | T | T | A | G | A | G | A | T | C | C | T | A | G | G | A | T | T | G | A | C | A | A | A | A | T | C | T | T | A | A | C | A | T | T | A | G | C | T | C | A | C | T | T | T | T | T | C | G | A | G | A | G | A | G | A | A | A | G | C | C | T | A | A | T | G | C | A | C | C | T | A | G | G | G | A | A | C |  | : |  |  | 6 | 8 | 4 |
| M | T | 0 | 3 | 6 | 0 | 5 | 4 |  | : |  | A | C | A | A | C | C | G | C | G | A | G | A | G | A | C | G | C | A | G | T | C | T | T | C | G | G | C | G | G | C | A | T | G | A | A | A | G | G | T | A | A | T | C | T | T | T | C | C | T | A | C | G | G | G | A | A | A | C | T | T | A | T | C | T | T | T | A | C | A | T | G | T | A | A | T | C | C | T | A | A | G | A | T | A | G | G | A | G | T | C | A | G | C | C | T | T | A | G | A | G | A | T | C | C | T | A | G | G | A | T | T | G | A | C | A | A | A | A | T | C | T | T | A | A | C | A | T | T | A | G | C | T | C | A | C | T | T | T | T | T | C | G | A | G | A | G | A | G | A | A | A | G | C | C | T | A | A | T | G | C | A | C | C | T | A | G | G | G | A | A | C |  | : |  |  | 6 | 8 | 4 |
| M | T | 0 | 3 | 6 | 0 | 5 | 5 |  | : |  | A | C | A | A | C | C | G | C | G | A | G | A | G | A | C | G | C | A | G | T | C | T | T | C | G | G | C | G | G | C | A | T | G | A | A | A | G | G | T | A | A | T | C | T | T | T | C | C | T | A | C | G | G | G | A | A | A | C | T | T | A | T | C | T | T | T | A | C | A | T | G | T | A | A | T | C | C | T | A | A | G | A | T | A | G | G | A | G | T | C | A | G | C | C | T | T | A | G | A | G | A | T | C | C | T | A | G | G | A | T | T | G | A | C | A | A | A | A | T | C | T | T | A | A | C | A | T | T | A | G | C | T | C | A | C | T | T | T | T | T | C | G | A | G | A | G | A | G | A | A | A | G | C | C | T | A | A | T | G | C | A | C | C | T | A | G | G | G | A | A | T |  | : |  |  | 6 | 8 | 4 |
| M | T | 0 | 3 | 6 | 0 | 5 | 6 |  | : |  | A | C | A | A | C | C | G | C | T | A | G | A | G | A | C | G | C | A | G | T | T | T | T | C | G | G | C | G | G | C | A | T | G | A | A | A | G | G | T | A | A | C | C | T | T | T | C | C | T | A | T | G | G | A | A | A | A | C | T | T | A | T | C | T | T | T | A | C | A | T | G | T | A | A | T | C | C | T | A | A | G | A | T | A | G | G | A | G | T | C | A | G | C | C | T | T | A | G | A | G | A | T | C | C | T | A | A | G | A | T | T | G | A | C | A | A | A | A | T | C | T | T | A | A | C | A | T | T | A | G | C | C | C | A | C | T | T | T | T | T | C | G | A | G | A | G | A | G | A | A | A | G | C | C | T | A | A | T | G | T | A | C | C | A | A | G | G | G | A | A | C |  | : |  |  | 6 | 8 | 4 |
| M | T | 0 | 3 | 6 | 0 | 5 | 7 |  | : |  | A | C | A | A | C | C | G | C | T | A | G | A | G | A | C | G | C | A | G | T | T | T | T | C | G | G | C | G | G | C | A | T | G | A | A | A | G | G | T | A | A | C | C | T | T | T | C | C | T | A | T | G | G | A | A | A | A | C | T | T | A | T | C | T | T | T | A | C | A | T | G | T | A | A | T | C | C | T | A | A | G | A | T | A | G | G | A | G | T | C | A | G | C | C | T | T | A | G | A | G | A | T | C | C | T | A | A | G | A | T | T | G | A | C | A | A | A | A | T | C | T | T | A | A | C | A | T | T | A | G | C | C | C | A | C | T | T | T | T | T | C | G | A | G | A | G | A | G | A | A | A | G | C | C | T | A | A | T | G | T | A | C | C | A | A | G | G | G | A | A | C |  | : |  |  | 6 | 8 | 4 |
| K | X | 2 | 4 | 9 | 7 | 3 | 8 |  | : |  | A | C | A | A | C | C | G | C | T | A | G | A | G | A | C | G | C | A | G | T | T | T | T | C | G | G | C | G | G | C | A | T | G | A | A | A | G | G | T | A | A | C | C | T | T | T | C | C | T | A | T | G | G | A | A | A | A | C | T | T | A | T | C | T | T | T | A | C | A | T | G | T | A | A | T | C | C | T | A | A | G | A | T | A | G | G | A | G | T | C | A | G | C | C | T | T | A | G | A | G | A | T | C | C | T | A | A | G | A | T | T | G | A | C | A | A | A | A | T | C | T | T | A | A | C | A | T | T | A | G | C | T | C | A | C | T | T | T | T | T | C | G | A | G | A | A | A | G | A | A | A | G | C | C | T | A | A | T | G | T | A | C | C | A | A | G | G | G | A | A | C |  | : |  |  | 6 | 8 | 4 |
| K | X | 2 | 4 | 9 | 7 | 3 | 7 |  | : |  | A | C | A | A | C | C | G | C | T | A | G | A | G | A | C | G | C | A | G | T | T | T | T | C | G | G | C | G | G | C | A | T | G | A | A | A | G | G | T | A | A | C | C | T | T | T | C | C | T | A | T | G | G | A | A | A | A | C | T | T | A | T | C | T | T | T | A | C | A | T | G | T | A | A | T | C | C | T | A | A | G | A | T | A | G | G | A | G | T | C | A | G | C | C | T | T | A | G | A | G | A | T | C | C | T | A | A | G | A | T | T | G | A | C | A | A | A | A | T | C | T | T | A | A | C | A | T | T | A | G | C | T | C | A | C | T | T | T | T | T | C | G | A | G | A | A | A | G | A | A | A | G | C | C | T | A | A | T | G | T | A | C | C | A | A | G | G | G | A | A | C |  | : |  |  | 6 | 8 | 4 |
| K | X | 2 | 4 | 9 | 7 | 3 | 6 |  | : |  | A | C | A | A | C | C | G | C | T | A | G | A | G | A | C | G | C | A | G | T | T | T | T | C | G | G | C | G | G | C | A | T | G | A | A | A | G | G | T | A | A | C | C | T | T | T | C | C | T | A | T | G | G | A | A | A | A | C | T | T | A | T | C | T | T | T | A | C | A | T | G | T | A | A | T | C | C | T | A | A | G | A | T | A | G | G | A | G | T | C | A | G | C | C | T | T | A | G | A | G | A | T | C | C | T | A | A | G | A | T | T | G | A | C | A | A | A | A | T | C | T | T | A | A | C | A | T | T | A | G | C | T | C | A | C | T | T | T | T | T | C | G | A | G | A | A | A | G | A | A | A | G | C | C | T | A | A | T | G | T | A | C | C | A | A | G | G | G | A | A | C |  | : |  |  | 6 | 8 | 4 |
| K | X | 2 | 4 | 9 | 7 | 3 | 5 |  | : |  | A | C | A | A | C | C | G | C | T | A | G | A | G | A | C | G | C | A | G | T | T | T | T | C | G | G | C | G | G | C | A | T | G | A | A | A | G | G | T | A | A | C | C | T | T | T | C | C | T | A | T | G | G | A | A | A | A | C | T | T | A | T | C | T | T | T | A | C | A | T | G | T | A | A | T | C | C | T | A | A | G | A | T | A | G | G | A | G | T | C | A | G | C | C | T | T | A | G | A | G | A | T | C | C | T | A | A | G | A | T | T | G | A | C | A | A | A | A | T | C | T | T | A | A | C | A | T | T | A | G | C | T | C | A | C | T | T | T | T | T | C | G | A | G | A | G | A | G | A | A | A | G | C | C | T | A | A | T | G | T | A | C | C | A | A | G | G | G | A | A | C |  | : |  |  | 6 | 8 | 4 |
| M | F | 1 | 9 | 7 | 9 | 1 | 6 |  | : |  | A | C | A | A | C | C | G | C | T | A | G | A | G | A | C | G | C | A | G | T | T | T | T | C | G | G | C | G | G | C | A | T | G | A | A | A | G | G | T | A | A | C | C | T | T | T | C | C | T | A | T | G | G | A | A | A | A | C | T | T | A | T | C | T | T | T | A | C | A | T | G | T | A | A | T | C | C | T | A | A | G | A | T | A | G | G | A | G | T | C | A | G | C | C | T | T | A | G | A | G | A | T | C | C | T | A | A | G | A | T | T | G | A | C | A | A | A | A | T | C | T | T | A | A | C | A | T | T | A | G | C | T | C | A | C | T | T | T | T | T | C | G | A | G | A | G | A | G | A | A | A | G | C | C | T | A | A | T | G | T | A | C | C | A | A | G | G | G | A | A | C |  | : |  |  | 6 | 8 | 4 |
| K | T | 2 | 5 | 0 | 6 | 3 | 2 |  | : |  | A | C | A | A | C | C | G | C | G | A | G | A | G | A | C | G | C | A | G | T | C | T | T | C | G | G | T | G | G | C | A | T | G | A | A | A | G | G | T | A | A | T | C | T | T | T | C | C | T | A | C | G | G | G | A | A | A | C | T | T | A | T | C | T | T | T | A | C | A | T | G | T | A | A | T | C | C | T | A | A | G | A | T | A | G | G | A | G | T | C | A | G | C | C | T | T | A | G | A | G | A | T | C | C | T | A | G | G | A | T | T | G | A | C | A | A | A | A | T | C | T | T | A | A | C | A | T | T | A | G | C | T | C | A | C | T | T | T | T | T | C | G | A | G | A | G | A | G | A | A | A | G | C | C | T | A | A | T | G | C | A | C | C | T | A | G | G | G | A | A | C |  | : |  |  | 6 | 8 | 4 |
|  |  |  |  |  |  |  |  |  |  |  | A | C | A | A | C | C | g | C |  | A | G | A | G | A | C | G | C | A | G | T |  | T | T | C | G | G | c | G | G | C | A | T | G | A | A | A | G | G | T | A | A |  | C | T | T | T | C | C | T | A |  | G | G |  | A | A | A | C | T | T | A | T | C | T | T | T | A | C | A | T | G | T | A | A | T | C | C | T | A | A | G | A | T | A | G | G | A | G | T | C | A | G | C | C | T | T | A | G | A | G | A | T | C | C | T | A |  | G | A | T | T | G | A | C | A | A | A | A | T | C | T | T | A | A | C | A | T | T | A | G | C | t | C | A | c | T | T | T | T | T | C | G | A | g | A | g | A | G | A | A | A | G | C | C | T | A | A | T | G |  | A | C | C |  | A | G | G | G | A | A | c |  |  |  |  |  |  |  |

|  |  |  |  |  |  |  |  |  |  |  |  |  |  |  |  |  |  |  |  |  |  |  |  |  |  |  |  |  |  |  |  |  |  |  |  |  |  |  |  |  |  |  |  |  |  |  |  |  |  |  |  |  |  |  |  |  |  |  |  |  |  |  |  |  |  |  |  |  |  |  |  |  |  |  |  |  |  |  |  |  |  |  |  |  |  |  |  |  |  |  |  |  |  |  |  |  |  |  |  |  |  |  |  |  |  |  |  |  |  |  |  |  |  |  |  |  |  |  |  |  |  |  |  |  |  |  |  |  |  |  |  |  |  |  |  |  |  |  |  |  |  |  |  |  |  |  |  |  |  |  |  |  |  |  |  |  |  |  |  |  |  |  |  |  |  |  |  |  |  |  |  |  |  |  |  |  |  |  |  |  |  |  |  |  |  |  |  |  |
| --- | --- | --- | --- | --- | --- | --- | --- | --- | --- | --- | --- | --- | --- | --- | --- | --- | --- | --- | --- | --- | --- | --- | --- | --- | --- | --- | --- | --- | --- | --- | --- | --- | --- | --- | --- | --- | --- | --- | --- | --- | --- | --- | --- | --- | --- | --- | --- | --- | --- | --- | --- | --- | --- | --- | --- | --- | --- | --- | --- | --- | --- | --- | --- | --- | --- | --- | --- | --- | --- | --- | --- | --- | --- | --- | --- | --- | --- | --- | --- | --- | --- | --- | --- | --- | --- | --- | --- | --- | --- | --- | --- | --- | --- | --- | --- | --- | --- | --- | --- | --- | --- | --- | --- | --- | --- | --- | --- | --- | --- | --- | --- | --- | --- | --- | --- | --- | --- | --- | --- | --- | --- | --- | --- | --- | --- | --- | --- | --- | --- | --- | --- | --- | --- | --- | --- | --- | --- | --- | --- | --- | --- | --- | --- | --- | --- | --- | --- | --- | --- | --- | --- | --- | --- | --- | --- | --- | --- | --- | --- | --- | --- | --- | --- | --- | --- | --- | --- | --- | --- | --- | --- | --- | --- | --- | --- | --- | --- | --- | --- | --- | --- | --- | --- | --- | --- | --- | --- | --- |
|  |  |  |  |  |  |  |  |  |  |  |  |  |  |  |  |  |  |  |  |  |  |  |  |  |  |  |  |  |  |  |  |  |  |  |  |  |  |  |  |  |  |  |  |  |  |  |  |  |  |  |  |  |  |  |  |  |  |  |  |  |  |  |  |  |  |  |  |  |  |  |  |  |  |  |  |  |  |  |  |  |  |  |  |  |  |  |  |  |  |  |  |  |  |  |  |  |  |  |  |  |  |  |  |  |  |  |  |  |  |  |  |  |  |  |  |  |  |  |  |  |  |  |  |  |  |  |  |  |  |  |  |  |  |  |  |  |  |  |  |  |  |  |  |  |  |  |  |  |  |  |  |  |  |  |  |  |  |  |  |  |  |  |  |  |  |  |  |  |  |  |  |  |  |  |  |  |  |  |  |  |  |  |  |  |  |  |  |  |
|  |  |  |  |  |  |  |  |  |  |  |  |  |  |  |  | \* |  |  |  |  |  |  |  | 7 | 0 | 0 |  |  |  |  |  |  |  |  |  | \* |  |  |  |  |  |  |  | 7 | 2 | 0 |  |  |  |  |  |  |  |  |  | \* |  |  |  |  |  |  |  | 7 | 4 | 0 |  |  |  |  |  |  |  |  |  | \* |  |  |  |  |  |  |  | 7 | 6 | 0 |  |  |  |  |  |  |  |  |  | \* |  |  |  |  |  |  |  | 7 | 8 | 0 |  |  |  |  |  |  |  |  |  | \* |  |  |  |  |  |  |  | 8 | 0 | 0 |  |  |  |  |  |  |  |  |  | \* |  |  |  |  |  |  |  | 8 | 2 | 0 |  |  |  |  |  |  |  |  |  | \* |  |  |  |  |  |  |  | 8 | 4 | 0 |  |  |  |  |  |  |  |  |  | \* |  |  |  |  |  |  |  |  |  |  |  |  |
| M | N | 9 | 5 | 6 | 5 | 2 | 0 |  | : |  | C | A | C | C | C | C | T | A | T | A | C | C | A | T | A | T | C | C | T | A | C | A | A | A | C | T | A | G | G | T | T | A | T | G | T | A | T | T | G | T | C | A | A | A | C | T | C | T | C | A | C | C | A | T | A | G | T | C | T | A | G | A | G | T | T | T | A | G | A | C | C | A | A | A | A | G | A | A | C | C | C | A | T | C | T | G | C | A | T | A | G | A | C | G | A | C | C | T | G | T | T | T | A | G | T | G | C | A | G | T | A | G | G | T | A | G | A | A | T | A | T | C | T | C | A | G | G | C | C | C | C | A | G | T | C | C | A | A | G | A | G | A | T | A | A | G | T | C | C | T | T | T | A | G | A | A | A | A | C | T | A | T | T | G | G | A | C | C | A | T | G |  | : |  |  | 8 | 5 | 5 |
| M | T | 0 | 1 | 2 | 7 | 3 | 2 |  | : |  | C | A | C | C | C | C | T | A | T | A | C | C | A | T | A | T | C | C | T | A | C | A | A | A | C | T | A | G | G | T | T | A | T | G | T | A | T | T | G | T | C | A | A | A | C | T | C | T | C | A | T | C | A | T | A | G | T | C | T | A | G | A | G | T | T | T | A | G | A | C | C | A | A | A | A | G | A | A | C | C | C | A | T | C | T | G | C | A | T | A | G | A | C | G | A | C | C | T | G | T | T | T | A | G | T | G | C | A | G | T | A | G | G | T | A | G | A | A | T | A | T | C | T | C | A | G | G | C | C | C | C | A | G | T | C | C | A | A | G | A | G | A | T | A | A | G | T | C | C | T | T | T | A | G | A | A | A | A | C | T | A | T | T | G | G | A | C | C | A | T | G |  | : |  |  | 8 | 5 | 5 |
| M | T | 0 | 1 | 2 | 7 | 3 | 4 |  | : |  | C | A | C | C | C | T | T | A | C | A | C | C | A | T | A | T | C | C | T | A | C | A | A | A | T | T | A | G | G | T | T | A | T | G | T | A | C | T | G | T | C | A | A | A | C | T | C | T | C | A | C | C | A | T | A | G | T | T | T | A | G | A | G | T | T | T | A | G | A | C | C | A | A | A | A | G | A | A | C | C | C | A | T | C | T | G | C | A | T | A | G | A | C | G | A | C | C | T | G | T | T | T | A | G | T | G | C | A | G | T | A | G | G | T | A | G | A | A | T | A | T | C | T | C | A | G | G | C | C | C | C | A | G | T | C | C | A | A | G | A | G | A | T | A | A | G | T | C | C | T | T | T | A | G | A | A | A | A | C | T | A | T | T | G | G | A | C | C | A | T | G |  | : |  |  | 8 | 5 | 5 |
| M | T | 0 | 2 | 7 | 0 | 0 | 6 |  | : |  | C | A | C | C | C | C | T | A | T | A | C | C | A | T | A | T | C | C | T | A | C | A | A | A | C | T | A | G | G | T | T | A | T | G | T | A | T | T | G | T | C | A | A | A | C | T | C | T | C | A | C | C | A | T | A | G | T | C | T | A | G | A | G | T | T | T | A | G | A | C | C | A | A | A | A | G | A | A | C | C | C | A | T | C | T | G | C | A | T | A | G | A | C | G | A | C | C | T | G | T | T | T | A | G | T | G | C | A | G | T | A | G | G | T | A | G | A | A | T | A | T | C | T | C | A | G | G | C | C | C | C | A | G | T | C | C | A | A | G | A | G | A | T | A | A | G | T | C | C | T | T | T | A | G | A | A | A | A | C | T | A | T | T | G | G | A | C | C | A | T | G |  | : |  |  | 8 | 5 | 5 |
| M | T | 0 | 2 | 7 | 0 | 0 | 7 |  | : |  | C | A | C | C | C | C | T | A | T | A | C | C | A | T | A | T | C | C | T | A | C | A | A | A | C | T | A | G | G | T | T | A | T | G | T | A | C | T | G | T | C | A | A | A | C | T | C | T | C | A | C | C | A | T | A | G | T | T | T | A | G | A | G | T | T | T | A | G | A | C | C | A | A | A | A | G | A | A | C | C | C | A | T | C | T | G | C | A | T | A | G | A | C | G | A | C | C | T | G | T | T | T | A | G | T | G | C | A | G | T | A | G | G | T | A | G | A | A | T | A | T | C | T | C | A | G | G | C | C | C | C | A | G | T | C | C | A | A | G | A | G | A | T | A | A | G | T | C | C | T | T | T | A | G | A | A | A | A | C | T | A | T | T | G | G | A | C | C | A | T | G |  | : |  |  | 8 | 5 | 5 |
| M | T | 0 | 3 | 6 | 0 | 5 | 3 |  | : |  | C | A | C | C | C | C | T | A | T | A | C | C | A | T | A | T | C | C | T | A | C | A | A | A | C | T | A | G | G | T | T | A | T | G | T | A | C | T | G | T | C | A | A | A | C | T | C | T | C | A | C | C | A | T | A | G | T | T | T | A | G | A | G | T | T | T | A | G | A | C | C | A | A | A | A | G | A | A | C | C | C | A | T | C | T | G | C | A | T | A | G | A | C | G | A | C | C | T | G | T | T | T | A | G | T | G | C | A | G | T | A | G | G | T | A | G | A | A | T | A | T | C | T | C | A | G | G | C | C | C | C | A | G | T | C | C | A | A | G | A | G | A | T | A | A | G | T | C | C | T | T | T | A | G | A | A | A | A | C | T | A | T | T | G | G | A | C | C | A | T | G |  | : |  |  | 8 | 5 | 5 |
| M | T | 0 | 3 | 6 | 0 | 5 | 4 |  | : |  | C | A | C | C | C | C | T | A | T | A | C | C | A | T | A | T | C | C | T | A | C | A | A | A | C | T | A | G | G | T | T | A | T | G | T | A | C | T | G | T | C | A | A | A | C | T | C | T | C | A | C | C | A | T | A | G | T | T | T | A | G | A | G | T | T | T | A | G | A | C | C | A | A | A | A | G | A | A | C | C | C | A | T | C | T | G | C | A | T | A | G | A | C | G | A | C | C | T | G | T | T | T | A | G | T | G | C | A | G | T | A | G | G | T | A | G | A | A | T | A | T | C | T | C | A | G | G | C | C | C | C | A | G | T | C | C | A | A | G | A | G | A | T | A | A | G | T | C | C | T | T | T | A | G | A | A | A | A | C | T | A | T | T | G | G | A | C | C | A | T | G |  | : |  |  | 8 | 5 | 5 |
| M | T | 0 | 3 | 6 | 0 | 5 | 5 |  | : |  | C | A | C | C | C | T | T | A | C | A | C | C | A | T | A | T | C | C | T | A | C | A | A | A | T | T | A | G | G | T | T | A | T | G | T | A | C | T | G | T | C | A | A | A | C | T | C | T | C | A | C | C | A | T | A | G | T | T | T | A | G | A | G | T | T | T | A | G | A | C | C | A | A | A | A | G | A | A | C | C | C | A | T | C | T | G | C | A | T | A | G | A | C | G | A | C | C | T | G | T | T | T | A | G | T | G | C | A | G | T | A | G | G | T | A | G | A | A | T | A | T | C | T | C | A | G | G | C | C | C | C | A | G | T | C | C | A | A | G | A | G | A | T | A | A | G | T | C | C | C | T | T | A | G | A | A | A | A | C | T | A | T | T | G | G | A | C | C | A | T | G |  | : |  |  | 8 | 5 | 5 |
| M | T | 0 | 3 | 6 | 0 | 5 | 6 |  | : |  | C | A | C | C | C | C | T | A | T | A | C | C | A | T | A | T | C | C | T | A | C | A | A | A | C | T | A | G | G | T | T | A | T | G | T | A | T | T | G | T | C | A | A | A | C | T | C | T | C | A | T | C | A | T | A | G | T | C | T | A | G | A | G | T | T | T | A | G | A | C | C | C | A | A | A | G | A | A | C | C | C | A | T | C | T | G | C | A | T | A | G | A | C | G | A | C | C | T | G | T | T | T | A | G | T | G | C | A | G | T | A | G | G | T | A | G | A | A | T | A | T | C | T | C | A | G | G | C | C | C | C | A | G | T | C | C | A | A | G | A | G | A | T | A | A | G | T | C | C | T | C | T | A | G | A | A | A | G | C | T | A | T | T | G | G | A | C | C | A | T | G |  | : |  |  | 8 | 5 | 5 |
| M | T | 0 | 3 | 6 | 0 | 5 | 7 |  | : |  | C | A | C | C | C | C | T | A | T | A | C | C | A | T | A | T | C | C | T | A | C | A | A | A | C | T | A | G | G | T | T | A | T | G | T | A | T | T | G | T | C | A | A | A | C | T | C | T | C | A | T | C | A | T | A | G | T | C | T | A | G | A | G | T | T | T | A | G | A | C | C | A | A | A | A | G | A | A | C | C | C | A | T | C | T | G | C | A | T | A | G | A | C | G | A | C | C | T | G | T | T | T | A | G | T | G | C | A | G | T | A | G | G | T | A | G | A | A | T | A | T | C | T | C | A | G | G | C | C | C | C | A | G | T | C | C | A | A | G | A | G | A | T | A | A | G | T | C | C | T | T | T | A | G | A | A | A | G | C | T | A | T | T | G | G | A | C | C | A | T | G |  | : |  |  | 8 | 5 | 5 |
| K | X | 2 | 4 | 9 | 7 | 3 | 8 |  | : |  | C | A | C | C | C | C | T | A | T | A | C | C | A | T | A | T | C | C | T | A | C | A | A | A | C | T | A | G | G | T | T | A | T | G | T | A | T | T | G | T | C | A | A | A | C | T | C | T | C | A | C | C | A | T | A | G | T | C | T | A | G | A | G | T | T | T | A | G | A | C | C | A | A | A | A | G | A | A | C | C | C | A | T | C | T | G | C | A | T | A | G | A | C | G | A | C | C | T | G | T | T | T | A | G | T | G | C | A | G | T | A | G | G | T | A | G | A | A | T | A | T | C | T | C | A | G | G | C | C | C | C | A | G | T | C | C | A | A | G | A | G | A | T | A | A | G | T | C | C | T | T | T | A | G | A | A | A | A | C | T | A | T | T | G | G | A | C | C | A | T | G |  | : |  |  | 8 | 5 | 5 |
| K | X | 2 | 4 | 9 | 7 | 3 | 7 |  | : |  | C | A | C | C | C | C | T | A | T | A | C | C | A | T | A | T | C | C | T | A | C | A | A | A | C | T | A | G | G | T | T | A | T | G | T | A | T | T | G | T | C | A | A | A | C | T | C | T | C | A | C | C | A | T | A | G | T | C | T | A | G | A | G | T | T | T | A | G | A | C | C | A | A | A | A | G | A | A | C | C | C | A | T | C | T | G | C | A | T | A | G | A | C | G | A | C | C | T | G | T | T | T | A | G | T | G | C | A | G | T | A | G | G | T | A | G | A | A | T | A | T | C | T | C | A | G | G | C | C | C | C | A | G | T | C | C | A | A | G | A | G | A | T | A | A | G | T | C | C | T | T | T | A | G | A | A | A | A | C | T | A | T | T | G | G | A | C | C | A | T | G |  | : |  |  | 8 | 5 | 5 |
| K | X | 2 | 4 | 9 | 7 | 3 | 6 |  | : |  | C | A | C | C | C | C | T | A | T | A | C | C | A | T | A | T | C | C | T | A | C | A | A | A | C | T | A | G | G | T | T | A | T | G | T | A | T | T | G | T | C | A | A | A | C | T | C | T | C | A | C | C | A | T | A | G | T | C | T | A | G | A | G | T | T | T | A | G | A | C | C | A | A | A | A | G | A | A | C | C | C | A | T | C | T | G | C | A | T | A | G | A | C | G | A | C | C | T | G | T | T | T | A | G | T | G | C | A | G | T | A | G | G | T | A | G | A | A | T | A | T | C | T | C | A | G | G | C | C | C | C | A | G | T | C | C | A | A | G | A | G | A | T | A | A | G | T | C | C | T | T | T | A | G | A | A | A | A | C | T | A | T | T | G | G | A | C | C | A | T | G |  | : |  |  | 8 | 5 | 5 |
| K | X | 2 | 4 | 9 | 7 | 3 | 5 |  | : |  | C | A | C | C | C | C | T | A | T | A | C | C | A | T | A | T | C | C | T | A | C | A | A | A | C | T | A | G | G | T | T | A | T | G | T | A | T | T | G | T | C | A | A | A | C | T | C | T | C | A | C | C | A | T | A | G | T | C | T | A | G | A | G | T | T | T | A | G | A | C | C | A | A | A | A | G | A | A | C | C | C | A | T | C | T | G | C | A | T | A | G | A | C | G | A | C | C | T | G | T | T | T | A | G | T | G | C | A | G | T | A | G | G | A | A | G | A | A | T | A | T | C | T | C | A | G | G | C | C | C | C | A | G | T | C | C | A | A | G | A | G | A | T | A | A | G | T | C | C | T | T | T | A | G | A | A | A | A | C | T | A | T | T | G | G | A | C | C | A | T | G |  | : |  |  | 8 | 5 | 5 |
| M | F | 1 | 9 | 7 | 9 | 1 | 6 |  | : |  | C | A | C | C | C | C | T | A | T | A | C | C | A | T | A | T | C | C | T | A | C | A | A | A | C | T | A | G | G | T | T | A | T | G | T | A | T | T | G | T | C | A | A | A | C | T | C | T | C | A | C | C | A | T | A | G | T | C | T | A | G | A | G | T | T | T | A | G | A | C | C | A | A | A | A | G | A | A | C | C | C | A | T | C | T | G | C | A | T | A | G | A | C | G | A | C | C | T | G | T | T | T | A | G | T | G | C | A | G | T | A | G | G | T | A | G | A | A | T | A | T | C | T | C | A | G | G | C | C | C | C | A | G | T | C | C | A | A | G | A | G | A | T | A | A | G | T | C | C | T | T | T | A | G | A | A | A | A | C | T | A | T | T | G | G | A | C | C | A | T | G |  | : |  |  | 8 | 5 | 5 |
| K | T | 2 | 5 | 0 | 6 | 3 | 2 |  | : |  | C | A | C | C | C | C | T | A | T | A | C | C | A | T | A | T | C | C | T | A | C | A | A | A | C | T | A | G | G | T | T | A | T | G | T | A | C | T | G | T | C | A | A | A | C | T | C | T | C | A | C | C | A | T | A | G | T | T | T | A | G | A | G | T | T | T | A | G | A | C | C | A | A | A | A | G | A | A | C | C | C | A | T | C | T | G | C | A | T | A | G | A | C | G | A | C | C | T | G | T | T | T | A | G | T | G | C | A | G | T | A | G | G | T | A | G | A | A | T | A | T | C | T | C | A | G | G | C | C | C | C | A | G | T | C | C | A | A | G | A | G | A | T | A | A | G | T | C | C | T | T | T | A | G | A | A | A | A | C | T | A | T | T | G | G | A | C | C | A | T | G |  | : |  |  | 8 | 5 | 5 |
|  |  |  |  |  |  |  |  |  |  |  | C | A | C | C | C | c | T | A | t | A | C | C | A | T | A | T | C | C | T | A | C | A | A | A | c | T | A | G | G | T | T | A | T | G | T | A |  | T | G | T | C | A | A | A | C | T | C | T | C | A | c | C | A | T | A | G | T |  | T | A | G | A | G | T | T | T | A | G | A | C | C | a | A | A | A | G | A | A | C | C | C | A | T | C | T | G | C | A | T | A | G | A | C | G | A | C | C | T | G | T | T | T | A | G | T | G | C | A | G | T | A | G | G | t | A | G | A | A | T | A | T | C | T | C | A | G | G | C | C | C | C | A | G | T | C | C | A | A | G | A | G | A | T | A | A | G | T | C | C | t | t | T | A | G | A | A | A | a | C | T | A | T | T | G | G | A | C | C | A | T | G |  |  |  |  |  |  |  |

|  |  |  |  |  |  |  |  |  |  |  |  |  |  |  |  |  |  |  |  |  |  |  |  |  |  |  |  |  |  |  |  |  |  |  |  |  |  |  |  |  |  |  |  |  |  |  |  |  |  |  |  |  |  |  |  |  |  |  |  |  |  |  |  |  |  |  |  |  |  |  |  |  |  |  |  |  |  |  |  |  |  |  |  |  |  |  |  |  |  |  |  |  |  |  |  |  |  |  |  |  |  |  |  |  |  |  |  |  |  |  |  |  |  |  |  |  |  |  |  |  |  |  |  |  |  |  |  |  |  |  |  |  |  |  |  |  |  |  |  |  |  |  |  |  |  |  |  |  |  |  |  |  |  |  |  |  |  |  |  |  |  |  |  |  |  |  |  |  |  |  |  |  |  |  |  |  |  |  |  |  |  |  |  |  |  |  |  |  |
| --- | --- | --- | --- | --- | --- | --- | --- | --- | --- | --- | --- | --- | --- | --- | --- | --- | --- | --- | --- | --- | --- | --- | --- | --- | --- | --- | --- | --- | --- | --- | --- | --- | --- | --- | --- | --- | --- | --- | --- | --- | --- | --- | --- | --- | --- | --- | --- | --- | --- | --- | --- | --- | --- | --- | --- | --- | --- | --- | --- | --- | --- | --- | --- | --- | --- | --- | --- | --- | --- | --- | --- | --- | --- | --- | --- | --- | --- | --- | --- | --- | --- | --- | --- | --- | --- | --- | --- | --- | --- | --- | --- | --- | --- | --- | --- | --- | --- | --- | --- | --- | --- | --- | --- | --- | --- | --- | --- | --- | --- | --- | --- | --- | --- | --- | --- | --- | --- | --- | --- | --- | --- | --- | --- | --- | --- | --- | --- | --- | --- | --- | --- | --- | --- | --- | --- | --- | --- | --- | --- | --- | --- | --- | --- | --- | --- | --- | --- | --- | --- | --- | --- | --- | --- | --- | --- | --- | --- | --- | --- | --- | --- | --- | --- | --- | --- | --- | --- | --- | --- | --- | --- | --- | --- | --- | --- | --- | --- | --- | --- | --- | --- | --- | --- | --- | --- | --- | --- | --- |
|  |  |  |  |  |  |  |  |  |  |  |  |  |  |  |  |  |  |  |  |  |  |  |  |  |  |  |  |  |  |  |  |  |  |  |  |  |  |  |  |  |  |  |  |  |  |  |  |  |  |  |  |  |  |  |  |  |  |  |  |  |  |  |  |  |  |  |  |  |  |  |  |  |  |  |  |  |  |  |  |  |  |  |  |  |  |  |  |  |  |  |  |  |  |  |  |  |  |  |  |  |  |  |  |  |  |  |  |  |  |  |  |  |  |  |  |  |  |  |  |  |  |  |  |  |  |  |  |  |  |  |  |  |  |  |  |  |  |  |  |  |  |  |  |  |  |  |  |  |  |  |  |  |  |  |  |  |  |  |  |  |  |  |  |  |  |  |  |  |  |  |  |  |  |  |  |  |  |  |  |  |  |  |  |  |  |  |  |  |
|  |  |  |  |  |  |  |  |  |  |  |  |  | 8 | 6 | 0 |  |  |  |  |  |  |  |  |  | \* |  |  |  |  |  |  |  | 8 | 8 | 0 |  |  |  |  |  |  |  |  |  | \* |  |  |  |  |  |  |  | 9 | 0 | 0 |  |  |  |  |  |  |  |  |  | \* |  |  |  |  |  |  |  | 9 | 2 | 0 |  |  |  |  |  |  |  |  |  | \* |  |  |  |  |  |  |  | 9 | 4 | 0 |  |  |  |  |  |  |  |  |  | \* |  |  |  |  |  |  |  | 9 | 6 | 0 |  |  |  |  |  |  |  |  |  | \* |  |  |  |  |  |  |  | 9 | 8 | 0 |  |  |  |  |  |  |  |  |  | \* |  |  |  |  |  |  | 1 | 0 | 0 | 0 |  |  |  |  |  |  |  |  |  | \* |  |  |  |  |  |  | 1 | 0 | 2 | 0 |  |  |  |  |  |  |  |  |  |  |  |  |  |
| M | N | 9 | 5 | 6 | 5 | 2 | 0 |  | : |  | C | A | T | C | T | G | G | G | T | A | A | C | A | C | C | A | G | T | A | G | A | A | G | A | A | T | G | C | T | A | G | G | A | G | A | A | A | G | A | C | C | T | A | G | G | A | T | G | A | T | T | G | T | C | A | T | T | G | A | G | G | A | C | G | A | T | G | A | A | G | A | A | C | A | G | A | C | C | C | C | T | C | A | T | G | A | C | A | G | A | C | A | A | T | T | A | C | A | A | G | A | A | A | G | A | G | C | A | T | T | A | G | C | T | A | G | A | T | C | C | C | A | A | T | C | T | A | G | G | A | T | G | C | T | A | G | G | T | T | T | A | A | G | A | C | C | C | A | C | A | G | A | G | G | A | T | C | T | T | A | G | A | A | C | C | A | C | C | A | G | T |  | : |  | 1 | 0 | 2 | 6 |
| M | T | 0 | 1 | 2 | 7 | 3 | 2 |  | : |  | C | A | T | C | T | G | G | G | T | A | A | C | A | C | C | A | G | T | A | G | A | A | G | A | A | T | G | C | T | A | G | G | A | G | A | A | A | G | A | C | C | T | A | G | G | A | T | G | A | T | T | G | T | C | A | T | T | G | A | G | G | A | C | G | A | T | G | A | A | G | A | A | C | A | G | A | C | C | C | C | T | C | A | T | G | A | C | A | G | A | C | A | A | T | T | A | C | A | A | G | A | A | A | G | A | G | C | A | T | T | A | G | C | T | A | G | A | T | C | C | C | A | A | T | C | T | A | G | G | A | T | G | C | T | A | G | G | T | T | T | A | A | G | A | C | C | C | A | C | A | G | A | G | G | A | T | C | T | T | A | G | A | A | C | C | A | C | C | A | G | T |  | : |  | 1 | 0 | 2 | 6 |
| M | T | 0 | 1 | 2 | 7 | 3 | 4 |  | : |  | C | A | T | C | T | G | G | G | T | A | A | T | A | C | C | A | G | T | A | G | A | A | G | A | A | T | G | C | T | A | G | G | A | G | A | A | A | G | A | C | C | T | A | G | G | A | T | G | A | T | T | G | T | C | A | T | T | G | A | G | G | A | C | G | A | T | G | A | A | G | A | A | C | A | G | A | C | C | C | C | T | C | A | T | G | A | C | A | G | A | C | T | A | T | T | A | C | A | A | G | A | C | A | G | A | G | C | A | T | T | A | G | C | T | A | G | A | T | C | C | C | A | A | T | C | T | A | G | G | A | T | G | C | T | A | G | G | T | T | T | A | A | G | A | C | C | C | A | C | A | G | A | G | G | A | T | C | T | T | A | G | A | A | C | C | A | C | C | A | G | T |  | : |  | 1 | 0 | 2 | 6 |
| M | T | 0 | 2 | 7 | 0 | 0 | 6 |  | : |  | C | A | T | C | T | G | G | G | T | A | A | C | A | C | C | A | G | T | A | G | A | A | G | A | A | T | G | C | T | A | G | G | A | G | A | A | A | G | A | C | C | T | A | G | G | A | T | G | A | T | T | G | T | C | A | T | T | G | A | G | G | A | C | G | A | T | G | A | A | G | A | A | C | A | G | A | T | C | C | C | T | C | A | T | G | A | C | A | G | A | C | A | A | T | T | A | C | A | A | G | A | A | A | G | A | G | C | A | T | T | A | G | C | T | A | G | A | T | C | C | C | A | A | T | C | T | A | G | G | A | T | G | C | T | A | G | G | T | T | T | A | A | G | A | C | C | C | A | C | A | G | A | G | G | A | T | C | T | T | A | G | A | A | C | C | A | C | C | A | G | T |  | : |  | 1 | 0 | 2 | 6 |
| M | T | 0 | 2 | 7 | 0 | 0 | 7 |  | : |  | C | A | T | C | T | G | G | G | T | A | A | C | A | C | C | A | G | T | A | G | A | A | G | A | A | T | G | C | T | A | G | G | A | G | A | A | A | G | A | C | C | T | A | G | G | A | T | G | A | T | T | G | T | C | A | T | T | G | A | G | G | A | C | G | A | T | G | A | A | G | A | A | C | A | G | A | C | C | C | C | T | C | A | T | G | A | C | A | G | A | C | A | A | T | T | A | C | A | A | G | A | A | A | G | A | G | C | A | T | T | A | G | C | T | A | G | A | T | C | C | C | A | A | T | C | T | A | G | G | A | T | G | C | T | A | G | G | T | T | T | A | A | G | A | C | C | C | A | C | A | G | A | G | G | A | T | C | T | T | A | G | A | A | C | C | A | C | T | A | G | T |  | : |  | 1 | 0 | 2 | 6 |
| M | T | 0 | 3 | 6 | 0 | 5 | 3 |  | : |  | C | A | T | C | T | G | G | G | T | A | A | C | A | C | C | A | G | T | A | G | A | A | G | A | A | T | G | C | T | A | G | G | A | G | A | A | A | G | A | C | C | T | A | G | G | A | T | G | A | T | T | G | T | C | A | T | T | G | A | G | G | A | C | G | A | T | G | A | A | G | A | A | C | A | G | A | C | C | C | C | T | C | A | T | G | A | C | A | G | A | C | A | A | T | T | A | C | A | A | G | A | A | A | G | A | G | C | A | T | T | A | G | C | T | A | G | A | T | C | C | C | A | A | T | C | T | A | G | G | A | T | G | C | T | A | G | G | T | T | T | A | A | G | A | C | C | C | A | C | A | G | A | G | G | A | T | C | T | T | A | G | A | A | C | C | A | C | T | A | G | T |  | : |  | 1 | 0 | 2 | 6 |
| M | T | 0 | 3 | 6 | 0 | 5 | 4 |  | : |  | C | A | T | C | T | G | G | G | T | A | A | C | A | C | C | A | G | T | A | G | A | A | G | A | A | T | G | C | T | A | G | G | A | G | A | A | A | G | A | C | C | T | A | G | G | A | T | G | A | T | T | G | T | C | A | T | T | G | A | G | G | A | C | G | A | T | G | A | A | G | A | A | C | A | G | A | C | C | C | C | T | C | A | T | G | A | C | A | G | A | C | A | A | T | T | A | C | A | A | G | A | A | A | G | A | G | C | A | T | T | A | G | C | T | A | G | A | T | C | C | C | A | A | T | C | T | A | G | G | A | T | G | C | T | A | G | G | T | T | T | A | A | G | A | C | C | C | A | C | A | G | A | G | G | A | T | C | T | T | A | G | A | A | C | C | A | C | T | A | G | T |  | : |  | 1 | 0 | 2 | 6 |
| M | T | 0 | 3 | 6 | 0 | 5 | 5 |  | : |  | C | A | T | C | T | G | G | G | T | A | A | C | A | C | C | A | A | T | A | G | A | A | G | A | A | T | G | C | T | A | G | G | A | G | A | A | A | G | A | C | C | T | A | G | G | A | T | G | A | T | T | G | T | C | A | T | T | G | A | G | G | A | C | G | A | T | G | A | A | G | A | A | C | A | G | A | C | C | C | C | T | C | A | T | G | A | C | A | G | A | C | A | A | T | T | A | C | A | A | G | A | A | A | G | A | G | C | A | T | T | A | G | C | T | A | G | A | T | C | C | C | A | A | T | C | T | A | G | G | A | T | G | C | T | A | G | G | T | T | T | A | A | G | A | C | C | C | A | C | A | G | A | G | G | A | T | C | T | T | A | G | A | A | C | C | A | C | C | A | G | T |  | : |  | 1 | 0 | 2 | 6 |
| M | T | 0 | 3 | 6 | 0 | 5 | 6 |  | : |  | C | A | T | C | T | A | G | G | T | A | A | C | A | C | T | A | G | T | A | G | A | A | G | A | A | T | G | C | T | A | G | G | A | G | A | A | A | G | A | C | C | T | A | G | G | A | T | G | A | T | T | G | T | C | A | T | T | G | A | G | G | A | C | G | A | T | G | A | A | G | A | A | C | A | G | A | C | C | C | C | T | C | A | T | G | A | C | A | G | A | C | A | A | T | T | A | C | A | A | G | A | A | A | G | A | G | C | A | T | T | A | G | C | T | A | G | A | T | C | C | C | A | A | T | C | T | A | G | G | A | T | G | C | T | A | G | G | T | T | T | A | A | G | A | C | C | C | A | C | A | G | A | G | G | A | T | C | T | T | A | G | A | A | C | C | A | C | C | A | G | T |  | : |  | 1 | 0 | 2 | 6 |
| M | T | 0 | 3 | 6 | 0 | 5 | 7 |  | : |  | C | A | T | C | T | A | G | G | T | A | A | C | A | C | T | A | G | T | A | G | A | A | G | A | A | T | G | C | T | A | G | G | A | G | A | A | A | G | A | C | C | T | A | G | G | A | T | G | A | T | T | G | T | C | A | T | T | G | A | G | G | A | C | G | A | T | G | A | A | G | A | A | C | A | G | A | C | C | C | C | T | C | A | T | G | A | C | A | G | A | C | A | A | T | T | A | C | A | A | G | A | A | A | G | A | G | C | A | T | T | A | G | C | T | A | G | A | T | C | C | C | A | A | T | C | T | A | G | G | A | T | G | C | T | A | G | G | T | T | T | A | A | G | A | C | C | C | A | C | A | G | A | G | G | A | T | C | T | T | A | G | A | A | C | C | A | C | C | A | G | T |  | : |  | 1 | 0 | 2 | 6 |
| K | X | 2 | 4 | 9 | 7 | 3 | 8 |  | : |  | C | A | T | C | T | G | G | G | T | A | A | C | A | C | C | A | G | T | A | G | A | A | G | A | A | T | G | C | T | A | G | G | A | G | A | A | A | A | A | C | C | T | A | G | G | A | T | G | A | T | T | G | T | C | A | T | T | G | A | G | G | A | C | G | A | T | G | A | A | G | A | A | C | A | G | A | C | C | C | C | T | C | A | T | G | A | C | A | G | A | C | A | A | T | T | A | C | A | A | G | A | A | A | G | A | G | C | A | T | T | A | G | C | T | A | G | A | T | C | C | C | A | A | T | C | T | A | G | G | A | T | G | C | T | A | G | G | T | T | T | A | A | G | A | C | C | C | A | C | A | G | A | G | G | A | T | C | T | T | A | G | A | A | C | C | A | C | T | A | G | T |  | : |  | 1 | 0 | 2 | 6 |
| K | X | 2 | 4 | 9 | 7 | 3 | 7 |  | : |  | C | A | T | C | T | G | G | G | T | A | A | C | A | C | C | A | G | T | A | G | A | A | G | A | A | T | G | C | T | A | G | G | A | G | A | A | A | A | A | C | C | T | A | G | G | A | T | G | A | T | T | G | T | C | A | T | T | G | A | G | G | A | C | G | A | T | G | A | A | G | A | A | C | A | G | A | C | C | C | C | T | C | A | T | G | A | C | A | G | A | C | A | A | T | T | A | C | A | A | G | A | A | A | G | A | G | C | A | T | T | A | G | C | T | A | G | A | T | C | C | C | A | A | T | C | T | A | G | G | A | T | G | C | T | A | G | G | T | T | T | A | A | G | A | C | C | C | A | C | A | G | A | G | G | A | T | C | T | T | A | G | A | A | C | C | A | C | T | A | G | T |  | : |  | 1 | 0 | 2 | 6 |
| K | X | 2 | 4 | 9 | 7 | 3 | 6 |  | : |  | C | A | T | C | T | G | G | G | T | A | A | C | A | C | C | A | G | T | A | G | A | A | G | A | A | T | G | C | T | A | G | G | A | G | A | A | A | A | A | C | C | T | A | G | G | A | T | G | A | T | T | G | T | C | A | T | T | G | A | G | G | A | C | G | A | T | G | A | A | G | A | A | C | A | G | A | C | C | C | C | T | C | A | T | G | A | C | A | G | A | C | A | A | T | T | A | C | A | A | G | A | A | A | G | A | G | C | A | T | T | A | G | C | T | A | G | A | T | C | C | C | A | A | T | C | T | A | G | G | A | T | G | C | T | A | G | G | T | T | T | A | A | G | A | C | C | C | A | C | A | G | A | G | G | A | T | C | T | T | A | G | A | A | C | C | A | C | T | A | G | T |  | : |  | 1 | 0 | 2 | 6 |
| K | X | 2 | 4 | 9 | 7 | 3 | 5 |  | : |  | C | A | T | C | T | G | G | G | T | A | A | C | A | C | C | A | G | T | A | G | A | A | G | A | A | T | G | C | T | A | G | G | A | G | A | G | A | G | A | C | C | T | A | G | G | A | T | G | A | T | T | G | T | C | A | T | T | G | A | G | G | A | C | G | A | T | G | A | A | G | A | A | C | A | G | A | C | C | C | C | T | C | A | T | G | A | C | A | G | A | C | A | A | T | T | A | C | A | A | G | A | A | A | G | A | G | C | A | T | T | A | G | C | T | A | G | A | T | C | C | C | A | A | T | C | T | A | G | G | A | T | G | C | T | A | G | G | T | T | T | A | A | G | A | C | C | C | A | C | A | G | A | G | G | A | T | C | T | T | A | G | A | A | C | C | A | C | C | A | G | T |  | : |  | 1 | 0 | 2 | 6 |
| M | F | 1 | 9 | 7 | 9 | 1 | 6 |  | : |  | C | A | T | C | T | G | G | G | T | A | A | C | A | C | C | A | G | T | A | G | A | A | G | A | A | T | G | C | T | A | G | G | A | G | A | A | A | A | A | C | C | T | A | G | G | A | T | G | A | T | T | G | T | C | A | T | T | G | A | G | G | A | C | G | A | T | G | A | A | G | A | A | C | A | G | A | C | C | C | C | T | C | A | T | G | A | C | A | G | A | C | A | A | T | T | A | C | A | A | G | A | A | A | G | A | G | C | A | T | T | A | G | C | T | A | G | A | T | C | C | C | A | A | T | C | T | A | G | G | A | T | G | C | T | A | G | G | T | T | T | A | A | G | A | C | C | C | A | C | A | G | A | G | G | A | T | C | T | T | A | G | A | A | C | C | A | C | T | A | G | T |  | : |  | 1 | 0 | 2 | 6 |
| K | T | 2 | 5 | 0 | 6 | 3 | 2 |  | : |  | C | A | T | C | T | G | G | G | T | A | A | C | A | C | C | A | G | T | A | G | A | A | G | A | A | T | G | C | T | A | G | G | A | G | A | A | A | G | A | C | C | T | A | G | G | A | T | G | A | T | T | G | T | C | A | T | T | G | A | G | G | A | C | G | A | T | G | A | A | G | A | A | C | A | G | A | C | C | C | C | T | C | A | T | G | A | C | A | G | A | C | A | A | T | T | A | C | A | A | G | A | A | A | G | A | G | C | A | T | T | A | G | C | T | A | G | A | T | C | C | C | A | A | T | C | T | A | G | G | A | T | G | C | T | A | G | G | T | T | T | A | A | G | A | C | C | C | A | C | A | G | A | G | G | A | T | C | T | T | A | G | A | A | C | C | A | C | T | A | G | C |  | : |  | 1 | 0 | 2 | 6 |
|  |  |  |  |  |  |  |  |  |  |  | C | A | T | C | T | g | G | G | T | A | A | c | A | C | c | A | g | T | A | G | A | A | G | A | A | T | G | C | T | A | G | G | A | G | A | a | A |  | A | C | C | T | A | G | G | A | T | G | A | T | T | G | T | C | A | T | T | G | A | G | G | A | C | G | A | T | G | A | A | G | A | A | C | A | G | A | c | C | C | C | T | C | A | T | G | A | C | A | G | A | C | a | A | T | T | A | C | A | A | G | A | a | A | G | A | G | C | A | T | T | A | G | C | T | A | G | A | T | C | C | C | A | A | T | C | T | A | G | G | A | T | G | C | T | A | G | G | T | T | T | A | A | G | A | C | C | C | A | C | A | G | A | G | G | A | T | C | T | T | A | G | A | A | C | C | A | C |  | A | G | t |  |  |  |  |  |  |  |

|  |  |  |  |  |  |  |  |  |  |  |  |  |  |  |  |  |  |  |  |  |  |  |  |  |  |  |  |  |  |  |  |  |  |  |  |  |  |  |  |  |  |  |  |  |  |  |  |  |  |  |  |  |  |  |  |  |  |  |  |  |  |  |  |  |  |  |  |  |  |  |  |  |  |  |  |  |  |  |  |  |  |  |  |  |  |  |  |  |  |  |  |  |  |  |  |  |  |  |  |  |  |  |  |  |  |  |  |  |  |  |  |  |  |  |  |  |  |  |  |  |  |  |  |  |  |  |  |  |  |  |  |  |  |  |  |  |  |  |  |  |  |  |  |  |  |  |  |  |  |  |  |  |  |  |  |  |  |  |  |  |  |  |  |  |  |  |  |  |  |  |  |  |  |  |  |  |  |  |  |  |  |  |  |  |  |  |  |  |
| --- | --- | --- | --- | --- | --- | --- | --- | --- | --- | --- | --- | --- | --- | --- | --- | --- | --- | --- | --- | --- | --- | --- | --- | --- | --- | --- | --- | --- | --- | --- | --- | --- | --- | --- | --- | --- | --- | --- | --- | --- | --- | --- | --- | --- | --- | --- | --- | --- | --- | --- | --- | --- | --- | --- | --- | --- | --- | --- | --- | --- | --- | --- | --- | --- | --- | --- | --- | --- | --- | --- | --- | --- | --- | --- | --- | --- | --- | --- | --- | --- | --- | --- | --- | --- | --- | --- | --- | --- | --- | --- | --- | --- | --- | --- | --- | --- | --- | --- | --- | --- | --- | --- | --- | --- | --- | --- | --- | --- | --- | --- | --- | --- | --- | --- | --- | --- | --- | --- | --- | --- | --- | --- | --- | --- | --- | --- | --- | --- | --- | --- | --- | --- | --- | --- | --- | --- | --- | --- | --- | --- | --- | --- | --- | --- | --- | --- | --- | --- | --- | --- | --- | --- | --- | --- | --- | --- | --- | --- | --- | --- | --- | --- | --- | --- | --- | --- | --- | --- | --- | --- | --- | --- | --- | --- | --- | --- | --- | --- | --- | --- | --- | --- | --- | --- | --- | --- | --- | --- |
|  |  |  |  |  |  |  |  |  |  |  |  |  |  |  |  |  |  |  |  |  |  |  |  |  |  |  |  |  |  |  |  |  |  |  |  |  |  |  |  |  |  |  |  |  |  |  |  |  |  |  |  |  |  |  |  |  |  |  |  |  |  |  |  |  |  |  |  |  |  |  |  |  |  |  |  |  |  |  |  |  |  |  |  |  |  |  |  |  |  |  |  |  |  |  |  |  |  |  |  |  |  |  |  |  |  |  |  |  |  |  |  |  |  |  |  |  |  |  |  |  |  |  |  |  |  |  |  |  |  |  |  |  |  |  |  |  |  |  |  |  |  |  |  |  |  |  |  |  |  |  |  |  |  |  |  |  |  |  |  |  |  |  |  |  |  |  |  |  |  |  |  |  |  |  |  |  |  |  |  |  |  |  |  |  |  |  |  |  |
|  |  |  |  |  |  |  |  |  |  |  |  |  |  | \* |  |  |  |  |  |  | 1 | 0 | 4 | 0 |  |  |  |  |  |  |  |  |  | \* |  |  |  |  |  |  | 1 | 0 | 6 | 0 |  |  |  |  |  |  |  |  |  | \* |  |  |  |  |  |  | 1 | 0 | 8 | 0 |  |  |  |  |  |  |  |  |  | \* |  |  |  |  |  |  | 1 | 1 | 0 | 0 |  |  |  |  |  |  |  |  |  | \* |  |  |  |  |  |  | 1 | 1 | 2 | 0 |  |  |  |  |  |  |  |  |  | \* |  |  |  |  |  |  | 1 | 1 | 4 | 0 |  |  |  |  |  |  |  |  |  | \* |  |  |  |  |  |  | 1 | 1 | 6 | 0 |  |  |  |  |  |  |  |  |  | \* |  |  |  |  |  |  | 1 | 1 | 8 | 0 |  |  |  |  |  |  |  |  |  | \* |  |  |  |  |  |  | 1 |  |  |  |  |  |  |  |
| M | N | 9 | 5 | 6 | 5 | 2 | 0 |  | : |  | C | G | C | A | G | G | A | T | T | A | A | C | A | G | C | C | T | A | G | C | A | C | A | C | C | A | A | T | T | A | T | A | A | A | T | A | T | G | G | C | A | T | A | C | A | A | A | G | A | C | C | A | A | C | C | C | C | A | T | A | T | T | T | A | T | T | A | C | A | A | G | A | A | G | C | A | A | T | A | C | C | T | A | G | C | T | C | T | C | T | A | C | C | A | T | C | A | A | A | A | C | G | T | A | G | A | A | A | G | A | G | G | T | A | G | C | C | C | C | G | A | G | T | A | T | T | T | C | T | T | T | A | C | C | G | G | A | G | G | A | A | A | A | G | G | A | A | T | T | G | A | A | G | G | A | T | G | T | T | T | G | A | A | A | C | A | C | C | T | C | A |  | : |  | 1 | 1 | 9 | 7 |
| M | T | 0 | 1 | 2 | 7 | 3 | 2 |  | : |  | C | G | C | A | G | G | A | T | T | A | A | C | A | G | C | C | T | A | G | C | A | C | A | C | C | A | A | T | T | A | T | A | A | A | T | A | T | G | G | C | G | T | A | C | A | A | A | G | A | C | C | A | A | C | C | C | C | A | T | A | T | T | T | A | T | T | A | C | A | A | G | A | A | G | C | A | A | T | A | C | C | T | A | G | C | T | C | T | C | T | A | C | C | A | T | C | A | A | A | A | C | G | T | A | G | A | A | A | G | A | G | G | T | A | G | C | C | C | C | G | A | G | T | A | T | T | T | C | T | T | T | A | C | C | G | G | A | G | G | A | A | A | A | G | G | A | A | T | T | G | A | A | G | G | A | T | G | T | T | T | G | A | A | A | C | A | C | C | T | C | A |  | : |  | 1 | 1 | 9 | 7 |
| M | T | 0 | 1 | 2 | 7 | 3 | 4 |  | : |  | C | G | C | A | G | G | A | T | T | A | A | C | A | G | C | C | T | A | G | C | A | C | A | C | C | A | A | T | T | A | T | A | A | A | T | A | T | G | G | C | G | T | A | C | A | A | A | G | A | C | C | A | A | C | C | C | C | A | T | A | T | T | T | A | T | T | A | C | A | A | G | A | A | G | C | A | A | T | A | C | C | T | A | G | C | T | C | T | C | T | A | C | C | A | T | C | A | A | A | A | C | G | T | A | G | A | A | A | G | A | G | G | T | A | G | C | C | C | C | G | A | G | T | A | T | T | T | C | T | T | T | A | C | C | G | G | A | G | G | A | A | A | A | G | G | A | A | T | T | G | A | A | G | G | A | T | G | T | T | T | G | A | A | A | C | A | C | C | T | C | A |  | : |  | 1 | 1 | 9 | 7 |
| M | T | 0 | 2 | 7 | 0 | 0 | 6 |  | : |  | C | G | C | A | G | G | A | T | T | A | A | C | A | G | C | C | T | A | G | C | A | C | A | C | C | A | A | T | T | A | T | A | A | A | T | A | T | G | G | C | G | T | A | C | A | A | A | G | A | C | C | A | A | C | C | C | C | A | T | A | T | T | T | A | T | T | A | C | A | A | G | A | A | G | C | A | A | T | A | C | C | T | A | G | C | T | C | T | C | T | A | C | C | A | T | C | A | A | A | A | C | G | T | A | G | A | A | A | G | A | G | G | T | A | G | C | C | C | C | G | A | G | T | A | T | T | T | C | T | T | T | A | C | C | G | G | A | G | G | A | A | A | A | G | G | A | A | T | T | G | A | A | G | G | A | T | G | T | T | T | G | A | A | A | C | A | C | C | T | C | A |  | : |  | 1 | 1 | 9 | 7 |
| M | T | 0 | 2 | 7 | 0 | 0 | 7 |  | : |  | C | G | C | A | G | G | A | T | T | A | A | C | A | G | C | C | T | A | G | C | A | C | A | C | C | A | A | T | T | A | T | A | A | A | T | A | T | G | G | C | G | T | A | C | A | A | A | G | A | C | C | A | A | C | C | C | C | A | T | A | T | T | T | A | T | T | A | C | A | A | G | A | A | G | C | A | A | T | A | C | C | T | A | G | C | T | C | T | C | T | A | C | C | A | T | C | A | A | A | A | C | G | T | A | G | A | A | A | G | A | G | G | T | A | G | C | C | C | C | G | A | G | T | A | T | T | T | C | T | T | T | A | C | C | G | G | A | G | G | A | A | A | A | G | G | A | A | T | T | G | A | A | G | G | A | T | G | T | T | T | G | A | A | A | C | A | C | C | T | C | A |  | : |  | 1 | 1 | 9 | 7 |
| M | T | 0 | 3 | 6 | 0 | 5 | 3 |  | : |  | C | G | C | A | G | G | A | T | T | A | A | C | A | G | C | C | T | A | G | C | A | C | A | C | C | A | A | T | T | A | T | A | A | A | T | A | T | G | G | C | G | T | A | C | A | A | A | G | A | C | C | A | A | C | C | C | C | A | T | A | T | T | T | A | T | T | A | C | A | A | G | A | A | G | C | A | A | T | A | C | C | T | A | G | C | T | C | T | C | T | A | C | C | A | T | C | A | A | A | A | C | G | T | A | G | A | A | A | G | A | G | G | T | A | G | C | C | C | C | G | A | G | T | A | T | T | T | C | T | T | T | A | C | C | G | G | A | G | G | A | A | A | A | G | G | A | A | T | T | G | A | A | G | G | A | T | G | T | T | T | G | A | A | A | C | A | C | C | T | C | A |  | : |  | 1 | 1 | 9 | 7 |
| M | T | 0 | 3 | 6 | 0 | 5 | 4 |  | : |  | C | G | C | A | G | G | A | T | T | A | A | C | A | G | C | C | T | A | G | C | A | C | A | C | C | A | A | T | T | A | T | A | A | A | T | A | T | G | G | C | G | T | A | C | A | A | A | G | A | C | C | A | A | C | C | C | C | A | T | A | T | T | T | A | T | T | A | C | A | A | G | A | A | G | C | A | A | T | A | C | C | T | A | G | C | T | C | T | C | T | A | C | C | A | T | C | A | A | A | A | C | G | T | A | G | A | A | A | G | A | G | G | T | A | G | C | C | C | C | G | A | G | T | A | T | T | T | C | T | T | T | A | C | C | G | G | A | G | G | A | A | A | A | G | G | A | A | T | T | G | A | A | G | G | A | T | G | T | T | T | G | A | A | A | C | A | C | C | T | C | A |  | : |  | 1 | 1 | 9 | 7 |
| M | T | 0 | 3 | 6 | 0 | 5 | 5 |  | : |  | C | G | C | A | G | G | A | T | T | A | A | C | A | G | C | C | T | A | G | C | A | C | A | C | C | A | A | T | T | A | T | A | A | A | T | A | T | G | G | C | G | T | A | C | A | A | A | G | A | C | C | A | A | C | C | C | C | A | T | A | T | T | T | A | T | T | A | C | A | A | G | A | A | G | C | A | A | T | A | C | C | T | A | G | C | T | C | T | C | T | A | C | C | A | T | C | A | A | A | A | C | G | T | A | G | A | A | A | G | A | G | G | T | A | G | C | C | C | C | G | A | G | T | A | T | T | T | C | T | T | T | A | C | C | A | G | A | G | G | A | A | A | A | G | G | A | A | T | T | G | A | A | G | G | A | T | G | T | T | T | G | A | A | A | C | A | C | C | T | C | A |  | : |  | 1 | 1 | 9 | 7 |
| M | T | 0 | 3 | 6 | 0 | 5 | 6 |  | : |  | C | G | C | A | G | G | A | T | T | A | A | C | A | G | C | C | T | A | G | C | A | C | A | C | C | A | A | T | T | A | T | A | A | A | T | A | T | G | G | C | G | T | A | C | A | A | A | G | A | C | C | A | A | C | C | C | C | A | T | A | T | T | T | A | T | T | A | C | A | A | G | A | A | G | C | A | A | T | A | C | C | T | A | G | C | T | C | T | C | T | A | C | C | A | T | C | A | A | A | A | C | G | T | A | G | A | A | A | G | A | G | G | T | A | G | C | C | C | C | G | A | G | T | A | T | T | T | C | T | T | T | A | C | C | G | G | A | G | G | A | A | A | A | G | G | A | A | T | T | G | A | A | G | G | A | T | G | T | T | T | G | A | A | A | C | A | C | C | T | C | A |  | : |  | 1 | 1 | 9 | 7 |
| M | T | 0 | 3 | 6 | 0 | 5 | 7 |  | : |  | C | G | C | A | G | G | A | T | T | A | A | C | A | G | C | C | T | A | G | C | A | C | A | C | C | A | A | T | T | A | T | A | A | A | T | A | T | G | G | C | G | T | G | C | A | A | A | G | A | C | C | A | A | C | C | C | C | A | T | A | T | T | T | A | T | T | A | C | A | A | G | A | A | G | C | A | A | T | A | C | C | T | A | G | C | T | C | T | C | T | A | C | C | G | T | C | A | A | A | A | C | G | T | A | G | A | A | A | G | A | G | G | T | A | G | C | C | C | C | G | A | G | T | A | T | T | T | C | T | T | T | A | C | C | G | G | A | G | G | A | A | A | A | G | G | A | A | T | T | G | A | A | G | G | A | T | G | T | T | T | G | A | A | A | C | A | C | C | T | C | A |  | : |  | 1 | 1 | 9 | 7 |
| K | X | 2 | 4 | 9 | 7 | 3 | 8 |  | : |  | C | G | C | A | G | G | A | T | T | A | A | C | A | G | C | C | T | A | G | C | A | C | A | C | C | A | A | T | T | A | T | A | A | A | T | A | T | G | G | C | G | T | A | C | A | A | A | G | A | C | C | A | A | C | C | C | C | A | T | A | T | T | T | A | T | T | A | C | A | A | G | A | A | G | C | A | A | T | A | C | C | T | A | G | C | T | C | T | C | T | A | C | C | A | T | C | A | A | A | A | C | G | T | A | G | A | A | A | G | A | G | G | T | A | G | C | C | C | C | G | A | G | T | A | T | T | T | C | T | T | T | A | C | C | G | G | A | G | G | A | A | A | A | G | G | A | A | T | T | G | A | A | G | G | A | T | G | T | T | T | G | A | A | A | C | A | C | C | T | C | A |  | : |  | 1 | 1 | 9 | 7 |
| K | X | 2 | 4 | 9 | 7 | 3 | 7 |  | : |  | C | G | C | A | G | G | A | T | T | A | A | C | A | G | C | C | T | A | G | C | A | C | A | C | C | A | A | T | T | A | T | A | A | A | T | A | T | G | G | C | G | T | A | C | A | A | A | G | A | C | C | A | A | C | C | C | C | A | T | A | T | T | T | A | T | T | A | C | A | A | G | A | A | G | C | A | A | T | A | C | C | T | A | G | C | T | C | T | C | T | A | C | C | A | T | C | A | A | A | A | C | G | T | A | G | A | A | A | G | A | G | G | T | A | G | C | C | C | C | G | A | G | T | A | T | T | T | C | T | T | T | A | C | C | G | G | A | G | G | A | A | A | A | G | G | A | A | T | T | G | A | A | G | G | A | T | G | T | T | T | G | A | A | A | C | A | C | C | T | C | A |  | : |  | 1 | 1 | 9 | 7 |
| K | X | 2 | 4 | 9 | 7 | 3 | 6 |  | : |  | C | G | C | A | G | G | A | T | T | A | A | C | A | G | C | C | T | A | G | C | A | C | A | C | C | A | A | T | T | A | T | A | A | A | T | A | T | G | G | C | G | T | A | C | A | A | A | G | A | C | C | A | A | C | C | C | C | A | T | A | T | T | T | A | T | T | A | C | A | A | G | A | A | G | C | A | A | T | A | C | C | T | A | G | C | T | C | T | C | T | A | C | C | A | T | C | A | A | A | A | C | G | T | A | G | A | A | A | G | A | G | G | T | A | G | C | C | C | C | G | A | G | T | A | T | T | T | C | T | T | T | A | C | C | G | G | A | G | G | A | A | A | A | G | G | A | A | T | T | G | A | A | G | G | A | T | G | T | T | T | G | A | A | A | C | A | C | C | T | C | A |  | : |  | 1 | 1 | 9 | 7 |
| K | X | 2 | 4 | 9 | 7 | 3 | 5 |  | : |  | C | G | C | A | G | G | A | T | T | A | A | C | A | G | C | C | T | A | G | C | A | C | A | C | C | A | A | T | T | A | T | A | A | A | T | A | T | G | G | C | G | T | A | C | A | A | A | G | A | C | C | A | A | C | C | C | C | A | T | A | T | T | T | A | T | T | A | C | A | A | G | A | A | G | C | A | A | T | A | C | C | T | A | G | C | T | C | T | C | T | A | C | C | A | T | C | A | A | A | A | C | G | T | A | G | A | A | A | G | A | G | G | T | A | G | C | C | C | C | G | A | G | T | A | T | T | T | C | T | T | T | A | C | C | G | G | A | G | G | A | A | A | A | G | G | A | A | T | T | G | A | A | G | G | A | T | G | T | T | T | G | A | A | A | C | A | C | C | T | C | A |  | : |  | 1 | 1 | 9 | 7 |
| M | F | 1 | 9 | 7 | 9 | 1 | 6 |  | : |  | C | G | C | A | G | G | A | T | T | A | A | C | A | G | C | C | T | A | G | C | A | C | A | C | C | A | A | T | T | A | T | A | A | A | T | A | T | G | G | C | G | T | A | C | A | A | A | G | A | C | C | A | A | C | C | C | C | A | T | A | T | T | T | A | T | T | A | C | A | A | G | A | A | G | C | A | A | T | A | C | C | T | A | G | C | T | C | T | C | T | A | C | C | A | T | C | A | A | A | A | C | G | T | A | G | A | A | A | G | A | G | G | T | A | G | C | C | C | C | G | A | G | T | A | T | T | T | C | T | T | T | A | C | C | G | G | A | G | G | A | A | A | A | G | G | A | A | T | T | G | A | A | G | G | A | T | G | T | T | T | G | A | A | A | C | A | C | C | T | C | A |  | : |  | 1 | 1 | 9 | 7 |
| K | T | 2 | 5 | 0 | 6 | 3 | 2 |  | : |  | C | G | C | A | G | G | A | T | T | A | A | C | A | G | C | C | T | A | G | C | A | C | A | C | C | A | A | T | T | A | T | A | A | A | T | A | T | G | G | C | G | T | A | C | A | A | A | G | A | C | C | A | A | C | C | C | C | A | T | A | T | T | T | A | T | T | A | C | A | A | G | A | A | G | C | A | A | T | A | C | C | T | A | G | C | T | C | T | C | T | A | C | C | A | T | C | A | A | A | A | C | G | T | A | G | A | A | A | G | A | G | G | T | A | G | C | C | C | C | G | A | G | T | A | T | T | T | C | T | T | T | A | C | C | G | G | A | G | G | A | A | A | A | G | G | A | A | T | T | G | A | A | G | G | A | T | G | T | T | T | G | A | A | A | C | A | C | C | T | C | A |  | : |  | 1 | 1 | 9 | 7 |
|  |  |  |  |  |  |  |  |  |  |  | C | G | C | A | G | G | A | T | T | A | A | C | A | G | C | C | T | A | G | C | A | C | A | C | C | A | A | T | T | A | T | A | A | A | T | A | T | G | G | C | g | T | a | C | A | A | A | G | A | C | C | A | A | C | C | C | C | A | T | A | T | T | T | A | T | T | A | C | A | A | G | A | A | G | C | A | A | T | A | C | C | T | A | G | C | T | C | T | C | T | A | C | C | a | T | C | A | A | A | A | C | G | T | A | G | A | A | A | G | A | G | G | T | A | G | C | C | C | C | G | A | G | T | A | T | T | T | C | T | T | T | A | C | C | g | G | A | G | G | A | A | A | A | G | G | A | A | T | T | G | A | A | G | G | A | T | G | T | T | T | G | A | A | A | C | A | C | C | T | C | A |  |  |  |  |  |  |  |

|  |  |  |  |  |  |  |  |  |  |  |  |  |  |  |  |  |  |  |  |  |  |  |  |  |  |  |  |  |  |  |  |  |  |  |  |  |  |  |  |  |  |  |  |  |  |  |  |  |  |  |  |  |  |  |  |  |  |  |  |  |  |  |  |  |  |  |  |  |  |  |  |  |  |  |  |  |  |  |  |  |  |  |  |  |  |  |  |  |  |  |  |  |  |  |  |  |  |  |  |  |  |  |  |  |  |  |  |  |  |  |  |  |  |  |  |  |  |  |  |  |  |  |  |  |  |  |  |  |  |  |  |  |  |  |  |  |  |  |  |  |  |  |  |  |  |  |  |  |  |  |  |  |  |  |  |  |  |  |  |  |  |  |  |  |  |  |  |  |  |  |  |  |  |  |  |  |  |  |  |  |  |  |  |  |  |  |  |  |
| --- | --- | --- | --- | --- | --- | --- | --- | --- | --- | --- | --- | --- | --- | --- | --- | --- | --- | --- | --- | --- | --- | --- | --- | --- | --- | --- | --- | --- | --- | --- | --- | --- | --- | --- | --- | --- | --- | --- | --- | --- | --- | --- | --- | --- | --- | --- | --- | --- | --- | --- | --- | --- | --- | --- | --- | --- | --- | --- | --- | --- | --- | --- | --- | --- | --- | --- | --- | --- | --- | --- | --- | --- | --- | --- | --- | --- | --- | --- | --- | --- | --- | --- | --- | --- | --- | --- | --- | --- | --- | --- | --- | --- | --- | --- | --- | --- | --- | --- | --- | --- | --- | --- | --- | --- | --- | --- | --- | --- | --- | --- | --- | --- | --- | --- | --- | --- | --- | --- | --- | --- | --- | --- | --- | --- | --- | --- | --- | --- | --- | --- | --- | --- | --- | --- | --- | --- | --- | --- | --- | --- | --- | --- | --- | --- | --- | --- | --- | --- | --- | --- | --- | --- | --- | --- | --- | --- | --- | --- | --- | --- | --- | --- | --- | --- | --- | --- | --- | --- | --- | --- | --- | --- | --- | --- | --- | --- | --- | --- | --- | --- | --- | --- | --- | --- | --- | --- | --- | --- |
|  |  |  |  |  |  |  |  |  |  |  |  |  |  |  |  |  |  |  |  |  |  |  |  |  |  |  |  |  |  |  |  |  |  |  |  |  |  |  |  |  |  |  |  |  |  |  |  |  |  |  |  |  |  |  |  |  |  |  |  |  |  |  |  |  |  |  |  |  |  |  |  |  |  |  |  |  |  |  |  |  |  |  |  |  |  |  |  |  |  |  |  |  |  |  |  |  |  |  |  |  |  |  |  |  |  |  |  |  |  |  |  |  |  |  |  |  |  |  |  |  |  |  |  |  |  |  |  |  |  |  |  |  |  |  |  |  |  |  |  |  |  |  |  |  |  |  |  |  |  |  |  |  |  |  |  |  |  |  |  |  |  |  |  |  |  |  |  |  |  |  |  |  |  |  |  |  |  |  |  |  |  |  |  |  |  |  |  |  |
|  |  |  |  |  |  |  |  |  |  |  | 2 | 0 | 0 |  |  |  |  |  |  |  |  |  | \* |  |  |  |  |  |  | 1 | 2 | 2 | 0 |  |  |  |  |  |  |  |  |  | \* |  |  |  |  |  |  | 1 | 2 | 4 | 0 |  |  |  |  |  |  |  |  |  | \* |  |  |  |  |  |  | 1 | 2 | 6 | 0 |  |  |  |  |  |  |  |  |  | \* |  |  |  |  |  |  | 1 | 2 | 8 | 0 |  |  |  |  |  |  |  |  |  | \* |  |  |  |  |  |  | 1 | 3 | 0 | 0 |  |  |  |  |  |  |  |  |  | \* |  |  |  |  |  |  | 1 | 3 | 2 | 0 |  |  |  |  |  |  |  |  |  | \* |  |  |  |  |  |  | 1 | 3 | 4 | 0 |  |  |  |  |  |  |  |  |  | \* |  |  |  |  |  |  | 1 | 3 | 6 | 0 |  |  |  |  |  |  |  |  |  |  |  |  |  |  |  |
| M | N | 9 | 5 | 6 | 5 | 2 | 0 |  | : |  | A | T | A | A | T | A | T | C | A | A | T | A | C | C | G | T | C | T | G | C | G | G | A | G | G | T | A | T | T | C | A | T | A | T | A | A | T | G | G | T | T | T | A | C | T | T | T | A | T | A | T | G | T | A | A | G | A | C | T | T | T | A | G | G | A | A | T | A | A | A | G | C | C | C | T | A | C | G | T | A | T | A | T | A | G | G | A | A | C | A | C | C | C | C | T | G | A | A | G | A | A | G | G | A | A | G | C | A | A | T | C | T | A | G | G | A | T | T | A | C | T | A | T | C | C | C | T | C | T | T | A | G | G | A | A | A | C | A | A | A | C | C | C | T | C | A | G | A | G | A | G | T | T | C | G | T | C | A | G | A | A | A | A | T | C | C | T | C | T | A | G |  | : |  | 1 | 3 | 6 | 8 |
| M | T | 0 | 1 | 2 | 7 | 3 | 2 |  | : |  | A | T | A | A | T | A | T | C | A | A | T | A | C | C | G | T | C | T | G | C | G | G | A | G | G | T | A | T | T | C | A | T | A | T | A | A | T | G | G | T | T | T | A | C | T | T | T | A | T | A | T | G | T | A | A | G | A | C | T | T | T | A | G | G | A | A | T | A | A | A | G | C | C | C | T | A | C | G | T | A | T | A | T | A | G | G | A | A | C | A | C | C | C | C | T | G | A | A | G | A | A | G | G | A | A | G | C | A | A | T | C | T | A | G | G | A | T | T | A | C | T | G | T | C | C | C | T | C | T | T | A | G | G | A | A | A | C | A | A | A | C | C | C | T | C | A | G | A | G | A | G | T | T | C | G | T | C | A | G | A | A | A | A | C | C | C | T | C | T | A | G |  | : |  | 1 | 3 | 6 | 8 |
| M | T | 0 | 1 | 2 | 7 | 3 | 4 |  | : |  | A | T | A | A | T | A | T | C | A | A | T | A | C | C | G | T | C | T | G | C | G | G | A | G | G | T | A | T | T | C | A | T | A | T | A | A | T | G | G | T | T | T | A | C | T | T | T | A | T | A | T | G | T | A | A | G | A | C | T | T | T | A | G | G | A | A | T | A | A | A | G | C | C | C | T | A | C | G | T | A | T | A | T | A | G | G | A | A | C | A | C | C | C | C | T | G | A | A | G | A | T | G | G | A | A | G | T | A | A | T | C | T | A | G | G | A | T | T | A | T | T | A | T | C | C | C | T | C | T | T | A | G | G | A | A | A | C | A | A | A | C | C | C | T | C | A | G | A | G | A | G | T | T | C | G | T | C | A | G | A | A | A | A | C | C | C | T | C | T | A | G |  | : |  | 1 | 3 | 6 | 8 |
| M | T | 0 | 2 | 7 | 0 | 0 | 6 |  | : |  | A | T | A | A | T | A | T | C | A | A | T | A | C | C | G | T | C | T | G | C | G | G | A | G | G | T | A | T | T | C | A | T | A | T | A | A | T | G | G | T | T | T | A | C | T | T | T | A | T | A | T | G | T | A | A | G | A | C | T | T | T | A | G | G | A | A | T | A | A | A | G | C | C | C | T | A | C | G | T | A | T | A | T | A | G | G | A | A | C | A | C | C | C | C | T | G | A | A | G | A | A | G | G | A | A | G | C | A | A | T | C | T | A | G | G | A | T | T | A | C | T | G | T | C | C | C | T | C | T | T | A | G | G | A | A | A | C | A | A | A | C | C | C | T | C | A | G | A | G | A | G | T | T | C | G | T | C | A | G | A | A | A | A | C | C | C | T | C | T | A | G |  | : |  | 1 | 3 | 6 | 8 |
| M | T | 0 | 2 | 7 | 0 | 0 | 7 |  | : |  | A | T | A | A | T | A | T | C | A | A | T | A | C | C | G | T | C | T | G | C | G | G | A | G | G | T | A | T | T | C | A | T | A | T | A | A | T | G | G | T | T | T | A | C | T | T | T | A | T | A | T | G | T | A | A | G | A | C | T | T | T | A | G | G | A | A | T | A | A | A | G | C | C | C | T | A | C | G | T | A | T | A | T | A | G | G | A | A | C | A | C | C | C | C | T | G | A | A | G | A | T | G | G | A | A | G | T | A | A | T | C | T | A | G | G | A | T | T | A | T | T | A | T | C | C | C | T | C | T | T | A | G | G | A | A | A | C | A | A | A | C | C | C | T | C | A | G | A | G | A | A | T | T | C | G | T | C | A | G | A | A | A | A | T | C | C | T | C | T | A | G |  | : |  | 1 | 3 | 6 | 8 |
| M | T | 0 | 3 | 6 | 0 | 5 | 3 |  | : |  | A | T | A | A | T | A | T | C | A | A | T | A | C | C | G | T | C | T | G | C | G | G | A | G | G | T | A | T | T | C | A | T | A | T | A | A | T | G | G | T | T | T | A | C | T | T | T | A | T | A | T | G | T | A | A | G | A | C | T | T | T | A | G | G | A | A | T | A | A | A | G | C | C | C | T | A | C | G | T | A | T | A | T | A | G | G | A | A | C | A | C | C | C | C | T | G | A | A | G | A | T | G | G | A | A | G | T | A | A | T | C | T | A | G | G | A | T | T | A | T | T | A | T | C | C | C | T | C | T | T | A | G | G | A | A | A | C | A | A | A | C | C | C | T | C | A | G | A | G | A | A | T | T | C | G | T | C | A | G | A | A | A | A | T | C | C | T | C | T | A | G |  | : |  | 1 | 3 | 6 | 8 |
| M | T | 0 | 3 | 6 | 0 | 5 | 4 |  | : |  | A | T | A | A | T | A | T | C | A | A | T | A | C | C | G | T | C | T | G | C | G | G | A | G | G | T | A | T | T | C | A | T | A | T | A | A | T | G | G | T | T | T | A | C | T | T | T | A | T | A | T | G | T | A | A | G | A | C | T | T | T | A | G | G | A | A | T | A | A | A | G | C | C | C | T | A | C | G | T | A | T | A | T | A | G | G | A | A | C | A | C | C | C | C | T | G | A | A | G | A | T | G | G | A | A | G | T | A | A | T | C | T | A | G | G | A | T | T | A | T | T | A | T | C | C | C | T | C | T | T | A | G | G | A | A | A | C | A | A | A | C | C | C | T | C | A | G | A | G | A | A | T | T | C | G | T | C | A | G | A | A | A | A | T | C | C | T | C | T | A | G |  | : |  | 1 | 3 | 6 | 8 |
| M | T | 0 | 3 | 6 | 0 | 5 | 5 |  | : |  | A | T | A | A | T | A | T | C | A | A | T | A | C | C | G | T | C | T | G | C | G | G | A | G | G | T | A | T | T | C | A | T | A | T | A | A | T | G | G | T | T | T | A | C | T | T | T | A | T | A | T | G | T | A | A | G | A | C | T | T | T | A | G | G | A | A | T | A | A | A | G | C | C | C | T | A | C | G | T | A | T | A | T | A | G | G | A | A | C | A | C | C | C | C | T | G | A | A | G | A | T | G | G | A | A | G | T | A | A | T | C | T | A | G | G | A | T | T | A | T | T | A | T | C | C | C | T | C | T | T | A | G | G | A | A | A | C | A | A | A | C | C | C | T | C | A | G | A | G | A | A | T | T | C | G | T | C | A | G | A | A | A | A | T | C | C | T | C | T | A | G |  | : |  | 1 | 3 | 6 | 8 |
| M | T | 0 | 3 | 6 | 0 | 5 | 6 |  | : |  | A | T | A | A | T | A | T | C | A | A | T | A | C | C | G | T | C | T | G | C | G | G | A | G | G | T | A | T | T | C | A | T | A | T | A | A | T | G | G | T | T | T | A | C | T | T | T | A | T | A | C | G | T | A | A | G | A | C | T | T | T | A | G | G | A | A | T | A | A | A | G | C | C | C | T | A | C | G | T | A | T | A | T | A | G | G | A | A | C | A | C | C | C | C | T | G | A | A | G | A | A | G | G | A | A | G | C | A | A | T | C | T | A | G | G | A | T | T | A | C | T | G | T | C | C | C | T | C | T | T | A | G | G | A | A | A | C | A | A | A | C | C | C | T | C | A | G | A | G | A | G | T | T | C | G | T | C | A | G | A | A | A | A | C | C | C | T | C | T | A | G |  | : |  | 1 | 3 | 6 | 8 |
| M | T | 0 | 3 | 6 | 0 | 5 | 7 |  | : |  | A | T | A | A | T | A | T | C | A | A | T | A | C | C | G | T | C | T | G | C | G | G | A | G | G | T | A | T | T | C | A | T | A | T | A | A | T | G | G | T | T | T | A | C | T | T | T | A | T | A | T | G | T | A | A | G | A | C | T | T | T | A | G | G | A | A | T | A | A | A | G | C | C | C | T | A | C | G | T | A | T | A | T | A | G | G | A | A | C | A | C | C | C | C | T | G | A | A | G | A | A | G | G | A | A | G | C | A | A | T | C | T | A | G | G | A | T | T | A | C | T | G | T | C | C | C | T | C | T | T | A | G | G | A | A | A | C | A | A | A | C | C | C | T | C | A | G | A | G | A | G | T | T | C | G | T | C | A | G | A | A | A | A | C | C | C | T | C | T | A | G |  | : |  | 1 | 3 | 6 | 8 |
| K | X | 2 | 4 | 9 | 7 | 3 | 8 |  | : |  | A | T | A | A | T | A | T | C | A | A | T | A | C | C | G | T | C | T | G | C | G | G | A | G | G | T | A | T | T | C | A | T | A | T | A | A | T | G | G | T | T | T | A | C | T | T | T | A | T | A | T | G | T | A | A | G | A | C | T | T | T | A | G | G | A | A | T | A | A | A | G | C | C | C | T | A | C | G | T | A | T | A | T | A | G | G | A | A | C | A | C | C | C | C | T | G | A | A | G | A | A | G | G | A | A | G | C | A | A | T | C | T | A | G | G | A | T | T | A | C | T | A | T | C | C | C | T | C | T | T | A | G | G | A | A | A | C | A | A | A | C | C | C | T | C | A | G | A | G | A | G | T | T | C | G | T | C | A | G | A | A | A | A | C | C | C | T | C | T | A | G |  | : |  | 1 | 3 | 6 | 8 |
| K | X | 2 | 4 | 9 | 7 | 3 | 7 |  | : |  | A | T | A | A | T | A | T | C | A | A | T | A | C | C | G | T | C | T | G | C | G | G | A | G | G | T | A | T | T | C | A | T | A | T | A | A | T | G | G | T | T | T | A | C | T | T | T | A | T | A | T | G | T | A | A | G | A | C | T | T | T | A | G | G | A | A | T | A | A | A | G | C | C | C | T | A | C | G | T | A | T | A | T | A | G | G | A | A | C | A | C | C | C | C | T | G | A | A | G | A | A | G | G | A | A | G | C | A | A | T | C | T | A | G | G | A | T | T | A | C | T | A | T | C | C | C | T | C | T | T | A | G | G | A | A | A | C | A | A | A | C | C | C | T | C | A | G | A | G | A | G | T | T | C | G | T | C | A | G | A | A | A | A | C | C | C | T | C | T | A | G |  | : |  | 1 | 3 | 6 | 8 |
| K | X | 2 | 4 | 9 | 7 | 3 | 6 |  | : |  | A | T | A | A | T | A | T | C | A | A | T | A | C | C | G | T | C | T | G | C | G | G | A | G | G | T | A | T | T | C | A | T | A | T | A | A | T | G | G | T | T | T | A | C | T | T | T | A | T | A | T | G | T | A | A | G | A | C | T | T | T | A | G | G | A | A | T | A | A | A | G | C | C | C | T | A | C | G | T | A | T | A | T | A | G | G | A | A | C | A | C | C | C | C | T | G | A | A | G | A | A | G | G | A | A | G | C | A | A | T | C | T | A | G | G | A | T | T | A | C | T | A | T | C | C | C | T | C | T | T | A | G | G | A | A | A | C | A | A | A | C | C | C | T | C | A | G | A | G | A | G | T | T | C | G | T | C | A | G | A | A | A | A | C | C | C | T | C | T | A | G |  | : |  | 1 | 3 | 6 | 8 |
| K | X | 2 | 4 | 9 | 7 | 3 | 5 |  | : |  | A | T | A | A | T | A | T | C | A | A | T | A | C | C | G | T | C | T | G | C | G | G | A | G | G | T | A | T | T | C | A | T | A | T | A | A | T | G | G | T | T | T | A | C | T | T | T | A | T | A | T | G | T | A | A | G | A | C | T | T | T | A | G | G | A | A | T | A | A | A | G | C | C | C | T | A | C | G | T | A | T | A | T | A | G | G | A | A | C | A | C | A | C | C | T | G | A | A | G | A | A | G | G | A | A | G | C | A | A | T | C | A | A | G | G | A | T | T | A | C | T | G | T | C | C | C | T | C | T | T | A | G | G | A | A | A | C | A | A | A | C | C | C | T | C | A | G | A | G | A | G | T | T | C | G | T | C | A | G | A | A | A | A | C | C | C | T | C | T | A | G |  | : |  | 1 | 3 | 6 | 8 |
| M | F | 1 | 9 | 7 | 9 | 1 | 6 |  | : |  | A | T | A | A | T | A | T | C | A | A | T | A | C | C | G | T | C | T | G | C | G | G | A | G | G | T | A | T | T | C | A | T | A | T | A | A | T | G | G | T | T | T | A | C | T | T | T | A | T | A | T | G | T | A | A | G | A | C | T | T | T | A | G | G | A | A | T | A | A | A | G | C | C | C | T | A | C | G | T | A | T | A | T | A | G | G | A | A | C | A | C | C | C | C | T | G | A | A | G | A | A | G | G | A | A | G | C | A | A | T | C | T | A | G | G | A | T | T | A | C | T | G | T | C | C | C | T | C | T | T | A | G | G | A | A | A | C | A | A | A | C | C | C | T | C | A | G | A | G | A | G | T | T | C | G | T | C | A | G | A | A | A | A | C | C | C | T | C | T | A | G |  | : |  | 1 | 3 | 6 | 8 |
| K | T | 2 | 5 | 0 | 6 | 3 | 2 |  | : |  | A | T | A | A | T | A | T | C | A | A | T | A | C | C | G | T | C | T | G | C | G | G | A | G | G | T | A | T | T | C | A | T | A | T | A | A | T | G | G | T | T | T | A | C | T | T | T | A | T | A | T | G | T | A | A | G | A | C | T | T | T | A | G | G | A | A | T | A | A | A | G | C | C | C | T | A | C | G | T | A | T | A | T | A | G | G | A | A | C | A | C | C | C | C | T | G | A | A | G | A | T | G | G | A | A | G | T | A | A | T | C | T | A | G | G | A | T | T | A | T | T | A | T | C | C | C | T | C | T | T | A | G | G | A | A | A | C | A | A | A | C | C | C | T | C | A | G | A | G | A | A | T | T | C | G | T | C | A | G | A | A | A | A | T | C | C | T | C | T | A | G |  | : |  | 1 | 3 | 6 | 8 |
|  |  |  |  |  |  |  |  |  |  |  | A | T | A | A | T | A | T | C | A | A | T | A | C | C | G | T | C | T | G | C | G | G | A | G | G | T | A | T | T | C | A | T | A | T | A | A | T | G | G | T | T | T | A | C | T | T | T | A | T | A | t | G | T | A | A | G | A | C | T | T | T | A | G | G | A | A | T | A | A | A | G | C | C | C | T | A | C | G | T | A | T | A | T | A | G | G | A | A | C | A | C | c | C | C | T | G | A | A | G | A |  | G | G | A | A | G |  | A | A | T | C | t | A | G | G | A | T | T | A |  | T |  | T | C | C | C | T | C | T | T | A | G | G | A | A | A | C | A | A | A | C | C | C | T | C | A | G | A | G | A |  | T | T | C | G | T | C | A | G | A | A | A | A |  | C | C | T | C | T | A | G |  |  |  |  |  |  |  |

|  |  |  |  |  |  |  |  |  |  |  |  |  |  |  |  |  |  |  |  |  |  |  |  |  |  |  |  |  |  |  |  |  |  |  |  |  |  |  |  |  |  |  |  |  |  |  |  |  |  |  |  |  |  |  |  |  |  |  |  |  |  |  |  |  |  |  |  |  |  |  |  |  |  |  |  |  |  |  |  |  |  |  |  |  |  |  |  |  |  |  |  |  |  |  |  |  |  |  |  |  |  |  |  |  |  |  |  |  |  |  |  |  |  |  |  |  |  |  |  |  |  |  |  |  |  |  |  |  |  |  |  |  |  |  |  |  |  |  |  |  |  |  |  |  |  |  |  |  |  |  |  |  |  |  |  |  |  |  |  |  |  |  |  |  |  |  |  |  |  |  |  |  |  |  |  |  |  |  |  |  |  |  |  |  |  |  |  |  |
| --- | --- | --- | --- | --- | --- | --- | --- | --- | --- | --- | --- | --- | --- | --- | --- | --- | --- | --- | --- | --- | --- | --- | --- | --- | --- | --- | --- | --- | --- | --- | --- | --- | --- | --- | --- | --- | --- | --- | --- | --- | --- | --- | --- | --- | --- | --- | --- | --- | --- | --- | --- | --- | --- | --- | --- | --- | --- | --- | --- | --- | --- | --- | --- | --- | --- | --- | --- | --- | --- | --- | --- | --- | --- | --- | --- | --- | --- | --- | --- | --- | --- | --- | --- | --- | --- | --- | --- | --- | --- | --- | --- | --- | --- | --- | --- | --- | --- | --- | --- | --- | --- | --- | --- | --- | --- | --- | --- | --- | --- | --- | --- | --- | --- | --- | --- | --- | --- | --- | --- | --- | --- | --- | --- | --- | --- | --- | --- | --- | --- | --- | --- | --- | --- | --- | --- | --- | --- | --- | --- | --- | --- | --- | --- | --- | --- | --- | --- | --- | --- | --- | --- | --- | --- | --- | --- | --- | --- | --- | --- | --- | --- | --- | --- | --- | --- | --- | --- | --- | --- | --- | --- | --- | --- | --- | --- | --- | --- | --- | --- | --- | --- | --- | --- | --- | --- | --- | --- | --- |
|  |  |  |  |  |  |  |  |  |  |  |  |  |  |  |  |  |  |  |  |  |  |  |  |  |  |  |  |  |  |  |  |  |  |  |  |  |  |  |  |  |  |  |  |  |  |  |  |  |  |  |  |  |  |  |  |  |  |  |  |  |  |  |  |  |  |  |  |  |  |  |  |  |  |  |  |  |  |  |  |  |  |  |  |  |  |  |  |  |  |  |  |  |  |  |  |  |  |  |  |  |  |  |  |  |  |  |  |  |  |  |  |  |  |  |  |  |  |  |  |  |  |  |  |  |  |  |  |  |  |  |  |  |  |  |  |  |  |  |  |  |  |  |  |  |  |  |  |  |  |  |  |  |  |  |  |  |  |  |  |  |  |  |  |  |  |  |  |  |  |  |  |  |  |  |  |  |  |  |  |  |  |  |  |  |  |  |  |  |
|  |  |  |  |  |  |  |  |  |  |  |  | \* |  |  |  |  |  |  | 1 | 3 | 8 | 0 |  |  |  |  |  |  |  |  |  | \* |  |  |  |  |  |  | 1 | 4 | 0 | 0 |  |  |  |  |  |  |  |  |  | \* |  |  |  |  |  |  | 1 | 4 | 2 | 0 |  |  |  |  |  |  |  |  |  | \* |  |  |  |  |  |  | 1 | 4 | 4 | 0 |  |  |  |  |  |  |  |  |  | \* |  |  |  |  |  |  | 1 | 4 | 6 | 0 |  |  |  |  |  |  |  |  |  | \* |  |  |  |  |  |  | 1 | 4 | 8 | 0 |  |  |  |  |  |  |  |  |  | \* |  |  |  |  |  |  | 1 | 5 | 0 | 0 |  |  |  |  |  |  |  |  |  | \* |  |  |  |  |  |  | 1 | 5 | 2 | 0 |  |  |  |  |  |  |  |  |  | \* |  |  |  |  |  |  | 1 | 5 | 4 |  |  |  |  |  |  |  |
| M | N | 9 | 5 | 6 | 5 | 2 | 0 |  | : |  | A | G | A | A | A | A | A | G | A | T | A | G | A | T | A | A | T | C | T | T | A | G | T | G | A | T | A | A | G | A | T | T | A | G | A | G | A | T | C | T | T | A | G | A | T | C | T | A | G | T | A | C | C | T | C | A | G | G | T | A | C | A | G | A | C | C | T | C | A | A | A | A | T | C | C | A | G | T | T | A | G | A | C | A | A | A | C | T | T | A | G | A | G | A | T | A | A | G | A | T | T | G | A | T | G | A | T | C | T | G | G | A | A | A | G | A | A | A | A | G | T | T | T | C | T | T | C | T | C | T | T | A | A | C | A | C | A | G | A | A | G | A | A | A | T | C | T | T | G | A | A | G | T | T | A | C | T | A | A | A | A | C | G | T | C | T | A | G | A | C | G |  | : |  | 1 | 5 | 3 | 9 |
| M | T | 0 | 1 | 2 | 7 | 3 | 2 |  | : |  | A | G | A | A | A | A | A | G | A | T | A | G | A | T | A | A | T | C | T | T | A | G | C | G | A | T | A | A | G | A | T | T | A | G | A | G | A | T | C | T | T | A | G | A | T | C | T | A | G | T | A | C | C | T | C | A | G | G | T | A | C | A | G | A | C | C | T | C | A | A | A | A | T | C | C | A | G | T | T | A | G | A | C | A | A | A | C | T | T | A | G | A | G | A | T | A | A | G | A | T | T | G | A | T | G | A | T | C | T | G | G | A | A | A | G | A | A | A | A | G | T | T | T | C | T | T | C | T | C | T | T | A | A | C | A | C | A | G | A | A | G | A | A | A | T | C | T | T | G | A | A | G | T | T | A | C | T | A | A | A | A | C | G | T | C | T | A | G | A | C | G |  | : |  | 1 | 5 | 3 | 9 |
| M | T | 0 | 1 | 2 | 7 | 3 | 4 |  | : |  | A | G | A | A | A | A | A | G | A | T | A | G | A | T | A | A | T | C | T | T | A | G | C | G | A | T | A | A | G | A | T | T | A | G | A | G | A | T | C | T | T | A | G | A | T | C | T | A | G | T | A | C | C | T | C | A | G | G | T | A | C | A | G | A | C | C | T | C | A | A | A | A | T | C | C | A | G | A | T | A | G | A | C | A | A | A | C | T | T | A | G | A | G | A | T | A | A | G | A | T | T | G | A | T | G | A | T | C | T | G | G | A | A | A | G | A | A | A | A | G | T | T | T | C | T | T | C | T | C | T | T | A | A | C | A | C | A | G | A | A | G | A | A | A | T | C | T | T | G | A | A | G | T | T | A | C | T | A | A | A | A | C | G | T | C | T | A | G | A | C | G |  | : |  | 1 | 5 | 3 | 9 |
| M | T | 0 | 2 | 7 | 0 | 0 | 6 |  | : |  | A | G | A | A | A | A | A | G | A | T | A | G | A | T | A | A | T | C | T | T | A | G | C | G | A | T | A | A | G | A | T | T | A | G | A | G | A | T | C | T | T | A | G | A | T | C | T | A | G | T | A | C | C | T | C | A | G | G | T | A | C | A | G | A | C | C | T | C | A | A | A | A | T | C | C | A | G | T | T | A | G | A | C | A | A | A | C | T | T | A | G | A | G | A | T | A | A | G | A | T | T | G | A | T | G | A | T | C | T | G | G | A | A | A | G | A | A | A | A | G | T | T | T | C | T | T | C | T | C | T | T | A | A | C | A | C | A | G | A | A | G | A | A | A | T | C | T | T | G | A | A | G | T | T | A | C | T | A | A | A | A | C | G | T | C | T | A | G | A | C | G |  | : |  | 1 | 5 | 3 | 9 |
| M | T | 0 | 2 | 7 | 0 | 0 | 7 |  | : |  | A | G | A | A | A | A | A | G | A | T | A | G | A | T | A | A | T | C | T | T | A | G | T | G | A | T | A | A | G | A | T | T | A | G | A | G | A | T | C | T | T | A | G | A | T | C | T | A | G | T | A | C | C | T | C | A | G | G | T | A | C | A | G | A | C | C | T | C | A | A | A | A | T | C | C | A | G | T | T | A | G | A | C | A | A | A | C | T | T | A | G | A | G | A | T | A | A | G | A | T | T | G | A | T | G | A | T | C | T | G | G | A | A | A | G | A | A | A | A | G | T | T | T | C | T | T | C | T | C | T | T | A | A | C | A | C | A | G | A | A | G | A | A | A | T | C | T | T | G | A | A | G | T | T | A | C | T | A | A | A | A | C | G | T | C | T | A | G | A | C | G |  | : |  | 1 | 5 | 3 | 9 |
| M | T | 0 | 3 | 6 | 0 | 5 | 3 |  | : |  | A | G | A | A | A | A | A | G | A | T | A | G | A | T | A | A | T | C | T | T | A | G | T | G | A | T | A | A | G | A | T | T | A | G | A | G | A | T | C | T | T | A | G | A | T | C | T | A | G | T | A | C | C | T | C | A | G | G | T | A | C | A | G | A | C | C | T | C | A | A | A | A | T | C | C | A | G | T | T | A | G | A | C | A | A | A | C | T | T | A | G | A | G | A | T | A | A | G | A | T | T | G | A | T | G | A | T | C | T | G | G | A | A | A | G | A | A | A | A | G | T | T | T | C | T | T | C | T | C | T | T | A | A | C | A | C | A | G | A | A | G | A | A | A | T | C | T | T | G | A | A | G | T | T | A | C | T | A | A | A | A | C | G | T | C | T | A | G | A | C | G |  | : |  | 1 | 5 | 3 | 9 |
| M | T | 0 | 3 | 6 | 0 | 5 | 4 |  | : |  | A | G | A | A | A | A | A | G | A | T | A | G | A | T | A | A | T | C | T | T | A | G | T | G | A | T | A | A | G | A | T | T | A | G | A | G | A | T | C | T | T | A | G | A | T | C | T | A | G | T | A | C | C | T | C | A | G | G | T | A | C | A | G | A | C | C | T | C | A | A | A | A | T | C | C | A | G | T | T | A | G | A | C | A | A | A | C | T | T | A | G | A | G | A | T | A | A | G | A | T | T | G | A | T | G | A | T | C | T | G | G | A | A | A | G | A | A | A | A | G | T | T | T | C | T | T | C | T | C | T | T | A | A | C | A | C | A | G | A | A | G | A | A | A | T | C | T | T | G | A | A | G | T | T | A | C | T | A | A | A | A | C | G | T | C | T | A | G | A | C | G |  | : |  | 1 | 5 | 3 | 9 |
| M | T | 0 | 3 | 6 | 0 | 5 | 5 |  | : |  | A | G | A | A | A | A | A | G | A | T | A | G | A | T | A | A | T | C | T | T | A | G | T | G | A | T | A | A | G | A | T | T | A | G | A | G | A | T | C | T | T | A | G | A | T | C | T | A | G | T | A | C | C | T | C | A | G | G | T | A | C | A | G | A | C | C | T | C | A | A | A | A | T | C | C | A | G | T | T | A | G | A | C | A | A | A | C | T | T | A | G | A | G | A | T | A | A | G | A | T | T | G | A | T | G | A | T | C | T | G | G | A | A | A | G | A | A | A | A | G | T | T | T | C | T | T | C | T | C | T | T | A | A | C | A | C | A | G | A | A | G | A | A | A | T | C | T | T | G | A | A | G | T | T | A | C | T | A | A | A | A | C | G | T | C | T | A | G | A | C | G |  | : |  | 1 | 5 | 3 | 9 |
| M | T | 0 | 3 | 6 | 0 | 5 | 6 |  | : |  | A | G | A | A | A | A | A | G | A | C | A | G | A | T | A | A | T | C | T | T | A | G | C | G | A | T | A | A | G | A | T | T | A | G | A | G | A | T | C | T | T | A | G | A | T | C | T | A | G | T | A | C | C | T | C | A | G | G | T | A | C | A | G | A | C | C | T | C | A | A | A | A | T | C | C | A | G | T | T | A | G | A | C | A | A | A | C | T | T | A | G | A | G | A | T | A | A | G | A | T | T | G | A | T | G | A | T | C | T | G | G | A | A | A | G | A | A | A | A | G | T | T | T | C | T | T | C | T | C | T | T | A | A | C | A | C | A | G | A | A | G | A | A | A | T | C | T | T | G | A | A | G | T | T | A | C | T | A | A | A | G | C | G | T | C | T | A | G | A | C | G |  | : |  | 1 | 5 | 3 | 9 |
| M | T | 0 | 3 | 6 | 0 | 5 | 7 |  | : |  | A | G | A | A | A | A | A | G | A | T | A | G | A | T | A | A | T | C | T | T | A | G | C | G | A | T | A | A | G | A | T | T | A | G | A | G | A | T | C | T | T | A | G | A | T | C | T | A | G | T | A | C | C | T | C | A | G | G | T | A | C | A | G | A | C | C | T | C | A | A | A | A | T | C | C | A | G | T | T | A | G | A | C | A | A | A | C | T | T | A | G | A | G | A | T | A | A | G | A | T | T | G | A | T | G | A | T | C | T | G | G | A | A | A | G | A | A | A | A | G | T | T | T | C | T | T | C | T | C | T | T | A | A | C | A | C | A | G | A | A | G | A | A | A | T | C | T | T | G | A | A | G | T | T | A | C | T | A | A | A | A | C | G | T | C | T | A | G | A | C | G |  | : |  | 1 | 5 | 3 | 9 |
| K | X | 2 | 4 | 9 | 7 | 3 | 8 |  | : |  | A | G | A | A | A | A | A | G | A | T | A | G | A | T | A | A | T | C | T | T | A | A | C | G | A | T | A | A | G | A | T | T | A | G | A | G | A | T | C | T | T | A | G | A | T | C | T | A | G | T | A | C | C | T | C | A | G | G | T | A | C | A | G | A | C | C | T | C | A | A | A | A | T | C | C | A | G | T | T | A | G | A | C | A | A | A | C | T | C | A | G | A | G | A | T | A | A | G | A | T | T | G | A | T | G | A | T | C | T | G | G | A | A | A | G | A | A | A | A | G | T | T | T | C | T | T | C | T | C | T | T | A | A | C | A | C | A | G | A | A | G | A | A | A | T | C | T | T | G | A | A | G | T | T | A | C | T | A | A | A | A | C | G | T | C | T | A | G | A | C | G |  | : |  | 1 | 5 | 3 | 9 |
| K | X | 2 | 4 | 9 | 7 | 3 | 7 |  | : |  | A | G | A | A | A | A | A | G | A | T | A | G | A | T | A | A | T | C | T | T | A | A | C | G | A | T | A | A | G | A | T | T | A | G | A | G | A | T | C | T | T | A | G | A | T | C | T | A | G | T | A | C | C | T | C | A | G | G | T | A | C | A | G | A | C | C | T | C | A | A | A | A | T | C | C | A | G | T | T | A | G | A | C | A | A | A | C | T | C | A | G | A | G | A | T | A | A | G | A | T | T | G | A | T | G | A | T | C | T | G | G | A | A | A | G | A | A | A | A | G | T | T | T | C | T | T | C | T | C | T | T | A | A | C | A | C | A | G | A | A | G | A | A | A | T | C | T | T | G | A | A | G | T | T | A | C | T | A | A | A | A | C | G | T | C | T | A | G | A | C | G |  | : |  | 1 | 5 | 3 | 9 |
| K | X | 2 | 4 | 9 | 7 | 3 | 6 |  | : |  | A | G | A | A | A | A | A | G | A | T | A | G | A | T | A | A | T | C | T | T | A | A | C | G | A | T | A | A | G | A | T | T | A | G | A | G | A | T | C | T | T | A | G | A | T | C | T | A | G | T | A | C | C | T | C | A | G | G | T | A | C | A | G | A | C | C | T | C | A | A | A | A | T | C | C | A | G | T | T | A | G | A | C | A | A | A | C | T | C | A | G | A | G | A | T | A | A | G | A | T | T | G | A | T | G | A | T | C | T | G | G | A | A | A | G | A | A | A | A | G | T | T | T | C | T | T | C | T | C | T | T | A | A | C | A | C | A | G | A | A | G | A | A | A | T | C | T | T | G | A | A | G | T | T | A | C | T | A | A | A | A | C | G | T | C | T | A | G | A | C | G |  | : |  | 1 | 5 | 3 | 9 |
| K | X | 2 | 4 | 9 | 7 | 3 | 5 |  | : |  | A | G | A | A | A | A | A | G | A | T | A | G | A | T | A | A | T | C | T | T | A | G | C | G | A | T | A | A | G | A | T | T | A | G | A | G | A | T | C | T | T | A | G | A | T | C | T | A | G | T | A | C | C | T | C | A | G | G | T | A | C | A | G | A | C | C | T | C | A | A | A | A | T | C | C | A | G | T | T | A | G | A | C | A | A | A | C | T | T | A | G | A | G | A | T | A | A | G | A | T | T | G | A | T | G | A | T | C | T | G | G | A | A | A | G | A | A | A | A | G | T | T | T | C | T | T | C | T | C | T | T | A | A | C | A | C | A | G | A | A | G | A | A | A | T | C | T | T | G | A | A | G | T | T | A | C | T | A | A | A | A | C | G | T | C | T | A | G | A | C | G |  | : |  | 1 | 5 | 3 | 9 |
| M | F | 1 | 9 | 7 | 9 | 1 | 6 |  | : |  | A | G | A | A | A | A | A | G | A | T | A | G | A | T | A | A | T | C | T | T | A | G | C | G | A | T | A | A | G | A | T | T | A | G | A | G | A | T | C | T | T | A | G | A | T | C | T | A | G | T | A | C | C | T | C | A | G | G | T | A | C | A | G | A | C | C | T | C | A | A | A | A | T | C | C | A | G | T | T | A | G | A | C | A | A | A | C | T | T | A | G | A | G | A | T | A | A | G | A | T | T | G | A | T | G | A | T | C | T | G | G | A | A | A | G | A | A | A | A | G | T | T | T | C | T | T | C | T | C | T | T | A | A | C | A | C | A | G | A | A | G | A | A | A | T | C | T | T | G | A | A | G | T | T | A | C | T | A | A | A | A | C | G | T | C | T | A | G | A | C | G |  | : |  | 1 | 5 | 3 | 9 |
| K | T | 2 | 5 | 0 | 6 | 3 | 2 |  | : |  | A | G | A | A | A | A | A | G | A | T | A | G | A | T | A | A | T | C | T | T | A | G | C | G | A | T | A | A | G | A | T | T | A | G | A | G | A | T | C | T | T | A | G | A | T | C | T | A | G | T | A | C | C | T | C | A | G | G | T | A | C | A | G | A | C | C | T | C | A | A | A | A | T | C | C | A | G | T | T | A | G | A | C | A | A | A | C | T | T | A | G | A | G | A | T | A | A | G | A | T | T | G | A | T | G | A | T | C | T | G | G | A | A | A | G | A | A | A | A | G | T | T | T | C | T | T | C | T | C | T | T | A | A | C | A | C | A | G | A | A | G | A | A | A | T | C | T | T | G | A | A | G | T | T | A | C | T | A | A | A | A | C | G | T | C | T | A | G | A | C | G |  | : |  | 1 | 5 | 3 | 9 |
|  |  |  |  |  |  |  |  |  |  |  | A | G | A | A | A | A | A | G | A | t | A | G | A | T | A | A | T | C | T | T | A | g |  | G | A | T | A | A | G | A | T | T | A | G | A | G | A | T | C | T | T | A | G | A | T | C | T | A | G | T | A | C | C | T | C | A | G | G | T | A | C | A | G | A | C | C | T | C | A | A | A | A | T | C | C | A | G | t | T | A | G | A | C | A | A | A | C | T | t | A | G | A | G | A | T | A | A | G | A | T | T | G | A | T | G | A | T | C | T | G | G | A | A | A | G | A | A | A | A | G | T | T | T | C | T | T | C | T | C | T | T | A | A | C | A | C | A | G | A | A | G | A | A | A | T | C | T | T | G | A | A | G | T | T | A | C | T | A | A | A | a | C | G | T | C | T | A | G | A | C | G |  |  |  |  |  |  |  |

|  |  |  |  |  |  |  |  |  |  |  |  |  |  |  |  |  |  |  |  |  |  |  |  |  |  |  |  |  |  |  |  |  |  |  |  |  |  |  |  |  |  |  |  |  |  |  |  |  |  |  |  |  |  |  |  |  |  |  |  |  |  |  |  |  |  |  |  |  |  |  |  |  |  |  |  |  |  |  |  |  |  |  |  |  |  |  |  |  |  |  |  |  |  |  |  |  |  |  |  |  |  |  |  |  |  |  |  |  |  |  |  |  |  |  |  |  |  |  |  |  |  |  |  |  |  |  |  |  |  |  |  |  |  |  |  |  |  |  |  |  |  |  |  |  |  |  |  |  |  |  |  |  |  |  |  |  |  |  |  |  |  |  |  |  |  |  |  |  |  |  |  |  |  |  |  |  |  |  |  |  |  |  |  |  |  |  |  |  |
| --- | --- | --- | --- | --- | --- | --- | --- | --- | --- | --- | --- | --- | --- | --- | --- | --- | --- | --- | --- | --- | --- | --- | --- | --- | --- | --- | --- | --- | --- | --- | --- | --- | --- | --- | --- | --- | --- | --- | --- | --- | --- | --- | --- | --- | --- | --- | --- | --- | --- | --- | --- | --- | --- | --- | --- | --- | --- | --- | --- | --- | --- | --- | --- | --- | --- | --- | --- | --- | --- | --- | --- | --- | --- | --- | --- | --- | --- | --- | --- | --- | --- | --- | --- | --- | --- | --- | --- | --- | --- | --- | --- | --- | --- | --- | --- | --- | --- | --- | --- | --- | --- | --- | --- | --- | --- | --- | --- | --- | --- | --- | --- | --- | --- | --- | --- | --- | --- | --- | --- | --- | --- | --- | --- | --- | --- | --- | --- | --- | --- | --- | --- | --- | --- | --- | --- | --- | --- | --- | --- | --- | --- | --- | --- | --- | --- | --- | --- | --- | --- | --- | --- | --- | --- | --- | --- | --- | --- | --- | --- | --- | --- | --- | --- | --- | --- | --- | --- | --- | --- | --- | --- | --- | --- | --- | --- | --- | --- | --- | --- | --- | --- | --- | --- | --- | --- | --- | --- | --- |
|  |  |  |  |  |  |  |  |  |  |  |  |  |  |  |  |  |  |  |  |  |  |  |  |  |  |  |  |  |  |  |  |  |  |  |  |  |  |  |  |  |  |  |  |  |  |  |  |  |  |  |  |  |  |  |  |  |  |  |  |  |  |  |  |  |  |  |  |  |  |  |  |  |  |  |  |  |  |  |  |  |  |  |  |  |  |  |  |  |  |  |  |  |  |  |  |  |  |  |  |  |  |  |  |  |  |  |  |  |  |  |  |  |  |  |  |  |  |  |  |  |  |  |  |  |  |  |  |  |  |  |  |  |  |  |  |  |  |  |  |  |  |  |  |  |  |  |  |  |  |  |  |  |  |  |  |  |  |  |  |  |  |  |  |  |  |  |  |  |  |  |  |  |  |  |  |  |  |  |  |  |  |  |  |  |  |  |  |  |
|  |  |  |  |  |  |  |  |  |  |  | 0 |  |  |  |  |  |  |  |  |  | \* |  |  |  |  |  |  | 1 | 5 | 6 | 0 |  |  |  |  |  |  |  |  |  | \* |  |  |  |  |  |  | 1 | 5 | 8 | 0 |  |  |  |  |  |  |  |  |  | \* |  |  |  |  |  |  | 1 | 6 | 0 | 0 |  |  |  |  |  |  |  |  |  | \* |  |  |  |  |  |  | 1 | 6 | 2 | 0 |  |  |  |  |  |  |  |  |  | \* |  |  |  |  |  |  | 1 | 6 | 4 | 0 |  |  |  |  |  |  |  |  |  | \* |  |  |  |  |  |  | 1 | 6 | 6 | 0 |  |  |  |  |  |  |  |  |  | \* |  |  |  |  |  |  | 1 | 6 | 8 | 0 |  |  |  |  |  |  |  |  |  | \* |  |  |  |  |  |  | 1 | 7 | 0 | 0 |  |  |  |  |  |  |  |  |  | \* |  |  |  |  |  |  |  |
| M | N | 9 | 5 | 6 | 5 | 2 | 0 |  | : |  | A | C | A | A | A | G | T | T | T | A | G | A | T | G | G | C | T | A | C | G | G | T | A | A | C | T | C | A | A | A | A | G | A | C | C | G | A | A | A | G | G | A | T | C | T | A | T | G | A | T | G | A | A | A | T | C | C | T | C | A | A | A | A | T | G | G | A | A | G | A | G | A | G | A | T | T | T | C | A | G | A | A | G | C | T | C | G | A | A | C | A | A | G | A | G | C | A | A | C | A | A | G | A | T | C | A | C | T | T | C | T | C | A | C | A | G | A | A | G | A | T | T | G | A | T | C | A | G | G | C | A | T | C | T | G | C | T | A | C | C | C | T | A | A | T | G | G | C | A | G | A | A | A | T | T | A | A | A | A | G | A | A | T | A | C | A | G | T | C | A | A |  | : |  | 1 | 7 | 1 | 0 |
| M | T | 0 | 1 | 2 | 7 | 3 | 2 |  | : |  | A | C | A | A | A | G | T | T | T | A | G | A | T | G | G | C | T | A | C | G | G | T | A | A | C | T | C | A | A | A | A | G | A | C | C | G | A | A | A | G | G | G | T | C | T | A | T | G | A | T | G | A | A | A | T | C | C | T | C | A | A | A | A | T | G | G | A | A | G | A | G | A | G | A | T | T | T | C | A | G | A | A | G | C | T | C | G | A | A | C | A | A | G | A | G | C | A | A | C | A | A | G | A | C | C | A | C | T | T | C | T | C | A | C | A | G | A | A | G | A | T | T | G | A | T | C | A | G | G | C | A | T | C | T | G | C | T | A | C | C | C | T | A | A | T | G | A | C | A | G | A | A | A | T | C | A | A | G | A | G | A | A | T | A | C | A | G | T | C | A | A |  | : |  | 1 | 7 | 1 | 0 |
| M | T | 0 | 1 | 2 | 7 | 3 | 4 |  | : |  | A | C | A | A | A | G | T | T | T | A | G | A | T | G | G | C | G | A | C | G | G | T | A | A | C | T | C | A | A | A | A | G | A | C | C | G | A | A | A | G | G | A | T | C | T | A | T | G | A | T | G | A | A | A | T | C | C | T | C | A | A | A | A | T | G | G | A | A | G | A | G | A | G | A | T | T | T | C | A | G | A | A | G | C | T | C | G | A | A | C | A | A | G | A | G | C | A | A | C | A | A | G | A | T | C | A | C | T | T | C | T | C | A | C | A | A | A | A | G | A | T | T | G | A | T | C | A | G | G | C | A | T | C | T | G | C | T | A | C | C | C | T | A | A | T | G | G | C | A | G | A | A | A | T | C | A | A | G | A | G | A | A | T | A | C | A | G | T | C | A | A |  | : |  | 1 | 7 | 1 | 0 |
| M | T | 0 | 2 | 7 | 0 | 0 | 6 |  | : |  | A | C | A | A | A | G | T | T | T | A | G | A | T | G | G | C | T | A | C | G | G | T | A | A | C | T | C | A | A | A | A | G | A | C | C | G | A | A | A | G | G | A | T | C | T | A | T | G | A | T | G | A | A | A | T | C | C | T | C | A | A | A | A | T | G | G | A | A | G | A | G | A | G | A | T | T | T | C | A | G | A | A | G | C | T | C | G | A | A | C | A | A | G | A | G | C | A | A | C | A | A | G | A | T | C | A | C | T | T | C | T | C | A | C | A | G | A | A | G | A | T | T | G | A | T | C | A | G | G | C | A | T | C | T | G | C | T | A | C | C | C | T | A | A | T | G | G | C | A | G | A | A | A | T | C | A | A | G | A | G | A | A | T | A | C | A | G | T | C | A | A |  | : |  | 1 | 7 | 1 | 0 |
| M | T | 0 | 2 | 7 | 0 | 0 | 7 |  | : |  | A | C | A | A | A | G | T | T | T | A | G | A | T | G | G | C | T | A | C | G | G | T | A | A | C | T | C | A | A | A | A | G | A | C | C | G | A | A | A | G | G | A | T | C | T | A | T | G | A | T | G | A | A | A | T | C | C | T | C | A | A | A | A | T | G | G | A | A | G | A | G | A | G | A | T | T | T | C | A | G | A | A | G | C | T | C | G | A | A | C | A | A | G | A | G | C | A | A | C | A | A | G | A | T | C | A | C | T | T | C | T | C | A | C | A | G | A | A | G | A | T | T | G | A | T | C | A | G | G | C | A | T | C | T | G | C | T | A | C | C | C | T | A | A | T | G | G | C | A | G | A | A | A | T | C | A | A | A | A | G | A | A | T | A | C | A | G | T | C | A | A |  | : |  | 1 | 7 | 1 | 0 |
| M | T | 0 | 3 | 6 | 0 | 5 | 3 |  | : |  | A | C | A | A | A | G | T | T | T | A | G | A | T | G | G | C | T | A | C | G | G | T | A | A | C | T | C | A | A | A | A | G | A | C | C | G | A | A | A | G | G | A | T | C | T | A | T | G | A | T | G | A | A | A | T | C | C | T | C | A | A | A | A | T | G | G | A | A | G | A | G | A | G | A | T | T | T | C | A | A | A | A | G | C | T | C | G | A | A | C | A | A | G | A | G | C | A | A | C | A | A | G | A | T | C | A | C | T | T | C | T | C | A | C | A | G | A | A | G | A | T | T | G | A | T | C | A | G | G | C | A | T | C | T | G | C | T | A | C | C | C | T | A | A | T | G | G | C | A | G | A | A | A | T | C | A | A | A | A | G | A | A | T | A | C | A | G | T | C | A | A |  | : |  | 1 | 7 | 1 | 0 |
| M | T | 0 | 3 | 6 | 0 | 5 | 4 |  | : |  | A | C | A | A | A | G | T | T | T | A | G | A | T | G | G | C | T | A | C | G | G | T | A | A | C | T | C | A | A | A | A | G | A | C | C | G | A | A | A | G | G | A | T | C | T | A | T | G | A | T | G | A | A | A | T | C | C | T | C | A | A | A | A | T | G | G | A | A | G | A | G | A | G | A | T | T | T | C | A | A | A | A | G | C | T | C | G | A | A | C | A | A | G | A | G | C | A | A | C | A | A | G | A | T | C | A | C | T | T | C | T | C | A | C | A | G | A | A | G | A | T | T | G | A | T | C | A | G | G | C | A | T | C | T | G | C | T | A | C | C | C | T | A | A | T | G | G | C | A | G | A | A | A | T | C | A | A | A | A | G | A | A | T | A | C | A | G | T | C | A | A |  | : |  | 1 | 7 | 1 | 0 |
| M | T | 0 | 3 | 6 | 0 | 5 | 5 |  | : |  | A | C | A | A | A | G | T | T | T | A | G | A | T | G | G | C | T | A | C | G | G | T | A | A | C | T | C | A | A | A | A | G | A | C | C | G | A | A | A | G | G | A | T | C | T | A | T | G | A | T | G | A | A | A | T | C | C | T | C | A | A | A | A | T | G | G | A | A | G | A | G | A | G | A | T | T | T | C | A | G | A | A | G | C | T | C | G | A | A | C | A | A | G | A | G | C | A | A | C | A | A | G | A | T | C | A | C | T | T | C | T | C | A | C | A | G | A | A | G | A | T | T | G | A | T | C | A | G | G | C | A | T | C | T | G | C | T | A | C | C | C | T | A | A | T | G | G | C | A | G | A | A | A | T | C | A | A | G | A | G | A | A | T | A | C | A | G | T | C | A | A |  | : |  | 1 | 7 | 1 | 0 |
| M | T | 0 | 3 | 6 | 0 | 5 | 6 |  | : |  | A | C | A | A | A | G | T | T | T | A | G | A | T | G | G | C | T | A | C | G | G | T | A | A | C | T | C | A | A | A | A | G | A | C | C | G | A | A | A | G | G | A | T | C | T | A | T | G | A | T | G | A | A | A | T | C | C | T | C | A | A | A | A | T | G | G | A | A | G | A | G | A | G | A | T | T | T | C | A | G | A | A | G | C | T | C | G | A | A | C | A | A | G | A | G | C | A | A | C | A | A | G | A | T | C | A | C | T | T | C | T | C | A | C | A | G | A | A | G | A | T | T | G | A | T | C | A | G | G | C | A | G | C | T | G | C | T | A | C | C | C | T | A | A | T | G | G | C | A | G | A | A | A | T | C | A | A | G | A | G | A | A | T | A | C | A | G | T | C | A | A |  | : |  | 1 | 7 | 1 | 0 |
| M | T | 0 | 3 | 6 | 0 | 5 | 7 |  | : |  | A | C | A | A | A | G | T | T | T | A | G | A | T | G | G | C | T | A | C | G | G | T | A | A | C | T | C | A | A | A | A | G | A | C | C | G | A | A | A | G | G | A | T | C | T | A | T | G | A | T | G | A | A | A | T | C | C | T | C | A | A | A | A | T | G | G | A | A | G | A | G | A | G | A | T | T | T | C | A | G | A | A | G | C | T | C | G | A | A | C | A | A | G | A | G | C | A | A | C | A | A | G | A | T | C | A | C | T | T | C | T | C | A | C | A | G | A | A | G | A | T | T | G | A | T | C | A | G | G | C | A | G | C | T | G | C | T | A | C | C | C | T | A | A | T | G | G | C | A | G | A | A | A | T | C | A | A | G | A | G | A | A | T | A | C | A | G | T | C | A | A |  | : |  | 1 | 7 | 1 | 0 |
| K | X | 2 | 4 | 9 | 7 | 3 | 8 |  | : |  | A | C | A | A | A | G | T | T | T | A | G | A | T | G | G | C | T | A | C | G | G | T | A | A | C | T | C | A | A | A | A | G | A | C | C | G | A | A | A | G | G | A | T | C | T | A | T | G | A | T | G | A | A | A | T | C | C | T | C | A | A | A | A | T | G | G | A | A | G | A | G | A | G | A | T | T | T | C | A | G | A | A | G | C | T | C | G | A | A | C | A | A | G | A | G | C | A | A | C | A | A | G | A | T | C | A | C | T | T | C | T | C | A | C | A | G | A | A | G | A | T | T | G | A | T | C | A | G | G | C | A | T | C | T | G | C | T | A | C | C | C | T | A | A | T | G | G | C | A | G | A | A | A | T | C | A | A | G | A | G | A | A | T | A | C | A | G | T | C | A | A |  | : |  | 1 | 7 | 1 | 0 |
| K | X | 2 | 4 | 9 | 7 | 3 | 7 |  | : |  | A | C | A | A | A | G | T | T | T | A | G | A | T | G | G | C | T | A | C | G | G | T | A | A | C | T | C | A | A | A | A | G | A | C | C | G | A | A | A | G | G | A | T | C | T | A | T | G | A | T | G | A | A | A | T | C | C | T | C | A | A | A | A | T | G | G | A | A | G | A | G | A | G | A | T | T | T | C | A | G | A | A | G | C | T | C | G | A | A | C | A | A | G | A | G | C | A | A | C | A | A | G | A | T | C | A | C | T | T | C | T | C | A | C | A | G | A | A | G | A | T | T | G | A | T | C | A | G | G | C | A | T | C | T | G | C | T | A | C | C | C | T | A | A | T | G | G | C | A | G | A | A | A | T | C | A | A | G | A | G | A | A | T | A | C | A | G | T | C | A | A |  | : |  | 1 | 7 | 1 | 0 |
| K | X | 2 | 4 | 9 | 7 | 3 | 6 |  | : |  | A | C | A | A | A | G | T | T | T | A | G | A | T | G | G | C | T | A | C | G | G | T | A | A | C | T | C | A | A | A | A | G | A | C | C | G | A | A | A | G | G | A | T | C | T | A | T | G | A | T | G | A | A | A | T | C | C | T | C | A | A | A | A | T | G | G | A | A | G | A | G | A | G | A | T | T | T | C | A | G | A | A | G | C | T | C | G | A | A | C | A | A | G | A | G | C | A | A | C | A | A | G | A | T | C | A | C | T | T | C | T | C | A | C | A | G | A | A | G | A | T | T | G | A | T | C | A | G | G | C | A | T | C | T | G | C | T | A | C | C | C | T | A | A | T | G | G | C | A | G | A | A | A | T | C | A | A | G | A | G | A | A | T | A | C | A | G | T | C | A | A |  | : |  | 1 | 7 | 1 | 0 |
| K | X | 2 | 4 | 9 | 7 | 3 | 5 |  | : |  | A | C | A | A | A | G | T | T | T | A | G | A | T | G | G | C | T | A | C | G | G | T | A | A | C | T | C | A | A | A | A | G | A | C | C | G | A | A | A | G | G | A | T | C | T | A | T | G | A | T | G | A | A | A | T | C | C | T | C | A | A | A | A | T | G | G | A | A | G | A | G | A | G | A | T | T | T | C | A | G | A | A | G | C | T | C | G | A | A | C | A | A | G | A | G | C | A | A | C | A | A | G | A | T | C | A | C | T | T | C | T | C | A | C | A | G | A | A | G | A | T | T | G | A | T | C | A | G | G | C | A | T | C | T | G | C | T | A | C | C | C | T | A | A | T | G | G | C | A | G | A | A | A | T | C | A | A | G | A | G | A | A | T | A | C | A | G | T | C | A | A |  | : |  | 1 | 7 | 1 | 0 |
| M | F | 1 | 9 | 7 | 9 | 1 | 6 |  | : |  | A | C | A | A | A | G | T | T | T | A | G | A | T | G | G | C | T | A | C | G | G | T | A | A | C | T | C | A | A | A | A | G | A | C | C | G | A | A | A | G | G | A | T | C | T | A | T | G | A | T | G | A | A | A | T | C | C | T | C | A | A | A | A | T | G | G | A | A | G | A | G | A | G | A | T | T | T | C | A | G | A | A | G | C | T | C | G | A | A | C | A | A | G | A | G | C | A | A | C | A | A | G | A | T | C | A | C | T | T | C | T | C | A | C | A | G | A | A | G | A | T | T | G | A | T | C | A | G | G | C | A | T | C | T | G | C | T | A | C | C | C | T | A | A | T | G | G | C | A | G | A | A | A | T | C | A | A | A | A | G | A | A | T | A | C | A | G | T | C | A | A |  | : |  | 1 | 7 | 1 | 0 |
| K | T | 2 | 5 | 0 | 6 | 3 | 2 |  | : |  | A | C | A | A | A | G | T | T | T | A | G | A | T | G | G | C | T | A | C | G | G | T | A | A | C | T | C | A | A | A | A | G | A | C | C | G | A | A | A | G | G | A | T | C | T | A | T | G | A | T | G | A | A | A | T | C | C | T | C | A | A | A | A | T | G | G | A | A | G | A | G | A | G | A | T | T | T | C | A | G | A | A | G | C | T | C | G | A | A | C | A | A | G | A | G | C | A | A | C | A | A | G | A | T | C | A | C | T | T | C | T | C | A | C | A | G | A | A | G | A | T | T | G | A | T | C | A | G | G | C | A | T | C | T | G | C | T | A | C | C | C | T | A | A | T | G | G | C | A | G | A | A | A | T | C | A | A | G | A | G | A | A | T | A | C | A | G | T | C | A | A |  | : |  | 1 | 7 | 1 | 0 |
|  |  |  |  |  |  |  |  |  |  |  | A | C | A | A | A | G | T | T | T | A | G | A | T | G | G | C | t | A | C | G | G | T | A | A | C | T | C | A | A | A | A | G | A | C | C | G | A | A | A | G | G | a | T | C | T | A | T | G | A | T | G | A | A | A | T | C | C | T | C | A | A | A | A | T | G | G | A | A | G | A | G | A | G | A | T | T | T | C | A | g | A | A | G | C | T | C | G | A | A | C | A | A | G | A | G | C | A | A | C | A | A | G | A | t | C | A | C | T | T | C | T | C | A | C | A | g | A | A | G | A | T | T | G | A | T | C | A | G | G | C | A | t | C | T | G | C | T | A | C | C | C | T | A | A | T | G | g | C | A | G | A | A | A | T | c | A | A |  | A | G | A | A | T | A | C | A | G | T | C | A | A |  |  |  |  |  |  |  |

|  |  |  |  |  |  |  |  |  |  |  |  |  |  |  |  |  |  |  |  |  |  |  |  |  |  |  |  |  |  |  |  |  |  |  |  |  |  |  |  |  |  |  |  |  |  |  |  |  |  |  |  |  |  |  |  |  |  |  |  |  |  |  |  |  |  |  |  |  |  |  |  |  |  |  |  |  |  |  |  |  |  |  |  |  |  |  |  |  |  |  |  |  |  |  |  |  |  |  |  |  |  |  |  |  |  |  |  |  |  |  |  |  |  |  |  |  |  |  |  |  |  |  |  |  |  |  |  |  |  |  |  |  |  |  |  |  |  |  |  |  |  |  |  |  |  |  |  |  |  |  |  |  |  |  |  |  |  |  |  |  |  |  |  |  |  |  |  |  |  |  |  |  |  |  |  |  |  |  |  |  |  |  |  |  |  |  |  |  |
| --- | --- | --- | --- | --- | --- | --- | --- | --- | --- | --- | --- | --- | --- | --- | --- | --- | --- | --- | --- | --- | --- | --- | --- | --- | --- | --- | --- | --- | --- | --- | --- | --- | --- | --- | --- | --- | --- | --- | --- | --- | --- | --- | --- | --- | --- | --- | --- | --- | --- | --- | --- | --- | --- | --- | --- | --- | --- | --- | --- | --- | --- | --- | --- | --- | --- | --- | --- | --- | --- | --- | --- | --- | --- | --- | --- | --- | --- | --- | --- | --- | --- | --- | --- | --- | --- | --- | --- | --- | --- | --- | --- | --- | --- | --- | --- | --- | --- | --- | --- | --- | --- | --- | --- | --- | --- | --- | --- | --- | --- | --- | --- | --- | --- | --- | --- | --- | --- | --- | --- | --- | --- | --- | --- | --- | --- | --- | --- | --- | --- | --- | --- | --- | --- | --- | --- | --- | --- | --- | --- | --- | --- | --- | --- | --- | --- | --- | --- | --- | --- | --- | --- | --- | --- | --- | --- | --- | --- | --- | --- | --- | --- | --- | --- | --- | --- | --- | --- | --- | --- | --- | --- | --- | --- | --- | --- | --- | --- | --- | --- | --- | --- | --- | --- | --- | --- | --- | --- | --- |
|  |  |  |  |  |  |  |  |  |  |  |  |  |  |  |  |  |  |  |  |  |  |  |  |  |  |  |  |  |  |  |  |  |  |  |  |  |  |  |  |  |  |  |  |  |  |  |  |  |  |  |  |  |  |  |  |  |  |  |  |  |  |  |  |  |  |  |  |  |  |  |  |  |  |  |  |  |  |  |  |  |  |  |  |  |  |  |  |  |  |  |  |  |  |  |  |  |  |  |  |  |  |  |  |  |  |  |  |  |  |  |  |  |  |  |  |  |  |  |  |  |  |  |  |  |  |  |  |  |  |  |  |  |  |  |  |  |  |  |  |  |  |  |  |  |  |  |  |  |  |  |  |  |  |  |  |  |  |  |  |  |  |  |  |  |  |  |  |  |  |  |  |  |  |  |  |  |  |  |  |  |  |  |  |  |  |  |  |  |
|  |  |  |  |  |  |  |  |  |  |  |  |  |  |  |  |  | 1 | 7 | 2 | 0 |  |  |  |  |  |  |  |  |  | \* |  |  |  |  |  |  | 1 | 7 | 4 | 0 |  |  |  |  |  |  |  |  |  | \* |  |  |  |  |  |  | 1 | 7 | 6 | 0 |  |  |  |  |  |  |  |  |  | \* |  |  |  |  |  |  | 1 | 7 | 8 | 0 |  |  |  |  |  |  |  |  |  | \* |  |  |  |  |  |  | 1 | 8 | 0 | 0 |  |  |  |  |  |  |  |  |  | \* |  |  |  |  |  |  | 1 | 8 | 2 | 0 |  |  |  |  |  |  |  |  |  | \* |  |  |  |  |  |  | 1 | 8 | 4 | 0 |  |  |  |  |  |  |  |  |  | \* |  |  |  |  |  |  | 1 | 8 | 6 | 0 |  |  |  |  |  |  |  |  |  | \* |  |  |  |  |  |  | 1 | 8 | 8 | 0 |  |  |  |  |  |  |  |  |
| M | N | 9 | 5 | 6 | 5 | 2 | 0 |  | : |  | A | A | T | T | G | G | A | C | A | G | A | T | G | T | G | A | C | T | G | T | A | A | C | A | A | A | G | A | A | A | T | C | T | T | A | G | A | T | G | C | T | T | T | A | A | A | A | G | C | A | C | A | G | G | A | T | A | G | A | A | C | T | A | A | G | G | G | A | A | A | A | G | G | A | T | C | G | G | G | - | - | - | C | T | C | A | A | A | C | C | T | T | G | C | C | G | A | T | G | C | C | T | T | A | G | A | C | T | T | A | G | C | C | A | A | T | A | G | A | G | A | T | C | C | T | C | C | T | A | G | A | A | G | G | A | A | C | T | C | T | A | T | C | A | A | G | G | G | T | A | C | A | G | A | A | A | A | C | T | G | G | C | A | C | C | C | T | C | A | A | G |  | : |  | 1 | 8 | 7 | 8 |
| M | T | 0 | 1 | 2 | 7 | 3 | 2 |  | : |  | A | A | T | T | G | G | A | C | A | G | A | T | G | T | G | A | C | T | G | T | A | A | C | A | A | A | G | A | A | A | T | C | T | T | A | G | A | T | G | C | T | T | T | A | A | A | A | G | C | A | C | A | G | G | A | T | A | G | A | A | C | T | A | A | G | G | G | A | A | A | A | G | G | A | T | C | G | G | G | - | - | - | C | T | C | A | A | A | C | C | T | T | G | C | C | G | A | T | G | C | C | T | T | A | G | A | C | T | T | A | G | C | C | A | A | T | A | G | A | A | G | T | C | C | T | C | C | C | A | G | G | A | G | A | A | A | C | T | C | T | A | T | C | A | A | G | G | G | T | A | C | A | G | A | A | A | A | C | T | G | G | C | A | C | C | C | T | C | A | A | G |  | : |  | 1 | 8 | 7 | 8 |
| M | T | 0 | 1 | 2 | 7 | 3 | 4 |  | : |  | A | A | T | T | G | G | A | C | A | G | A | T | G | T | G | A | C | T | G | T | A | A | C | A | A | A | G | A | A | A | T | C | T | T | A | G | A | T | G | C | T | T | T | A | A | A | A | G | C | A | C | A | G | G | A | T | A | G | A | A | C | T | A | A | G | G | A | A | A | A | A | G | G | A | T | C | G | G | G | - | - | - | C | T | C | A | A | A | C | C | T | T | G | C | C | G | A | T | G | C | C | T | T | A | G | A | C | T | T | A | G | C | C | A | A | T | A | G | A | A | G | T | C | C | T | C | C | C | A | G | G | A | G | A | A | A | C | T | C | T | A | T | C | A | A | G | G | G | T | A | C | A | G | A | A | A | A | C | T | G | G | C | A | C | C | C | T | C | A | A | G |  | : |  | 1 | 8 | 7 | 8 |
| M | T | 0 | 2 | 7 | 0 | 0 | 6 |  | : |  | A | A | T | T | G | G | A | C | A | G | A | T | G | T | G | A | C | T | G | T | A | A | C | A | A | A | G | A | A | A | T | C | T | T | A | G | A | T | G | C | T | T | T | A | A | A | A | G | C | A | C | A | G | G | A | T | A | G | A | A | C | T | A | A | G | G | G | A | A | A | A | G | G | A | T | C | G | G | G | - | - | - | C | T | C | A | A | A | C | C | T | T | G | C | C | G | A | T | G | C | C | T | T | A | G | A | C | T | T | A | G | C | C | A | A | T | A | G | A | A | G | T | C | C | T | C | C | C | A | G | G | A | G | A | A | A | C | T | C | T | A | T | C | A | A | G | G | G | T | A | C | A | G | A | A | A | A | C | T | G | G | C | A | C | C | C | T | C | A | A | G |  | : |  | 1 | 8 | 7 | 8 |
| M | T | 0 | 2 | 7 | 0 | 0 | 7 |  | : |  | A | A | T | T | G | G | A | C | A | G | A | T | G | T | G | A | C | T | G | T | A | A | C | A | A | A | G | A | A | A | T | C | T | T | A | G | A | T | G | C | T | T | T | A | A | A | A | G | C | A | C | A | G | G | A | T | A | G | A | A | C | T | A | A | G | G | G | A | A | A | A | G | G | A | T | C | A | G | G | T | A | G | C | T | C | A | A | G | C | T | T | A | G | C | C | G | A | T | G | C | C | T | T | A | G | A | C | T | T | A | G | C | C | A | A | T | A | G | A | G | A | T | C | C | T | C | C | T | A | G | A | A | G | G | A | A | C | T | C | T | A | T | C | A | A | G | G | G | T | A | C | A | G | A | A | A | A | C | T | G | G | C | A | C | C | C | T | C | A | A | G |  | : |  | 1 | 8 | 8 | 1 |
| M | T | 0 | 3 | 6 | 0 | 5 | 3 |  | : |  | A | A | T | T | G | G | A | C | A | G | A | T | G | T | G | A | C | T | G | T | A | A | C | A | A | A | G | A | A | A | T | C | T | T | A | G | A | T | G | C | T | T | T | A | A | A | A | G | C | A | C | A | G | G | A | T | A | G | A | A | C | T | A | A | G | G | G | A | A | A | A | G | G | A | T | C | A | G | G | T | A | G | C | T | C | A | A | G | C | T | T | A | G | C | C | G | A | T | G | C | C | T | T | A | G | A | C | T | T | A | G | C | C | A | A | T | A | G | A | G | A | T | C | C | T | C | C | T | A | G | A | A | G | G | A | A | C | T | C | T | A | T | C | A | A | G | G | G | T | A | C | A | G | A | A | A | A | C | T | G | G | C | A | C | C | C | T | C | A | A | G |  | : |  | 1 | 8 | 8 | 1 |
| M | T | 0 | 3 | 6 | 0 | 5 | 4 |  | : |  | A | A | T | T | G | G | A | C | A | G | A | T | G | T | G | A | C | T | G | T | A | A | C | A | A | A | G | A | A | A | T | C | T | T | A | G | A | T | G | C | T | T | T | A | A | A | A | G | C | A | C | A | G | G | A | T | A | G | A | A | C | T | A | A | G | G | G | A | A | A | A | G | G | A | T | C | A | G | G | T | A | G | C | T | C | A | A | G | C | T | T | A | G | C | C | G | A | T | G | C | C | T | T | A | G | A | C | T | T | A | G | C | C | A | A | T | A | G | A | G | A | T | C | C | T | C | C | T | A | G | A | A | G | G | A | A | C | T | C | T | A | T | C | A | A | G | G | G | T | A | C | A | G | A | A | A | A | C | T | G | G | C | A | C | C | C | T | C | A | A | G |  | : |  | 1 | 8 | 8 | 1 |
| M | T | 0 | 3 | 6 | 0 | 5 | 5 |  | : |  | A | A | T | T | A | G | A | T | A | G | A | T | G | T | G | A | C | T | G | T | A | A | C | A | A | A | G | A | A | A | T | C | T | T | A | G | A | T | G | C | T | T | T | A | A | A | A | G | C | A | C | A | G | G | A | T | A | G | A | A | C | T | A | A | G | G | G | A | A | A | A | G | G | A | T | C | A | G | G | A | A | G | C | T | C | A | A | G | C | T | T | A | G | C | C | G | A | T | G | C | C | T | T | A | G | A | C | T | T | A | G | C | C | A | A | T | A | G | A | G | A | T | C | C | C | C | C | T | A | G | A | A | G | G | A | C | C | T | C | T | A | T | C | A | A | G | G | G | T | A | C | A | G | A | A | A | A | C | T | G | G | C | A | C | C | C | T | C | A | A | G |  | : |  | 1 | 8 | 8 | 1 |
| M | T | 0 | 3 | 6 | 0 | 5 | 6 |  | : |  | A | A | T | T | G | G | A | C | A | G | A | T | G | T | G | A | C | T | G | T | A | A | C | A | A | A | G | A | A | A | T | C | T | T | A | G | A | T | G | C | T | T | T | A | A | A | A | G | C | A | C | A | G | G | A | T | A | G | A | A | C | T | A | A | G | G | G | A | A | A | A | G | G | A | T | C | G | G | G | - | - | - | C | T | C | A | A | A | C | C | T | T | G | C | C | G | A | T | G | C | C | T | T | A | G | A | C | T | T | A | G | C | C | A | A | T | A | G | A | A | G | T | C | C | T | C | C | C | A | G | G | A | G | A | A | A | C | T | C | T | A | T | C | A | A | G | G | G | T | A | C | A | G | A | A | A | A | C | T | G | G | C | A | C | C | C | T | C | A | A | G |  | : |  | 1 | 8 | 7 | 8 |
| M | T | 0 | 3 | 6 | 0 | 5 | 7 |  | : |  | A | A | T | T | G | G | A | C | A | G | A | T | G | T | G | A | C | T | G | T | A | A | C | A | A | A | G | A | A | A | T | C | T | T | A | G | A | T | G | C | T | T | T | A | A | A | A | G | C | A | C | A | G | G | A | T | A | G | A | A | C | T | A | A | G | G | G | A | A | A | A | G | G | A | T | C | G | G | G | - | - | - | C | T | C | A | A | A | C | C | T | T | G | C | C | G | A | T | G | C | C | T | T | A | G | A | C | T | T | A | G | C | C | A | A | T | A | G | A | A | G | T | C | C | T | C | C | C | A | G | G | A | G | A | A | A | C | T | C | T | A | T | C | A | A | G | G | G | T | A | C | A | G | A | A | A | A | C | T | G | G | C | A | C | C | C | T | C | A | A | G |  | : |  | 1 | 8 | 7 | 8 |
| K | X | 2 | 4 | 9 | 7 | 3 | 8 |  | : |  | A | A | T | T | G | G | A | C | A | G | A | T | G | T | G | A | C | T | G | T | A | A | C | A | A | A | G | A | A | A | T | C | T | T | A | G | A | T | G | C | T | T | T | A | A | A | A | G | C | A | C | A | G | G | A | T | A | G | A | A | C | T | A | A | G | G | G | A | A | A | A | G | G | A | T | C | G | G | G | - | - | - | C | T | C | A | A | A | C | C | T | T | G | C | C | G | A | T | G | C | C | T | T | A | G | A | C | T | T | A | G | C | C | A | A | T | A | G | A | A | G | T | C | C | T | C | C | C | A | G | G | A | G | A | A | A | C | T | C | T | A | T | C | A | A | G | G | G | T | A | C | A | G | A | A | A | A | C | T | G | G | C | A | C | C | C | T | C | A | A | G |  | : |  | 1 | 8 | 7 | 8 |
| K | X | 2 | 4 | 9 | 7 | 3 | 7 |  | : |  | A | A | T | T | G | G | A | C | A | A | A | T | G | T | G | A | C | T | G | T | A | A | C | A | A | A | G | A | A | A | T | C | T | T | A | G | A | T | G | C | T | T | T | A | A | A | A | G | C | A | C | A | G | G | A | T | A | G | A | A | C | T | A | A | G | G | G | A | A | A | A | G | G | A | T | C | G | G | G | - | - | - | C | T | C | A | A | A | C | C | T | T | G | C | C | G | A | T | G | C | C | T | T | A | G | A | C | T | T | A | G | C | C | A | A | T | A | G | A | A | G | T | C | C | T | C | C | C | A | G | G | A | G | A | A | A | C | T | C | T | A | T | C | A | A | G | G | G | T | A | C | A | G | A | A | A | A | C | T | G | G | C | A | C | C | C | T | C | A | A | G |  | : |  | 1 | 8 | 7 | 8 |
| K | X | 2 | 4 | 9 | 7 | 3 | 6 |  | : |  | A | A | T | T | G | G | A | C | A | G | A | T | G | T | G | A | C | T | G | T | A | A | C | A | A | A | G | A | A | A | T | C | T | T | A | G | A | T | G | C | T | T | T | A | A | A | A | G | C | A | C | A | G | G | A | T | A | G | A | A | C | T | A | A | G | G | G | A | A | A | A | G | G | A | T | C | G | G | G | - | - | - | C | T | C | A | A | A | C | C | T | T | G | C | C | G | A | T | G | C | C | T | T | A | G | A | C | T | T | A | G | C | C | A | A | T | A | G | A | A | G | T | C | C | T | C | C | C | A | G | G | A | C | A | A | A | C | T | C | T | A | T | C | A | A | G | G | G | T | A | C | A | G | A | A | A | A | C | T | G | G | C | A | C | C | C | T | C | A | A | G |  | : |  | 1 | 8 | 7 | 8 |
| K | X | 2 | 4 | 9 | 7 | 3 | 5 |  | : |  | A | A | T | T | G | G | A | C | A | G | A | T | G | T | G | A | C | T | G | T | A | A | C | A | A | A | G | A | A | A | T | C | T | T | A | G | A | T | G | C | T | T | T | A | A | A | A | G | C | A | C | A | G | G | A | T | A | G | A | A | C | T | A | A | G | G | G | A | A | A | A | G | G | A | T | C | G | G | G | - | - | - | C | T | C | A | A | A | C | C | T | T | G | C | C | G | A | T | G | C | C | T | T | A | G | A | C | T | T | A | G | C | C | A | A | T | A | G | A | A | G | T | C | C | T | C | C | C | A | G | G | A | G | A | A | A | C | T | C | T | A | T | C | A | A | G | G | G | T | A | C | A | G | A | A | A | A | C | T | G | G | C | A | C | C | C | T | C | A | A | G |  | : |  | 1 | 8 | 7 | 8 |
| M | F | 1 | 9 | 7 | 9 | 1 | 6 |  | : |  | A | A | T | T | G | G | A | C | A | G | A | T | G | T | G | A | C | T | G | T | A | A | C | A | A | A | G | A | A | A | T | C | T | T | A | G | A | T | G | C | T | T | T | A | A | A | A | G | C | A | C | A | G | G | A | T | A | G | A | A | C | T | A | A | G | G | G | A | A | A | A | G | G | A | T | C | A | G | G | T | A | G | C | T | C | A | A | G | C | T | T | A | G | C | C | G | A | T | G | C | C | T | T | A | G | A | C | T | T | A | G | C | C | A | A | T | A | G | A | G | A | T | C | C | T | C | C | T | A | A | A | A | G | G | A | A | C | T | C | T | A | T | C | A | A | G | G | G | T | A | C | A | G | A | A | A | A | C | T | G | G | C | A | C | C | C | T | C | A | A | G |  | : |  | 1 | 8 | 8 | 1 |
| K | T | 2 | 5 | 0 | 6 | 3 | 2 |  | : |  | A | A | T | T | G | G | A | C | A | G | A | T | G | T | G | A | C | T | G | T | A | A | C | A | A | A | G | A | A | A | T | C | T | T | A | G | A | T | G | C | T | T | T | A | A | A | A | G | C | A | C | A | G | G | A | T | A | G | A | A | C | T | A | A | G | G | G | A | A | A | A | G | G | A | T | C | A | G | G | T | A | G | C | T | C | A | A | G | C | T | T | A | G | C | C | G | A | T | G | C | C | T | T | A | G | A | C | T | T | A | G | C | C | A | A | T | A | G | A | G | A | T | C | C | T | C | T | T | A | G | A | A | G | G | A | A | C | T | C | T | A | T | C | A | A | G | G | G | T | A | C | A | G | A | A | A | A | C | T | G | G | C | A | C | C | C | T | C | A | A | G |  | : |  | 1 | 8 | 8 | 1 |
|  |  |  |  |  |  |  |  |  |  |  | A | A | T | T | g | G | A | c | A | g | A | T | G | T | G | A | C | T | G | T | A | A | C | A | A | A | G | A | A | A | T | C | T | T | A | G | A | T | G | C | T | T | T | A | A | A | A | G | C | A | C | A | G | G | A | T | A | G | A | A | C | T | A | A | G | G | g | A | A | A | A | G | G | A | T | C |  | G | G |  |  |  | C | T | C | A | A |  | C |  | T |  | G | C | C | G | A | T | G | C | C | T | T | A | G | A | C | T | T | A | G | C | C | A | A | T | A | G | A |  |  | T | C | C | t | C | c |  | A | g |  | A | g |  | A | a | C | T | C | T | A | T | C | A | A | G | G | G | T | A | C | A | G | A | A | A | A | C | T | G | G | C | A | C | C | C | T | C | A | A | G |  |  |  |  |  |  |  |

|  |  |  |  |  |  |  |  |  |  |  |  |  |  |  |  |  |  |  |  |  |  |  |  |  |  |  |  |  |  |  |  |  |  |  |  |  |  |  |  |  |  |  |  |  |  |  |  |  |  |  |  |  |  |  |  |  |  |  |  |  |  |  |  |  |  |  |  |  |  |  |  |  |  |  |  |  |  |  |  |  |  |  |  |  |  |  |  |  |  |  |  |  |  |  |  |  |  |  |  |  |  |  |  |  |  |  |  |  |  |  |  |  |  |  |  |  |  |  |  |  |  |  |  |  |  |  |  |  |  |  |  |  |  |  |  |  |  |  |  |  |  |  |  |  |  |  |  |  |  |  |  |  |  |  |  |  |  |  |  |  |  |  |  |  |  |  |  |  |  |  |  |  |  |  |  |  |  |  |  |  |  |  |  |  |  |  |  |  |
| --- | --- | --- | --- | --- | --- | --- | --- | --- | --- | --- | --- | --- | --- | --- | --- | --- | --- | --- | --- | --- | --- | --- | --- | --- | --- | --- | --- | --- | --- | --- | --- | --- | --- | --- | --- | --- | --- | --- | --- | --- | --- | --- | --- | --- | --- | --- | --- | --- | --- | --- | --- | --- | --- | --- | --- | --- | --- | --- | --- | --- | --- | --- | --- | --- | --- | --- | --- | --- | --- | --- | --- | --- | --- | --- | --- | --- | --- | --- | --- | --- | --- | --- | --- | --- | --- | --- | --- | --- | --- | --- | --- | --- | --- | --- | --- | --- | --- | --- | --- | --- | --- | --- | --- | --- | --- | --- | --- | --- | --- | --- | --- | --- | --- | --- | --- | --- | --- | --- | --- | --- | --- | --- | --- | --- | --- | --- | --- | --- | --- | --- | --- | --- | --- | --- | --- | --- | --- | --- | --- | --- | --- | --- | --- | --- | --- | --- | --- | --- | --- | --- | --- | --- | --- | --- | --- | --- | --- | --- | --- | --- | --- | --- | --- | --- | --- | --- | --- | --- | --- | --- | --- | --- | --- | --- | --- | --- | --- | --- | --- | --- | --- | --- | --- | --- | --- | --- | --- | --- |
|  |  |  |  |  |  |  |  |  |  |  |  |  |  |  |  |  |  |  |  |  |  |  |  |  |  |  |  |  |  |  |  |  |  |  |  |  |  |  |  |  |  |  |  |  |  |  |  |  |  |  |  |  |  |  |  |  |  |  |  |  |  |  |  |  |  |  |  |  |  |  |  |  |  |  |  |  |  |  |  |  |  |  |  |  |  |  |  |  |  |  |  |  |  |  |  |  |  |  |  |  |  |  |  |  |  |  |  |  |  |  |  |  |  |  |  |  |  |  |  |  |  |  |  |  |  |  |  |  |  |  |  |  |  |  |  |  |  |  |  |  |  |  |  |  |  |  |  |  |  |  |  |  |  |  |  |  |  |  |  |  |  |  |  |  |  |  |  |  |  |  |  |  |  |  |  |  |  |  |  |  |  |  |  |  |  |  |  |  |
|  |  |  |  |  |  |  |  |  |  |  |  |  |  |  |  |  |  |  | \* |  |  |  |  |  |  | 1 | 9 | 0 | 0 |  |  |  |  |  |  |  |  |  | \* |  |  |  |  |  |  | 1 | 9 | 2 | 0 |  |  |  |  |  |  |  |  |  | \* |  |  |  |  |  |  | 1 | 9 | 4 | 0 |  |  |  |  |  |  |  |  |  | \* |  |  |  |  |  |  | 1 | 9 | 6 | 0 |  |  |  |  |  |  |  |  |  | \* |  |  |  |  |  |  | 1 | 9 | 8 | 0 |  |  |  |  |  |  |  |  |  | \* |  |  |  |  |  |  | 2 | 0 | 0 | 0 |  |  |  |  |  |  |  |  |  | \* |  |  |  |  |  |  | 2 | 0 | 2 | 0 |  |  |  |  |  |  |  |  |  | \* |  |  |  |  |  |  | 2 | 0 | 4 | 0 |  |  |  |  |  |  |  |  |  | \* |  |  |  |  |  |  |  |  |  |
| M | N | 9 | 5 | 6 | 5 | 2 | 0 |  | : |  | A | T | T | T | A | C | C | T | C | T | C | A | C | C | A | A | G | T | G | G | T | A | A | A | C | A | T | G | T | C | T | T | C | T | C | G | C | A | G | A | G | A | A | A | G | A | C | T | C | G | A | A | C | A | A | C | T | G | T | T | C | G | A | A | G | A | A | G | A | T | C | A | T | C | C | A | G | A | A | A | T | G | G | A | T | A | C | A | A | T | A | A | T | A | C | A | A | T | A | T | C | T | G | T | C | T | T | T | A | C | T | T | G | A | T | G | A | T | G | A | A | C | T | T | G | A | T | A | A | T | T | G | T | C | A | G | G | A | A | G | A | A | A | A | G | T | T | A | C | A | G | G | T | A | C | T | T | G | T | A | G | C | A | A | A | A | G | A | A | A | T |  | : |  | 2 | 0 | 4 | 9 |
| M | T | 0 | 1 | 2 | 7 | 3 | 2 |  | : |  | A | T | T | T | A | C | C | T | C | T | T | A | C | A | A | A | G | T | G | G | T | A | A | A | T | A | T | G | T | C | T | T | C | T | C | G | C | A | G | A | G | A | A | A | G | A | C | T | C | G | A | A | C | A | A | C | T | G | T | T | C | G | A | A | G | A | A | G | A | T | C | A | T | C | C | A | G | A | A | A | T | G | G | A | T | A | C | C | A | T | A | A | T | A | C | A | A | T | A | T | C | T | G | T | C | T | T | T | A | C | T | T | G | A | T | G | A | T | G | A | A | C | T | T | G | A | T | A | A | T | T | G | T | C | A | G | G | A | A | G | A | A | A | A | G | T | T | A | C | A | G | G | T | A | C | T | T | G | T | A | G | C | A | A | A | A | G | A | A | A | T |  | : |  | 2 | 0 | 4 | 9 |
| M | T | 0 | 1 | 2 | 7 | 3 | 4 |  | : |  | A | T | T | T | A | C | C | T | C | T | T | A | C | A | A | A | G | T | G | G | T | A | A | A | T | A | T | G | T | C | T | T | C | T | C | G | C | A | G | A | G | A | A | A | G | A | C | T | C | G | A | A | C | A | A | C | T | G | T | T | C | G | A | A | G | A | A | G | A | T | C | A | T | C | C | A | G | A | A | A | T | G | G | A | T | A | C | A | A | T | A | A | T | A | C | A | A | T | A | T | C | T | G | T | C | T | T | T | A | C | T | T | G | A | T | G | A | T | G | A | A | C | T | T | G | A | T | A | A | T | T | G | T | C | A | G | G | A | A | G | A | A | A | A | G | T | T | A | C | A | G | G | T | A | C | T | T | G | T | A | G | C | A | A | A | A | G | A | A | A | T |  | : |  | 2 | 0 | 4 | 9 |
| M | T | 0 | 2 | 7 | 0 | 0 | 6 |  | : |  | A | T | T | T | A | C | C | T | C | T | T | A | C | A | A | A | G | T | G | G | T | A | A | A | T | A | T | G | T | C | T | T | C | T | C | G | C | A | G | A | G | A | A | A | G | A | C | T | C | G | A | A | C | A | A | C | T | G | T | T | C | G | A | A | G | A | A | G | A | T | C | A | T | C | C | A | G | A | A | A | T | G | G | A | T | A | C | A | A | T | A | A | T | A | C | A | A | T | A | T | C | T | G | T | C | T | T | T | A | C | T | T | G | A | T | G | A | T | G | A | A | C | T | T | G | A | T | A | A | T | T | G | T | C | A | G | G | A | A | G | A | A | A | A | G | T | T | A | C | A | G | G | T | A | C | T | T | G | T | A | G | C | A | A | A | A | G | A | A | A | T |  | : |  | 2 | 0 | 4 | 9 |
| M | T | 0 | 2 | 7 | 0 | 0 | 7 |  | : |  | A | T | T | T | A | C | C | T | C | T | C | A | C | C | A | A | G | T | G | G | T | A | A | A | C | A | T | G | T | C | T | T | C | T | C | G | C | A | G | A | G | A | A | A | G | A | C | T | C | G | A | A | C | A | A | C | T | G | T | T | C | G | A | A | G | A | A | G | A | T | C | A | T | C | C | A | G | A | A | A | T | G | G | A | T | A | C | A | A | T | A | A | T | A | C | A | A | T | A | T | C | T | G | T | C | T | T | T | A | C | T | T | G | A | T | G | A | T | G | A | A | C | T | T | G | A | T | A | A | T | T | G | T | C | A | G | G | A | A | G | A | A | A | A | G | T | T | A | C | A | G | G | T | A | C | T | T | G | T | A | G | C | A | A | A | A | G | A | A | A | T |  | : |  | 2 | 0 | 5 | 2 |
| M | T | 0 | 3 | 6 | 0 | 5 | 3 |  | : |  | A | T | T | T | A | C | C | T | C | T | C | A | C | C | A | A | G | T | G | G | T | A | A | A | C | A | T | G | T | C | T | T | C | T | C | G | C | A | G | A | G | A | A | A | G | A | C | T | C | G | A | A | C | A | A | C | T | G | T | T | C | G | A | A | G | A | A | G | A | T | C | A | T | C | C | A | G | A | A | A | T | G | G | A | T | A | C | A | A | T | A | A | T | A | C | A | A | T | A | T | C | T | G | T | C | T | T | T | A | C | T | T | G | A | C | G | A | T | G | A | A | C | T | T | G | A | T | A | A | T | T | G | T | C | A | G | G | A | A | G | A | A | A | A | G | T | T | A | C | A | G | G | T | A | C | T | T | G | T | A | G | C | A | A | A | A | G | A | A | A | T |  | : |  | 2 | 0 | 5 | 2 |
| M | T | 0 | 3 | 6 | 0 | 5 | 4 |  | : |  | A | T | T | T | A | C | C | T | C | T | C | A | C | C | A | A | G | T | G | G | T | A | A | A | C | A | T | G | T | C | T | T | C | T | C | G | C | A | G | A | G | A | A | A | G | A | C | T | C | G | A | A | C | A | A | C | T | G | T | T | C | G | A | A | G | A | A | G | A | T | C | A | T | C | C | A | G | A | A | A | T | G | G | A | T | A | C | A | A | T | A | A | T | A | C | A | A | T | A | T | C | T | G | T | C | T | T | T | A | C | T | T | G | A | T | G | A | T | G | A | A | C | T | T | G | A | T | A | A | T | T | G | T | C | A | G | G | A | A | G | A | A | A | A | G | T | T | A | C | A | G | G | T | A | C | T | T | G | T | A | G | C | A | A | A | A | G | A | A | A | T |  | : |  | 2 | 0 | 5 | 2 |
| M | T | 0 | 3 | 6 | 0 | 5 | 5 |  | : |  | A | T | T | T | A | C | C | T | C | T | C | A | C | C | A | A | G | T | G | G | T | A | A | A | C | A | T | G | T | C | T | T | C | T | C | G | C | A | G | A | G | A | A | A | G | A | C | T | C | G | A | A | C | A | A | T | T | G | T | T | C | G | A | A | G | A | A | G | A | T | C | A | T | C | C | A | G | A | A | A | T | G | G | A | T | A | C | A | A | T | A | A | T | A | C | A | A | T | A | T | C | T | G | T | C | T | T | T | A | C | T | T | G | A | T | G | A | T | G | A | A | C | T | T | G | A | T | A | A | T | T | G | T | C | A | G | G | A | A | G | A | A | A | A | G | T | T | A | C | A | G | G | T | A | C | T | C | G | T | A | G | C | A | A | A | A | G | A | G | A | T |  | : |  | 2 | 0 | 5 | 2 |
| M | T | 0 | 3 | 6 | 0 | 5 | 6 |  | : |  | A | T | T | T | A | C | C | T | C | T | T | A | C | A | A | A | G | T | G | G | T | A | A | A | T | A | T | G | T | C | T | T | C | T | C | G | C | A | G | A | G | A | A | A | G | A | C | T | C | G | A | A | C | A | A | C | T | G | T | C | C | G | A | A | G | A | A | G | A | T | C | A | T | C | C | A | G | A | G | A | T | G | G | A | T | A | C | A | A | T | A | A | T | A | C | A | A | T | A | T | C | T | G | T | C | T | T | T | A | C | T | T | G | A | T | G | A | T | G | A | A | C | T | T | G | A | T | A | A | T | T | G | T | C | A | G | G | A | A | G | A | A | A | A | G | T | T | A | C | A | G | G | T | A | C | T | T | G | T | A | G | C | A | A | A | A | G | A | A | A | T |  | : |  | 2 | 0 | 4 | 9 |
| M | T | 0 | 3 | 6 | 0 | 5 | 7 |  | : |  | A | T | T | T | A | C | C | T | C | T | T | A | C | A | A | A | G | T | G | G | T | A | A | A | T | A | T | G | T | C | T | T | C | T | C | G | C | A | G | A | G | A | A | A | G | A | C | T | C | G | A | A | C | A | A | C | T | G | T | T | C | G | A | A | G | A | A | G | A | T | C | A | T | C | C | A | G | A | A | A | T | G | G | A | T | A | C | A | A | T | A | A | T | A | C | A | A | T | A | T | C | T | G | T | C | T | T | T | A | C | T | T | G | A | T | G | A | T | G | A | A | C | T | T | G | A | T | A | A | T | T | G | T | C | A | G | G | A | A | G | A | A | A | A | G | T | T | A | C | A | G | G | T | A | C | T | T | G | T | A | G | C | A | A | A | A | G | A | A | A | T |  | : |  | 2 | 0 | 4 | 9 |
| K | X | 2 | 4 | 9 | 7 | 3 | 8 |  | : |  | A | T | T | T | A | C | C | T | C | T | T | A | C | A | A | A | G | T | G | G | T | A | A | A | T | A | T | G | T | C | T | T | C | T | C | G | C | A | G | A | G | A | A | A | G | A | C | T | C | G | A | A | C | A | A | C | T | G | T | T | C | G | A | A | G | A | A | G | A | T | C | A | T | C | C | A | G | A | A | A | T | G | G | A | T | A | C | A | A | T | A | A | T | A | C | A | A | T | A | T | C | T | G | T | C | T | T | T | A | C | T | T | G | A | T | G | A | T | G | A | A | C | T | T | G | A | T | A | A | T | T | G | T | C | A | G | G | A | A | G | A | A | A | A | G | T | T | A | C | A | G | G | T | A | C | T | T | G | T | A | G | C | A | A | A | A | G | A | A | A | T |  | : |  | 2 | 0 | 4 | 9 |
| K | X | 2 | 4 | 9 | 7 | 3 | 7 |  | : |  | A | T | T | T | A | C | C | T | C | T | T | A | C | A | A | A | G | T | G | G | T | A | A | A | T | A | T | G | T | C | T | T | C | T | C | G | C | A | G | A | G | A | A | A | G | A | C | T | C | G | A | A | C | A | A | C | T | G | T | T | C | G | A | A | G | A | A | G | A | T | C | A | T | C | C | A | G | A | A | A | T | G | G | A | T | A | C | A | A | T | A | A | T | A | C | A | A | T | A | T | C | T | G | T | C | T | T | T | A | C | T | T | G | A | T | G | A | T | G | A | A | C | T | T | G | A | T | A | A | T | T | G | T | C | A | G | G | A | A | G | A | A | A | A | G | T | T | A | C | A | G | G | T | A | C | T | T | G | T | A | G | C | A | A | A | A | G | A | A | A | T |  | : |  | 2 | 0 | 4 | 9 |
| K | X | 2 | 4 | 9 | 7 | 3 | 6 |  | : |  | A | T | T | T | A | C | C | T | C | T | T | A | C | A | A | A | G | T | G | G | T | A | A | A | T | A | T | G | T | C | T | T | C | T | C | G | C | A | G | A | G | A | A | A | G | A | C | T | C | G | A | A | C | A | A | C | T | G | T | T | C | G | A | A | G | A | A | G | A | T | C | A | T | C | C | A | G | A | A | A | T | G | G | A | T | A | C | A | A | T | A | A | T | A | C | A | A | T | A | T | C | T | G | T | C | T | T | T | A | C | T | T | G | A | T | G | A | T | G | A | A | C | T | T | G | A | T | A | A | T | T | G | T | C | A | G | G | A | A | G | A | A | A | A | G | T | T | A | C | A | G | G | T | A | C | T | T | G | T | A | G | C | A | A | A | A | G | A | A | A | T |  | : |  | 2 | 0 | 4 | 9 |
| K | X | 2 | 4 | 9 | 7 | 3 | 5 |  | : |  | A | T | T | T | A | C | C | T | C | T | T | A | C | A | A | A | G | T | G | G | T | A | A | A | T | A | T | G | T | C | T | T | C | T | C | G | C | A | G | A | G | A | A | A | G | A | C | T | C | G | A | A | C | A | A | C | T | G | T | T | C | G | A | A | G | A | A | G | A | T | C | A | T | C | C | A | G | A | A | A | T | G | G | A | T | A | C | A | A | T | A | A | T | A | C | A | A | T | A | T | C | T | G | T | C | T | T | T | A | C | T | T | G | A | T | G | A | T | G | A | A | C | T | T | G | A | T | A | A | T | T | G | T | C | A | G | G | A | A | G | A | A | A | A | G | T | T | A | C | A | G | G | T | A | C | T | T | G | T | A | G | C | A | A | A | A | G | A | A | A | T |  | : |  | 2 | 0 | 4 | 9 |
| M | F | 1 | 9 | 7 | 9 | 1 | 6 |  | : |  | A | T | T | T | A | C | C | T | C | T | C | A | C | C | A | A | G | T | G | G | T | A | A | A | C | A | T | G | T | C | T | T | C | T | C | G | C | A | G | A | G | A | A | A | G | A | C | T | C | G | A | A | C | A | A | C | T | G | T | T | C | G | A | A | G | A | A | G | A | T | C | A | T | C | C | A | G | A | A | A | T | G | G | A | T | A | C | A | A | T | A | A | T | A | C | A | A | T | A | T | C | T | G | T | C | T | T | T | A | C | T | T | G | A | T | G | A | T | G | A | A | C | T | T | G | A | T | A | A | T | T | G | T | C | A | G | G | A | A | G | A | A | A | A | G | T | T | A | C | A | G | G | T | A | C | T | T | G | T | A | G | C | A | A | A | A | G | A | A | A | T |  | : |  | 2 | 0 | 5 | 2 |
| K | T | 2 | 5 | 0 | 6 | 3 | 2 |  | : |  | A | T | T | T | A | C | C | T | C | T | C | A | C | C | A | A | G | T | G | G | T | A | A | A | C | A | T | G | T | C | T | T | C | T | C | G | C | A | G | A | G | A | A | A | G | A | C | T | C | G | A | A | C | A | A | C | T | G | T | T | C | G | A | A | G | A | A | G | A | T | C | A | T | C | C | A | G | A | A | A | T | G | G | A | T | A | C | A | A | T | A | A | T | A | C | A | A | T | A | T | C | T | G | T | C | T | T | T | A | C | T | T | G | A | T | G | A | T | G | A | A | C | T | T | G | A | T | A | A | T | T | G | T | C | A | G | G | A | A | G | A | A | A | A | G | T | T | A | C | A | G | G | T | A | C | T | T | G | T | A | G | C | A | A | A | A | G | A | A | A | T |  | : |  | 2 | 0 | 5 | 2 |
|  |  |  |  |  |  |  |  |  |  |  | A | T | T | T | A | C | C | T | C | T |  | A | C |  | A | A | G | T | G | G | T | A | A | A |  | A | T | G | T | C | T | T | C | T | C | G | C | A | G | A | G | A | A | A | G | A | C | T | C | G | A | A | C | A | A | c | T | G | T | t | C | G | A | A | G | A | A | G | A | T | C | A | T | C | C | A | G | A | a | A | T | G | G | A | T | A | C | a | A | T | A | A | T | A | C | A | A | T | A | T | C | T | G | T | C | T | T | T | A | C | T | T | G | A | t | G | A | T | G | A | A | C | T | T | G | A | T | A | A | T | T | G | T | C | A | G | G | A | A | G | A | A | A | A | G | T | T | A | C | A | G | G | T | A | C | T | t | G | T | A | G | C | A | A | A | A | G | A | a | A | T |  |  |  |  |  |  |  |

|  |  |  |  |  |  |  |  |  |  |  |  |  |  |  |  |  |  |  |  |  |  |  |  |  |  |  |  |  |  |  |  |  |  |  |  |  |  |  |  |  |  |  |  |  |  |  |  |  |  |  |  |  |  |  |  |  |  |  |  |  |  |  |  |  |  |  |  |  |  |  |  |  |  |  |  |  |  |  |  |  |  |  |  |  |  |  |  |  |  |  |  |  |  |  |  |  |  |  |  |  |  |  |  |  |  |  |  |  |  |  |  |  |  |  |  |  |  |  |  |  |  |  |  |  |  |  |  |  |  |  |  |  |  |  |  |  |  |  |  |  |  |  |  |  |  |  |  |  |  |  |  |  |  |  |  |  |  |  |  |  |  |  |  |  |  |  |  |  |  |  |  |  |  |  |  |  |  |  |  |  |  |  |  |  |  |  |  |  |
| --- | --- | --- | --- | --- | --- | --- | --- | --- | --- | --- | --- | --- | --- | --- | --- | --- | --- | --- | --- | --- | --- | --- | --- | --- | --- | --- | --- | --- | --- | --- | --- | --- | --- | --- | --- | --- | --- | --- | --- | --- | --- | --- | --- | --- | --- | --- | --- | --- | --- | --- | --- | --- | --- | --- | --- | --- | --- | --- | --- | --- | --- | --- | --- | --- | --- | --- | --- | --- | --- | --- | --- | --- | --- | --- | --- | --- | --- | --- | --- | --- | --- | --- | --- | --- | --- | --- | --- | --- | --- | --- | --- | --- | --- | --- | --- | --- | --- | --- | --- | --- | --- | --- | --- | --- | --- | --- | --- | --- | --- | --- | --- | --- | --- | --- | --- | --- | --- | --- | --- | --- | --- | --- | --- | --- | --- | --- | --- | --- | --- | --- | --- | --- | --- | --- | --- | --- | --- | --- | --- | --- | --- | --- | --- | --- | --- | --- | --- | --- | --- | --- | --- | --- | --- | --- | --- | --- | --- | --- | --- | --- | --- | --- | --- | --- | --- | --- | --- | --- | --- | --- | --- | --- | --- | --- | --- | --- | --- | --- | --- | --- | --- | --- | --- | --- | --- | --- | --- | --- |
|  |  |  |  |  |  |  |  |  |  |  |  |  |  |  |  |  |  |  |  |  |  |  |  |  |  |  |  |  |  |  |  |  |  |  |  |  |  |  |  |  |  |  |  |  |  |  |  |  |  |  |  |  |  |  |  |  |  |  |  |  |  |  |  |  |  |  |  |  |  |  |  |  |  |  |  |  |  |  |  |  |  |  |  |  |  |  |  |  |  |  |  |  |  |  |  |  |  |  |  |  |  |  |  |  |  |  |  |  |  |  |  |  |  |  |  |  |  |  |  |  |  |  |  |  |  |  |  |  |  |  |  |  |  |  |  |  |  |  |  |  |  |  |  |  |  |  |  |  |  |  |  |  |  |  |  |  |  |  |  |  |  |  |  |  |  |  |  |  |  |  |  |  |  |  |  |  |  |  |  |  |  |  |  |  |  |  |  |  |
|  |  |  |  |  |  |  |  |  |  |  |  |  |  |  | 2 | 0 | 6 | 0 |  |  |  |  |  |  |  |  |  | \* |  |  |  |  |  |  | 2 | 0 | 8 | 0 |  |  |  |  |  |  |  |  |  | \* |  |  |  |  |  |  | 2 | 1 | 0 | 0 |  |  |  |  |  |  |  |  |  | \* |  |  |  |  |  |  | 2 | 1 | 2 | 0 |  |  |  |  |  |  |  |  |  | \* |  |  |  |  |  |  | 2 | 1 | 4 | 0 |  |  |  |  |  |  |  |  |  | \* |  |  |  |  |  |  | 2 | 1 | 6 | 0 |  |  |  |  |  |  |  |  |  | \* |  |  |  |  |  |  | 2 | 1 | 8 | 0 |  |  |  |  |  |  |  |  |  | \* |  |  |  |  |  |  | 2 | 2 | 0 | 0 |  |  |  |  |  |  |  |  |  | \* |  |  |  |  |  |  | 2 | 2 | 2 | 0 |  |  |  |  |  |  |  |  |  |  |
| M | N | 9 | 5 | 6 | 5 | 2 | 0 |  | : |  | G | A | T | T | A | G | C | T | A | C | A | G | C | A | G | C | A | G | C | A | G | T | G | A | A | G | A | T | G | A | A | G | C | T | G | C | A | A | A | T | G | T | C | A | A | A | G | C | T | A | A | C | A | T | T | A | A | G | G | A | A | G | A | A | G | A | C | G | A | A | G | A | C | A | C | C | T | A | T | C | G | T | C | C | G | A | A | C | C | G | A | A | A | G | C | G | A | A | G | A | G | G | A | A | C | A | A | G | T | T | C | C | T | C | T | C | A | G | C | C | A | A | A | T | T | A | T | A | C | A | A | G | G | T | A | T | G | A | T | A | T | T | C | C | T | T | C | A | G | A | A | T | A | C | A | T | T | C | C | A | A | A | T | A | A | A | A | A | G | A | C |  | : |  | 2 | 2 | 2 | 0 |
| M | T | 0 | 1 | 2 | 7 | 3 | 2 |  | : |  | G | A | T | T | A | G | C | T | A | C | A | G | C | A | G | C | A | G | C | A | G | T | G | A | A | G | A | T | G | A | A | G | C | T | G | C | A | A | A | T | G | T | C | A | A | A | G | C | T | A | A | C | A | T | T | A | A | G | G | A | A | G | A | A | G | A | C | G | A | A | G | A | C | A | C | C | T | A | T | C | G | T | C | C | G | A | A | C | C | G | A | A | A | G | C | G | A | A | G | A | G | G | A | A | C | A | A | G | C | T | C | C | T | C | T | C | A | G | C | C | A | A | A | T | T | A | T | A | C | A | A | G | G | T | A | T | G | A | T | A | T | T | C | C | T | T | C | A | G | A | A | T | A | C | A | T | T | C | C | A | A | A | C | A | A | A | A | A | G | A | C |  | : |  | 2 | 2 | 2 | 0 |
| M | T | 0 | 1 | 2 | 7 | 3 | 4 |  | : |  | G | A | T | T | A | G | C | T | A | C | A | G | C | A | G | C | A | G | C | A | G | T | G | A | A | G | A | T | G | A | A | G | C | T | G | C | A | A | A | T | G | T | C | A | A | A | G | C | T | A | A | C | A | T | T | A | A | G | G | A | A | G | A | A | G | A | C | G | A | A | G | A | C | A | C | C | T | A | T | C | G | T | C | C | G | A | A | C | C | G | A | A | A | G | C | G | A | A | G | A | G | G | A | A | C | A | A | A | C | T | C | C | T | C | T | C | A | G | C | C | A | A | A | T | T | A | T | A | C | A | A | G | G | T | A | T | G | A | T | A | T | T | C | C | T | T | C | C | G | A | A | T | A | C | A | T | T | C | C | A | A | A | C | A | A | A | A | A | G | A | C |  | : |  | 2 | 2 | 2 | 0 |
| M | T | 0 | 2 | 7 | 0 | 0 | 6 |  | : |  | G | A | T | T | A | G | C | T | A | C | A | G | C | A | G | C | A | G | C | A | G | T | G | A | A | G | A | T | G | A | A | G | C | T | G | C | A | A | A | T | G | T | C | A | A | A | G | C | T | A | A | C | A | T | T | A | A | G | G | A | A | G | A | A | G | A | C | G | A | A | G | A | C | A | C | C | T | A | T | C | G | T | C | C | G | A | A | C | C | G | A | A | A | G | C | G | A | A | G | A | G | G | A | A | C | A | A | G | C | T | C | C | T | C | T | C | A | G | C | C | A | A | A | T | T | A | T | A | C | A | A | G | G | T | A | T | G | A | T | A | T | T | C | C | T | T | C | A | G | A | A | T | A | C | A | T | T | C | C | A | A | A | C | A | A | A | A | A | G | A | C |  | : |  | 2 | 2 | 2 | 0 |
| M | T | 0 | 2 | 7 | 0 | 0 | 7 |  | : |  | G | A | T | T | A | G | C | T | A | C | A | G | C | A | G | C | A | G | C | A | G | T | G | A | A | G | A | T | G | A | A | G | C | T | G | C | A | A | A | T | G | T | C | A | A | A | G | C | T | A | A | C | A | T | T | A | A | G | G | A | A | G | A | A | G | A | C | G | A | A | G | A | C | A | C | C | T | A | T | C | G | T | C | C | G | A | A | C | C | G | A | A | A | G | C | G | A | A | G | A | G | G | A | A | C | A | A | G | T | T | C | C | T | C | T | C | A | G | C | C | A | A | A | T | T | A | T | A | C | A | A | G | G | T | A | T | G | A | T | A | T | T | C | C | T | T | C | A | G | A | A | T | A | C | A | T | T | C | C | A | A | A | T | A | A | A | A | A | G | A | C |  | : |  | 2 | 2 | 2 | 3 |
| M | T | 0 | 3 | 6 | 0 | 5 | 3 |  | : |  | G | A | T | T | A | G | C | T | A | C | A | G | C | A | G | C | A | G | C | A | G | T | G | A | A | G | A | T | G | A | A | G | C | T | G | C | A | A | A | T | G | T | C | A | A | A | G | C | T | A | A | C | A | T | T | A | A | G | G | A | A | G | A | A | G | A | C | G | A | A | G | A | C | A | C | C | T | A | T | C | G | T | C | C | G | A | A | C | C | G | A | A | A | G | C | G | A | A | G | A | G | G | A | A | C | A | A | G | T | T | C | C | T | C | T | C | A | G | C | C | A | A | A | T | T | A | T | A | C | A | A | G | G | T | A | T | G | A | T | A | T | T | C | C | T | T | C | A | G | A | A | T | A | C | A | T | T | C | C | A | A | A | T | A | A | A | A | A | G | A | C |  | : |  | 2 | 2 | 2 | 3 |
| M | T | 0 | 3 | 6 | 0 | 5 | 4 |  | : |  | G | A | T | T | A | G | C | T | A | C | A | G | C | A | G | C | A | G | C | A | G | T | G | A | A | G | A | T | G | A | A | G | C | T | G | C | A | A | A | T | G | T | C | A | A | A | G | C | T | A | A | C | A | T | T | A | A | G | G | A | A | G | A | A | G | A | C | G | A | A | G | A | C | A | C | C | T | A | T | C | G | T | C | C | G | A | A | C | C | G | A | A | A | G | C | G | A | A | G | A | G | G | A | A | C | A | A | G | T | T | C | C | T | C | T | C | A | G | C | C | A | A | A | T | T | A | T | A | C | A | A | G | G | T | A | T | G | A | T | A | T | T | C | C | T | T | C | A | G | A | A | T | A | C | A | T | T | C | C | A | A | A | T | A | A | A | A | A | G | A | C |  | : |  | 2 | 2 | 2 | 3 |
| M | T | 0 | 3 | 6 | 0 | 5 | 5 |  | : |  | G | A | T | T | A | G | C | T | A | C | A | G | C | A | G | C | A | G | C | G | A | A | G | A | T | G | A | A | G | A | A | G | C | T | G | C | A | A | A | T | G | T | C | A | A | A | G | C | T | A | A | C | A | T | C | A | A | G | G | A | A | G | A | A | G | A | C | G | A | A | G | A | T | A | C | C | T | A | T | C | G | T | C | C | G | A | A | C | C | G | A | A | A | G | C | G | A | A | G | A | G | G | A | A | C | A | A | G | C | T | C | C | T | C | T | C | A | G | C | C | A | A | A | T | T | A | T | A | C | A | A | G | G | T | A | T | G | A | T | A | T | T | C | C | T | T | C | A | G | A | A | T | A | C | A | T | T | C | C | A | A | A | T | A | A | A | A | A | G | A | C |  | : |  | 2 | 2 | 2 | 3 |
| M | T | 0 | 3 | 6 | 0 | 5 | 6 |  | : |  | G | A | T | T | A | G | C | T | A | C | A | G | C | A | G | C | A | G | C | A | G | T | G | A | A | A | A | T | G | A | A | G | C | T | G | C | A | A | A | T | G | T | C | A | A | A | G | C | T | A | A | C | A | T | T | A | A | G | G | A | A | G | A | A | G | A | C | G | A | A | G | A | C | A | C | C | T | A | T | C | G | T | C | C | G | A | A | C | C | G | A | A | A | G | C | G | A | A | G | G | G | G | A | A | C | A | A | G | T | T | C | C | T | C | T | C | G | G | C | C | A | A | A | T | T | A | T | A | C | A | A | G | G | T | A | T | G | A | T | A | T | T | C | C | T | T | C | A | G | A | A | T | A | C | A | T | T | C | C | A | A | A | T | A | A | A | A | A | G | A | C |  | : |  | 2 | 2 | 2 | 0 |
| M | T | 0 | 3 | 6 | 0 | 5 | 7 |  | : |  | G | A | T | T | A | G | C | T | A | C | A | G | C | A | G | C | A | G | C | A | G | T | G | A | A | G | A | T | G | A | A | G | C | T | G | C | A | A | A | T | G | T | C | A | A | A | G | C | T | A | A | C | A | T | T | A | A | G | G | A | A | G | A | A | G | A | C | G | A | A | G | A | C | A | C | C | T | A | T | C | G | T | C | C | G | A | A | C | C | G | A | A | A | G | C | G | A | A | G | A | G | G | A | A | C | A | A | G | C | T | C | C | T | C | T | C | A | G | C | C | A | A | A | T | T | A | T | A | C | A | A | G | G | T | A | T | G | A | T | A | T | T | C | C | T | T | C | A | G | A | A | T | A | C | A | T | T | C | C | A | A | A | C | A | A | A | A | A | G | A | C |  | : |  | 2 | 2 | 2 | 0 |
| K | X | 2 | 4 | 9 | 7 | 3 | 8 |  | : |  | G | A | T | T | A | G | C | T | A | C | A | G | C | A | G | C | A | G | C | A | G | T | G | A | A | G | A | T | G | A | A | G | C | T | G | C | A | A | A | T | G | T | C | A | A | G | G | C | T | A | A | C | A | T | T | A | A | G | G | A | A | G | A | A | G | A | C | G | A | A | G | A | C | A | C | C | T | A | T | C | G | T | C | C | G | A | A | C | C | G | A | A | A | G | C | G | A | A | G | A | G | G | A | A | C | A | A | G | C | T | C | C | T | C | T | C | A | G | C | C | A | A | A | T | T | A | T | A | C | A | A | G | G | T | A | T | G | A | T | A | T | T | C | C | T | T | C | A | G | A | A | T | A | C | A | T | T | C | C | A | A | A | C | A | A | A | A | A | G | A | C |  | : |  | 2 | 2 | 2 | 0 |
| K | X | 2 | 4 | 9 | 7 | 3 | 7 |  | : |  | G | A | T | T | A | G | C | T | A | C | A | G | C | A | G | C | A | G | C | A | G | T | G | A | A | G | A | T | G | A | A | G | C | T | G | C | A | A | A | T | G | T | C | A | A | G | G | C | T | A | A | C | A | T | T | A | A | G | G | A | A | G | A | A | G | A | C | G | A | A | G | A | C | A | C | C | T | A | T | C | G | T | C | C | G | A | A | C | C | G | A | A | A | G | C | G | A | A | G | A | G | G | A | A | C | A | A | G | C | T | C | C | T | C | T | C | A | G | C | C | A | A | A | T | T | A | T | A | C | A | A | G | G | T | A | T | G | A | T | A | T | T | C | C | T | T | C | A | G | A | A | T | A | C | A | T | T | C | C | A | A | A | C | A | A | A | A | A | G | A | C |  | : |  | 2 | 2 | 2 | 0 |
| K | X | 2 | 4 | 9 | 7 | 3 | 6 |  | : |  | G | A | T | T | A | G | C | T | A | C | A | G | C | A | G | C | A | G | C | A | G | T | G | A | A | G | A | T | G | A | A | G | C | T | G | C | A | A | A | T | G | T | C | A | A | G | G | C | T | A | A | C | A | T | T | A | A | G | G | A | A | G | A | A | G | A | C | G | A | A | G | A | C | A | C | C | T | A | T | C | G | T | C | C | G | A | A | C | C | G | A | A | A | G | C | G | A | A | G | A | G | G | A | A | C | A | A | G | C | T | C | C | T | C | T | C | A | G | C | C | A | A | A | T | T | A | T | A | C | A | A | G | G | T | A | T | G | A | T | A | T | T | C | C | T | T | C | A | G | A | A | T | A | C | A | T | T | C | C | A | A | A | C | A | A | A | A | A | G | A | C |  | : |  | 2 | 2 | 2 | 0 |
| K | X | 2 | 4 | 9 | 7 | 3 | 5 |  | : |  | G | A | T | T | A | G | C | T | A | C | A | G | C | A | G | C | A | G | C | A | G | T | G | A | A | G | A | T | G | A | A | G | C | T | G | C | A | A | A | T | G | T | C | A | A | A | G | C | T | A | A | C | A | T | T | A | A | G | G | A | A | G | A | A | G | A | C | G | A | A | G | A | C | A | C | C | T | A | T | C | G | T | C | C | G | A | A | C | C | G | A | A | A | G | C | G | A | A | G | A | G | G | A | A | C | A | A | G | C | T | C | C | T | C | T | C | A | G | C | C | A | A | A | T | T | A | T | A | C | A | A | G | G | T | A | T | G | A | T | A | T | T | C | C | T | T | C | A | G | A | A | T | A | C | A | T | T | C | C | A | A | A | C | A | A | A | A | A | G | A | C |  | : |  | 2 | 2 | 2 | 0 |
| M | F | 1 | 9 | 7 | 9 | 1 | 6 |  | : |  | G | A | T | T | A | G | C | T | A | C | A | G | C | A | G | C | A | G | C | A | G | T | G | A | A | G | A | T | G | A | A | G | C | T | G | C | A | A | A | T | G | T | C | A | A | A | G | C | T | A | A | C | A | T | T | A | A | G | G | A | A | G | A | A | G | A | C | G | A | A | G | A | C | A | C | C | T | A | T | C | G | T | C | C | G | A | A | C | C | G | A | A | A | G | C | G | A | A | G | A | G | G | A | A | C | A | A | G | C | T | C | C | T | C | T | C | A | G | C | C | A | A | A | T | T | A | T | A | C | A | A | G | G | T | A | T | G | A | T | A | T | T | C | C | T | T | C | A | G | A | A | T | A | C | A | T | T | C | C | A | A | A | C | A | A | A | A | A | G | A | C |  | : |  | 2 | 2 | 2 | 3 |
| K | T | 2 | 5 | 0 | 6 | 3 | 2 |  | : |  | G | A | T | T | A | G | C | T | A | C | A | G | C | A | G | C | A | G | C | A | G | T | G | A | A | G | A | T | G | A | A | G | C | T | G | C | A | A | A | T | G | T | C | A | A | A | G | C | T | A | A | C | A | T | T | A | A | G | G | A | A | G | A | A | G | A | C | G | A | A | G | A | C | A | C | C | T | A | T | C | G | T | C | C | G | A | A | C | C | G | A | A | A | G | C | G | A | A | G | A | G | G | A | A | C | A | A | G | T | T | C | C | T | C | T | C | A | G | C | C | A | A | A | T | T | A | T | A | C | A | A | G | G | T | A | T | G | A | T | A | T | T | C | C | T | T | C | A | G | A | A | T | A | C | A | T | T | C | C | A | A | A | T | A | A | A | A | A | G | A | C |  | : |  | 2 | 2 | 2 | 3 |
|  |  |  |  |  |  |  |  |  |  |  | G | A | T | T | A | G | C | T | A | C | A | G | C | A | G | C | A | G | C | a | g | t | G | A | a | g | A | t | G | A | A | G | C | T | G | C | A | A | A | T | G | T | C | A | A | a | G | C | T | A | A | C | A | T | t | A | A | G | G | A | A | G | A | A | G | A | C | G | A | A | G | A | c | A | C | C | T | A | T | C | G | T | C | C | G | A | A | C | C | G | A | A | A | G | C | G | A | A | G | a | G | G | A | A | C | A | A | g |  | T | C | C | T | C | T | C | a | G | C | C | A | A | A | T | T | A | T | A | C | A | A | G | G | T | A | T | G | A | T | A | T | T | C | C | T | T | C | a | G | A | A | T | A | C | A | T | T | C | C | A | A | A |  | A | A | A | A | A | G | A | C |  |  |  |  |  |  |  |

|  |  |  |  |  |  |  |  |  |  |  |  |  |  |  |  |  |  |  |  |  |  |  |  |  |  |  |  |  |  |  |  |  |  |  |  |  |  |  |  |  |  |  |  |  |  |  |  |  |  |  |  |  |  |  |  |  |  |  |  |  |  |  |  |  |  |  |  |  |  |  |  |  |  |  |  |  |  |  |  |  |  |  |  |  |  |  |  |  |  |  |  |  |  |  |  |  |  |  |  |  |  |  |  |  |  |  |  |  |  |  |  |  |  |  |  |  |  |  |  |  |  |  |  |  |  |  |  |  |  |  |  |  |  |  |  |  |  |  |  |  |  |  |  |  |  |  |  |  |  |  |  |  |  |  |  |  |  |  |  |  |  |  |  |  |  |  |  |  |  |  |  |  |  |  |  |  |  |  |  |  |  |  |  |  |  |  |  |  |
| --- | --- | --- | --- | --- | --- | --- | --- | --- | --- | --- | --- | --- | --- | --- | --- | --- | --- | --- | --- | --- | --- | --- | --- | --- | --- | --- | --- | --- | --- | --- | --- | --- | --- | --- | --- | --- | --- | --- | --- | --- | --- | --- | --- | --- | --- | --- | --- | --- | --- | --- | --- | --- | --- | --- | --- | --- | --- | --- | --- | --- | --- | --- | --- | --- | --- | --- | --- | --- | --- | --- | --- | --- | --- | --- | --- | --- | --- | --- | --- | --- | --- | --- | --- | --- | --- | --- | --- | --- | --- | --- | --- | --- | --- | --- | --- | --- | --- | --- | --- | --- | --- | --- | --- | --- | --- | --- | --- | --- | --- | --- | --- | --- | --- | --- | --- | --- | --- | --- | --- | --- | --- | --- | --- | --- | --- | --- | --- | --- | --- | --- | --- | --- | --- | --- | --- | --- | --- | --- | --- | --- | --- | --- | --- | --- | --- | --- | --- | --- | --- | --- | --- | --- | --- | --- | --- | --- | --- | --- | --- | --- | --- | --- | --- | --- | --- | --- | --- | --- | --- | --- | --- | --- | --- | --- | --- | --- | --- | --- | --- | --- | --- | --- | --- | --- | --- | --- | --- | --- |
|  |  |  |  |  |  |  |  |  |  |  |  |  |  |  |  |  |  |  |  |  |  |  |  |  |  |  |  |  |  |  |  |  |  |  |  |  |  |  |  |  |  |  |  |  |  |  |  |  |  |  |  |  |  |  |  |  |  |  |  |  |  |  |  |  |  |  |  |  |  |  |  |  |  |  |  |  |  |  |  |  |  |  |  |  |  |  |  |  |  |  |  |  |  |  |  |  |  |  |  |  |  |  |  |  |  |  |  |  |  |  |  |  |  |  |  |  |  |  |  |  |  |  |  |  |  |  |  |  |  |  |  |  |  |  |  |  |  |  |  |  |  |  |  |  |  |  |  |  |  |  |  |  |  |  |  |  |  |  |  |  |  |  |  |  |  |  |  |  |  |  |  |  |  |  |  |  |  |  |  |  |  |  |  |  |  |  |  |  |
|  |  |  |  |  |  |  |  |  |  |  |  |  |  |  |  |  | \* |  |  |  |  |  |  | 2 | 2 | 4 | 0 |  |  |  |  |  |  |  |  |  | \* |  |  |  |  |  |  | 2 | 2 | 6 | 0 |  |  |  |  |  |  |  |  |  | \* |  |  |  |  |  |  | 2 | 2 | 8 | 0 |  |  |  |  |  |  |  |  |  | \* |  |  |  |  |  |  | 2 | 3 | 0 | 0 |  |  |  |  |  |  |  |  |  | \* |  |  |  |  |  |  | 2 | 3 | 2 | 0 |  |  |  |  |  |  |  |  |  | \* |  |  |  |  |  |  | 2 | 3 | 4 | 0 |  |  |  |  |  |  |  |  |  | \* |  |  |  |  |  |  | 2 | 3 | 6 | 0 |  |  |  |  |  |  |  |  |  | \* |  |  |  |  |  |  | 2 | 3 | 8 | 0 |  |  |  |  |  |  |  |  |  | \* |  |  |  |  |  |  |  |  |  |  |  |
| M | N | 9 | 5 | 6 | 5 | 2 | 0 |  | : |  | A | G | G | T | C | T | A | A | C | A | A | A | C | A | C | C | A | A | A | A | G | T | T | T | G | G | A | T | C | T | G | G | A | T | T | G | C | G | C | A | A | G | C | A | A | C | C | G | A | A | G | A | C | A | A | C | T | C | A | T | T | G | A | A | G | A | A | T | G | G | G | A | C | A | A | T | G | A | G | A | T | G | A | G | A | T | T | A | A | T | C | A | T | T | A | A | A | A | C | A | G | A | G | A | A | G | G | C | T | C | T | T | A | C | A | A | A | T | G | A | T | T | T | T | G | A | T | C | T | T | A | T | C | C | T | T | A | C | T | C | T | C | G | C | A | A | A | G | A | G | T | A | A | A | A | C | A | G | T | T | G | G | C | A | A | C | G | C | C | A | A |  | : |  | 2 | 3 | 9 | 1 |
| M | T | 0 | 1 | 2 | 7 | 3 | 2 |  | : |  | A | G | G | T | C | T | A | A | C | A | A | A | C | A | C | C | A | A | A | A | G | T | T | T | G | G | A | T | C | T | G | G | A | T | T | G | C | G | C | A | A | G | C | A | A | C | C | G | A | A | G | A | C | A | A | C | T | C | A | T | T | G | A | A | G | A | A | T | G | G | G | A | C | A | A | T | G | A | G | A | T | G | A | G | A | C | T | A | A | T | C | A | T | T | A | A | A | A | C | A | G | A | G | A | A | G | G | C | T | C | T | T | A | C | A | A | A | T | G | A | T | T | T | T | G | A | T | C | T | T | A | T | C | C | T | C | A | C | T | C | T | T | G | C | A | A | A | G | A | G | T | A | A | A | A | C | A | G | T | T | G | G | C | A | A | C | G | C | C | A | A |  | : |  | 2 | 3 | 9 | 1 |
| M | T | 0 | 1 | 2 | 7 | 3 | 4 |  | : |  | A | G | G | T | C | T | A | A | C | A | A | A | C | A | C | C | A | A | A | A | G | T | T | T | G | G | A | T | C | T | G | G | A | T | T | G | C | G | C | A | A | G | C | A | A | C | C | G | A | A | G | A | C | A | A | C | T | C | A | T | T | G | A | A | G | A | A | T | G | G | G | A | C | A | A | T | G | A | G | A | T | G | A | G | A | C | T | A | A | T | C | A | T | T | A | A | A | A | C | A | G | A | G | A | A | G | G | C | T | C | T | T | A | C | A | A | A | T | G | A | T | T | T | T | G | A | T | C | T | T | A | T | C | C | T | T | A | C | T | C | T | C | G | C | A | A | A | G | A | G | T | A | A | A | A | C | A | G | T | T | G | G | C | A | A | C | G | C | C | A | A |  | : |  | 2 | 3 | 9 | 1 |
| M | T | 0 | 2 | 7 | 0 | 0 | 6 |  | : |  | A | G | G | T | C | T | A | A | C | A | A | A | C | A | C | C | A | A | A | A | G | T | T | T | G | G | A | T | C | T | G | G | A | T | T | G | C | G | C | A | A | G | C | A | A | C | C | G | A | A | G | A | C | A | A | C | T | C | A | T | T | G | A | A | G | A | A | T | G | G | G | A | C | A | A | T | G | A | G | A | T | G | A | G | A | C | T | A | A | T | C | A | T | T | A | A | A | A | C | A | G | A | G | A | A | G | G | C | T | C | T | T | A | C | A | A | A | T | G | A | T | T | T | T | G | A | T | C | T | T | A | T | C | C | T | T | A | C | T | C | T | C | G | C | A | A | A | G | A | G | T | A | A | A | A | C | A | G | T | T | G | G | C | A | A | C | G | C | C | A | A |  | : |  | 2 | 3 | 9 | 1 |
| M | T | 0 | 2 | 7 | 0 | 0 | 7 |  | : |  | A | G | G | T | C | T | A | A | C | A | A | A | C | A | C | C | A | A | A | A | G | T | T | T | G | G | A | T | C | T | G | G | A | T | T | G | C | G | C | A | A | G | C | A | A | C | C | G | A | A | G | A | C | A | A | C | T | C | A | T | T | G | A | A | G | A | A | T | G | G | G | A | C | A | A | T | G | A | G | A | T | G | A | G | A | T | T | A | A | T | C | A | T | T | A | A | A | A | C | A | G | A | G | A | A | G | G | C | T | C | T | T | A | C | A | A | A | T | G | A | T | T | T | T | G | A | T | C | T | T | A | T | C | C | T | T | A | C | T | C | T | C | G | C | A | A | A | G | A | G | T | A | A | A | A | C | A | G | T | T | G | G | C | A | A | T | G | C | C | A | A |  | : |  | 2 | 3 | 9 | 4 |
| M | T | 0 | 3 | 6 | 0 | 5 | 3 |  | : |  | A | G | G | T | C | T | A | A | C | A | A | A | C | A | C | C | A | A | A | A | G | T | T | T | G | G | A | T | C | T | G | G | A | T | T | G | C | G | C | A | A | G | C | A | A | C | C | G | A | A | G | A | C | A | A | C | T | C | A | T | T | G | A | A | G | A | A | T | G | G | G | A | C | A | A | T | G | A | G | A | T | G | A | G | A | T | T | A | A | T | C | A | T | T | A | A | A | A | C | A | G | A | G | A | A | G | G | C | T | C | T | T | A | C | A | A | A | T | G | A | T | T | T | T | G | A | T | C | T | T | A | T | C | C | T | T | A | C | T | C | T | C | G | C | A | A | A | G | A | G | T | A | A | A | A | C | A | G | T | T | G | G | C | A | A | T | G | C | C | A | A |  | : |  | 2 | 3 | 9 | 4 |
| M | T | 0 | 3 | 6 | 0 | 5 | 4 |  | : |  | A | G | G | T | C | T | A | A | C | A | A | A | C | A | C | C | A | A | A | A | G | T | T | T | G | G | A | T | C | T | G | G | A | T | T | G | C | G | C | A | A | G | C | A | A | C | C | G | A | A | G | A | C | A | A | C | T | C | A | T | T | G | A | A | G | A | A | T | G | G | G | A | C | A | A | T | G | A | G | A | T | G | A | G | A | T | T | A | A | T | C | A | T | T | A | A | A | A | C | A | G | A | G | A | A | G | G | C | T | C | T | T | A | C | A | A | A | T | G | A | T | T | T | T | G | A | T | C | T | T | A | T | C | C | T | T | A | C | T | C | T | C | G | C | A | A | A | G | A | G | T | A | A | A | A | C | A | G | T | T | G | G | C | A | A | T | G | C | C | A | A |  | : |  | 2 | 3 | 9 | 4 |
| M | T | 0 | 3 | 6 | 0 | 5 | 5 |  | : |  | A | G | G | T | C | T | A | A | C | A | A | A | C | A | C | C | A | A | A | A | G | T | T | T | G | G | A | T | C | T | G | G | A | T | T | G | T | G | C | A | A | G | C | A | A | C | C | G | A | A | G | A | C | A | A | C | T | C | A | T | T | G | A | A | G | A | A | T | G | G | G | A | C | A | A | T | G | A | G | A | T | G | A | G | A | T | T | A | A | T | C | A | T | T | A | A | A | A | C | A | G | A | G | A | A | G | G | C | T | C | T | T | A | C | A | A | A | T | G | A | T | T | T | T | G | A | T | C | T | T | A | T | C | C | T | T | A | C | T | C | T | C | G | C | A | A | A | G | A | G | T | A | A | A | A | C | A | G | T | T | G | G | C | A | A | T | G | C | C | A | A |  | : |  | 2 | 3 | 9 | 4 |
| M | T | 0 | 3 | 6 | 0 | 5 | 6 |  | : |  | A | G | G | T | C | T | A | A | C | A | A | A | C | A | C | C | A | A | A | A | G | T | T | T | G | G | A | T | C | T | G | G | A | T | T | G | C | G | C | A | A | G | C | A | A | C | C | G | A | A | G | A | C | A | A | C | T | C | A | T | T | G | A | A | G | A | A | T | G | G | G | A | C | A | A | T | G | A | G | A | T | G | A | G | A | T | T | A | A | T | C | A | T | T | A | A | A | A | C | A | G | A | G | A | A | G | G | C | T | C | T | T | A | C | A | A | A | T | G | A | T | T | T | T | G | A | T | C | T | T | A | T | C | C | T | T | A | C | T | C | T | C | G | C | A | A | A | G | A | G | T | A | A | A | A | C | A | G | T | T | G | G | C | A | A | T | G | C | C | A | A |  | : |  | 2 | 3 | 9 | 1 |
| M | T | 0 | 3 | 6 | 0 | 5 | 7 |  | : |  | A | G | G | T | C | T | A | A | C | A | A | A | C | A | C | C | A | A | A | A | G | T | T | T | G | G | A | T | C | T | G | G | A | T | T | G | C | G | C | A | A | G | C | A | A | C | C | G | A | A | G | A | C | A | A | C | T | C | A | T | T | G | A | A | G | A | A | T | G | G | G | A | C | A | A | T | G | A | G | A | T | G | A | G | A | T | T | A | A | T | C | A | T | T | A | A | A | A | C | A | G | G | G | A | A | G | G | C | T | C | T | T | A | C | A | A | A | T | G | A | T | T | T | T | G | A | T | C | T | T | A | T | C | C | T | T | A | C | T | C | T | C | G | C | A | A | A | G | A | G | T | A | A | A | A | C | A | G | T | T | G | G | C | A | A | C | G | C | C | A | A |  | : |  | 2 | 3 | 9 | 1 |
| K | X | 2 | 4 | 9 | 7 | 3 | 8 |  | : |  | A | G | G | T | C | T | A | A | C | A | A | A | C | A | C | C | A | A | A | A | G | T | T | T | G | G | A | T | C | T | G | G | A | T | T | G | C | G | C | A | A | G | C | A | A | C | C | G | A | A | G | A | C | A | A | C | T | C | A | T | T | G | A | A | G | A | A | T | G | G | G | A | C | A | A | T | G | A | G | A | T | G | A | G | A | C | T | A | A | T | C | A | T | T | A | A | A | A | C | A | G | A | G | A | A | G | G | C | T | C | T | T | A | C | A | A | A | T | G | A | T | T | T | T | G | A | T | C | T | T | A | T | C | C | T | T | A | C | T | C | T | C | G | C | A | A | A | G | A | G | T | A | A | A | A | C | A | G | T | T | G | G | C | A | A | C | G | C | C | A | A |  | : |  | 2 | 3 | 9 | 1 |
| K | X | 2 | 4 | 9 | 7 | 3 | 7 |  | : |  | A | G | G | T | C | T | A | A | C | A | A | A | C | A | C | C | A | A | A | A | G | T | T | T | G | G | A | T | C | T | G | G | A | T | T | G | C | G | C | A | A | G | C | A | A | C | C | G | A | A | G | A | C | A | A | C | T | C | A | T | T | G | A | A | G | A | A | T | G | G | G | A | C | A | A | T | G | A | G | A | T | G | A | G | A | C | T | A | A | T | C | A | T | T | A | A | A | A | C | A | G | A | G | A | A | G | G | C | T | C | T | T | A | C | A | A | A | T | G | A | T | T | T | T | G | A | T | C | T | T | A | T | C | C | T | T | A | C | T | C | T | C | G | C | A | A | A | G | A | G | T | A | A | A | A | C | A | G | T | T | G | G | C | A | A | C | G | C | C | A | A |  | : |  | 2 | 3 | 9 | 1 |
| K | X | 2 | 4 | 9 | 7 | 3 | 6 |  | : |  | A | G | G | T | C | T | A | A | C | A | A | A | C | A | C | C | A | A | A | A | G | T | T | T | G | G | A | T | C | T | G | G | A | T | T | G | C | G | C | A | A | G | C | A | A | C | C | G | A | A | G | A | C | A | A | C | T | C | A | T | T | G | A | A | G | A | A | T | G | G | G | A | C | A | A | T | G | A | G | A | T | G | A | G | A | C | T | A | A | T | C | A | T | T | A | A | A | A | C | A | G | A | G | A | A | G | G | C | T | C | T | T | A | C | A | A | A | T | G | A | T | T | T | T | G | A | T | C | T | T | A | T | C | C | T | T | A | C | T | C | T | C | G | C | A | A | A | G | A | G | T | A | A | A | A | C | A | G | T | T | G | G | C | A | A | C | G | C | C | A | A |  | : |  | 2 | 3 | 9 | 1 |
| K | X | 2 | 4 | 9 | 7 | 3 | 5 |  | : |  | A | G | G | T | C | T | A | A | C | A | A | A | C | A | C | C | A | A | A | A | G | T | T | T | G | G | A | T | C | T | G | G | A | T | T | G | C | G | C | A | A | G | C | A | G | C | C | G | A | A | G | A | C | A | A | C | T | C | A | T | T | G | A | A | G | A | A | T | G | G | G | A | C | A | A | T | G | A | G | A | T | G | A | G | A | C | T | A | A | T | C | A | T | T | A | A | A | A | C | A | G | A | G | A | A | G | G | C | T | C | T | T | A | C | A | A | A | T | G | A | T | T | T | T | G | A | T | C | T | T | A | T | C | C | T | T | A | C | T | C | T | C | G | C | A | A | A | G | A | G | T | A | A | A | A | C | A | G | T | T | G | G | C | A | A | C | G | C | C | A | A |  | : |  | 2 | 3 | 9 | 1 |
| M | F | 1 | 9 | 7 | 9 | 1 | 6 |  | : |  | A | G | G | T | C | T | A | A | C | A | A | A | C | A | C | C | A | A | A | A | G | T | T | T | A | G | A | T | C | T | G | G | A | T | T | G | T | G | C | A | A | G | C | A | A | C | C | G | A | A | G | A | C | A | A | C | T | C | A | T | T | G | A | A | G | A | A | T | G | G | G | A | C | A | A | T | G | A | G | A | T | G | A | G | A | C | T | A | A | T | C | A | T | T | A | A | A | A | C | A | G | A | G | A | A | G | G | C | T | C | T | T | A | C | A | A | A | T | G | A | T | T | T | T | G | A | T | C | T | T | A | T | C | C | T | T | A | C | T | C | T | C | G | C | A | A | A | G | A | G | T | A | A | A | A | C | A | G | T | T | G | G | C | A | A | T | G | C | C | A | A |  | : |  | 2 | 3 | 9 | 4 |
| K | T | 2 | 5 | 0 | 6 | 3 | 2 |  | : |  | A | G | G | T | C | T | A | A | C | A | A | A | C | A | C | C | A | A | A | A | G | T | T | T | G | G | A | T | C | T | G | G | A | T | T | G | C | G | C | A | A | G | C | A | A | C | C | G | A | A | G | A | C | A | A | C | T | C | A | T | T | G | A | A | G | A | A | T | G | G | G | A | C | A | A | T | G | A | G | A | T | G | A | G | A | T | T | A | A | T | C | A | T | T | A | A | A | A | C | A | G | A | G | A | A | G | G | C | T | C | T | T | A | C | A | A | A | T | G | A | T | T | T | T | G | A | T | C | T | T | A | T | C | C | T | T | A | C | T | C | T | C | G | C | A | A | A | G | A | G | T | A | A | A | A | C | A | G | T | T | G | G | C | A | A | T | G | C | C | A | A |  | : |  | 2 | 3 | 9 | 4 |
|  |  |  |  |  |  |  |  |  |  |  | A | G | G | T | C | T | A | A | C | A | A | A | C | A | C | C | A | A | A | A | G | T | T | T | g | G | A | T | C | T | G | G | A | T | T | G | c | G | C | A | A | G | C | A | a | C | C | G | A | A | G | A | C | A | A | C | T | C | A | T | T | G | A | A | G | A | A | T | G | G | G | A | C | A | A | T | G | A | G | A | T | G | A | G | A |  | T | A | A | T | C | A | T | T | A | A | A | A | C | A | G | a | G | A | A | G | G | C | T | C | T | T | A | C | A | A | A | T | G | A | T | T | T | T | G | A | T | C | T | T | A | T | C | C | T | t | A | C | T | C | T | c | G | C | A | A | A | G | A | G | T | A | A | A | A | C | A | G | T | T | G | G | C | A | A |  | G | C | C | A | A |  |  |  |  |  |  |  |

|  |  |  |  |  |  |  |  |  |  |  |  |  |  |  |  |  |  |  |  |  |  |  |  |  |  |  |  |  |  |  |  |  |  |  |  |  |  |  |  |  |  |  |  |  |  |  |  |  |  |  |  |  |  |  |  |  |  |  |  |  |  |  |  |  |  |  |  |  |  |  |  |  |  |  |  |  |  |  |  |  |  |  |  |  |  |  |  |  |  |  |  |  |  |  |  |  |  |  |  |  |  |  |  |  |  |  |  |  |  |  |  |  |  |  |  |  |  |  |  |  |  |  |  |  |  |  |  |  |  |  |  |  |  |  |  |  |  |  |  |  |  |  |  |  |  |  |  |  |  |  |  |  |  |  |  |  |  |  |  |  |  |  |  |  |  |  |  |  |  |  |  |  |  |  |  |  |  |  |  |  |  |  |  |  |  |  |  |  |
| --- | --- | --- | --- | --- | --- | --- | --- | --- | --- | --- | --- | --- | --- | --- | --- | --- | --- | --- | --- | --- | --- | --- | --- | --- | --- | --- | --- | --- | --- | --- | --- | --- | --- | --- | --- | --- | --- | --- | --- | --- | --- | --- | --- | --- | --- | --- | --- | --- | --- | --- | --- | --- | --- | --- | --- | --- | --- | --- | --- | --- | --- | --- | --- | --- | --- | --- | --- | --- | --- | --- | --- | --- | --- | --- | --- | --- | --- | --- | --- | --- | --- | --- | --- | --- | --- | --- | --- | --- | --- | --- | --- | --- | --- | --- | --- | --- | --- | --- | --- | --- | --- | --- | --- | --- | --- | --- | --- | --- | --- | --- | --- | --- | --- | --- | --- | --- | --- | --- | --- | --- | --- | --- | --- | --- | --- | --- | --- | --- | --- | --- | --- | --- | --- | --- | --- | --- | --- | --- | --- | --- | --- | --- | --- | --- | --- | --- | --- | --- | --- | --- | --- | --- | --- | --- | --- | --- | --- | --- | --- | --- | --- | --- | --- | --- | --- | --- | --- | --- | --- | --- | --- | --- | --- | --- | --- | --- | --- | --- | --- | --- | --- | --- | --- | --- | --- | --- | --- | --- |
|  |  |  |  |  |  |  |  |  |  |  |  |  |  |  |  |  |  |  |  |  |  |  |  |  |  |  |  |  |  |  |  |  |  |  |  |  |  |  |  |  |  |  |  |  |  |  |  |  |  |  |  |  |  |  |  |  |  |  |  |  |  |  |  |  |  |  |  |  |  |  |  |  |  |  |  |  |  |  |  |  |  |  |  |  |  |  |  |  |  |  |  |  |  |  |  |  |  |  |  |  |  |  |  |  |  |  |  |  |  |  |  |  |  |  |  |  |  |  |  |  |  |  |  |  |  |  |  |  |  |  |  |  |  |  |  |  |  |  |  |  |  |  |  |  |  |  |  |  |  |  |  |  |  |  |  |  |  |  |  |  |  |  |  |  |  |  |  |  |  |  |  |  |  |  |  |  |  |  |  |  |  |  |  |  |  |  |  |  |
|  |  |  |  |  |  |  |  |  |  |  |  |  | 2 | 4 | 0 | 0 |  |  |  |  |  |  |  |  |  | \* |  |  |  |  |  |  | 2 | 4 | 2 | 0 |  |  |  |  |  |  |  |  |  | \* |  |  |  |  |  |  | 2 | 4 | 4 | 0 |  |  |  |  |  |  |  |  |  | \* |  |  |  |  |  |  | 2 | 4 | 6 | 0 |  |  |  |  |  |  |  |  |  | \* |  |  |  |  |  |  | 2 | 4 | 8 | 0 |  |  |  |  |  |  |  |  |  | \* |  |  |  |  |  |  | 2 | 5 | 0 | 0 |  |  |  |  |  |  |  |  |  | \* |  |  |  |  |  |  | 2 | 5 | 2 | 0 |  |  |  |  |  |  |  |  |  | \* |  |  |  |  |  |  | 2 | 5 | 4 | 0 |  |  |  |  |  |  |  |  |  | \* |  |  |  |  |  |  | 2 | 5 | 6 | 0 |  |  |  |  |  |  |  |  |  |  |  |  |
| M | N | 9 | 5 | 6 | 5 | 2 | 0 |  | : |  | A | C | A | A | T | T | G | T | T | G | G | A | A | T | C | A | C | T | T | C | A | C | A | C | T | G | A | T | G | C | C | T | T | C | A | A | A | C | A | A | G | C | T | T | C | T | T | C | A | A | C | A | G | G | A | G | A | A | G | A | A | T | T | T | T | T | G | A | C | A | A | C | T | C | T | G | A | C | A | A | A | T | T | C | G | T | T | T | T | A | C | A | C | T | A | T | A | T | T | T | G | T | T | G | G | A | A | C | T | A | A | T | T | A | T | C | T | T | A | C | A | C | A | G | G | G | A | A | C | T | C | G | T | G | A | A | A | A | G | G | A | G | A | A | A | G | C | T | G | T | G | C | A | A | G | A | A | G | C | T | A | G | A | A | A | C | A | G | G | T | T |  | : |  | 2 | 5 | 6 | 2 |
| M | T | 0 | 1 | 2 | 7 | 3 | 2 |  | : |  | A | C | A | A | T | T | G | T | T | G | G | A | A | T | C | A | C | T | T | C | A | C | A | C | T | G | A | T | G | C | C | T | T | C | A | A | A | C | A | A | G | C | T | T | C | T | T | C | A | A | C | A | G | G | A | G | A | A | G | A | A | T | T | T | T | T | G | A | C | A | A | C | T | C | T | G | A | C | A | A | A | T | T | C | G | T | T | T | T | A | C | A | C | T | A | T | A | T | T | T | G | T | T | G | G | A | A | C | T | A | A | C | T | A | T | C | T | T | A | C | A | C | A | G | G | G | A | A | C | T | C | G | T | G | A | A | A | A | G | G | A | G | A | A | A | G | C | T | G | T | G | C | A | A | G | A | A | G | C | T | A | G | A | A | A | C | A | G | G | T | T |  | : |  | 2 | 5 | 6 | 2 |
| M | T | 0 | 1 | 2 | 7 | 3 | 4 |  | : |  | A | C | A | A | T | T | G | T | T | G | G | A | A | T | C | A | C | T | T | C | A | C | A | C | T | G | A | T | G | C | C | T | T | C | A | A | A | C | A | A | G | C | T | T | C | T | T | C | A | A | C | A | G | G | A | G | A | A | G | A | A | T | T | T | T | T | G | A | C | A | A | C | T | C | T | G | A | C | A | A | A | T | T | C | G | T | T | T | T | A | C | A | C | T | A | T | A | T | T | T | G | T | T | G | G | A | A | C | T | A | A | C | T | A | T | C | T | T | A | C | A | C | A | G | G | G | A | A | C | T | C | G | T | G | A | A | A | A | G | G | A | G | A | A | A | G | C | T | G | T | G | C | A | A | G | A | A | G | C | T | A | G | A | A | A | C | A | G | G | T | T |  | : |  | 2 | 5 | 6 | 2 |
| M | T | 0 | 2 | 7 | 0 | 0 | 6 |  | : |  | A | C | A | A | T | T | G | T | T | G | G | A | A | T | C | A | C | T | T | C | A | C | A | C | T | G | A | T | G | C | C | T | T | C | A | A | A | C | A | A | G | C | T | T | C | T | T | C | A | A | C | A | G | G | A | G | A | A | G | A | A | T | T | T | T | T | G | A | C | A | A | C | T | C | T | G | A | C | A | A | A | T | T | C | G | T | T | T | T | A | C | A | C | T | A | T | A | T | T | T | G | T | T | G | G | A | A | C | T | A | A | C | T | A | T | C | T | T | A | C | A | C | A | G | G | G | A | A | C | T | C | G | T | G | A | A | A | A | G | G | A | G | A | A | A | G | C | T | G | T | G | C | A | A | G | A | A | G | C | T | A | G | A | A | A | C | A | G | G | T | T |  | : |  | 2 | 5 | 6 | 2 |
| M | T | 0 | 2 | 7 | 0 | 0 | 7 |  | : |  | A | C | A | A | T | T | G | T | T | G | G | A | A | T | C | A | C | T | T | C | A | C | A | C | T | G | A | T | G | C | C | T | T | C | A | A | A | C | A | A | G | C | T | T | C | T | T | C | A | A | C | A | G | G | A | G | A | A | G | A | A | T | T | T | T | T | G | A | C | A | A | C | T | C | T | G | A | C | A | A | A | T | T | C | G | T | T | T | T | A | C | A | C | T | A | T | A | T | T | T | G | T | T | G | G | A | A | C | T | A | A | C | T | A | T | C | T | T | A | C | A | C | A | G | G | G | A | A | C | T | C | G | T | G | A | A | A | A | A | G | A | G | A | A | A | G | C | T | G | T | G | C | A | A | G | A | A | G | C | T | A | G | A | A | A | C | A | G | G | T | T |  | : |  | 2 | 5 | 6 | 5 |
| M | T | 0 | 3 | 6 | 0 | 5 | 3 |  | : |  | A | C | A | A | T | T | G | T | T | G | G | A | A | T | C | A | C | T | T | C | A | C | A | C | T | G | A | T | G | C | C | T | T | C | A | A | A | C | A | A | G | C | T | T | C | T | T | C | A | A | C | A | G | G | A | G | A | A | G | A | A | T | T | T | T | T | G | A | C | A | A | C | T | C | T | G | A | C | A | A | A | T | T | C | G | T | T | T | T | A | C | A | C | T | A | T | A | T | T | T | G | T | T | G | G | A | A | C | T | A | A | C | T | A | T | C | T | T | A | C | A | C | A | G | G | G | A | A | C | T | C | G | T | G | A | A | A | A | A | G | A | G | A | A | A | G | C | T | G | T | G | C | A | A | G | A | A | G | C | T | A | G | A | A | A | C | A | G | G | T | T |  | : |  | 2 | 5 | 6 | 5 |
| M | T | 0 | 3 | 6 | 0 | 5 | 4 |  | : |  | A | C | A | A | T | T | G | T | T | G | G | A | A | T | C | A | C | T | T | C | A | C | A | C | T | G | A | T | G | C | C | T | T | C | A | A | A | C | A | A | G | C | T | T | C | T | T | C | A | A | C | A | G | G | A | G | A | A | G | A | A | T | T | T | T | T | G | A | C | A | A | C | T | C | T | G | A | C | A | A | A | T | T | C | G | T | T | T | T | A | C | A | C | T | A | T | A | T | T | T | G | T | T | G | G | A | A | C | T | A | A | T | T | A | T | C | T | T | A | C | A | C | A | G | G | G | A | A | C | T | C | G | T | G | A | A | A | A | A | G | A | G | A | A | A | G | C | T | G | T | G | C | A | A | G | A | A | G | C | T | A | G | A | A | A | C | A | G | G | T | T |  | : |  | 2 | 5 | 6 | 5 |
| M | T | 0 | 3 | 6 | 0 | 5 | 5 |  | : |  | A | C | A | A | T | T | G | T | T | G | G | A | A | T | C | A | C | T | T | C | A | T | A | C | T | G | A | T | G | C | C | T | T | C | A | A | A | C | A | A | G | C | T | T | C | T | T | C | A | A | C | A | G | G | A | G | A | A | G | A | A | T | T | T | T | T | G | A | C | A | A | C | T | C | T | G | A | C | A | A | A | T | T | C | G | T | T | T | T | A | C | A | C | T | A | T | A | T | T | T | G | T | T | G | G | A | A | C | T | A | A | C | T | A | T | C | T | T | A | C | A | C | A | G | G | G | A | A | C | T | C | G | T | G | A | A | A | A | A | G | A | G | A | A | A | G | C | T | G | T | G | C | A | A | G | A | A | G | C | T | A | G | A | A | A | C | A | G | G | T | T |  | : |  | 2 | 5 | 6 | 5 |
| M | T | 0 | 3 | 6 | 0 | 5 | 6 |  | : |  | A | C | A | A | T | T | G | T | T | G | G | A | A | T | C | A | C | T | T | C | A | C | A | C | T | G | A | T | G | C | C | T | T | C | A | A | A | C | A | A | G | C | T | T | C | T | T | C | A | A | C | A | G | G | A | G | A | A | G | A | A | T | T | T | T | T | G | A | C | A | A | C | T | C | T | G | A | C | A | A | A | T | T | C | G | T | T | T | T | A | C | A | C | T | A | T | A | T | T | T | G | T | T | G | G | A | A | C | T | A | A | C | T | A | T | C | T | T | A | C | A | C | A | G | G | G | A | A | C | T | C | G | T | G | A | A | A | A | A | G | A | G | A | A | A | G | C | T | G | T | G | C | A | A | G | A | A | G | C | T | A | G | A | A | A | C | A | G | G | T | T |  | : |  | 2 | 5 | 6 | 2 |
| M | T | 0 | 3 | 6 | 0 | 5 | 7 |  | : |  | A | C | A | A | T | T | G | T | T | G | G | A | A | T | C | A | C | T | C | C | A | C | A | C | T | G | A | T | G | C | C | T | T | C | A | A | A | C | A | A | G | C | T | T | C | T | T | C | A | A | C | A | G | G | A | G | A | A | G | A | A | T | T | T | T | T | G | A | C | A | A | C | T | C | T | G | A | C | A | A | A | T | T | C | G | T | T | T | T | A | C | A | C | T | A | T | A | T | T | T | G | T | T | G | G | A | A | C | T | A | A | C | T | A | T | C | T | T | A | C | A | C | A | G | G | G | A | A | C | T | C | G | T | G | A | A | A | A | G | G | A | G | A | A | A | G | C | T | G | T | G | C | A | A | G | A | A | G | C | T | A | G | A | A | A | C | A | G | G | T | T |  | : |  | 2 | 5 | 6 | 2 |
| K | X | 2 | 4 | 9 | 7 | 3 | 8 |  | : |  | A | C | A | A | T | T | G | T | T | G | G | A | A | T | C | A | C | T | T | C | A | C | A | C | T | G | A | T | G | C | C | T | T | C | A | A | A | C | A | A | G | C | T | T | C | T | T | C | A | A | C | A | G | G | A | G | A | A | G | A | A | T | T | T | T | T | G | A | C | A | A | C | T | C | T | G | A | C | A | A | A | T | T | C | G | T | T | T | T | A | C | A | C | T | A | T | A | T | T | T | G | T | T | G | G | A | A | C | T | A | A | C | T | A | T | C | T | T | A | C | A | C | A | G | G | G | A | A | C | T | C | G | T | G | A | A | A | A | G | G | A | G | A | A | A | G | C | T | G | T | G | C | A | A | G | A | A | G | C | T | A | G | A | A | A | C | A | G | G | T | T |  | : |  | 2 | 5 | 6 | 2 |
| K | X | 2 | 4 | 9 | 7 | 3 | 7 |  | : |  | A | C | A | A | T | T | G | T | T | G | G | A | A | T | C | A | C | T | T | C | A | C | A | C | T | G | A | T | G | C | C | T | T | C | A | A | A | C | A | A | G | C | T | T | C | T | T | C | A | A | C | A | G | G | A | G | A | A | G | A | A | T | T | T | T | T | G | A | C | A | A | C | T | C | T | G | A | C | A | A | A | T | T | C | G | T | T | T | T | A | C | A | C | T | A | T | A | T | T | T | G | T | T | G | G | A | A | C | T | A | A | C | T | A | T | C | T | T | A | C | A | C | A | G | G | G | A | A | C | T | C | G | T | G | A | A | A | A | G | G | A | G | A | A | A | G | C | T | G | T | G | C | A | A | G | A | A | G | C | T | A | G | A | A | A | C | A | G | G | T | T |  | : |  | 2 | 5 | 6 | 2 |
| K | X | 2 | 4 | 9 | 7 | 3 | 6 |  | : |  | A | C | A | A | T | T | G | T | T | G | G | A | A | T | C | A | C | T | T | C | A | C | A | C | T | G | A | T | G | C | C | T | T | C | A | A | A | C | A | A | G | C | T | T | C | T | T | C | A | A | C | A | G | G | A | G | A | A | G | A | A | T | T | T | T | T | G | A | C | A | A | C | T | C | T | G | A | C | A | A | A | T | T | C | G | T | T | T | T | A | C | A | C | T | A | T | A | T | T | T | G | T | T | G | G | A | A | C | T | A | A | C | T | A | T | C | T | T | A | C | A | C | A | G | G | G | A | A | C | T | C | G | T | G | A | A | A | A | G | G | A | G | A | A | A | G | C | T | G | T | G | C | A | A | G | A | A | G | C | T | A | G | A | A | A | C | A | G | G | T | T |  | : |  | 2 | 5 | 6 | 2 |
| K | X | 2 | 4 | 9 | 7 | 3 | 5 |  | : |  | A | C | A | A | T | T | G | T | T | G | G | A | A | T | C | A | C | T | T | C | A | C | A | C | T | G | A | T | G | C | C | T | T | C | A | A | A | C | A | A | G | C | T | T | C | T | T | C | A | A | C | A | G | G | A | G | A | A | G | A | A | T | T | T | T | T | G | A | C | A | A | C | T | C | T | G | A | C | A | A | A | T | T | C | G | T | T | T | T | A | C | A | C | T | A | T | A | T | T | T | G | T | T | G | G | A | A | C | T | A | A | C | T | A | T | C | T | T | A | C | A | C | A | G | G | G | A | A | C | T | C | G | T | G | A | A | A | A | G | G | A | G | A | A | A | G | C | T | G | T | G | C | A | A | G | A | A | G | C | T | A | G | A | A | A | C | A | G | G | T | T |  | : |  | 2 | 5 | 6 | 2 |
| M | F | 1 | 9 | 7 | 9 | 1 | 6 |  | : |  | A | C | A | A | T | T | G | T | T | G | G | A | A | T | C | A | C | T | T | C | A | C | A | C | T | G | A | T | G | C | C | T | T | C | A | A | A | C | A | A | G | C | T | T | C | T | T | C | A | A | C | A | G | G | A | G | A | A | G | A | A | T | T | T | T | T | G | A | C | A | A | C | T | C | T | A | A | C | A | A | A | T | T | C | G | T | T | T | T | A | T | A | C | T | A | T | A | T | T | T | G | T | T | G | G | A | A | C | T | A | A | C | T | A | T | C | T | T | A | C | A | C | A | G | G | G | A | A | C | C | C | G | T | G | A | A | A | A | G | G | A | G | A | A | A | G | C | T | G | T | G | C | A | A | G | A | A | G | C | T | A | G | A | A | A | C | A | G | G | T | T |  | : |  | 2 | 5 | 6 | 5 |
| K | T | 2 | 5 | 0 | 6 | 3 | 2 |  | : |  | A | C | A | A | T | T | G | T | T | G | G | A | A | T | C | A | C | T | T | C | A | C | A | C | T | G | A | T | G | C | C | T | T | C | A | A | A | C | A | A | G | C | T | T | C | T | T | C | A | A | C | A | G | G | A | G | A | A | G | A | A | T | T | T | T | T | G | A | C | A | A | C | T | C | T | G | A | C | A | A | A | T | T | C | G | T | T | T | T | A | C | A | C | T | A | T | A | T | T | T | G | T | T | G | G | A | A | C | T | A | A | C | T | A | T | C | T | T | A | C | A | C | A | G | G | G | A | A | C | T | C | G | T | G | A | A | A | A | A | G | A | G | A | A | A | G | C | T | G | T | G | C | A | A | G | A | A | G | C | T | A | G | A | A | A | C | A | G | G | T | T |  | : |  | 2 | 5 | 6 | 5 |
|  |  |  |  |  |  |  |  |  |  |  | A | C | A | A | T | T | G | T | T | G | G | A | A | T | C | A | C | T | t | C | A | c | A | C | T | G | A | T | G | C | C | T | T | C | A | A | A | C | A | A | G | C | T | T | C | T | T | C | A | A | C | A | G | G | A | G | A | A | G | A | A | T | T | T | T | T | G | A | C | A | A | C | T | C | T | g | A | C | A | A | A | T | T | C | G | T | T | T | T | A | c | A | C | T | A | T | A | T | T | T | G | T | T | G | G | A | A | C | T | A | A | c | T | A | T | C | T | T | A | C | A | C | A | G | G | G | A | A | C | t | C | G | T | G | A | A | A | A |  | G | A | G | A | A | A | G | C | T | G | T | G | C | A | A | G | A | A | G | C | T | A | G | A | A | A | C | A | G | G | T | T |  |  |  |  |  |  |  |

|  |  |  |  |  |  |  |  |  |  |  |  |  |  |  |  |  |  |  |  |  |  |  |  |  |  |  |  |  |  |  |  |  |  |  |  |  |  |  |  |  |  |  |  |  |  |  |  |  |  |  |  |  |  |  |  |  |  |  |  |  |  |  |  |  |  |  |  |  |  |  |  |  |  |  |  |  |  |  |  |  |  |  |  |  |  |  |  |  |  |  |  |  |  |  |  |  |  |  |  |  |  |  |  |  |  |  |  |  |  |  |  |  |  |  |  |  |  |  |  |  |  |  |  |  |  |  |  |  |  |  |  |  |  |  |  |  |  |  |  |  |  |  |  |  |  |  |  |  |  |  |  |  |  |  |  |  |  |  |  |  |  |  |  |  |  |  |  |  |  |  |  |  |  |  |  |  |  |  |  |  |  |  |  |  |  |  |  |  |
| --- | --- | --- | --- | --- | --- | --- | --- | --- | --- | --- | --- | --- | --- | --- | --- | --- | --- | --- | --- | --- | --- | --- | --- | --- | --- | --- | --- | --- | --- | --- | --- | --- | --- | --- | --- | --- | --- | --- | --- | --- | --- | --- | --- | --- | --- | --- | --- | --- | --- | --- | --- | --- | --- | --- | --- | --- | --- | --- | --- | --- | --- | --- | --- | --- | --- | --- | --- | --- | --- | --- | --- | --- | --- | --- | --- | --- | --- | --- | --- | --- | --- | --- | --- | --- | --- | --- | --- | --- | --- | --- | --- | --- | --- | --- | --- | --- | --- | --- | --- | --- | --- | --- | --- | --- | --- | --- | --- | --- | --- | --- | --- | --- | --- | --- | --- | --- | --- | --- | --- | --- | --- | --- | --- | --- | --- | --- | --- | --- | --- | --- | --- | --- | --- | --- | --- | --- | --- | --- | --- | --- | --- | --- | --- | --- | --- | --- | --- | --- | --- | --- | --- | --- | --- | --- | --- | --- | --- | --- | --- | --- | --- | --- | --- | --- | --- | --- | --- | --- | --- | --- | --- | --- | --- | --- | --- | --- | --- | --- | --- | --- | --- | --- | --- | --- | --- | --- | --- | --- |
|  |  |  |  |  |  |  |  |  |  |  |  |  |  |  |  |  |  |  |  |  |  |  |  |  |  |  |  |  |  |  |  |  |  |  |  |  |  |  |  |  |  |  |  |  |  |  |  |  |  |  |  |  |  |  |  |  |  |  |  |  |  |  |  |  |  |  |  |  |  |  |  |  |  |  |  |  |  |  |  |  |  |  |  |  |  |  |  |  |  |  |  |  |  |  |  |  |  |  |  |  |  |  |  |  |  |  |  |  |  |  |  |  |  |  |  |  |  |  |  |  |  |  |  |  |  |  |  |  |  |  |  |  |  |  |  |  |  |  |  |  |  |  |  |  |  |  |  |  |  |  |  |  |  |  |  |  |  |  |  |  |  |  |  |  |  |  |  |  |  |  |  |  |  |  |  |  |  |  |  |  |  |  |  |  |  |  |  |  |
|  |  |  |  |  |  |  |  |  |  |  |  |  |  |  | \* |  |  |  |  |  |  | 2 | 5 | 8 | 0 |  |  |  |  |  |  |  |  |  | \* |  |  |  |  |  |  | 2 | 6 | 0 | 0 |  |  |  |  |  |  |  |  |  | \* |  |  |  |  |  |  | 2 | 6 | 2 | 0 |  |  |  |  |  |  |  |  |  | \* |  |  |  |  |  |  | 2 | 6 | 4 | 0 |  |  |  |  |  |  |  |  |  | \* |  |  |  |  |  |  | 2 | 6 | 6 | 0 |  |  |  |  |  |  |  |  |  | \* |  |  |  |  |  |  | 2 | 6 | 8 | 0 |  |  |  |  |  |  |  |  |  | \* |  |  |  |  |  |  | 2 | 7 | 0 | 0 |  |  |  |  |  |  |  |  |  | \* |  |  |  |  |  |  | 2 | 7 | 2 | 0 |  |  |  |  |  |  |  |  |  | \* |  |  |  |  |  |  |  |  |  |  |  |  |  |
| M | N | 9 | 5 | 6 | 5 | 2 | 0 |  | : |  | A | G | T | C | A | A | A | T | T | A | C | A | A | A | T | T | T | G | C | A | A | T | T | T | A | T | G | T | T | C | A | C | T | A | G | A | A | A | G | C | T | T | T | T | T | C | T | G | T | G | A | C | T | A | T | G | A | A | A | C | C | A | A | T | C | T | T | C | T | T | A | A | G | T | T | A | C | C | T | A | T | A | G | A | A | G | A | A | T | G | G | C | C | C | A | A | A | T | A | T | A | T | T | G | A | A | G | A | A | T | A | T | A | T | T | A | G | G | A | A | A | A | T | A | C | C | C | T | T | T | G | T | A | G | G | G | A | T | G | G | A | A | G | T | G | C | T | C | G | A | A | G | A | G | T | A | T | T | C | C | A | A | G | C | A | G | G | A | C | A | A |  | : |  | 2 | 7 | 3 | 3 |
| M | T | 0 | 1 | 2 | 7 | 3 | 2 |  | : |  | A | G | T | C | A | A | A | T | T | A | C | A | A | A | T | T | T | G | C | A | A | T | T | T | A | T | G | T | T | C | A | C | T | A | G | A | A | A | G | C | T | T | T | T | T | C | T | G | T | G | A | C | T | A | T | G | A | A | A | C | C | A | A | T | C | T | T | C | T | T | A | A | G | T | T | A | C | C | T | A | T | A | G | A | A | G | A | A | T | G | G | C | C | C | A | A | A | T | A | T | A | T | T | G | A | A | G | A | A | T | A | T | A | T | T | A | G | G | A | A | A | A | T | A | C | C | C | T | T | T | G | T | A | G | G | G | A | T | G | G | A | A | G | T | G | C | T | C | G | A | A | G | A | G | T | A | T | T | C | C | A | A | G | C | A | G | G | A | C | A | A |  | : |  | 2 | 7 | 3 | 3 |
| M | T | 0 | 1 | 2 | 7 | 3 | 4 |  | : |  | A | G | T | C | A | A | A | T | T | A | C | A | A | A | T | T | T | G | C | A | A | T | T | T | A | T | G | T | T | C | A | C | T | A | G | A | A | A | G | C | T | T | T | T | T | C | T | G | T | G | A | C | T | A | T | G | A | A | A | C | C | A | A | T | C | T | T | C | T | T | A | A | G | T | T | A | C | C | T | A | T | A | G | A | A | G | A | A | T | G | G | C | C | C | A | A | A | T | A | T | A | T | T | G | A | A | G | A | A | T | A | T | A | T | T | A | G | G | A | A | A | A | T | A | C | C | C | T | T | T | G | T | A | G | G | G | A | T | G | G | A | A | G | T | G | C | T | C | G | A | A | G | A | G | T | A | T | T | C | C | A | A | G | C | A | G | G | A | C | A | A |  | : |  | 2 | 7 | 3 | 3 |
| M | T | 0 | 2 | 7 | 0 | 0 | 6 |  | : |  | A | G | T | C | A | A | A | T | T | A | C | A | A | A | T | T | T | G | C | A | A | T | T | T | A | T | G | T | T | C | A | C | T | A | G | A | A | A | G | C | T | T | T | T | T | C | T | G | T | G | A | C | T | A | T | G | A | A | A | C | C | A | A | T | C | T | T | C | T | T | A | A | G | T | T | A | C | C | A | A | T | A | G | A | A | G | A | A | T | G | G | C | C | C | A | A | A | T | A | T | A | T | T | G | A | A | G | A | A | T | A | T | A | T | T | A | G | G | A | A | A | A | T | A | C | C | C | T | T | G | G | T | G | G | G | A | A | T | G | G | A | A | G | T | A | C | T | C | G | A | A | G | A | A | T | A | C | T | C | C | A | A | G | C | A | G | G | A | T | A | A |  | : |  | 2 | 7 | 3 | 3 |
| M | T | 0 | 2 | 7 | 0 | 0 | 7 |  | : |  | A | G | T | C | A | A | A | T | T | A | C | A | A | A | T | T | T | G | C | A | A | T | T | T | A | T | G | T | T | C | A | C | T | A | G | A | A | A | G | C | T | T | T | T | T | C | T | G | T | G | A | C | T | A | T | G | A | A | A | C | C | A | A | T | C | T | T | C | T | G | A | A | G | T | T | A | C | C | T | A | T | A | G | A | A | G | A | A | T | G | G | C | C | C | A | A | A | T | A | T | A | T | T | G | A | A | G | A | A | T | A | T | A | T | T | A | G | G | A | A | A | A | T | A | C | C | C | T | T | T | G | T | A | G | G | G | A | T | G | G | A | A | G | T | A | C | T | C | G | A | A | G | A | G | T | A | T | T | C | C | A | A | G | C | A | G | G | A | C | A | A |  | : |  | 2 | 7 | 3 | 6 |
| M | T | 0 | 3 | 6 | 0 | 5 | 3 |  | : |  | A | G | T | C | A | A | A | T | T | A | C | A | A | A | T | T | T | G | C | A | A | T | T | T | A | T | G | T | T | C | A | C | T | A | G | A | A | A | G | C | T | T | T | T | T | C | T | G | T | G | A | C | T | A | T | G | A | A | A | C | C | A | A | T | C | T | T | C | T | G | A | A | G | T | T | A | C | C | T | A | T | A | G | A | A | G | A | A | T | G | G | C | C | C | A | A | A | T | A | T | A | T | T | G | A | A | G | A | A | T | A | T | A | T | T | A | G | G | A | A | A | A | T | A | C | C | C | T | T | T | G | T | A | G | G | G | A | T | G | G | A | A | G | T | A | C | T | C | G | A | A | G | A | G | T | A | T | T | C | C | A | A | G | C | A | G | G | A | C | A | A |  | : |  | 2 | 7 | 3 | 6 |
| M | T | 0 | 3 | 6 | 0 | 5 | 4 |  | : |  | A | G | T | C | A | A | A | T | T | A | C | A | A | A | T | T | T | G | C | A | A | T | T | T | A | T | G | T | T | C | A | C | T | A | G | A | A | A | G | C | T | T | T | T | T | C | T | G | T | G | A | C | T | A | T | G | A | A | A | C | C | A | A | T | C | T | T | C | T | G | A | A | G | T | T | A | C | C | T | A | T | A | G | A | A | G | A | A | T | G | G | C | C | C | A | A | A | T | A | T | A | T | T | G | A | A | G | A | A | T | A | T | A | T | T | A | G | G | A | A | A | A | T | A | C | C | C | T | T | T | G | T | A | G | G | G | A | T | G | G | A | A | G | T | A | C | T | C | G | A | A | G | A | G | T | A | T | T | C | T | A | A | G | C | A | G | G | A | C | A | A |  | : |  | 2 | 7 | 3 | 6 |
| M | T | 0 | 3 | 6 | 0 | 5 | 5 |  | : |  | A | G | T | C | A | A | A | T | T | A | C | A | A | A | T | T | T | G | C | A | A | T | T | T | A | T | G | T | T | C | A | C | T | A | G | A | A | A | G | C | T | T | T | T | T | C | T | G | T | G | A | C | T | A | T | G | A | A | A | C | C | A | A | T | C | T | T | C | T | G | A | A | G | T | T | A | C | C | T | A | T | A | G | A | A | G | A | A | T | G | G | C | C | C | A | A | A | T | A | T | A | T | T | G | A | A | G | A | A | T | A | T | A | T | T | A | G | G | A | A | A | A | T | A | C | C | C | T | T | T | G | T | A | G | G | G | A | T | G | G | A | A | G | T | A | C | T | C | G | A | A | G | A | G | T | A | T | T | C | C | A | A | G | C | A | G | G | A | C | A | A |  | : |  | 2 | 7 | 3 | 6 |
| M | T | 0 | 3 | 6 | 0 | 5 | 6 |  | : |  | A | G | T | C | A | A | A | T | T | A | C | A | A | A | T | T | T | G | C | A | A | T | T | T | A | T | G | T | T | C | A | C | T | A | G | A | A | A | G | C | T | T | T | T | T | C | A | G | T | G | A | T | T | A | T | G | A | A | A | C | C | A | A | T | C | T | T | C | T | A | A | A | G | T | T | A | C | C | A | A | T | A | G | A | A | G | A | A | T | G | G | C | C | C | A | A | A | T | A | T | A | T | T | G | A | A | G | A | A | T | A | T | A | T | T | A | G | G | A | A | A | A | T | A | C | C | C | T | T | T | G | T | G | G | G | A | A | T | G | G | A | A | G | T | A | C | T | C | G | A | A | G | A | A | T | A | C | T | C | C | A | A | G | C | A | G | G | A | T | A | A |  | : |  | 2 | 7 | 3 | 3 |
| M | T | 0 | 3 | 6 | 0 | 5 | 7 |  | : |  | A | G | T | C | A | A | A | T | T | A | C | A | A | A | T | T | T | G | C | A | A | T | T | T | A | T | G | T | T | C | A | C | T | A | G | A | A | A | G | C | T | T | T | T | T | C | C | G | T | G | A | C | T | A | T | G | A | A | A | C | C | A | A | T | C | T | T | C | T | T | A | A | G | T | T | A | C | C | T | A | T | A | G | A | A | G | A | A | T | G | G | C | C | C | A | A | A | T | A | T | A | T | T | G | A | A | G | A | A | T | A | T | A | T | T | A | G | G | A | A | A | A | T | A | C | C | C | T | T | T | G | T | A | G | G | G | A | T | G | G | A | A | G | T | G | C | T | C | G | A | A | G | A | G | T | A | T | T | C | C | A | A | G | C | A | G | G | A | C | A | A |  | : |  | 2 | 7 | 3 | 3 |
| K | X | 2 | 4 | 9 | 7 | 3 | 8 |  | : |  | A | G | T | C | A | A | A | T | T | A | C | A | A | A | T | T | T | G | C | A | A | T | T | T | A | T | G | T | T | C | A | C | T | A | G | A | A | A | G | C | T | T | T | T | T | C | T | G | T | G | A | C | T | A | T | G | A | A | A | C | C | A | A | T | C | T | T | C | T | T | A | A | G | T | T | A | C | C | T | A | T | A | G | A | A | G | A | A | T | G | G | C | C | C | A | A | A | T | A | T | A | T | T | G | A | A | G | A | A | T | A | T | A | T | T | A | G | G | A | A | A | A | T | A | C | C | C | T | T | T | G | T | A | G | G | G | A | T | G | G | A | A | G | T | G | C | T | C | G | A | A | G | A | G | T | A | T | T | C | C | A | A | G | C | A | G | G | A | C | A | A |  | : |  | 2 | 7 | 3 | 3 |
| K | X | 2 | 4 | 9 | 7 | 3 | 7 |  | : |  | A | G | T | C | A | A | A | T | T | A | C | A | A | A | T | T | T | G | C | A | A | T | T | T | A | T | G | T | T | C | A | C | T | A | G | A | A | A | G | C | T | T | T | T | T | C | T | G | T | G | A | C | T | A | T | G | A | A | A | C | C | A | A | T | C | T | T | C | T | T | A | A | G | T | T | A | C | C | T | A | T | A | G | A | A | G | A | A | T | G | G | C | C | C | A | A | A | T | A | T | A | T | T | G | A | A | G | A | A | T | A | T | A | T | T | A | G | G | A | A | A | A | T | A | C | C | C | T | T | T | G | T | A | G | G | G | A | T | G | G | A | A | G | T | G | C | T | C | G | A | A | G | A | G | T | A | T | T | C | C | A | A | G | C | A | G | G | A | C | A | A |  | : |  | 2 | 7 | 3 | 3 |
| K | X | 2 | 4 | 9 | 7 | 3 | 6 |  | : |  | A | G | T | C | A | A | A | T | T | A | C | A | A | A | T | T | T | G | C | A | A | T | T | T | A | T | G | T | T | C | A | C | T | A | G | A | A | A | G | C | T | T | T | T | T | C | T | G | T | G | A | C | T | A | T | G | A | A | A | C | C | A | A | T | C | T | T | C | T | T | A | A | G | T | T | A | C | C | T | A | T | A | G | A | A | G | A | A | T | G | G | C | C | C | A | A | A | T | A | T | A | T | T | G | A | A | G | A | A | T | A | T | A | T | T | A | G | G | A | A | A | A | T | A | C | C | C | T | T | T | G | T | A | G | G | G | A | T | G | G | A | A | G | T | G | C | T | C | G | A | A | G | A | G | T | A | T | T | C | C | A | A | G | C | A | G | G | A | C | A | A |  | : |  | 2 | 7 | 3 | 3 |
| K | X | 2 | 4 | 9 | 7 | 3 | 5 |  | : |  | A | G | T | C | A | A | A | T | T | A | C | A | A | A | T | T | T | G | C | A | A | T | T | T | A | T | G | T | T | C | A | C | T | A | G | A | A | A | G | C | T | T | T | T | T | C | T | G | T | G | A | C | T | A | T | G | A | A | A | C | C | A | A | T | C | T | T | C | T | T | A | A | G | T | T | A | C | C | T | A | T | A | G | A | A | G | A | A | T | G | G | C | C | C | A | A | A | T | A | T | A | T | T | G | A | A | G | A | A | T | A | T | A | T | T | A | G | G | A | A | A | A | T | A | C | C | C | T | T | T | G | T | A | G | G | G | A | T | G | G | A | A | G | T | G | C | T | C | G | A | A | G | A | G | T | A | T | T | C | C | A | A | G | C | A | G | G | A | C | A | A |  | : |  | 2 | 7 | 3 | 3 |
| M | F | 1 | 9 | 7 | 9 | 1 | 6 |  | : |  | A | G | T | C | A | A | A | T | T | A | C | A | A | A | T | T | T | G | C | A | A | T | C | T | A | T | G | T | T | C | A | C | T | A | G | A | A | A | G | T | T | T | T | T | T | C | T | G | T | G | A | C | T | A | T | G | A | A | A | C | C | A | A | T | C | T | T | C | T | G | A | A | G | T | T | A | C | C | T | A | T | A | G | A | A | G | A | A | T | G | G | C | C | C | A | A | A | T | A | T | A | T | T | G | A | A | G | A | A | T | A | T | A | T | T | A | G | G | A | A | A | A | T | A | C | C | C | T | T | T | G | T | A | G | G | G | A | T | G | G | A | A | G | T | A | C | T | C | G | A | A | G | A | G | T | A | T | T | C | T | A | A | A | C | A | A | G | A | C | A | A |  | : |  | 2 | 7 | 3 | 6 |
| K | T | 2 | 5 | 0 | 6 | 3 | 2 |  | : |  | A | G | T | C | A | A | A | T | T | A | C | A | A | A | T | T | T | G | C | A | A | T | T | T | A | T | G | T | T | C | A | C | T | A | G | A | A | A | G | C | T | T | T | T | T | C | T | G | T | G | A | C | T | A | T | G | A | A | A | C | C | A | A | T | C | T | T | C | T | G | A | A | G | T | T | A | C | C | T | A | T | A | G | A | A | G | A | A | T | G | G | C | C | C | A | A | A | T | A | T | A | T | T | G | A | A | G | A | A | T | A | T | A | T | T | A | G | G | A | A | A | A | T | A | C | C | C | T | T | T | G | T | A | G | G | G | A | T | G | G | A | A | G | T | A | C | T | C | G | A | A | G | A | G | T | A | T | T | C | C | A | A | G | C | A | A | G | A | C | A | A |  | : |  | 2 | 7 | 3 | 6 |
|  |  |  |  |  |  |  |  |  |  |  | A | G | T | C | A | A | A | T | T | A | C | A | A | A | T | T | T | G | C | A | A | T | t | T | A | T | G | T | T | C | A | C | T | A | G | A | A | A | G | c | T | T | T | T | T | C | t | G | T | G | A | c | T | A | T | G | A | A | A | C | C | A | A | T | C | T | T | C | T |  | A | A | G | T | T | A | C | C | t | A | T | A | G | A | A | G | A | A | T | G | G | C | C | C | A | A | A | T | A | T | A | T | T | G | A | A | G | A | A | T | A | T | A | T | T | A | G | G | A | A | A | A | T | A | C | C | C | T | T | t | G | T | a | G | G | g | A | T | G | G | A | A | G | T |  | C | T | C | G | A | A | G | A | g | T | A | t | T | C | c | A | A | g | C | A | g | G | A | c | A | A |  |  |  |  |  |  |  |

|  |  |  |  |  |  |  |  |  |  |  |  |  |  |  |  |  |  |  |  |  |  |  |  |  |  |  |  |  |  |  |  |  |  |  |  |  |  |  |  |  |  |  |  |  |  |  |  |  |  |  |  |  |  |  |  |  |  |  |  |  |  |  |  |  |  |  |  |  |  |  |  |  |  |  |  |  |  |  |  |  |  |  |  |  |  |  |  |  |  |  |  |  |  |  |  |  |  |  |  |  |  |  |  |  |  |  |  |  |  |  |  |  |  |  |  |  |  |  |  |  |  |  |  |  |  |  |  |  |  |  |  |  |  |  |  |  |  |  |  |  |  |  |  |  |  |  |  |  |  |  |  |  |  |  |  |  |  |  |  |  |  |  |  |  |  |  |  |  |  |  |  |  |  |  |  |  |  |  |  |  |  |  |  |  |  |  |  |  |
| --- | --- | --- | --- | --- | --- | --- | --- | --- | --- | --- | --- | --- | --- | --- | --- | --- | --- | --- | --- | --- | --- | --- | --- | --- | --- | --- | --- | --- | --- | --- | --- | --- | --- | --- | --- | --- | --- | --- | --- | --- | --- | --- | --- | --- | --- | --- | --- | --- | --- | --- | --- | --- | --- | --- | --- | --- | --- | --- | --- | --- | --- | --- | --- | --- | --- | --- | --- | --- | --- | --- | --- | --- | --- | --- | --- | --- | --- | --- | --- | --- | --- | --- | --- | --- | --- | --- | --- | --- | --- | --- | --- | --- | --- | --- | --- | --- | --- | --- | --- | --- | --- | --- | --- | --- | --- | --- | --- | --- | --- | --- | --- | --- | --- | --- | --- | --- | --- | --- | --- | --- | --- | --- | --- | --- | --- | --- | --- | --- | --- | --- | --- | --- | --- | --- | --- | --- | --- | --- | --- | --- | --- | --- | --- | --- | --- | --- | --- | --- | --- | --- | --- | --- | --- | --- | --- | --- | --- | --- | --- | --- | --- | --- | --- | --- | --- | --- | --- | --- | --- | --- | --- | --- | --- | --- | --- | --- | --- | --- | --- | --- | --- | --- | --- | --- | --- | --- | --- | --- |
|  |  |  |  |  |  |  |  |  |  |  |  |  |  |  |  |  |  |  |  |  |  |  |  |  |  |  |  |  |  |  |  |  |  |  |  |  |  |  |  |  |  |  |  |  |  |  |  |  |  |  |  |  |  |  |  |  |  |  |  |  |  |  |  |  |  |  |  |  |  |  |  |  |  |  |  |  |  |  |  |  |  |  |  |  |  |  |  |  |  |  |  |  |  |  |  |  |  |  |  |  |  |  |  |  |  |  |  |  |  |  |  |  |  |  |  |  |  |  |  |  |  |  |  |  |  |  |  |  |  |  |  |  |  |  |  |  |  |  |  |  |  |  |  |  |  |  |  |  |  |  |  |  |  |  |  |  |  |  |  |  |  |  |  |  |  |  |  |  |  |  |  |  |  |  |  |  |  |  |  |  |  |  |  |  |  |  |  |  |
|  |  |  |  |  |  |  |  |  |  |  | 2 | 7 | 4 | 0 |  |  |  |  |  |  |  |  |  | \* |  |  |  |  |  |  | 2 | 7 | 6 | 0 |  |  |  |  |  |  |  |  |  | \* |  |  |  |  |  |  | 2 | 7 | 8 | 0 |  |  |  |  |  |  |  |  |  | \* |  |  |  |  |  |  | 2 | 8 | 0 | 0 |  |  |  |  |  |  |  |  |  | \* |  |  |  |  |  |  | 2 | 8 | 2 | 0 |  |  |  |  |  |  |  |  |  | \* |  |  |  |  |  |  | 2 | 8 | 4 | 0 |  |  |  |  |  |  |  |  |  | \* |  |  |  |  |  |  | 2 | 8 | 6 | 0 |  |  |  |  |  |  |  |  |  | \* |  |  |  |  |  |  | 2 | 8 | 8 | 0 |  |  |  |  |  |  |  |  |  | \* |  |  |  |  |  |  | 2 | 9 | 0 | 0 |  |  |  |  |  |  |  |  |  |  |  |  |  |  |
| M | N | 9 | 5 | 6 | 5 | 2 | 0 |  | : |  | T | A | T | C | A | C | T | A | A | A | G | G | T | T | C | T | T | T | A | G | G | A | T | A | T | G | C | C | C | A | C | A | A | T | C | T | G | A | T | A | A | A | G | A | G | T | T | A | C | A | T | G | G | A | G | A | A | G | A | A | G | T | G | T | A | A | G | T | C | T | T | T | A | A | A | G | A | T | A | A | A | A | A | A | G | G | A | A | A | T | A | C | G | T | A | G | A | A | A | T | A | T | G | T | G | T | T | G | T | C | C | T | A | A | G | T | T | T | T | C | T | T | C | A | C | C | G | G | A | A | A | C | A | C | A | A | T | A | T | G | G | G | T | G | C | A | A | A | C | C | C | A | T | T | A | G | C | C | A | T | A | A | A | A | A | A | G | C | T | A | A |  | : |  | 2 | 9 | 0 | 4 |
| M | T | 0 | 1 | 2 | 7 | 3 | 2 |  | : |  | T | A | T | C | A | C | T | A | A | A | G | G | T | T | C | T | T | T | A | G | G | A | T | A | T | G | C | C | C | A | C | A | A | T | C | T | G | A | T | A | A | A | G | A | G | T | T | A | C | A | T | G | G | A | A | A | A | G | A | A | G | T | G | T | A | A | G | T | C | T | T | T | A | A | A | G | A | T | A | A | A | A | A | A | G | G | A | A | A | T | A | C | G | T | A | G | A | A | A | T | A | T | G | T | G | T | T | G | T | C | C | T | A | A | G | T | T | T | T | C | T | T | C | A | C | C | G | G | A | A | A | C | A | C | A | A | T | A | T | G | G | G | T | G | C | A | A | A | C | C | C | A | T | T | A | G | C | C | A | T | A | A | A | A | A | A | G | C | T | A | A |  | : |  | 2 | 9 | 0 | 4 |
| M | T | 0 | 1 | 2 | 7 | 3 | 4 |  | : |  | T | A | T | C | A | C | T | A | A | A | G | G | T | T | C | T | T | T | A | G | G | A | T | A | T | G | C | C | C | A | C | A | A | T | C | T | G | A | T | A | A | A | G | A | G | T | T | A | C | A | T | G | G | A | A | A | A | G | A | A | G | T | G | T | A | A | G | T | C | T | T | T | A | A | A | G | A | T | A | A | A | A | A | A | G | G | A | A | A | T | A | C | G | T | A | G | A | A | A | T | A | T | G | T | G | T | T | G | T | C | C | T | A | A | G | T | T | T | T | C | T | T | C | A | C | C | G | G | A | A | A | C | A | C | A | A | T | A | T | G | G | G | T | G | C | A | A | A | C | C | C | A | T | T | A | G | C | C | A | T | A | A | A | A | A | A | G | C | T | A | A |  | : |  | 2 | 9 | 0 | 4 |
| M | T | 0 | 2 | 7 | 0 | 0 | 6 |  | : |  | C | A | T | A | A | C | T | A | A | A | G | G | A | T | C | A | C | T | T | G | G | T | T | A | T | G | C | C | C | A | C | A | A | C | C | T | C | A | T | C | A | A | A | G | C | C | T | A | C | A | T | G | G | A | A | A | A | G | A | A | G | T | G | T | A | A | G | T | C | T | T | T | G | A | A | G | A | T | C | A | A | A | A | A | G | G | A | A | A | T | A | C | G | T | A | G | A | A | A | T | A | T | G | T | G | T | T | G | T | C | C | T | A | A | G | T | T | T | T | C | C | T | C | A | C | C | G | G | A | A | A | C | A | C | A | A | T | A | T | G | G | G | T | G | T | A | A | A | C | C | C | T | T | T | A | G | C | C | A | T | A | A | A | A | A | A | G | C | T | A | A |  | : |  | 2 | 9 | 0 | 4 |
| M | T | 0 | 2 | 7 | 0 | 0 | 7 |  | : |  | T | A | T | C | A | C | T | A | A | A | G | G | A | T | C | T | T | T | A | G | G | A | T | A | T | G | C | C | C | A | C | A | A | T | C | T | G | A | T | A | A | A | G | A | G | T | T | A | C | A | T | G | G | A | A | A | A | G | A | A | G | T | G | T | A | A | G | T | C | T | T | T | A | A | A | G | A | T | A | A | A | A | A | A | G | G | A | A | A | T | A | C | G | T | A | G | A | A | A | T | A | T | G | T | G | T | T | G | T | C | C | T | A | A | G | T | T | T | T | C | T | T | C | A | C | C | G | G | A | A | A | C | A | C | A | A | T | A | T | G | G | G | T | G | T | A | A | A | C | C | C | T | T | T | A | G | C | C | A | T | A | A | A | A | A | A | G | C | T | A | A |  | : |  | 2 | 9 | 0 | 7 |
| M | T | 0 | 3 | 6 | 0 | 5 | 3 |  | : |  | T | A | T | C | A | C | T | A | A | A | G | G | A | T | C | T | T | T | A | G | G | A | T | A | T | G | C | C | C | A | C | A | A | T | C | T | G | A | T | A | A | A | G | A | G | T | T | A | C | A | T | G | G | A | A | A | A | G | A | A | G | T | G | T | A | A | G | T | C | T | T | T | A | A | A | G | A | T | A | A | A | A | A | A | G | G | A | A | A | T | A | C | G | T | A | G | A | A | A | T | A | T | G | T | G | T | T | G | T | C | C | T | A | A | G | T | T | T | T | C | T | T | C | A | C | C | G | G | A | A | A | C | A | C | A | A | T | A | T | G | G | G | T | G | T | A | A | A | C | C | C | T | T | T | A | G | C | C | A | T | A | A | A | A | A | A | G | C | T | A | A |  | : |  | 2 | 9 | 0 | 7 |
| M | T | 0 | 3 | 6 | 0 | 5 | 4 |  | : |  | T | A | T | C | A | C | T | A | A | A | G | G | A | T | C | T | T | T | A | G | G | A | T | A | T | G | C | C | C | A | C | A | A | T | C | T | G | A | T | A | A | A | G | A | G | T | T | A | C | A | T | G | G | A | A | A | A | G | A | A | G | T | G | T | A | A | G | T | C | T | T | T | A | A | A | G | A | T | A | A | A | A | A | A | G | G | A | A | A | T | A | C | G | T | A | G | A | A | A | T | A | T | G | T | G | T | T | G | T | C | C | T | A | A | G | T | T | T | T | C | T | T | C | A | C | C | G | G | A | A | A | C | A | C | A | A | T | A | T | G | G | G | T | G | T | A | A | A | C | C | C | T | T | T | A | G | C | C | A | T | A | A | A | A | A | A | G | C | T | A | A |  | : |  | 2 | 9 | 0 | 7 |
| M | T | 0 | 3 | 6 | 0 | 5 | 5 |  | : |  | T | A | T | C | A | C | T | A | A | A | G | G | A | T | C | T | T | T | A | G | G | A | T | A | T | G | C | C | C | A | C | A | A | T | C | T | G | A | T | A | A | A | G | A | G | T | T | A | C | A | T | G | G | A | A | A | A | G | A | A | G | T | G | T | A | A | G | T | C | T | T | T | A | A | A | G | A | T | A | A | A | A | A | A | G | G | A | A | A | T | A | C | G | T | A | G | A | A | A | T | A | T | G | T | G | T | T | G | T | C | C | T | A | A | G | T | T | T | T | C | T | T | C | A | C | C | G | G | A | A | A | C | A | C | A | A | T | A | T | G | G | G | T | G | T | A | A | A | C | C | C | A | T | T | A | G | C | C | A | T | A | A | A | A | A | A | G | C | T | A | A |  | : |  | 2 | 9 | 0 | 7 |
| M | T | 0 | 3 | 6 | 0 | 5 | 6 |  | : |  | C | A | T | A | A | C | T | A | A | A | G | G | A | T | C | A | C | T | T | G | G | T | T | A | T | G | C | C | C | A | C | A | A | C | C | T | C | A | T | C | A | A | A | G | C | C | T | A | C | A | T | G | G | A | A | A | A | G | A | A | A | T | G | T | A | A | G | T | C | T | T | T | G | A | A | G | A | T | C | A | A | A | A | A | G | G | A | A | A | T | A | C | G | T | A | G | A | A | A | T | A | T | G | T | G | T | T | G | T | C | C | T | A | A | G | T | T | T | T | C | C | T | C | A | C | C | G | G | A | A | A | C | A | C | A | A | T | A | T | G | G | G | T | G | T | A | A | A | C | C | C | T | T | T | A | G | C | C | A | T | A | A | A | A | A | A | G | C | T | A | A |  | : |  | 2 | 9 | 0 | 4 |
| M | T | 0 | 3 | 6 | 0 | 5 | 7 |  | : |  | T | A | T | C | A | C | T | A | A | A | G | G | T | T | C | T | T | T | A | G | G | A | T | A | T | G | C | C | C | A | C | A | A | T | C | T | G | A | T | A | A | A | G | A | G | T | T | A | C | A | T | G | G | A | A | A | A | G | A | A | G | T | G | T | A | A | G | T | C | T | T | T | A | A | A | G | A | T | A | A | A | A | A | A | G | G | A | A | A | T | A | C | G | T | A | G | A | A | A | T | A | T | G | T | G | T | T | G | T | C | C | T | A | A | G | T | T | T | T | C | T | T | C | A | C | C | G | G | A | A | A | C | A | C | A | A | T | A | T | G | G | G | T | G | C | A | A | A | C | C | C | A | T | T | A | G | C | C | A | T | A | A | A | A | A | A | G | C | T | A | A |  | : |  | 2 | 9 | 0 | 4 |
| K | X | 2 | 4 | 9 | 7 | 3 | 8 |  | : |  | T | A | T | C | A | C | T | A | A | A | G | G | T | T | C | T | T | T | A | G | G | A | T | A | T | G | C | C | C | A | C | A | A | T | C | T | G | A | T | A | A | A | G | A | G | T | T | A | C | A | T | G | G | A | A | A | A | G | A | A | G | T | G | T | A | A | G | T | C | T | T | T | A | A | A | G | A | T | A | A | A | A | A | A | G | G | A | A | A | T | A | C | G | T | A | G | A | A | A | T | A | T | G | T | G | T | T | G | T | C | C | T | A | A | G | T | T | T | T | C | T | T | C | A | C | C | G | G | A | A | A | C | A | C | A | A | T | A | T | G | G | G | T | G | C | A | A | A | C | C | C | A | T | T | A | G | C | C | A | T | A | A | A | A | A | A | G | C | T | A | A |  | : |  | 2 | 9 | 0 | 4 |
| K | X | 2 | 4 | 9 | 7 | 3 | 7 |  | : |  | T | A | T | C | A | C | T | A | A | A | G | G | T | T | C | T | T | T | A | G | G | A | T | A | T | G | C | C | C | A | C | A | A | T | C | T | G | A | T | A | A | A | G | A | G | T | T | A | C | A | T | G | G | A | A | A | A | G | A | A | G | T | G | T | A | A | G | T | C | T | T | T | A | A | A | G | A | T | A | A | A | A | A | A | G | G | A | A | A | T | A | C | G | T | A | G | A | A | A | T | A | T | G | T | G | T | T | G | T | C | C | T | A | A | G | T | T | T | T | C | T | T | C | A | C | C | G | G | A | A | A | C | A | C | A | A | T | A | T | G | G | G | T | G | C | A | A | A | C | C | C | A | T | T | A | G | C | C | A | T | A | A | A | A | A | A | G | C | T | A | A |  | : |  | 2 | 9 | 0 | 4 |
| K | X | 2 | 4 | 9 | 7 | 3 | 6 |  | : |  | T | A | T | C | A | C | T | A | A | A | G | G | T | T | C | T | T | T | A | G | G | A | T | A | T | G | C | C | C | A | C | A | A | T | C | T | G | A | T | A | A | A | G | A | G | T | T | A | C | A | T | G | G | A | A | A | A | G | A | A | G | T | G | T | A | A | G | T | C | T | T | T | A | A | A | G | A | T | A | A | A | A | A | A | G | G | A | A | A | T | A | C | G | T | A | G | A | A | A | T | A | T | G | T | G | T | T | G | T | C | C | T | A | A | G | T | T | T | T | C | T | T | C | A | C | C | G | G | A | A | A | C | A | C | A | A | T | A | T | G | G | G | T | G | C | A | A | A | C | C | C | A | T | T | A | G | C | C | A | T | A | A | A | A | A | A | G | C | T | A | A |  | : |  | 2 | 9 | 0 | 4 |
| K | X | 2 | 4 | 9 | 7 | 3 | 5 |  | : |  | T | A | T | C | A | C | T | A | A | A | G | G | T | T | C | T | T | T | A | G | G | A | T | A | T | G | C | C | C | A | C | A | A | T | T | T | G | A | T | A | A | A | G | A | G | T | T | A | C | A | T | G | G | A | A | A | A | G | A | A | G | T | G | T | A | A | G | T | C | T | T | T | A | A | A | G | A | T | A | A | A | A | A | A | G | G | A | A | A | T | A | C | G | T | A | G | A | A | A | T | A | T | G | T | G | T | T | G | T | C | C | T | A | A | G | T | T | T | T | C | T | T | C | A | C | C | G | G | A | A | A | C | A | C | A | A | T | A | T | G | G | G | T | G | C | A | A | A | C | C | C | A | T | T | A | G | C | C | A | T | A | A | A | A | A | A | G | C | T | A | A |  | : |  | 2 | 9 | 0 | 4 |
| M | F | 1 | 9 | 7 | 9 | 1 | 6 |  | : |  | T | A | T | C | A | C | T | A | A | A | G | G | A | T | C | T | T | T | A | G | G | A | T | A | T | G | C | C | C | A | C | A | A | T | C | T | G | A | T | A | A | A | G | A | G | T | T | A | C | A | T | G | G | A | G | A | A | G | A | A | G | T | G | T | A | A | G | T | C | T | T | T | A | A | A | G | A | T | A | A | A | A | A | A | G | G | A | A | A | T | A | C | G | T | A | G | A | A | A | T | A | T | G | T | G | T | T | G | T | C | C | T | A | A | G | T | T | T | T | C | T | T | C | A | C | C | G | G | A | A | A | C | A | C | A | A | T | A | T | G | G | T | T | G | T | A | A | A | C | C | C | A | T | T | A | G | C | C | A | T | A | A | A | A | A | A | G | C | T | A | A |  | : |  | 2 | 9 | 0 | 7 |
| K | T | 2 | 5 | 0 | 6 | 3 | 2 |  | : |  | T | A | T | C | A | C | T | A | A | A | G | G | A | T | C | T | T | T | A | G | G | A | T | A | T | G | C | C | C | A | C | A | A | T | C | T | G | A | T | A | A | A | G | A | G | T | T | A | C | A | T | G | G | A | A | A | A | G | A | A | G | T | G | T | A | A | G | T | C | T | T | T | A | A | A | G | A | T | A | A | A | A | A | A | G | G | A | A | A | T | A | C | G | T | A | G | A | A | A | T | A | T | G | T | G | T | T | G | T | C | C | T | A | A | G | T | T | T | T | C | T | T | C | A | C | C | G | G | A | A | A | C | A | C | A | A | T | A | T | G | G | G | T | G | T | A | A | A | C | C | C | T | T | T | A | G | C | C | A | T | A | A | A | A | A | A | G | C | T | A | A |  | : |  | 2 | 9 | 0 | 7 |
|  |  |  |  |  |  |  |  |  |  |  | t | A | T | c | A | C | T | A | A | A | G | G |  | T | C | t | t | T | a | G | G | a | T | A | T | G | C | C | C | A | C | A | A | t | c | T | g | A | T | a | A | A | g | a | g | t | T | A | C | A | T | G | G | A | a | A | A | G | A | A | g | T | G | T | A | A | G | T | C | T | T | T | a | A | A | G | A | T | a | A | A | A | A | A | G | G | A | A | A | T | A | C | G | T | A | G | A | A | A | T | A | T | G | T | G | T | T | G | T | C | C | T | A | A | G | T | T | T | T | C | t | T | C | A | C | C | G | G | A | A | A | C | A | C | A | A | T | A | T | G | G | g | T | G |  | A | A | A | C | C | C |  | T | T | A | G | C | C | A | T | A | A | A | A | A | A | G | C | T | A | A |  |  |  |  |  |  |  |

|  |  |  |  |  |  |  |  |  |  |  |  |  |  |  |  |  |  |  |  |  |  |  |  |  |  |  |  |  |  |  |  |  |  |  |  |  |  |  |  |  |  |  |  |  |  |  |  |  |  |  |  |  |  |  |  |  |  |  |  |  |  |  |  |  |  |  |  |  |  |  |  |  |  |  |  |  |  |  |  |  |  |  |  |  |  |  |  |  |  |  |  |  |  |  |  |  |  |  |  |  |  |  |  |  |  |  |  |  |  |  |  |  |  |  |  |  |  |  |  |  |  |  |  |  |  |  |  |  |  |  |  |  |  |  |  |  |  |  |  |  |  |  |  |  |  |  |  |  |  |  |  |  |  |  |  |  |  |  |  |  |  |  |  |  |  |  |  |  |  |  |  |  |  |  |  |  |  |  |  |  |  |  |  |  |  |  |  |  |
| --- | --- | --- | --- | --- | --- | --- | --- | --- | --- | --- | --- | --- | --- | --- | --- | --- | --- | --- | --- | --- | --- | --- | --- | --- | --- | --- | --- | --- | --- | --- | --- | --- | --- | --- | --- | --- | --- | --- | --- | --- | --- | --- | --- | --- | --- | --- | --- | --- | --- | --- | --- | --- | --- | --- | --- | --- | --- | --- | --- | --- | --- | --- | --- | --- | --- | --- | --- | --- | --- | --- | --- | --- | --- | --- | --- | --- | --- | --- | --- | --- | --- | --- | --- | --- | --- | --- | --- | --- | --- | --- | --- | --- | --- | --- | --- | --- | --- | --- | --- | --- | --- | --- | --- | --- | --- | --- | --- | --- | --- | --- | --- | --- | --- | --- | --- | --- | --- | --- | --- | --- | --- | --- | --- | --- | --- | --- | --- | --- | --- | --- | --- | --- | --- | --- | --- | --- | --- | --- | --- | --- | --- | --- | --- | --- | --- | --- | --- | --- | --- | --- | --- | --- | --- | --- | --- | --- | --- | --- | --- | --- | --- | --- | --- | --- | --- | --- | --- | --- | --- | --- | --- | --- | --- | --- | --- | --- | --- | --- | --- | --- | --- | --- | --- | --- | --- | --- | --- | --- |
|  |  |  |  |  |  |  |  |  |  |  |  |  |  |  |  |  |  |  |  |  |  |  |  |  |  |  |  |  |  |  |  |  |  |  |  |  |  |  |  |  |  |  |  |  |  |  |  |  |  |  |  |  |  |  |  |  |  |  |  |  |  |  |  |  |  |  |  |  |  |  |  |  |  |  |  |  |  |  |  |  |  |  |  |  |  |  |  |  |  |  |  |  |  |  |  |  |  |  |  |  |  |  |  |  |  |  |  |  |  |  |  |  |  |  |  |  |  |  |  |  |  |  |  |  |  |  |  |  |  |  |  |  |  |  |  |  |  |  |  |  |  |  |  |  |  |  |  |  |  |  |  |  |  |  |  |  |  |  |  |  |  |  |  |  |  |  |  |  |  |  |  |  |  |  |  |  |  |  |  |  |  |  |  |  |  |  |  |  |
|  |  |  |  |  |  |  |  |  |  |  |  |  | \* |  |  |  |  |  |  | 2 | 9 | 2 | 0 |  |  |  |  |  |  |  |  |  | \* |  |  |  |  |  |  | 2 | 9 | 4 | 0 |  |  |  |  |  |  |  |  |  | \* |  |  |  |  |  |  | 2 | 9 | 6 | 0 |  |  |  |  |  |  |  |  |  | \* |  |  |  |  |  |  | 2 | 9 | 8 | 0 |  |  |  |  |  |  |  |  |  | \* |  |  |  |  |  |  | 3 | 0 | 0 | 0 |  |  |  |  |  |  |  |  |  | \* |  |  |  |  |  |  | 3 | 0 | 2 | 0 |  |  |  |  |  |  |  |  |  | \* |  |  |  |  |  |  | 3 | 0 | 4 | 0 |  |  |  |  |  |  |  |  |  | \* |  |  |  |  |  |  | 3 | 0 | 6 | 0 |  |  |  |  |  |  |  |  |  | \* |  |  |  |  |  |  | 3 | 0 |  |  |  |  |  |  |  |
| M | N | 9 | 5 | 6 | 5 | 2 | 0 |  | : |  | G | A | A | A | A | A | G | A | A | G | T | A | C | A | A | A | C | A | G | T | A | C | T | A | C | A | A | A | A | G | G | A | A | G | T | A | T | A | G | A | C | T | T | A | G | A | A | A | A | C | C | T | A | A | A | A | G | G | T | G | G | A | C | T | A | A | C | T | C | T | A | G | A | A | G | A | A | A | A | T | A | T | T | C | T | G | G | T | A | G | A | A | A | A | C | T | T | T | T | T | C | G | C | A | G | G | A | A | A | A | G | A | G | A | C | C | A | G | A | A | A | G | A | A | G | A | G | A | C | G | G | C | T | C | A | G | C | C | A | T | C | C | G | A | A | G | G | A | A | A | G | A | A | G | A | A | A | T | T | C | T | G | C | C | C | G | C | A | A | G | G |  | : |  | 3 | 0 | 7 | 5 |
| M | T | 0 | 1 | 2 | 7 | 3 | 2 |  | : |  | G | A | A | A | A | A | G | A | A | G | T | A | C | A | A | A | C | A | G | T | A | C | T | A | C | A | A | A | A | G | G | A | A | G | T | A | T | A | G | A | C | T | T | A | G | A | A | A | A | C | C | T | A | A | A | A | G | G | T | G | G | A | C | T | A | A | C | T | C | T | A | G | A | A | G | A | A | A | A | T | A | T | T | C | T | G | G | T | A | G | A | A | A | A | C | T | T | T | T | T | C | G | C | A | G | G | A | A | A | A | G | A | G | A | C | C | A | G | A | A | A | G | A | A | G | A | G | A | C | G | G | C | T | C | A | G | C | C | A | T | C | C | G | A | A | G | G | A | A | A | G | A | A | G | A | A | A | T | T | C | T | G | C | C | C | G | C | A | A | G | G |  | : |  | 3 | 0 | 7 | 5 |
| M | T | 0 | 1 | 2 | 7 | 3 | 4 |  | : |  | G | A | A | A | A | A | G | A | A | G | T | A | C | A | A | A | C | A | G | T | A | C | T | A | C | A | A | A | A | G | G | A | A | G | T | A | T | A | G | A | C | T | T | A | G | A | A | A | A | C | C | T | A | A | A | A | G | G | T | G | G | A | C | T | A | A | C | T | C | T | A | G | A | A | G | A | A | A | A | T | A | T | T | C | T | G | G | T | A | G | A | A | A | A | C | T | T | T | T | T | C | G | C | A | G | G | A | A | A | A | G | A | G | A | C | C | A | G | A | A | A | G | A | A | G | A | G | A | C | G | G | C | C | C | A | G | C | C | A | T | C | C | G | A | A | G | A | A | A | A | G | A | A | G | A | A | A | T | T | C | T | G | C | C | C | A | C | A | A | G | G |  | : |  | 3 | 0 | 7 | 5 |
| M | T | 0 | 2 | 7 | 0 | 0 | 6 |  | : |  | A | A | A | G | A | A | G | A | A | G | T | A | C | A | A | A | C | A | G | T | A | C | T | A | T | A | A | A | A | A | G | A | A | G | T | A | T | A | G | A | C | T | T | A | G | G | A | A | A | C | C | T | A | A | A | A | G | G | T | G | G | A | C | T | A | A | C | T | C | T | A | G | A | A | G | A | A | A | A | T | A | T | T | C | T | G | G | T | A | G | A | A | A | A | C | T | T | T | T | T | C | G | C | A | G | G | A | A | A | A | G | A | G | A | C | C | A | G | A | A | G | G | A | A | G | A | G | T | C | T | T | C | A | T | C | A | A | C | A | A | C | A | G | A | A | G | A | C | A | A | G | A | A | G | A | A | G | T | T | C | T | G | C | C | C | A | C | A | A | G | G |  | : |  | 3 | 0 | 7 | 5 |
| M | T | 0 | 2 | 7 | 0 | 0 | 7 |  | : |  | G | A | A | A | A | A | G | A | A | G | T | A | C | A | A | G | C | A | G | T | A | C | T | A | C | A | A | A | A | G | A | A | A | G | T | A | T | A | G | A | C | T | T | A | G | G | A | A | A | C | C | T | A | A | A | A | G | G | T | G | G | A | C | T | A | A | C | T | C | T | A | G | A | A | G | A | A | A | A | T | A | C | T | C | T | G | G | T | A | G | A | A | A | A | C | T | T | T | T | T | C | G | C | A | G | G | A | A | A | A | G | A | G | A | C | C | A | G | A | A | A | G | A | A | G | A | G | A | C | G | G | C | T | C | A | G | C | C | A | T | C | C | G | A | A | G | A | A | A | A | G | A | A | G | A | A | A | T | T | C | T | G | C | C | C | A | C | A | A | G | G |  | : |  | 3 | 0 | 7 | 8 |
| M | T | 0 | 3 | 6 | 0 | 5 | 3 |  | : |  | G | A | A | A | A | A | G | A | A | G | T | A | C | A | A | G | C | A | G | T | A | C | T | A | C | A | A | A | A | G | A | A | A | G | T | A | T | A | G | A | C | T | T | A | G | G | A | A | A | C | C | T | A | A | A | A | G | G | T | G | G | A | C | T | A | A | C | T | C | T | A | G | A | A | G | A | A | A | A | T | A | C | T | C | T | G | G | T | A | G | A | A | A | A | C | T | T | T | T | T | C | G | C | A | G | G | A | A | A | A | G | A | G | A | C | C | A | G | A | A | A | G | A | A | G | A | G | A | C | G | G | C | T | C | A | G | C | C | A | T | C | C | G | A | A | G | A | A | A | A | G | A | A | G | A | A | A | T | T | C | T | G | C | C | C | A | C | A | A | G | G |  | : |  | 3 | 0 | 7 | 8 |
| M | T | 0 | 3 | 6 | 0 | 5 | 4 |  | : |  | G | A | A | A | A | A | G | A | A | G | T | A | C | A | A | G | C | A | G | T | A | C | T | A | C | A | A | A | A | G | A | A | A | G | T | A | T | A | G | A | C | T | T | A | G | G | A | A | A | C | C | T | A | A | A | A | G | G | T | G | G | A | C | T | A | A | C | T | C | T | A | G | A | A | G | A | A | A | A | T | A | C | T | C | T | G | G | T | A | G | A | A | A | A | C | T | T | T | T | T | C | G | C | A | G | G | A | A | A | A | G | A | G | A | C | C | A | G | A | A | A | G | A | A | G | A | G | A | C | G | G | C | T | C | A | G | C | C | A | T | C | C | G | A | A | G | A | A | A | A | G | A | A | G | A | A | A | T | T | C | T | G | C | C | C | A | C | A | A | G | G |  | : |  | 3 | 0 | 7 | 8 |
| M | T | 0 | 3 | 6 | 0 | 5 | 5 |  | : |  | G | A | A | A | A | A | G | A | A | G | T | A | C | A | A | G | C | A | G | T | A | C | T | A | C | A | A | A | A | G | A | A | A | G | T | A | T | A | G | A | C | T | T | A | G | G | A | A | A | C | C | T | A | A | A | A | G | G | T | G | G | A | C | T | A | A | C | T | C | T | A | G | A | A | G | A | A | A | A | T | A | C | T | C | T | G | G | T | A | G | A | A | A | A | C | T | T | T | T | T | C | G | C | A | G | G | A | A | A | A | G | A | G | A | T | C | n | G | A | A | A | G | A | A | G | A | G | A | C | G | G | C | T | C | A | G | C | C | A | T | C | C | G | A | A | G | A | A | A | A | G | A | A | G | A | A | A | T | T | C | T | G | C | C | C | G | C | A | A | G | G |  | : |  | 3 | 0 | 7 | 8 |
| M | T | 0 | 3 | 6 | 0 | 5 | 6 |  | : |  | A | A | A | G | A | A | G | A | A | G | T | A | C | A | A | G | C | A | G | T | A | C | T | A | C | A | A | A | A | G | A | A | A | G | T | A | T | A | G | A | C | T | T | A | G | G | A | A | A | C | C | C | A | A | A | A | G | G | T | G | G | A | C | T | A | A | C | T | C | T | A | G | A | A | G | A | A | A | A | T | A | T | T | C | T | G | G | T | A | G | A | A | A | A | C | T | T | T | T | T | C | G | C | A | G | G | A | A | A | A | G | A | G | A | C | C | A | G | A | A | G | G | A | A | G | A | G | T | C | T | T | C | A | T | C | A | A | C | A | A | C | A | G | A | A | G | A | C | A | A | G | A | A | G | A | A | G | T | T | C | T | G | C | C | C | A | C | A | A | G | G |  | : |  | 3 | 0 | 7 | 5 |
| M | T | 0 | 3 | 6 | 0 | 5 | 7 |  | : |  | G | A | A | A | A | A | G | A | A | G | T | A | C | A | A | A | C | A | G | T | A | C | T | A | C | A | A | A | n | G | G | A | A | G | T | A | T | A | G | A | C | T | T | A | G | A | A | A | A | C | C | T | A | A | A | A | G | G | T | G | G | A | C | T | A | A | C | T | C | T | A | G | A | A | G | A | A | A | A | T | A | T | T | C | T | G | G | T | A | G | A | A | A | A | C | T | T | T | T | T | C | G | C | A | G | G | A | A | A | A | G | A | G | A | C | C | A | G | A | A | A | G | A | A | G | A | G | A | C | G | G | C | T | C | A | G | C | C | A | T | C | C | G | A | A | G | G | A | A | A | G | A | A | G | A | A | A | T | T | C | T | G | C | C | C | G | C | A | A | G | G |  | : |  | 3 | 0 | 7 | 5 |
| K | X | 2 | 4 | 9 | 7 | 3 | 8 |  | : |  | G | A | A | A | A | A | G | A | A | G | T | A | C | A | A | A | C | A | G | T | A | C | T | A | C | A | A | A | A | G | G | A | A | G | T | A | T | A | G | A | C | T | T | A | G | A | A | A | A | C | C | T | A | A | A | A | G | G | T | G | G | A | C | T | A | A | C | T | C | T | A | G | A | A | G | A | A | A | A | T | A | T | T | C | T | G | G | T | A | G | A | A | A | A | C | T | T | T | T | T | C | G | C | A | G | G | A | A | A | A | G | A | G | A | C | C | A | G | A | A | A | G | A | A | G | A | G | A | C | G | G | C | C | C | A | G | C | C | A | T | C | C | G | A | A | G | A | A | A | A | G | A | A | G | A | A | A | T | T | C | T | G | C | C | C | A | C | A | A | G | G |  | : |  | 3 | 0 | 7 | 5 |
| K | X | 2 | 4 | 9 | 7 | 3 | 7 |  | : |  | G | A | A | A | A | A | G | A | A | G | T | A | C | A | A | A | C | A | G | T | A | C | T | A | C | A | A | A | A | G | G | A | A | G | T | A | T | A | G | A | C | T | T | A | G | A | A | A | A | C | C | T | A | A | A | A | G | G | T | G | G | A | C | T | A | A | C | T | C | T | A | G | A | A | G | A | A | A | A | T | A | T | T | C | T | G | G | T | A | G | A | A | A | A | C | T | T | T | T | T | C | G | C | A | G | G | A | A | A | A | G | A | G | A | C | C | A | G | A | A | A | G | A | A | G | A | G | A | C | G | G | C | C | C | A | G | C | C | A | T | C | C | G | A | A | G | A | A | A | A | G | A | A | G | A | A | A | T | T | C | T | G | C | C | C | A | C | A | A | G | G |  | : |  | 3 | 0 | 7 | 5 |
| K | X | 2 | 4 | 9 | 7 | 3 | 6 |  | : |  | G | A | A | A | A | A | G | A | A | G | T | A | C | A | A | A | C | A | G | T | A | C | T | A | C | A | A | A | A | G | G | A | A | G | T | A | T | A | G | A | C | T | T | A | G | A | A | A | A | C | C | T | A | A | A | A | G | G | T | G | G | A | C | T | A | A | C | T | C | T | A | G | A | A | G | A | A | A | A | T | A | T | T | C | T | G | G | T | A | G | A | A | A | A | C | T | T | T | T | T | C | G | C | A | G | G | A | A | A | A | G | A | G | A | C | C | A | G | A | A | A | G | A | A | G | A | G | A | C | G | G | C | C | C | A | G | C | C | A | T | C | C | G | A | A | G | A | A | A | A | G | A | A | G | A | A | A | T | T | C | T | G | C | C | C | A | C | A | A | G | G |  | : |  | 3 | 0 | 7 | 5 |
| K | X | 2 | 4 | 9 | 7 | 3 | 5 |  | : |  | G | A | A | A | A | A | G | A | A | G | T | A | C | A | A | A | C | A | G | T | A | C | T | A | C | A | A | A | A | G | G | A | A | G | T | A | T | A | G | A | C | T | T | A | G | A | A | A | A | C | C | T | A | A | A | A | G | G | T | G | G | A | C | T | A | A | C | T | C | T | A | G | A | A | G | A | A | A | A | T | A | T | T | C | T | G | G | T | A | G | A | A | A | A | C | T | T | T | T | T | C | G | C | A | G | G | A | A | A | A | G | A | G | A | C | C | A | G | A | A | A | G | A | A | G | A | G | A | C | G | G | C | C | C | A | G | C | C | A | T | C | C | G | A | A | G | A | A | A | A | G | A | A | G | A | A | A | T | T | C | T | G | C | C | C | A | C | A | A | G | G |  | : |  | 3 | 0 | 7 | 5 |
| M | F | 1 | 9 | 7 | 9 | 1 | 6 |  | : |  | G | A | A | A | A | A | G | A | A | G | T | A | C | A | A | G | C | A | G | T | A | C | T | A | C | A | A | A | A | G | A | A | A | G | T | A | T | A | G | A | C | T | T | A | G | G | A | A | A | C | C | T | A | A | A | A | G | G | T | G | G | A | C | T | A | A | C | T | C | T | A | G | A | A | G | A | A | A | A | T | A | T | T | C | T | G | G | T | A | G | A | A | A | A | C | T | T | T | T | T | C | G | C | A | G | G | A | A | A | A | G | A | G | A | C | C | A | G | A | A | A | G | A | A | G | A | G | A | C | G | G | C | T | C | A | G | C | C | A | T | C | C | G | A | A | G | G | A | A | A | G | A | A | G | A | A | A | T | T | C | T | G | C | C | C | G | C | A | A | G | G |  | : |  | 3 | 0 | 7 | 8 |
| K | T | 2 | 5 | 0 | 6 | 3 | 2 |  | : |  | G | A | A | A | A | A | G | A | A | G | T | A | C | A | A | G | C | A | G | T | A | C | T | A | C | A | A | A | A | G | A | A | A | G | T | A | T | A | G | A | C | T | T | A | G | G | A | A | A | C | C | T | A | A | A | A | G | G | T | G | G | A | C | T | A | A | C | T | C | T | A | G | A | A | G | A | A | A | A | T | A | C | T | C | T | G | G | T | A | G | A | A | A | G | C | T | T | T | T | T | C | G | C | A | G | G | A | A | A | A | G | A | G | A | C | C | A | G | A | A | A | G | A | A | G | A | G | A | C | G | G | C | T | C | A | G | C | C | A | T | C | C | G | A | A | G | A | A | A | A | G | A | A | G | A | A | A | T | T | C | T | G | C | C | C | A | C | A | A | G | G |  | : |  | 3 | 0 | 7 | 8 |
|  |  |  |  |  |  |  |  |  |  |  | g | A | A | a | A | A | G | A | A | G | T | A | C | A | A |  | C | A | G | T | A | C | T | A | c | A | A | A | a | g |  | A | A | G | T | A | T | A | G | A | C | T | T | A | G |  | A | A | A | C | C | t | A | A | A | A | G | G | T | G | G | A | C | T | A | A | C | T | C | T | A | G | A | A | G | A | A | A | A | T | A |  | T | C | T | G | G | T | A | G | A | A | A | a | C | T | T | T | T | T | C | G | C | A | G | G | A | A | A | A | G | A | G | A | c | C | a | G | A | A | a | G | A | A | G | A | G | a | C | g | g | C |  | c | a | g | c | C | A | t | C | c | G | A | A | G |  | a | A | A | G | A | A | G | A | A | a | T | T | C | T | G | C | C | C |  | C | A | A | G | G |  |  |  |  |  |  |  |

|  |  |  |  |  |  |  |  |  |  |  |  |  |  |  |  |  |  |  |  |  |  |  |  |  |  |  |  |  |  |  |  |  |  |  |  |  |  |  |  |  |  |  |  |  |  |  |  |  |  |  |  |  |  |  |  |  |  |  |  |  |  |  |  |  |  |  |  |  |  |  |  |  |  |  |  |  |  |  |  |  |  |  |  |  |  |  |  |  |  |  |  |  |  |  |  |  |  |  |  |  |  |  |  |  |  |  |  |  |  |  |  |  |  |  |  |  |  |  |  |  |  |  |  |  |  |  |  |  |  |  |  |  |  |  |  |  |  |  |  |  |  |  |  |  |  |  |  |  |  |  |  |  |  |  |  |  |  |  |  |  |  |  |  |  |  |  |  |  |  |  |  |  |  |  |  |  |  |  |  |  |  |  |  |  |  |  |  |  |
| --- | --- | --- | --- | --- | --- | --- | --- | --- | --- | --- | --- | --- | --- | --- | --- | --- | --- | --- | --- | --- | --- | --- | --- | --- | --- | --- | --- | --- | --- | --- | --- | --- | --- | --- | --- | --- | --- | --- | --- | --- | --- | --- | --- | --- | --- | --- | --- | --- | --- | --- | --- | --- | --- | --- | --- | --- | --- | --- | --- | --- | --- | --- | --- | --- | --- | --- | --- | --- | --- | --- | --- | --- | --- | --- | --- | --- | --- | --- | --- | --- | --- | --- | --- | --- | --- | --- | --- | --- | --- | --- | --- | --- | --- | --- | --- | --- | --- | --- | --- | --- | --- | --- | --- | --- | --- | --- | --- | --- | --- | --- | --- | --- | --- | --- | --- | --- | --- | --- | --- | --- | --- | --- | --- | --- | --- | --- | --- | --- | --- | --- | --- | --- | --- | --- | --- | --- | --- | --- | --- | --- | --- | --- | --- | --- | --- | --- | --- | --- | --- | --- | --- | --- | --- | --- | --- | --- | --- | --- | --- | --- | --- | --- | --- | --- | --- | --- | --- | --- | --- | --- | --- | --- | --- | --- | --- | --- | --- | --- | --- | --- | --- | --- | --- | --- | --- | --- | --- | --- |
|  |  |  |  |  |  |  |  |  |  |  |  |  |  |  |  |  |  |  |  |  |  |  |  |  |  |  |  |  |  |  |  |  |  |  |  |  |  |  |  |  |  |  |  |  |  |  |  |  |  |  |  |  |  |  |  |  |  |  |  |  |  |  |  |  |  |  |  |  |  |  |  |  |  |  |  |  |  |  |  |  |  |  |  |  |  |  |  |  |  |  |  |  |  |  |  |  |  |  |  |  |  |  |  |  |  |  |  |  |  |  |  |  |  |  |  |  |  |  |  |  |  |  |  |  |  |  |  |  |  |  |  |  |  |  |  |  |  |  |  |  |  |  |  |  |  |  |  |  |  |  |  |  |  |  |  |  |  |  |  |  |  |  |  |  |  |  |  |  |  |  |  |  |  |  |  |  |  |  |  |  |  |  |  |  |  |  |  |  |
|  |  |  |  |  |  |  |  |  |  |  | 8 | 0 |  |  |  |  |  |  |  |  |  | \* |  |  |  |  |  |  | 3 | 1 | 0 | 0 |  |  |  |  |  |  |  |  |  | \* |  |  |  |  |  |  | 3 | 1 | 2 | 0 |  |  |  |  |  |  |  |  |  | \* |  |  |  |  |  |  | 3 | 1 | 4 | 0 |  |  |  |  |  |  |  |  |  | \* |  |  |  |  |  |  | 3 | 1 | 6 | 0 |  |  |  |  |  |  |  |  |  | \* |  |  |  |  |  |  | 3 | 1 | 8 | 0 |  |  |  |  |  |  |  |  |  | \* |  |  |  |  |  |  | 3 | 2 | 0 | 0 |  |  |  |  |  |  |  |  |  | \* |  |  |  |  |  |  | 3 | 2 | 2 | 0 |  |  |  |  |  |  |  |  |  | \* |  |  |  |  |  |  | 3 | 2 | 4 | 0 |  |  |  |  |  |  |  |  |  |  |  |  |  |  |  |  |
| M | N | 9 | 5 | 6 | 5 | 2 | 0 |  | : |  | G | A | A | A | A | C | A | A | G | C | T | G | C | A | G | A | T | G | T | T | G | G | A | T | T | T | G | T | A | A | T | G | A | G | A | T | C | G | G | A | C | A | T | T | T | T | G | C | A | A | A | A | G | A | T | T | G | C | A | G | A | A | A | T | A | A | A | A | C | T | G | C | A | A | A | T | C | A | C | A | A | T | A | A | G | G | T | A | C | T | C | G | A | G | G | A | A | C | T | C | A | A | G | A | C | C | T | T | A | C | A | A | C | T | C | G | A | A | C | C | A | G | T | C | T | T | T | G | A | C | A | C | A | A | G | C | A | G | A | C | T | G | G | A | A | G | A | A | G | A | A | G | A | A | A | T | G | T | T | C | T | G | G | G | A | A | A | T | C | A | T |  | : |  | 3 | 2 | 4 | 6 |
| M | T | 0 | 1 | 2 | 7 | 3 | 2 |  | : |  | G | A | A | A | A | C | A | A | G | T | T | G | C | A | G | A | T | G | T | T | G | G | A | T | T | T | G | T | A | A | T | G | A | A | A | T | C | G | G | A | C | A | T | T | T | C | G | C | A | A | A | A | G | A | T | T | G | C | A | G | A | A | A | T | A | A | A | T | C | T | G | C | A | A | A | T | C | A | C | A | A | T | A | A | G | G | T | A | C | T | C | G | A | G | G | A | A | C | T | C | A | A | G | A | C | C | T | T | A | C | A | A | C | T | C | G | A | A | C | C | A | G | T | C | T | T | T | G | A | C | A | C | A | A | G | C | A | G | A | C | T | G | G | A | A | G | A | A | G | A | A | G | A | A | A | T | G | T | T | C | T | G | G | G | A | A | A | T | C | A | T |  | : |  | 3 | 2 | 4 | 6 |
| M | T | 0 | 1 | 2 | 7 | 3 | 4 |  | : |  | G | A | A | A | A | C | A | A | G | C | T | G | C | A | G | A | T | G | T | T | G | G | A | T | T | T | G | T | A | A | C | G | A | A | A | T | C | G | G | A | C | A | T | T | T | C | G | C | A | A | A | A | G | A | T | T | G | C | A | G | A | A | A | T | A | A | A | T | C | T | G | C | A | A | A | T | C | A | C | A | A | T | A | A | G | G | T | A | C | T | C | G | A | G | G | A | A | C | T | C | A | A | G | A | C | C | T | T | A | C | A | A | C | T | C | G | A | A | C | C | A | G | T | C | T | T | T | G | A | C | A | C | A | A | G | C | A | G | A | C | T | G | G | A | A | G | A | A | G | A | A | G | A | A | A | T | G | T | T | C | T | G | G | G | A | A | A | T | C | A | T |  | : |  | 3 | 2 | 4 | 6 |
| M | T | 0 | 2 | 7 | 0 | 0 | 6 |  | : |  | C | A | A | A | A | C | T | A | C | A | T | G | C | A | G | G | T | G | C | T | G | G | A | T | T | T | G | T | A | A | T | G | A | A | A | T | C | G | G | C | C | A | C | T | T | T | G | C | A | A | A | A | G | A | C | T | G | C | A | G | A | A | A | C | A | A | A | T | C | T | G | C | A | G | G | A | C | A | T | A | A | C | A | G | A | A | T | A | A | T | T | G | A | A | G | A | A | C | T | T | C | A | A | A | G | T | C | T | T | C | A | A | T | T | C | G | A | A | C | C | A | G | T | C | T | T | C | A | A | T | C | T | T | G | A | C | G | A | A | C | T | C | A | A | G | G | A | A | G | A | A | G | A | A | A | T | G | T | T | C | T | G | G | G | A | A | A | T | C | A | T |  | : |  | 3 | 2 | 4 | 6 |
| M | T | 0 | 2 | 7 | 0 | 0 | 7 |  | : |  | G | A | A | A | A | C | A | A | G | C | T | G | C | A | G | A | T | G | T | T | G | G | A | T | T | T | G | T | A | A | C | G | A | A | A | T | C | G | G | A | C | A | T | T | T | C | G | C | A | A | A | A | G | A | T | T | G | C | A | G | A | A | A | T | A | A | A | T | C | T | G | C | A | A | A | T | C | A | C | A | A | T | A | A | G | G | T | A | C | T | C | G | A | G | G | A | A | C | T | C | A | A | G | A | C | C | T | T | A | C | A | A | C | T | C | G | A | A | C | C | A | G | T | C | T | T | T | G | A | C | A | C | A | A | G | C | A | G | A | C | T | G | G | A | A | G | A | A | G | A | A | G | A | A | A | T | G | T | T | C | T | G | G | G | A | A | A | T | C | A | T |  | : |  | 3 | 2 | 4 | 9 |
| M | T | 0 | 3 | 6 | 0 | 5 | 3 |  | : |  | G | A | A | A | A | C | A | A | G | C | T | G | C | A | G | A | T | G | T | T | G | G | A | T | T | T | G | T | A | A | C | G | A | A | A | T | C | G | G | A | C | A | T | T | T | C | G | C | A | A | A | A | G | A | T | T | G | C | A | G | A | A | A | C | A | A | A | T | C | T | G | C | A | A | A | T | C | A | C | A | A | T | A | A | G | G | T | A | C | T | C | G | A | A | G | A | A | C | T | C | A | A | G | A | C | C | T | T | A | C | A | A | C | T | C | G | A | A | C | C | A | G | T | C | T | T | T | G | A | C | A | C | A | A | G | C | A | G | A | C | T | G | G | A | A | G | A | A | G | A | A | G | A | A | A | T | G | T | T | C | T | G | G | G | A | A | A | T | C | A | T |  | : |  | 3 | 2 | 4 | 9 |
| M | T | 0 | 3 | 6 | 0 | 5 | 4 |  | : |  | G | A | A | A | A | C | A | A | G | C | T | G | C | A | G | A | T | G | T | T | G | G | A | T | T | T | G | T | A | A | C | G | A | A | A | T | C | G | G | A | C | A | T | T | T | C | G | C | A | A | A | A | G | A | T | T | G | C | A | G | A | A | A | C | A | A | A | T | C | T | G | C | A | A | A | T | C | A | C | A | A | T | A | A | G | G | T | A | C | T | C | G | A | A | G | A | A | C | T | C | A | A | G | A | C | C | T | T | A | C | A | A | C | T | C | G | A | A | C | C | A | G | T | C | T | T | T | G | A | C | A | C | A | A | G | C | A | G | A | C | T | G | G | A | A | G | A | A | G | A | A | G | A | A | A | T | G | T | T | C | T | G | G | G | A | A | A | T | C | A | T |  | : |  | 3 | 2 | 4 | 9 |
| M | T | 0 | 3 | 6 | 0 | 5 | 5 |  | : |  | G | A | A | A | A | C | A | A | G | C | T | G | C | A | G | A | T | G | T | T | G | G | A | T | T | T | G | T | A | A | T | G | A | G | A | T | C | G | G | A | C | A | T | T | T | T | G | C | A | A | A | A | G | A | T | T | G | C | A | G | A | A | A | T | A | A | A | A | C | T | G | C | A | A | A | T | C | A | C | A | A | T | A | A | G | G | T | A | C | T | C | G | A | G | G | A | A | C | T | C | A | A | G | A | C | C | T | T | A | C | A | A | C | T | C | G | A | A | C | C | A | G | T | C | T | T | T | G | A | C | A | C | A | A | G | C | A | G | A | C | T | G | G | A | A | G | A | A | G | A | A | G | A | A | A | T | G | T | T | C | T | G | G | G | A | A | A | T | C | A | T |  | : |  | 3 | 2 | 4 | 9 |
| M | T | 0 | 3 | 6 | 0 | 5 | 6 |  | : |  | C | G | A | A | A | C | T | A | C | A | T | G | C | A | G | G | T | G | C | T | G | G | A | T | T | T | G | T | A | A | T | G | A | A | A | T | C | G | G | C | C | A | C | T | T | T | G | C | A | A | A | A | G | A | C | T | G | C | A | G | A | A | A | C | A | A | A | T | C | T | G | C | A | A | A | T | C | A | C | A | A | T | A | A | G | G | T | A | C | T | C | G | A | A | G | A | A | C | T | C | A | A | G | A | C | C | T | T | A | C | A | A | C | T | C | G | A | A | C | C | A | G | T | C | T | T | T | G | A | C | A | C | A | A | G | C | A | G | A | C | T | G | G | A | A | G | A | A | G | A | A | G | A | A | A | T | G | T | T | C | T | G | G | G | A | A | A | T | C | A | T |  | : |  | 3 | 2 | 4 | 6 |
| M | T | 0 | 3 | 6 | 0 | 5 | 7 |  | : |  | G | A | A | A | A | C | A | A | G | C | T | G | T | A | G | A | T | G | T | T | G | G | A | T | T | T | G | T | A | A | T | G | A | A | A | T | C | G | G | A | C | A | T | T | T | C | G | C | A | A | A | A | G | A | T | T | G | C | A | G | A | A | A | T | A | A | A | T | C | T | G | C | A | A | A | T | C | A | C | A | A | T | A | A | G | G | T | A | C | T | C | G | A | A | G | A | A | C | T | C | A | A | G | A | C | C | T | T | A | C | A | A | C | T | T | G | A | A | C | C | A | G | T | C | T | T | T | G | A | C | A | C | A | A | G | C | A | G | A | C | T | G | G | A | A | G | A | A | G | A | A | G | A | A | A | T | G | T | T | C | T | G | G | G | A | A | A | T | C | A | T |  | : |  | 3 | 2 | 4 | 6 |
| K | X | 2 | 4 | 9 | 7 | 3 | 8 |  | : |  | G | A | A | A | A | C | A | A | G | C | T | G | C | A | G | A | T | G | T | T | G | G | A | T | T | T | G | T | A | A | C | G | A | A | A | T | C | G | G | A | C | A | T | T | T | C | G | C | A | A | A | A | G | A | T | T | G | C | A | G | A | A | A | T | A | A | A | T | C | T | G | C | A | A | A | T | C | A | C | A | A | T | A | A | G | G | T | A | C | T | C | G | A | G | G | A | A | C | T | C | A | A | G | A | C | C | T | T | A | C | A | A | C | T | C | G | A | A | C | C | A | G | T | C | T | T | T | G | A | C | A | C | A | A | G | C | A | G | A | C | T | G | G | A | A | G | A | A | G | A | A | G | A | A | A | T | G | T | T | C | T | G | G | G | A | A | A | T | C | A | T |  | : |  | 3 | 2 | 4 | 6 |
| K | X | 2 | 4 | 9 | 7 | 3 | 7 |  | : |  | G | A | A | A | A | C | A | A | G | C | T | G | C | A | G | A | T | G | T | T | G | G | A | T | T | T | G | T | A | A | C | G | A | A | A | T | C | G | G | A | C | A | T | T | T | C | G | C | A | A | A | A | G | A | T | T | G | C | A | G | A | A | A | T | A | A | A | T | C | T | G | C | A | A | A | T | C | A | C | A | A | T | A | A | G | G | T | A | C | T | C | G | A | G | G | A | A | C | T | C | A | A | G | A | C | C | T | T | A | C | A | A | C | T | C | G | A | A | C | C | A | G | T | C | T | T | T | G | A | C | A | C | A | A | G | C | A | G | A | C | T | G | G | A | A | G | A | A | G | A | A | G | A | A | A | T | G | T | T | C | T | G | G | G | A | A | A | T | C | A | T |  | : |  | 3 | 2 | 4 | 6 |
| K | X | 2 | 4 | 9 | 7 | 3 | 6 |  | : |  | G | A | A | A | A | C | A | A | G | C | T | G | C | A | G | A | T | G | T | T | G | G | A | T | T | T | G | T | A | A | C | G | A | A | A | T | C | G | G | A | C | A | T | T | T | C | G | C | A | A | A | A | G | A | T | T | G | C | A | G | A | A | A | T | A | A | A | T | C | T | G | C | A | A | A | T | C | A | C | A | A | T | A | A | G | G | T | A | C | T | C | G | A | G | G | A | A | C | T | C | A | A | G | A | C | C | T | T | A | C | A | A | C | T | C | G | A | A | C | C | A | G | T | C | T | T | T | G | A | C | A | C | A | A | G | C | A | G | A | C | T | G | G | A | A | G | A | A | G | A | A | G | A | A | A | T | G | T | T | C | T | G | G | G | A | A | A | T | C | A | T |  | : |  | 3 | 2 | 4 | 6 |
| K | X | 2 | 4 | 9 | 7 | 3 | 5 |  | : |  | G | A | A | A | A | C | A | A | G | C | T | G | C | A | G | A | T | G | T | T | G | G | A | T | T | T | G | T | A | A | C | G | A | A | A | T | C | G | G | A | C | A | T | T | T | C | G | C | A | A | A | A | G | A | T | T | G | C | A | G | A | A | A | T | A | A | A | T | C | T | G | C | A | A | A | T | C | A | C | A | A | T | A | A | G | G | T | A | C | T | C | G | A | G | G | A | A | C | T | C | A | A | G | A | C | C | T | T | A | C | A | A | C | T | C | G | A | A | C | C | A | G | T | C | T | T | T | G | A | C | A | C | A | A | G | C | A | G | A | C | T | G | G | A | A | G | A | A | G | A | A | G | A | A | A | T | G | T | T | C | T | G | G | G | A | A | A | T | C | A | T |  | : |  | 3 | 2 | 4 | 6 |
| M | F | 1 | 9 | 7 | 9 | 1 | 6 |  | : |  | G | A | G | A | A | C | A | A | G | C | T | G | C | A | G | A | T | G | T | T | G | G | A | T | T | T | G | T | A | A | T | G | A | G | A | T | C | G | G | A | C | A | T | T | T | T | G | C | A | A | A | A | G | A | T | T | G | C | A | G | A | A | A | T | A | A | A | A | C | T | G | C | A | A | A | T | C | A | C | A | A | T | A | A | G | G | T | A | C | T | C | G | A | G | G | A | A | C | T | C | A | A | G | A | C | C | T | T | A | C | A | A | C | T | C | G | A | A | C | C | A | G | T | C | T | T | T | G | A | C | A | C | A | A | G | C | A | G | A | C | T | G | G | A | A | G | A | A | G | A | A | G | A | A | A | T | G | T | T | C | T | G | G | G | A | A | A | T | C | A | T |  | : |  | 3 | 2 | 4 | 9 |
| K | T | 2 | 5 | 0 | 6 | 3 | 2 |  | : |  | G | A | A | A | A | C | A | A | G | C | T | G | C | A | G | A | T | G | T | T | G | G | A | T | T | T | G | T | A | A | C | G | A | A | A | T | C | G | G | A | C | A | T | T | T | C | G | C | A | A | A | A | G | A | T | T | G | C | A | G | A | A | A | C | A | A | A | T | C | T | G | C | A | A | A | T | C | A | C | A | A | T | A | A | G | G | T | A | C | T | C | G | A | A | G | A | A | C | T | C | A | A | G | A | C | C | T | T | A | C | A | A | C | T | C | G | A | A | C | C | A | G | T | C | T | T | T | G | A | C | A | C | A | A | G | C | A | G | A | C | T | G | G | A | A | G | A | A | G | A | A | G | A | A | A | T | G | T | T | C | T | G | G | G | A | A | A | T | C | A | T |  | : |  | 3 | 2 | 4 | 9 |
|  |  |  |  |  |  |  |  |  |  |  | g | a | a | A | A | C | a | A | g | c | T | G | c | A | G | a | T | G | t | T | G | G | A | T | T | T | G | T | A | A |  | G | A | a | A | T | C | G | G | a | C | A | t | T | T |  | G | C | A | A | A | A | G | A | t | T | G | C | A | G | A | A | A |  | A | A | A | t | C | T | G | C | A | a | a | t | C | A | c | A | A | t | A | a | g | g | T | A | c | T | c | G | A |  | G | A | A | C | T | c | a | A | g | A | c | c | t | T | a | C | A | A | c | T | c | G | A | A | C | C | A | G | T | C | T | T | t | g | A | c | a | c | a | a | g | C | a | g | A | C | T | g | g | A | a | G | A | A | G | A | A | G | A | A | A | T | G | T | T | C | T | G | G | G | A | A | A | T | C | A | T |  |  |  |  |  |  |  |

|  |  |  |  |  |  |  |  |  |  |  |  |  |  |  |  |  |  |  |  |  |  |  |  |  |  |  |  |  |  |  |  |  |  |  |  |  |  |  |  |  |  |  |  |  |  |  |  |  |  |  |  |  |  |  |  |  |  |  |  |  |  |  |  |  |  |  |  |  |  |  |  |  |  |  |  |  |  |  |  |  |  |  |  |  |  |  |  |  |  |  |  |  |  |  |  |  |  |  |  |  |  |  |  |  |  |  |  |  |  |  |  |  |  |  |  |  |  |  |  |  |  |  |  |  |  |  |  |  |  |  |  |  |  |  |  |  |  |  |  |  |  |  |  |  |  |  |  |  |  |  |  |  |  |  |  |  |  |  |  |  |  |  |  |  |  |  |  |  |  |  |  |  |  |  |  |  |  |  |  |  |  |  |  |  |  |  |  |  |
| --- | --- | --- | --- | --- | --- | --- | --- | --- | --- | --- | --- | --- | --- | --- | --- | --- | --- | --- | --- | --- | --- | --- | --- | --- | --- | --- | --- | --- | --- | --- | --- | --- | --- | --- | --- | --- | --- | --- | --- | --- | --- | --- | --- | --- | --- | --- | --- | --- | --- | --- | --- | --- | --- | --- | --- | --- | --- | --- | --- | --- | --- | --- | --- | --- | --- | --- | --- | --- | --- | --- | --- | --- | --- | --- | --- | --- | --- | --- | --- | --- | --- | --- | --- | --- | --- | --- | --- | --- | --- | --- | --- | --- | --- | --- | --- | --- | --- | --- | --- | --- | --- | --- | --- | --- | --- | --- | --- | --- | --- | --- | --- | --- | --- | --- | --- | --- | --- | --- | --- | --- | --- | --- | --- | --- | --- | --- | --- | --- | --- | --- | --- | --- | --- | --- | --- | --- | --- | --- | --- | --- | --- | --- | --- | --- | --- | --- | --- | --- | --- | --- | --- | --- | --- | --- | --- | --- | --- | --- | --- | --- | --- | --- | --- | --- | --- | --- | --- | --- | --- | --- | --- | --- | --- | --- | --- | --- | --- | --- | --- | --- | --- | --- | --- | --- | --- | --- | --- | --- |
|  |  |  |  |  |  |  |  |  |  |  |  |  |  |  |  |  |  |  |  |  |  |  |  |  |  |  |  |  |  |  |  |  |  |  |  |  |  |  |  |  |  |  |  |  |  |  |  |  |  |  |  |  |  |  |  |  |  |  |  |  |  |  |  |  |  |  |  |  |  |  |  |  |  |  |  |  |  |  |  |  |  |  |  |  |  |  |  |  |  |  |  |  |  |  |  |  |  |  |  |  |  |  |  |  |  |  |  |  |  |  |  |  |  |  |  |  |  |  |  |  |  |  |  |  |  |  |  |  |  |  |  |  |  |  |  |  |  |  |  |  |  |  |  |  |  |  |  |  |  |  |  |  |  |  |  |  |  |  |  |  |  |  |  |  |  |  |  |  |  |  |  |  |  |  |  |  |  |  |  |  |  |  |  |  |  |  |  |  |
|  |  |  |  |  |  |  |  |  |  |  | \* |  |  |  |  |  |  | 3 | 2 | 6 | 0 |  |  |  |  |  |  |  |  |  | \* |  |  |  |  |  |  | 3 | 2 | 8 | 0 |  |  |  |  |  |  |  |  |  | \* |  |  |  |  |  |  | 3 | 3 | 0 | 0 |  |  |  |  |  |  |  |  |  | \* |  |  |  |  |  |  | 3 | 3 | 2 | 0 |  |  |  |  |  |  |  |  |  | \* |  |  |  |  |  |  | 3 | 3 | 4 | 0 |  |  |  |  |  |  |  |  |  | \* |  |  |  |  |  |  | 3 | 3 | 6 | 0 |  |  |  |  |  |  |  |  |  | \* |  |  |  |  |  |  | 3 | 3 | 8 | 0 |  |  |  |  |  |  |  |  |  | \* |  |  |  |  |  |  | 3 | 4 | 0 | 0 |  |  |  |  |  |  |  |  |  | \* |  |  |  |  |  |  | 3 | 4 | 2 | 0 |  |  |  |  |  |  |  |
| M | N | 9 | 5 | 6 | 5 | 2 | 0 |  | : |  | C | T | C | A | G | A | A | A | C | A | G | A | C | T | C | A | G | A | G | T | C | A | G | A | A | T | C | A | G | A | A | T | C | G | T | C | A | T | C | A | G | A | T | G | A | A | T | C | A | G | A | C | G | A | C | T | C | A | A | C | A | G | A | T | C | T | G | G | A | A | T | G | A | A | C | T | T | C | T | A | G | A | T | A | C | T | G | A | A | G | A | A | G | G | A | T | G | G | G | A | T | C | T | T | C | T | C | T | T | A | C | A | A | G | A | A | G | A | T | T | A | C | A | G | T | C | C | A | G | A | G | C | T | T | G | A | A | G | C | C | T | T | C | G | C | A | G | A | A | G | C | C | A | A | G | C | T | T | C | A | A | C | A | A | A | T | G | G | A | G | C |  | : |  | 3 | 4 | 1 | 7 |
| M | T | 0 | 1 | 2 | 7 | 3 | 2 |  | : |  | C | T | C | A | G | A | A | A | C | A | G | A | C | T | C | A | G | A | G | T | C | A | G | A | A | T | C | A | G | A | A | T | C | G | T | C | A | T | C | A | G | A | T | G | A | A | T | C | A | G | A | C | G | A | C | T | C | A | A | C | A | G | A | T | C | T | G | G | A | A | T | A | A | A | C | T | T | C | T | A | G | A | T | A | C | T | G | A | A | G | A | A | G | G | A | T | G | G | G | A | T | C | T | T | C | T | C | T | T | A | C | A | A | G | A | A | G | A | T | T | A | C | A | G | T | C | C | A | G | A | G | C | T | T | G | A | A | G | C | C | T | T | C | G | C | A | G | A | A | G | C | C | A | A | G | C | T | T | C | A | A | C | A | A | A | T | G | G | A | G | C |  | : |  | 3 | 4 | 1 | 7 |
| M | T | 0 | 1 | 2 | 7 | 3 | 4 |  | : |  | C | T | C | A | G | A | A | A | C | A | G | A | C | T | C | A | G | A | G | T | C | A | G | A | A | T | C | A | G | A | G | T | C | G | T | C | A | T | C | A | G | A | T | G | A | A | T | C | A | G | A | C | G | A | C | T | C | A | A | C | A | G | A | T | C | T | G | G | A | A | T | G | A | A | C | T | T | C | T | A | G | A | T | A | C | T | G | A | A | G | A | A | G | G | A | T | G | G | G | A | T | C | T | T | C | T | C | T | T | A | C | A | A | G | A | A | G | A | T | T | A | C | A | A | T | C | C | A | G | A | G | C | T | T | G | A | A | G | C | C | T | T | C | G | C | A | G | A | A | G | C | A | A | A | G | C | T | T | C | A | A | C | A | A | A | T | G | G | A | G | C |  | : |  | 3 | 4 | 1 | 7 |
| M | T | 0 | 2 | 7 | 0 | 0 | 6 |  | : |  | C | T | C | A | G | A | A | A | C | A | G | A | C | T | C | A | G | A | G | T | C | A | G | A | A | T | C | A | G | A | A | T | C | G | T | C | A | T | C | A | G | A | T | G | A | A | T | C | A | G | A | C | G | A | C | T | C | A | A | C | A | G | A | T | C | T | G | G | A | A | T | A | A | A | C | T | T | C | T | A | G | A | T | A | C | T | G | A | A | G | A | A | G | G | A | T | G | G | G | A | T | C | T | T | C | T | C | T | T | A | C | A | A | G | A | A | G | A | T | T | A | C | A | G | T | C | C | A | G | A | G | C | T | T | G | A | A | G | C | C | T | T | C | G | C | A | G | A | A | G | C | C | A | A | G | C | T | T | C | A | A | C | A | A | A | T | G | G | A | G | C |  | : |  | 3 | 4 | 1 | 7 |
| M | T | 0 | 2 | 7 | 0 | 0 | 7 |  | : |  | C | T | C | A | G | A | A | A | C | A | G | A | C | T | C | A | G | A | G | T | C | A | G | A | A | T | C | A | G | A | A | T | C | G | T | C | A | T | C | A | G | A | T | G | A | A | T | C | A | G | A | C | G | A | C | T | C | A | A | C | A | G | A | T | C | T | G | G | A | A | T | G | A | A | C | T | T | C | T | A | G | A | T | A | C | T | G | A | A | G | A | A | G | G | A | T | G | G | G | A | T | C | T | T | C | T | C | T | T | A | C | A | A | G | A | A | G | A | T | T | A | C | A | G | T | C | C | A | G | A | G | C | T | T | G | A | A | G | C | C | T | T | C | G | C | A | G | A | A | G | C | C | A | A | G | C | T | T | C | A | A | C | A | A | A | T | G | G | A | G | C |  | : |  | 3 | 4 | 2 | 0 |
| M | T | 0 | 3 | 6 | 0 | 5 | 3 |  | : |  | C | T | C | A | G | A | A | A | C | A | G | A | C | T | C | A | G | A | G | T | C | A | G | A | A | T | C | A | G | A | A | T | C | G | T | C | A | T | C | A | G | A | T | G | A | A | T | C | A | G | A | C | G | A | C | T | C | A | A | C | A | G | A | T | C | T | G | G | A | A | T | G | A | A | C | T | T | C | T | A | G | A | T | A | C | T | G | A | A | G | A | A | G | G | A | T | G | G | G | A | T | C | T | T | C | T | C | T | T | A | C | A | A | G | A | A | G | A | T | T | A | C | A | G | T | C | C | A | G | A | G | C | T | T | G | A | A | G | C | C | T | T | C | G | C | A | G | A | A | G | C | C | A | A | G | C | T | T | C | A | A | C | A | A | A | T | G | G | A | G | C |  | : |  | 3 | 4 | 2 | 0 |
| M | T | 0 | 3 | 6 | 0 | 5 | 4 |  | : |  | C | T | C | A | G | A | A | A | C | A | G | A | C | T | C | A | G | A | G | T | C | A | G | A | A | T | C | A | G | A | A | T | C | G | T | C | A | T | C | A | G | A | T | G | A | A | T | C | A | G | A | C | G | A | C | T | C | A | A | C | A | G | A | T | C | T | G | G | A | A | T | G | A | A | C | T | T | C | T | A | G | A | T | A | C | T | G | A | A | G | A | A | G | G | A | T | G | G | G | A | T | C | T | T | C | T | C | T | T | A | C | A | A | G | A | A | G | A | T | T | A | C | A | G | T | C | C | A | G | A | G | C | T | T | G | A | A | G | C | C | T | T | C | G | C | A | G | A | A | G | C | C | A | A | G | C | T | T | C | A | A | C | A | A | A | T | G | G | A | G | C |  | : |  | 3 | 4 | 2 | 0 |
| M | T | 0 | 3 | 6 | 0 | 5 | 5 |  | : |  | C | T | C | A | G | A | A | A | C | A | G | A | C | T | C | A | G | A | G | T | C | A | G | A | A | T | C | A | G | A | A | T | C | G | T | C | A | T | C | A | G | A | T | G | A | A | T | C | A | G | A | C | G | A | C | T | C | A | A | C | A | G | A | T | C | T | G | G | A | A | T | G | A | A | C | T | T | C | T | A | G | A | T | A | C | T | G | A | A | G | A | A | G | G | A | T | G | G | G | A | T | C | T | T | C | T | C | T | T | A | C | A | A | G | A | A | G | A | T | T | A | C | A | G | T | C | C | A | G | A | G | C | T | T | G | A | A | G | C | C | T | T | C | G | C | A | G | A | A | G | C | C | A | A | G | C | T | T | C | A | A | C | A | A | A | T | G | G | A | G | C |  | : |  | 3 | 4 | 2 | 0 |
| M | T | 0 | 3 | 6 | 0 | 5 | 6 |  | : |  | C | T | C | A | G | A | A | A | C | A | G | A | C | T | C | A | G | A | G | T | C | A | G | A | A | T | C | A | G | A | A | T | C | G | T | C | A | T | C | A | G | A | T | G | A | A | T | C | A | G | A | C | G | A | C | T | C | A | A | C | A | G | A | T | C | T | G | G | A | A | T | G | A | A | C | T | T | C | T | A | G | A | T | A | C | T | G | A | A | G | A | A | G | G | A | T | G | G | G | A | T | C | T | T | C | T | C | T | T | A | C | A | A | G | A | A | G | A | T | T | A | C | A | G | T | C | C | A | G | A | G | C | T | T | G | A | A | G | C | C | T | T | C | G | C | A | G | A | A | G | C | C | A | A | G | C | T | T | C | A | A | C | A | A | A | T | G | G | A | G | C |  | : |  | 3 | 4 | 1 | 7 |
| M | T | 0 | 3 | 6 | 0 | 5 | 7 |  | : |  | C | T | C | A | G | A | A | A | C | A | G | A | C | T | C | A | G | A | G | T | C | A | G | A | A | T | C | A | G | A | A | T | C | G | T | C | A | T | C | A | G | A | T | G | A | A | T | C | A | G | A | C | G | A | C | T | C | A | A | C | A | G | A | T | C | T | G | G | A | A | T | G | A | A | C | T | T | C | T | A | G | A | T | A | C | T | G | A | A | G | A | A | G | G | A | T | G | G | G | A | T | C | T | T | C | T | C | T | T | A | C | A | A | G | A | A | G | A | T | T | A | C | A | G | T | C | C | A | G | A | G | C | T | T | G | A | A | G | C | C | T | T | C | G | C | A | G | A | A | G | C | C | A | A | G | C | T | T | C | A | A | C | A | A | A | T | G | G | A | G | C |  | : |  | 3 | 4 | 1 | 7 |
| K | X | 2 | 4 | 9 | 7 | 3 | 8 |  | : |  | C | T | C | A | G | A | A | A | C | A | G | A | C | T | C | A | G | A | G | T | C | A | G | A | A | T | C | A | G | A | A | T | C | G | T | C | A | T | C | A | G | A | T | G | A | A | T | C | A | G | A | T | G | A | C | T | C | A | A | C | A | G | A | T | C | T | G | G | A | A | T | G | A | A | C | T | T | C | T | A | G | A | T | A | C | T | G | A | A | G | A | A | G | G | A | T | G | G | G | A | T | C | T | T | C | T | C | T | T | A | C | A | A | G | A | A | G | A | T | T | A | C | A | G | T | C | C | A | G | A | G | C | T | T | G | A | A | G | C | C | T | T | C | G | C | A | G | A | A | G | C | C | A | A | G | C | T | T | C | A | A | C | A | A | A | T | G | G | A | G | C |  | : |  | 3 | 4 | 1 | 7 |
| K | X | 2 | 4 | 9 | 7 | 3 | 7 |  | : |  | C | T | C | A | G | A | A | A | C | A | G | A | C | T | C | A | G | A | G | T | C | A | G | A | A | T | C | A | G | A | A | T | C | G | T | C | A | T | C | A | G | A | T | G | A | A | T | C | A | G | A | T | G | A | C | T | C | A | A | C | A | G | A | T | C | T | G | G | A | A | T | G | A | A | C | T | T | C | T | A | G | A | T | A | C | T | G | A | A | G | A | A | G | G | A | T | G | G | G | A | T | C | T | T | C | T | C | T | T | A | C | A | A | G | A | A | G | A | T | T | A | C | A | G | T | C | C | A | G | A | G | C | T | T | G | A | A | G | C | C | T | T | C | G | C | A | G | A | A | G | C | C | A | A | G | C | T | T | C | A | A | C | A | A | A | T | G | G | A | G | C |  | : |  | 3 | 4 | 1 | 7 |
| K | X | 2 | 4 | 9 | 7 | 3 | 6 |  | : |  | C | T | C | A | G | A | A | A | C | A | G | A | C | T | C | A | G | A | G | T | C | A | G | A | A | T | C | A | G | A | A | T | C | G | T | C | A | T | C | A | G | A | T | G | A | A | T | C | A | G | A | T | G | A | C | T | C | A | A | C | A | G | A | T | C | T | G | G | A | A | T | G | A | A | C | T | T | C | T | A | G | A | T | A | C | T | G | A | A | G | A | A | G | G | A | T | G | G | G | A | T | C | T | T | C | T | C | T | T | A | C | A | A | G | A | A | G | A | T | T | A | C | A | G | T | C | C | A | G | A | G | C | T | T | G | A | A | G | C | C | T | T | C | G | C | A | G | A | A | G | C | C | A | A | G | C | T | T | C | A | A | C | A | A | A | T | G | G | A | G | C |  | : |  | 3 | 4 | 1 | 7 |
| K | X | 2 | 4 | 9 | 7 | 3 | 5 |  | : |  | C | T | C | A | G | A | A | A | C | A | G | A | C | T | C | A | G | A | G | T | C | A | G | A | A | T | C | A | G | A | A | T | C | G | T | C | A | T | C | A | G | A | T | G | A | A | T | C | A | G | A | C | G | A | C | T | C | A | A | C | A | G | A | T | C | T | G | G | A | A | T | G | A | A | C | T | T | C | T | A | G | A | T | A | C | T | G | A | A | G | A | A | G | G | A | T | G | G | G | A | T | C | T | T | C | T | C | T | T | A | C | A | A | G | A | A | G | A | T | T | A | C | A | G | T | C | C | A | G | A | G | C | T | T | G | A | A | G | C | C | T | T | C | G | C | A | G | A | A | G | C | C | A | A | G | C | T | T | C | A | A | C | A | A | A | T | G | G | A | G | C |  | : |  | 3 | 4 | 1 | 7 |
| M | F | 1 | 9 | 7 | 9 | 1 | 6 |  | : |  | C | T | C | A | G | A | A | A | C | A | G | A | C | T | C | A | G | A | G | T | C | A | G | A | A | T | C | A | G | A | A | T | C | G | T | C | A | T | C | A | G | A | T | G | A | A | T | C | A | G | A | C | G | A | C | T | C | A | A | C | A | G | A | T | C | T | G | G | A | A | T | G | A | A | C | T | T | C | T | A | G | A | T | A | C | T | G | A | A | G | A | A | G | G | A | T | G | G | G | A | T | C | T | T | C | T | C | T | T | A | C | A | A | G | A | A | G | A | T | T | A | C | A | G | T | C | C | A | G | A | G | C | T | T | G | A | A | G | C | C | T | T | C | G | C | A | G | A | A | G | C | C | A | A | G | C | T | T | C | A | A | C | A | A | A | T | G | G | A | G | C |  | : |  | 3 | 4 | 2 | 0 |
| K | T | 2 | 5 | 0 | 6 | 3 | 2 |  | : |  | C | T | C | A | G | A | A | A | C | A | G | A | C | T | C | A | G | A | G | T | C | A | G | A | A | T | C | A | G | A | A | T | C | G | T | C | A | T | C | A | G | A | T | G | A | A | T | C | A | G | A | C | G | A | C | T | C | A | A | C | A | G | A | T | C | T | G | G | A | A | T | G | A | A | C | T | T | C | T | A | G | A | T | A | C | T | G | A | A | G | A | A | G | G | A | T | G | G | G | A | T | C | T | T | C | T | C | T | T | A | C | A | A | G | A | A | G | A | T | T | A | C | A | G | T | C | C | A | G | A | G | C | T | T | G | A | A | G | C | C | T | T | C | G | C | A | G | A | A | G | C | C | A | A | G | C | T | T | C | A | A | C | A | A | A | T | G | G | A | G | C |  | : |  | 3 | 4 | 2 | 0 |
|  |  |  |  |  |  |  |  |  |  |  | C | T | C | A | G | A | A | A | C | A | G | A | C | T | C | A | G | A | G | T | C | A | G | A | A | T | C | A | G | A | a | T | C | G | T | C | A | T | C | A | G | A | T | G | A | A | T | C | A | G | A | c | G | A | C | T | C | A | A | C | A | G | A | T | C | T | G | G | A | A | T | g | A | A | C | T | T | C | T | A | G | A | T | A | C | T | G | A | A | G | A | A | G | G | A | T | G | G | G | A | T | C | T | T | C | T | C | T | T | A | C | A | A | G | A | A | G | A | T | T | A | C | A | g | T | C | C | A | G | A | G | C | T | T | G | A | A | G | C | C | T | T | C | G | C | A | G | A | A | G | C | c | A | A | G | C | T | T | C | A | A | C | A | A | A | T | G | G | A | G | C |  |  |  |  |  |  |  |

|  |  |  |  |  |  |  |  |  |  |  |  |  |  |  |  |  |  |  |  |  |  |  |  |  |  |  |  |  |  |  |  |  |  |  |  |  |  |  |  |  |  |  |  |  |  |  |  |  |  |  |  |  |  |  |  |  |  |  |  |  |  |  |  |  |  |  |  |  |  |  |  |  |  |  |  |  |  |  |  |  |  |  |  |  |  |  |  |  |  |  |  |  |  |  |  |  |  |  |  |  |  |  |  |  |  |  |  |  |  |  |  |  |  |  |  |  |  |  |  |  |  |  |  |  |  |  |  |  |  |  |  |  |  |  |  |  |  |  |  |  |  |  |  |  |  |  |  |  |  |  |  |  |  |  |  |  |  |  |  |  |  |  |  |  |  |  |  |  |  |  |  |  |  |  |  |  |  |  |  |  |  |  |  |  |  |  |  |  |
| --- | --- | --- | --- | --- | --- | --- | --- | --- | --- | --- | --- | --- | --- | --- | --- | --- | --- | --- | --- | --- | --- | --- | --- | --- | --- | --- | --- | --- | --- | --- | --- | --- | --- | --- | --- | --- | --- | --- | --- | --- | --- | --- | --- | --- | --- | --- | --- | --- | --- | --- | --- | --- | --- | --- | --- | --- | --- | --- | --- | --- | --- | --- | --- | --- | --- | --- | --- | --- | --- | --- | --- | --- | --- | --- | --- | --- | --- | --- | --- | --- | --- | --- | --- | --- | --- | --- | --- | --- | --- | --- | --- | --- | --- | --- | --- | --- | --- | --- | --- | --- | --- | --- | --- | --- | --- | --- | --- | --- | --- | --- | --- | --- | --- | --- | --- | --- | --- | --- | --- | --- | --- | --- | --- | --- | --- | --- | --- | --- | --- | --- | --- | --- | --- | --- | --- | --- | --- | --- | --- | --- | --- | --- | --- | --- | --- | --- | --- | --- | --- | --- | --- | --- | --- | --- | --- | --- | --- | --- | --- | --- | --- | --- | --- | --- | --- | --- | --- | --- | --- | --- | --- | --- | --- | --- | --- | --- | --- | --- | --- | --- | --- | --- | --- | --- | --- | --- | --- | --- |
|  |  |  |  |  |  |  |  |  |  |  |  |  |  |  |  |  |  |  |  |  |  |  |  |  |  |  |  |  |  |  |  |  |  |  |  |  |  |  |  |  |  |  |  |  |  |  |  |  |  |  |  |  |  |  |  |  |  |  |  |  |  |  |  |  |  |  |  |  |  |  |  |  |  |  |  |  |  |  |  |  |  |  |  |  |  |  |  |  |  |  |  |  |  |  |  |  |  |  |  |  |  |  |  |  |  |  |  |  |  |  |  |  |  |  |  |  |  |  |  |  |  |  |  |  |  |  |  |  |  |  |  |  |  |  |  |  |  |  |  |  |  |  |  |  |  |  |  |  |  |  |  |  |  |  |  |  |  |  |  |  |  |  |  |  |  |  |  |  |  |  |  |  |  |  |  |  |  |  |  |  |  |  |  |  |  |  |  |  |
|  |  |  |  |  |  |  |  |  |  |  |  |  |  |  |  |  |  |  |  | \* |  |  |  |  |  |  | 3 | 4 | 4 | 0 |  |  |  |  |  |  |  |  |  | \* |  |  |  |  |  |  | 3 | 4 | 6 | 0 |  |  |  |  |  |  |  |  |  | \* |  |  |  |  |  |  | 3 | 4 | 8 | 0 |  |  |  |  |  |  |  |  |  | \* |  |  |  |  |  |  | 3 | 5 | 0 | 0 |  |  |  |  |  |  |  |  |  | \* |  |  |  |  |  |  | 3 | 5 | 2 | 0 |  |  |  |  |  |  |  |  |  | \* |  |  |  |  |  |  | 3 | 5 | 4 | 0 |  |  |  |  |  |  |  |  |  | \* |  |  |  |  |  |  | 3 | 5 | 6 | 0 |  |  |  |  |  |  |  |  |  | \* |  |  |  |  |  |  | 3 | 5 | 8 | 0 |  |  |  |  |  |  |  |  |  | \* |  |  |  |  |  |  |  |  |
| M | N | 9 | 5 | 6 | 5 | 2 | 0 |  | : |  | A | A | G | A | A | C | T | C | C | A | A | C | A | A | G | A | A | G | A | A | C | T | A | C | A | A | C | A | T | C | T | T | G | A | A | G | A | A | G | G | A | T | G | G | G | A | G | T | T | C | C | T | A | A | C | A | G | A | T | G | A | A | G | A | C | T | T | C | A | A | T | C | T | C | C | A | G | C | A | A | G | A | A | G | A | T | G | A | A | G | A | A | C | A | A | C | T | T | C | T | T | G | A | T | T | C | C | G | T | C | G | A | A | T | C | A | G | A | A | G | A | A | C | G | G | T | T | T | A | G | T | A | C | T | C | T | A | A | C | C | A | A | G | A | C | T | A | A | T | C | C | A | A | A | T | A | G | C | A | T | T | T | A | T | A | T | A | A | A | A | G |  | : |  | 3 | 5 | 8 | 8 |
| M | T | 0 | 1 | 2 | 7 | 3 | 2 |  | : |  | A | A | G | A | A | C | T | C | C | A | A | C | A | A | G | A | A | G | A | A | C | T | A | C | A | A | C | A | T | C | T | T | G | A | A | G | A | A | G | G | A | T | G | G | G | A | G | T | T | C | C | T | A | A | C | A | G | A | T | G | A | A | G | A | C | T | T | C | A | A | T | C | T | C | C | A | G | C | A | A | G | A | A | G | A | T | G | A | A | G | A | A | C | A | A | C | T | T | C | T | T | G | A | T | T | C | C | G | T | C | G | A | A | T | C | A | G | A | A | G | A | A | C | G | G | T | T | T | A | G | T | A | C | T | C | T | A | A | C | C | A | A | G | A | C | T | A | A | T | C | C | A | A | A | T | A | G | C | A | T | T | T | A | T | A | T | A | A | A | A | G |  | : |  | 3 | 5 | 8 | 8 |
| M | T | 0 | 1 | 2 | 7 | 3 | 4 |  | : |  | A | A | G | A | A | C | T | C | C | A | A | C | A | A | G | A | A | G | A | A | C | T | A | C | A | A | C | A | T | C | T | T | G | A | A | G | A | A | G | G | A | T | G | G | G | A | G | T | T | C | C | T | A | A | C | A | G | A | T | G | A | A | G | A | C | T | T | C | A | A | T | C | T | C | C | A | G | C | A | A | G | A | A | G | A | T | G | A | A | G | A | A | C | A | A | C | T | T | C | T | T | G | A | T | T | C | C | G | T | C | G | A | A | T | C | A | G | A | A | G | A | A | C | G | G | T | T | T | A | G | T | A | C | T | C | T | A | A | C | C | A | A | G | A | C | T | A | A | T | C | C | A | A | A | T | A | G | C | A | T | T | T | A | T | A | T | A | A | A | A | G |  | : |  | 3 | 5 | 8 | 8 |
| M | T | 0 | 2 | 7 | 0 | 0 | 6 |  | : |  | A | A | G | A | A | C | T | C | C | A | A | C | A | A | G | A | A | G | A | A | C | T | A | C | A | A | C | A | T | C | T | T | G | A | A | G | A | A | G | G | A | T | G | G | G | A | G | T | T | C | C | T | A | A | C | A | G | A | T | G | A | A | G | A | C | T | T | C | A | A | T | C | T | C | C | A | G | C | A | A | G | A | A | G | A | T | G | A | A | G | A | A | C | A | A | C | T | T | C | T | T | G | A | T | T | C | C | G | T | C | G | A | A | T | C | A | G | A | A | G | A | A | C | G | G | T | T | T | A | G | T | A | C | T | C | T | A | A | C | C | A | A | G | A | C | T | A | A | T | C | C | A | A | A | T | A | G | C | A | T | T | T | A | T | A | T | A | A | A | A | G |  | : |  | 3 | 5 | 8 | 8 |
| M | T | 0 | 2 | 7 | 0 | 0 | 7 |  | : |  | A | A | G | A | A | C | T | C | C | A | A | C | A | A | G | A | A | G | A | A | C | T | A | C | A | A | C | A | T | C | T | T | G | A | A | G | A | A | G | G | A | T | G | G | G | A | G | T | T | C | C | T | A | A | C | A | G | A | C | G | A | A | G | A | C | T | T | C | A | A | T | C | T | C | C | A | G | C | A | A | G | A | A | G | A | T | G | A | A | G | A | A | C | A | A | C | T | T | C | T | T | G | A | T | T | C | C | G | T | C | G | A | A | T | C | A | G | A | A | G | A | A | C | G | G | T | T | T | A | G | T | A | C | T | C | T | A | A | C | C | A | A | G | A | C | T | A | A | T | C | C | A | A | A | T | A | G | C | A | T | T | T | A | T | A | T | A | A | A | A | G |  | : |  | 3 | 5 | 9 | 1 |
| M | T | 0 | 3 | 6 | 0 | 5 | 3 |  | : |  | A | A | G | A | A | C | T | C | C | A | A | C | A | A | G | A | A | G | A | A | C | T | A | C | A | A | C | A | T | C | T | T | G | A | A | G | A | A | G | G | A | T | G | G | G | A | G | T | T | C | C | T | A | A | C | A | G | A | T | G | A | A | G | A | C | T | T | C | A | A | T | C | T | C | C | A | G | C | A | A | G | A | A | G | A | T | G | A | A | G | A | A | C | A | A | C | T | T | C | T | T | G | A | T | T | C | C | G | T | C | G | A | A | T | C | A | G | A | A | G | A | A | C | G | G | T | T | T | A | G | T | A | C | T | C | T | A | A | C | C | A | A | G | A | C | T | A | A | T | C | C | A | A | A | T | A | G | C | A | T | T | T | A | T | A | T | A | A | A | A | G |  | : |  | 3 | 5 | 9 | 1 |
| M | T | 0 | 3 | 6 | 0 | 5 | 4 |  | : |  | A | A | G | A | A | C | T | C | C | A | A | C | A | A | G | A | A | G | A | A | C | T | A | C | A | A | C | A | T | C | T | T | G | A | A | G | A | A | G | G | A | T | G | G | G | A | G | T | T | C | C | T | A | A | C | A | G | A | T | G | A | A | G | A | C | T | T | C | A | A | T | C | T | C | C | A | G | C | A | A | G | A | A | G | A | T | G | A | A | G | A | A | C | A | A | C | T | T | C | T | T | G | A | T | T | C | C | G | T | C | G | A | A | T | C | A | G | A | A | G | A | A | C | G | G | T | T | T | A | G | T | A | C | T | C | T | A | A | C | C | A | A | G | A | C | T | A | A | T | C | C | A | A | A | T | A | G | C | A | T | T | T | A | T | A | T | A | A | A | A | G |  | : |  | 3 | 5 | 9 | 1 |
| M | T | 0 | 3 | 6 | 0 | 5 | 5 |  | : |  | A | A | G | A | A | C | T | C | C | A | A | C | A | A | G | A | A | G | A | A | C | T | A | C | A | A | C | A | T | C | T | T | G | A | A | G | A | A | G | G | A | T | G | G | G | A | G | T | T | C | C | T | A | A | C | A | G | A | T | G | A | A | G | A | C | T | T | C | A | A | T | C | T | C | C | A | G | C | A | A | G | A | A | G | A | T | G | A | A | G | A | A | C | A | A | C | T | T | C | T | T | G | A | T | T | C | C | G | T | C | G | A | A | T | C | A | G | A | A | G | A | A | C | G | G | T | T | T | A | G | T | A | C | T | C | T | A | A | C | C | A | A | G | A | C | T | A | A | T | C | C | A | A | A | T | A | G | C | A | T | T | T | A | T | A | T | A | A | A | A | G |  | : |  | 3 | 5 | 9 | 1 |
| M | T | 0 | 3 | 6 | 0 | 5 | 6 |  | : |  | A | A | G | G | A | C | T | C | C | A | A | C | A | A | G | A | A | G | A | A | C | T | A | C | A | A | C | A | T | C | T | T | G | A | A | G | A | A | G | G | A | T | G | G | G | A | G | T | T | C | C | T | A | A | C | A | G | A | T | G | A | A | G | A | C | T | T | C | A | A | T | C | T | C | C | A | G | C | A | A | G | A | A | G | A | T | G | A | A | G | A | A | C | A | A | C | T | T | C | T | T | G | A | T | T | C | C | G | T | C | G | A | A | T | C | A | G | A | A | G | A | A | C | G | G | T | T | T | A | G | T | A | C | T | C | T | A | A | C | C | A | A | G | A | C | T | A | A | T | C | C | A | A | A | T | A | G | C | A | T | T | T | A | T | A | T | A | A | A | A | G |  | : |  | 3 | 5 | 8 | 8 |
| M | T | 0 | 3 | 6 | 0 | 5 | 7 |  | : |  | A | A | G | A | A | C | T | C | C | A | A | C | A | A | G | A | A | G | A | A | C | T | A | C | A | A | C | A | T | C | T | T | G | A | A | G | A | A | G | G | A | T | G | G | G | A | G | T | T | C | C | T | A | A | C | A | G | A | T | G | A | A | G | A | C | T | T | C | A | A | T | C | T | C | C | A | G | C | A | A | G | A | A | G | A | T | G | A | A | G | A | A | C | A | A | C | T | T | C | T | T | G | A | T | T | C | C | G | T | C | G | A | A | T | C | A | G | A | A | G | A | A | C | G | G | T | T | T | A | G | T | A | C | T | C | T | A | A | C | C | A | A | G | A | C | T | A | A | T | C | C | A | A | A | T | A | G | C | A | T | T | T | A | T | A | T | A | A | A | A | G |  | : |  | 3 | 5 | 8 | 8 |
| K | X | 2 | 4 | 9 | 7 | 3 | 8 |  | : |  | A | A | G | A | A | C | T | C | C | A | A | C | A | A | G | A | A | G | A | A | C | T | A | C | A | A | C | A | T | C | T | T | G | A | A | G | A | A | G | G | A | T | G | G | G | A | G | T | T | C | C | T | A | A | C | A | G | A | T | G | A | A | G | A | C | T | T | C | A | A | T | C | T | C | C | A | G | C | A | A | G | A | A | G | A | T | G | A | A | G | A | A | C | A | A | C | T | T | C | T | T | G | A | T | T | C | C | G | T | C | G | A | A | T | C | A | G | A | A | G | A | A | C | G | G | T | T | T | A | G | T | A | C | T | C | T | A | A | C | C | A | A | G | A | C | T | A | A | T | C | C | A | A | A | T | A | G | C | A | T | T | T | A | T | A | T | A | A | A | A | G |  | : |  | 3 | 5 | 8 | 8 |
| K | X | 2 | 4 | 9 | 7 | 3 | 7 |  | : |  | A | A | G | A | A | C | T | C | C | A | A | C | A | A | G | A | A | G | A | A | C | T | A | C | A | A | C | A | T | C | T | T | G | A | A | G | A | A | G | G | A | T | G | G | G | A | G | T | T | C | C | T | A | A | C | A | G | A | T | G | A | A | G | A | C | T | T | C | A | A | T | C | T | C | C | A | G | C | A | A | G | A | A | G | A | T | G | A | A | G | A | A | C | A | A | C | T | T | C | T | T | G | A | T | T | C | C | G | T | C | G | A | A | T | C | A | G | A | A | G | A | A | C | G | G | T | T | T | A | G | T | A | C | T | C | T | A | A | C | C | A | A | G | A | C | T | A | A | T | C | C | A | A | A | T | A | G | C | A | T | T | T | A | T | A | T | A | A | A | A | G |  | : |  | 3 | 5 | 8 | 8 |
| K | X | 2 | 4 | 9 | 7 | 3 | 6 |  | : |  | A | A | G | A | A | C | T | C | C | A | A | C | A | A | G | A | A | G | A | A | C | T | A | C | A | A | C | A | T | C | T | T | G | A | A | G | A | A | G | G | A | T | G | G | G | A | G | T | T | C | C | T | A | A | C | A | G | A | T | G | A | A | G | A | C | T | T | C | A | A | T | C | T | C | C | A | G | C | A | A | G | A | A | G | A | T | G | A | A | G | A | A | C | A | A | C | T | T | C | T | T | G | A | T | T | C | C | G | T | C | G | A | A | T | C | A | G | A | A | G | A | A | C | G | G | T | T | T | A | G | T | A | C | T | C | T | A | A | C | C | A | A | G | A | C | T | A | A | T | C | C | A | A | A | T | A | G | C | A | T | T | T | A | T | A | T | A | A | A | A | G |  | : |  | 3 | 5 | 8 | 8 |
| K | X | 2 | 4 | 9 | 7 | 3 | 5 |  | : |  | A | A | G | A | A | C | T | C | C | A | A | C | A | A | G | A | A | G | A | A | C | T | A | C | A | A | C | A | T | C | T | T | G | A | A | G | A | A | G | G | A | T | G | G | G | A | G | T | T | C | C | T | A | A | C | A | G | A | T | G | A | A | G | A | C | T | T | C | A | A | T | C | T | C | C | A | G | C | A | A | G | A | A | G | A | T | G | A | A | G | A | A | C | A | A | C | T | T | C | T | T | G | A | T | T | C | C | G | T | C | G | A | A | T | C | A | G | A | A | G | A | A | C | G | G | T | T | T | A | G | T | A | C | T | C | T | A | A | C | C | A | A | G | A | C | T | A | A | T | C | C | A | A | A | T | A | G | C | A | T | T | T | A | T | A | T | A | A | A | A | G |  | : |  | 3 | 5 | 8 | 8 |
| M | F | 1 | 9 | 7 | 9 | 1 | 6 |  | : |  | A | A | G | A | A | C | T | T | C | A | A | C | A | A | G | A | A | G | A | A | C | T | A | C | A | A | C | A | T | C | T | T | G | A | A | G | A | A | G | G | A | T | G | G | G | A | G | T | T | C | C | T | A | A | C | A | G | A | T | G | A | A | G | A | C | T | T | C | A | A | T | C | T | C | C | A | G | C | A | A | G | A | A | G | A | T | G | A | A | G | A | A | C | A | A | C | T | T | C | T | T | G | A | T | T | C | C | G | T | C | G | A | A | T | C | A | G | A | A | G | A | A | C | G | G | T | T | T | A | G | T | A | C | T | C | T | A | A | C | C | A | A | G | A | C | T | A | A | T | C | C | A | A | A | T | A | G | C | A | T | T | T | A | T | A | T | A | A | A | A | G |  | : |  | 3 | 5 | 9 | 1 |
| K | T | 2 | 5 | 0 | 6 | 3 | 2 |  | : |  | A | A | G | A | A | C | T | C | C | A | A | C | A | A | G | A | A | G | A | A | C | T | A | C | A | A | C | A | T | C | T | T | G | A | A | G | A | A | G | G | A | T | G | G | G | A | G | T | T | C | C | T | A | A | C | A | G | A | T | G | A | A | G | A | C | T | T | C | A | A | T | C | T | C | C | A | G | C | A | A | G | A | A | G | A | T | G | A | A | G | A | A | C | A | A | C | T | T | C | T | T | G | A | T | T | C | C | G | T | C | G | A | A | T | C | A | G | A | A | G | A | A | C | G | G | T | T | T | A | G | T | A | C | T | C | T | A | A | C | C | A | A | G | A | C | T | A | A | T | C | C | A | A | A | T | A | G | C | A | T | T | T | A | T | A | T | A | A | A | A | G |  | : |  | 3 | 5 | 9 | 1 |
|  |  |  |  |  |  |  |  |  |  |  | A | A | G | a | A | C | T | c | C | A | A | C | A | A | G | A | A | G | A | A | C | T | A | C | A | A | C | A | T | C | T | T | G | A | A | G | A | A | G | G | A | T | G | G | G | A | G | T | T | C | C | T | A | A | C | A | G | A | t | G | A | A | G | A | C | T | T | C | A | A | T | C | T | C | C | A | G | C | A | A | G | A | A | G | A | T | G | A | A | G | A | A | C | A | A | C | T | T | C | T | T | G | A | T | T | C | C | G | T | C | G | A | A | T | C | A | G | A | A | G | A | A | C | G | G | T | T | T | A | G | T | A | C | T | C | T | A | A | C | C | A | A | G | A | C | T | A | A | T | C | C | A | A | A | T | A | G | C | A | T | T | T | A | T | A | T | A | A | A | A | G |  |  |  |  |  |  |  |

|  |  |  |  |  |  |  |  |  |  |  |  |  |  |  |  |  |  |  |  |  |  |  |  |  |  |  |  |  |  |  |  |  |  |  |  |  |  |  |  |  |  |  |  |  |  |  |  |  |  |  |  |  |  |  |  |  |  |  |  |  |  |  |  |  |  |  |  |  |  |  |  |  |  |  |  |  |  |  |  |  |  |  |  |  |  |  |  |  |  |  |  |  |  |  |  |  |  |  |  |  |  |  |  |  |  |  |  |  |  |  |  |  |  |  |  |  |  |  |  |  |  |  |  |  |  |  |  |  |  |  |  |  |  |  |  |  |  |  |  |  |  |  |  |  |  |  |  |  |  |  |  |  |  |  |  |  |  |  |  |  |  |  |  |  |  |  |  |  |  |  |  |  |  |  |  |  |  |  |  |  |  |  |  |  |  |  |  |  |
| --- | --- | --- | --- | --- | --- | --- | --- | --- | --- | --- | --- | --- | --- | --- | --- | --- | --- | --- | --- | --- | --- | --- | --- | --- | --- | --- | --- | --- | --- | --- | --- | --- | --- | --- | --- | --- | --- | --- | --- | --- | --- | --- | --- | --- | --- | --- | --- | --- | --- | --- | --- | --- | --- | --- | --- | --- | --- | --- | --- | --- | --- | --- | --- | --- | --- | --- | --- | --- | --- | --- | --- | --- | --- | --- | --- | --- | --- | --- | --- | --- | --- | --- | --- | --- | --- | --- | --- | --- | --- | --- | --- | --- | --- | --- | --- | --- | --- | --- | --- | --- | --- | --- | --- | --- | --- | --- | --- | --- | --- | --- | --- | --- | --- | --- | --- | --- | --- | --- | --- | --- | --- | --- | --- | --- | --- | --- | --- | --- | --- | --- | --- | --- | --- | --- | --- | --- | --- | --- | --- | --- | --- | --- | --- | --- | --- | --- | --- | --- | --- | --- | --- | --- | --- | --- | --- | --- | --- | --- | --- | --- | --- | --- | --- | --- | --- | --- | --- | --- | --- | --- | --- | --- | --- | --- | --- | --- | --- | --- | --- | --- | --- | --- | --- | --- | --- | --- | --- | --- |
|  |  |  |  |  |  |  |  |  |  |  |  |  |  |  |  |  |  |  |  |  |  |  |  |  |  |  |  |  |  |  |  |  |  |  |  |  |  |  |  |  |  |  |  |  |  |  |  |  |  |  |  |  |  |  |  |  |  |  |  |  |  |  |  |  |  |  |  |  |  |  |  |  |  |  |  |  |  |  |  |  |  |  |  |  |  |  |  |  |  |  |  |  |  |  |  |  |  |  |  |  |  |  |  |  |  |  |  |  |  |  |  |  |  |  |  |  |  |  |  |  |  |  |  |  |  |  |  |  |  |  |  |  |  |  |  |  |  |  |  |  |  |  |  |  |  |  |  |  |  |  |  |  |  |  |  |  |  |  |  |  |  |  |  |  |  |  |  |  |  |  |  |  |  |  |  |  |  |  |  |  |  |  |  |  |  |  |  |  |
|  |  |  |  |  |  |  |  |  |  |  |  |  |  |  |  | 3 | 6 | 0 | 0 |  |  |  |  |  |  |  |  |  | \* |  |  |  |  |  |  | 3 | 6 | 2 | 0 |  |  |  |  |  |  |  |  |  | \* |  |  |  |  |  |  | 3 | 6 | 4 | 0 |  |  |  |  |  |  |  |  |  | \* |  |  |  |  |  |  | 3 | 6 | 6 | 0 |  |  |  |  |  |  |  |  |  | \* |  |  |  |  |  |  | 3 | 6 | 8 | 0 |  |  |  |  |  |  |  |  |  | \* |  |  |  |  |  |  | 3 | 7 | 0 | 0 |  |  |  |  |  |  |  |  |  | \* |  |  |  |  |  |  | 3 | 7 | 2 | 0 |  |  |  |  |  |  |  |  |  | \* |  |  |  |  |  |  | 3 | 7 | 4 | 0 |  |  |  |  |  |  |  |  |  | \* |  |  |  |  |  |  | 3 | 7 | 6 | 0 |  |  |  |  |  |  |  |  |  |
| M | N | 9 | 5 | 6 | 5 | 2 | 0 |  | : |  | G | T | A | A | C | T | T | T | T | A | T | T | T | T | A | A | A | G | G | C | T | A | T | A | A | A | A | A | G | T | A | C | T | C | A | C | T | C | G | A | T | C | T | T | T | A | T | G | T | T | G | A | T | A | C | A | G | G | T | G | C | G | A | G | T | A | T | G | T | G | T | A | C | A | G | C | A | A | A | C | A | A | G | C | A | T | G | T | A | A | T | T | C | C | A | G | A | A | G | A | A | T | T | C | T | G | G | G | T | T | A | A | T | G | C | T | A | A | A | A | A | T | C | C | T | A | T | C | A | A | A | G | C | T | C | G | C | A | T | A | G | C | T | A | A | T | G | A | T | A | G | T | A | T | T | A | T | G | A | C | C | T | T | T | A | A | C | A | A | G | G |  | : |  | 3 | 7 | 5 | 9 |
| M | T | 0 | 1 | 2 | 7 | 3 | 2 |  | : |  | G | T | A | A | C | T | T | T | T | A | T | T | T | T | A | A | A | G | G | C | T | A | T | A | A | A | A | A | G | T | A | C | T | C | A | C | T | C | G | A | T | C | T | T | T | A | T | G | T | T | G | A | T | A | C | A | G | G | T | G | C | G | A | G | T | A | T | G | T | G | T | A | C | A | G | C | A | A | A | C | A | A | G | C | A | T | G | T | A | A | T | T | C | C | A | G | A | A | G | A | A | T | T | C | T | G | G | G | T | T | A | A | T | G | C | T | A | A | A | A | A | T | C | C | T | A | T | C | A | A | A | G | C | T | C | G | C | A | T | A | G | C | T | A | A | T | G | A | T | A | G | T | A | T | T | A | T | G | A | C | C | T | T | T | A | A | C | A | A | G | G |  | : |  | 3 | 7 | 5 | 9 |
| M | T | 0 | 1 | 2 | 7 | 3 | 4 |  | : |  | G | T | A | A | C | T | T | T | T | A | T | T | T | T | A | A | A | G | G | C | T | A | T | A | A | A | A | A | G | T | A | C | T | C | A | C | T | C | G | A | T | C | T | T | T | A | T | G | T | T | G | A | T | A | C | A | G | G | T | G | C | G | A | G | T | A | T | G | T | G | T | A | C | A | G | C | A | A | A | C | A | A | G | C | A | T | G | T | A | A | T | T | C | C | A | G | A | A | G | A | A | T | T | C | T | G | G | G | T | T | A | A | T | G | C | T | A | A | A | A | A | T | C | C | T | A | T | C | A | A | A | G | C | T | C | G | C | A | T | A | G | C | T | A | A | T | G | A | T | A | G | T | A | T | T | A | T | G | A | C | C | T | T | T | A | A | C | A | A | G | G |  | : |  | 3 | 7 | 5 | 9 |
| M | T | 0 | 2 | 7 | 0 | 0 | 6 |  | : |  | G | T | A | A | C | T | T | T | T | A | T | T | T | T | A | A | A | G | G | C | T | A | T | A | A | A | A | A | G | T | A | C | T | C | A | C | T | C | G | A | T | C | T | T | T | A | T | G | T | T | G | A | T | A | C | A | G | G | T | G | C | G | A | G | T | A | T | G | T | G | T | A | C | A | G | C | A | A | A | C | A | A | G | C | A | T | G | T | A | A | T | T | C | C | A | G | A | A | G | A | A | T | T | C | T | G | G | G | T | T | A | A | T | G | C | T | A | A | A | A | A | T | C | C | T | A | T | C | A | A | A | G | C | T | C | G | C | A | T | A | G | C | T | A | A | T | G | A | T | A | G | T | A | T | T | A | T | G | A | C | C | T | T | T | A | A | C | A | A | G | G |  | : |  | 3 | 7 | 5 | 9 |
| M | T | 0 | 2 | 7 | 0 | 0 | 7 |  | : |  | G | T | A | A | C | T | T | T | T | A | T | T | T | T | A | A | A | G | G | C | T | A | T | A | A | A | A | A | G | T | A | C | T | C | A | C | T | C | G | A | T | C | T | T | T | A | T | G | T | T | G | A | T | A | C | A | G | G | T | G | C | G | A | G | T | A | T | G | T | G | T | A | C | A | G | C | A | A | A | C | A | A | G | C | A | T | G | T | A | A | T | T | C | C | A | G | A | A | G | A | A | T | T | C | T | G | G | G | T | T | A | A | T | G | C | T | A | A | A | A | A | T | C | C | T | A | T | C | A | A | A | G | C | T | C | G | C | A | T | A | G | C | T | A | A | T | G | A | T | A | G | T | A | T | T | A | T | G | A | C | C | T | T | T | A | A | C | A | A | G | G |  | : |  | 3 | 7 | 6 | 2 |
| M | T | 0 | 3 | 6 | 0 | 5 | 3 |  | : |  | G | T | A | A | C | T | T | T | T | A | T | T | T | T | A | A | A | G | G | C | T | A | T | A | A | A | A | A | G | T | A | C | T | C | A | C | T | C | G | A | T | C | T | T | T | A | T | G | T | T | G | A | T | A | C | T | G | G | T | G | C | G | A | G | T | A | T | G | T | G | T | A | C | A | G | C | A | A | A | C | A | A | G | C | A | T | G | T | A | A | T | T | C | C | A | G | A | A | G | A | A | T | T | C | T | G | G | G | T | T | A | A | T | G | C | T | A | A | A | A | A | T | C | C | T | A | T | C | A | A | A | G | C | T | C | G | C | A | T | A | G | C | T | A | A | T | G | A | T | A | G | T | A | T | T | A | T | G | A | C | C | T | T | T | A | A | C | A | A | G | G |  | : |  | 3 | 7 | 6 | 2 |
| M | T | 0 | 3 | 6 | 0 | 5 | 4 |  | : |  | G | T | A | A | C | T | T | T | T | A | T | T | T | T | A | A | A | G | G | C | T | A | T | A | A | A | A | A | G | T | A | C | T | C | A | C | T | C | G | A | T | C | T | T | T | A | T | G | T | T | G | A | T | A | C | A | G | G | T | G | C | G | A | G | T | A | T | G | T | G | T | A | C | A | G | C | A | A | A | C | A | A | G | C | A | T | G | T | A | A | T | T | C | C | A | G | A | A | G | A | A | T | T | C | T | G | G | G | T | T | A | A | T | G | C | T | A | A | A | A | A | T | C | C | T | A | T | C | A | A | A | G | C | T | C | G | C | A | T | A | G | C | T | A | A | T | G | A | T | A | G | T | A | T | T | A | T | G | A | C | C | T | T | T | A | A | C | A | A | G | G |  | : |  | 3 | 7 | 6 | 2 |
| M | T | 0 | 3 | 6 | 0 | 5 | 5 |  | : |  | G | T | A | A | C | T | T | T | T | A | T | T | T | T | A | A | A | G | G | C | T | A | T | A | A | A | A | A | G | T | A | C | T | C | A | C | T | C | G | A | T | C | T | T | T | A | T | G | T | T | G | A | T | A | C | A | G | G | T | G | C | G | A | G | T | A | T | G | T | G | T | A | C | A | G | C | A | A | A | C | A | A | G | C | A | T | G | T | A | A | T | T | C | C | A | G | A | A | G | A | A | T | T | C | T | G | G | G | T | T | A | A | T | G | C | T | A | A | A | A | A | T | C | C | T | A | T | C | A | A | A | G | C | T | C | G | C | A | T | A | G | C | T | A | A | T | G | A | T | A | G | T | A | T | T | A | T | G | A | C | C | T | T | T | A | A | C | A | A | G | G |  | : |  | 3 | 7 | 6 | 2 |
| M | T | 0 | 3 | 6 | 0 | 5 | 6 |  | : |  | G | T | A | A | C | T | T | T | T | A | T | T | T | T | A | A | A | G | G | C | T | A | T | A | A | A | A | A | G | T | A | C | T | C | A | C | T | C | G | A | T | C | T | T | T | A | T | G | T | T | G | A | T | A | C | A | G | G | T | G | C | G | A | G | T | A | T | G | T | G | T | A | C | A | G | C | A | A | A | C | A | A | G | C | A | T | G | T | A | A | T | T | C | C | A | G | A | A | G | A | A | T | T | C | T | G | G | G | T | T | A | A | T | G | C | T | A | A | A | A | A | T | C | C | T | A | T | C | A | A | A | G | C | T | C | G | C | A | T | A | G | C | T | A | A | T | G | A | T | A | G | T | A | T | T | A | T | G | A | C | C | T | T | T | A | A | C | A | A | G | G |  | : |  | 3 | 7 | 5 | 9 |
| M | T | 0 | 3 | 6 | 0 | 5 | 7 |  | : |  | G | T | A | A | C | T | T | T | T | A | T | T | T | T | A | A | A | G | G | C | T | A | T | A | A | A | A | A | G | T | A | C | T | C | A | C | T | C | G | A | T | C | T | T | T | A | T | G | T | T | G | A | T | A | C | A | G | G | T | G | C | G | A | G | T | A | T | G | T | G | T | A | C | A | G | C | A | A | A | C | A | A | G | C | A | T | G | T | A | A | T | T | C | C | A | G | A | A | G | A | A | T | T | C | T | G | G | G | T | T | A | A | T | G | C | T | A | A | A | A | A | T | C | C | T | A | T | C | A | A | A | G | C | T | C | G | C | A | T | A | G | C | T | A | A | T | G | A | T | A | G | T | A | T | T | A | T | G | A | C | C | T | T | T | A | A | C | A | A | G | G |  | : |  | 3 | 7 | 5 | 9 |
| K | X | 2 | 4 | 9 | 7 | 3 | 8 |  | : |  | G | T | A | A | C | T | T | T | T | A | T | T | T | T | A | A | A | G | G | C | T | A | T | A | A | A | A | A | G | T | A | C | T | C | A | C | T | C | G | A | T | C | T | T | T | A | T | G | T | T | G | A | T | A | C | A | G | G | T | G | C | G | A | G | T | A | T | G | T | G | T | A | C | A | G | C | A | A | A | C | A | A | G | C | A | T | G | T | A | A | T | T | C | C | A | G | A | A | G | A | A | T | T | C | T | G | G | G | T | T | A | A | T | G | C | T | A | A | A | A | A | T | C | C | T | A | T | C | A | A | A | G | C | T | C | G | C | A | T | A | G | C | T | A | A | T | G | A | T | A | G | T | A | T | T | A | T | G | A | C | C | T | T | T | A | A | C | A | A | G | G |  | : |  | 3 | 7 | 5 | 9 |
| K | X | 2 | 4 | 9 | 7 | 3 | 7 |  | : |  | G | T | A | A | C | T | T | T | T | A | T | T | T | T | A | A | A | G | G | C | T | A | T | A | A | A | A | A | G | T | A | C | T | C | A | C | T | C | G | A | T | C | T | T | T | A | T | G | T | T | G | A | T | A | C | A | G | G | T | G | C | G | A | G | T | A | T | G | T | G | T | A | C | A | G | C | A | A | A | C | A | A | G | C | A | T | G | T | A | A | T | T | C | C | A | G | A | A | G | A | A | T | T | C | T | G | G | G | T | T | A | A | T | G | C | T | A | A | A | A | A | T | C | C | T | A | T | C | A | A | A | G | C | T | C | G | C | A | T | A | G | C | T | A | A | T | G | A | T | A | G | T | A | T | T | A | T | G | A | C | C | T | T | T | A | A | C | A | A | G | G |  | : |  | 3 | 7 | 5 | 9 |
| K | X | 2 | 4 | 9 | 7 | 3 | 6 |  | : |  | G | T | A | A | C | T | T | T | T | A | T | T | T | T | A | A | A | G | G | C | T | A | T | A | A | A | A | A | G | T | A | C | T | C | A | C | T | C | G | A | T | C | T | T | T | A | T | G | T | T | G | A | T | A | C | A | G | G | T | G | C | G | A | G | T | A | T | G | T | G | T | A | C | A | G | C | A | A | A | C | A | A | G | C | A | T | G | T | A | A | T | T | C | C | A | G | A | A | G | A | A | T | T | C | T | G | G | G | T | T | A | A | T | G | C | T | A | A | A | A | A | T | C | C | T | A | T | C | A | A | A | G | C | T | C | G | C | A | T | A | G | C | T | A | A | T | G | A | T | A | G | T | A | T | T | A | T | G | A | C | C | T | T | T | A | A | C | A | A | G | G |  | : |  | 3 | 7 | 5 | 9 |
| K | X | 2 | 4 | 9 | 7 | 3 | 5 |  | : |  | G | T | A | A | C | T | T | T | T | A | T | T | T | T | A | A | A | G | G | C | T | A | T | A | A | A | A | A | G | T | A | C | T | C | A | C | T | C | G | A | T | C | T | T | T | A | T | G | T | T | G | A | T | A | C | A | G | G | T | G | C | G | A | G | T | A | T | G | T | G | T | A | C | A | G | C | A | A | A | C | A | A | G | C | A | T | G | T | A | A | T | T | C | C | A | G | A | A | G | A | A | T | T | C | T | G | G | G | T | T | A | A | T | G | C | T | A | A | A | A | A | T | C | C | T | A | T | C | A | A | A | G | C | T | C | G | C | A | T | A | G | C | T | A | A | T | G | A | T | A | G | T | A | T | T | A | T | G | A | C | C | T | T | T | A | A | C | A | A | G | G |  | : |  | 3 | 7 | 5 | 9 |
| M | F | 1 | 9 | 7 | 9 | 1 | 6 |  | : |  | G | T | A | A | C | T | T | T | T | A | T | T | T | T | A | A | A | G | G | C | T | A | T | A | A | A | A | A | G | T | A | C | T | C | A | C | T | C | G | A | T | C | T | T | T | A | T | G | T | T | G | A | T | A | C | A | G | G | T | G | C | G | A | G | T | A | T | G | T | G | T | A | C | A | G | C | A | A | A | C | A | A | G | C | A | T | G | T | A | A | T | T | C | C | A | G | A | A | G | A | A | T | T | C | T | G | G | G | T | T | A | A | T | G | C | T | A | A | A | A | A | T | C | C | T | A | T | C | A | A | A | G | C | T | C | G | C | A | T | A | G | C | T | A | A | T | G | A | T | A | G | T | A | T | T | A | T | G | A | C | C | T | T | T | A | A | C | A | A | G | G |  | : |  | 3 | 7 | 6 | 2 |
| K | T | 2 | 5 | 0 | 6 | 3 | 2 |  | : |  | G | T | A | A | C | T | T | T | T | A | T | T | T | T | A | A | A | G | G | C | T | A | T | A | A | A | A | A | G | T | A | C | T | C | A | C | T | C | G | A | T | C | T | T | T | A | T | G | T | T | G | A | T | A | C | A | G | G | T | G | C | G | A | G | T | A | T | G | T | G | T | A | C | A | G | C | A | A | A | C | A | A | G | C | A | T | G | T | A | A | T | T | C | C | A | G | A | A | G | A | A | T | T | C | T | G | G | G | T | T | A | A | T | G | C | T | A | A | A | A | A | T | C | C | T | A | T | C | A | A | A | G | C | T | C | G | C | A | T | A | G | C | T | A | A | T | G | A | T | A | G | T | A | T | T | A | T | G | A | C | C | T | T | T | A | A | C | A | A | G | G |  | : |  | 3 | 7 | 6 | 2 |
|  |  |  |  |  |  |  |  |  |  |  | G | T | A | A | C | T | T | T | T | A | T | T | T | T | A | A | A | G | G | C | T | A | T | A | A | A | A | A | G | T | A | C | T | C | A | C | T | C | G | A | T | C | T | T | T | A | T | G | T | T | G | A | T | A | C | a | G | G | T | G | C | G | A | G | T | A | T | G | T | G | T | A | C | A | G | C | A | A | A | C | A | A | G | C | A | T | G | T | A | A | T | T | C | C | A | G | A | A | G | A | A | T | T | C | T | G | G | G | T | T | A | A | T | G | C | T | A | A | A | A | A | T | C | C | T | A | T | C | A | A | A | G | C | T | C | G | C | A | T | A | G | C | T | A | A | T | G | A | T | A | G | T | A | T | T | A | T | G | A | C | C | T | T | T | A | A | C | A | A | G | G |  |  |  |  |  |  |  |

|  |  |  |  |  |  |  |  |  |  |  |  |  |  |  |  |  |  |  |  |  |  |  |  |  |  |  |  |  |  |  |  |  |  |  |  |  |  |  |  |  |  |  |  |  |  |  |  |  |  |  |  |  |  |  |  |  |  |  |  |  |  |  |  |  |  |  |  |  |  |  |  |  |  |  |  |  |  |  |  |  |  |  |  |  |  |  |  |  |  |  |  |  |  |  |  |  |  |  |  |  |  |  |  |  |  |  |  |  |  |  |  |  |  |  |  |  |  |  |  |  |  |  |  |  |  |  |  |  |  |  |  |  |  |  |  |  |  |  |  |  |  |  |  |  |  |  |  |  |  |  |  |  |  |  |  |  |  |  |  |  |  |  |  |  |  |  |  |  |  |  |  |  |  |  |  |  |  |  |  |  |  |  |  |  |  |  |  |  |
| --- | --- | --- | --- | --- | --- | --- | --- | --- | --- | --- | --- | --- | --- | --- | --- | --- | --- | --- | --- | --- | --- | --- | --- | --- | --- | --- | --- | --- | --- | --- | --- | --- | --- | --- | --- | --- | --- | --- | --- | --- | --- | --- | --- | --- | --- | --- | --- | --- | --- | --- | --- | --- | --- | --- | --- | --- | --- | --- | --- | --- | --- | --- | --- | --- | --- | --- | --- | --- | --- | --- | --- | --- | --- | --- | --- | --- | --- | --- | --- | --- | --- | --- | --- | --- | --- | --- | --- | --- | --- | --- | --- | --- | --- | --- | --- | --- | --- | --- | --- | --- | --- | --- | --- | --- | --- | --- | --- | --- | --- | --- | --- | --- | --- | --- | --- | --- | --- | --- | --- | --- | --- | --- | --- | --- | --- | --- | --- | --- | --- | --- | --- | --- | --- | --- | --- | --- | --- | --- | --- | --- | --- | --- | --- | --- | --- | --- | --- | --- | --- | --- | --- | --- | --- | --- | --- | --- | --- | --- | --- | --- | --- | --- | --- | --- | --- | --- | --- | --- | --- | --- | --- | --- | --- | --- | --- | --- | --- | --- | --- | --- | --- | --- | --- | --- | --- | --- | --- | --- |
|  |  |  |  |  |  |  |  |  |  |  |  |  |  |  |  |  |  |  |  |  |  |  |  |  |  |  |  |  |  |  |  |  |  |  |  |  |  |  |  |  |  |  |  |  |  |  |  |  |  |  |  |  |  |  |  |  |  |  |  |  |  |  |  |  |  |  |  |  |  |  |  |  |  |  |  |  |  |  |  |  |  |  |  |  |  |  |  |  |  |  |  |  |  |  |  |  |  |  |  |  |  |  |  |  |  |  |  |  |  |  |  |  |  |  |  |  |  |  |  |  |  |  |  |  |  |  |  |  |  |  |  |  |  |  |  |  |  |  |  |  |  |  |  |  |  |  |  |  |  |  |  |  |  |  |  |  |  |  |  |  |  |  |  |  |  |  |  |  |  |  |  |  |  |  |  |  |  |  |  |  |  |  |  |  |  |  |  |  |
|  |  |  |  |  |  |  |  |  |  |  |  |  |  |  |  |  |  | \* |  |  |  |  |  |  | 3 | 7 | 8 | 0 |  |  |  |  |  |  |  |  |  | \* |  |  |  |  |  |  | 3 | 8 | 0 | 0 |  |  |  |  |  |  |  |  |  | \* |  |  |  |  |  |  | 3 | 8 | 2 | 0 |  |  |  |  |  |  |  |  |  | \* |  |  |  |  |  |  | 3 | 8 | 4 | 0 |  |  |  |  |  |  |  |  |  | \* |  |  |  |  |  |  | 3 | 8 | 6 | 0 |  |  |  |  |  |  |  |  |  | \* |  |  |  |  |  |  | 3 | 8 | 8 | 0 |  |  |  |  |  |  |  |  |  | \* |  |  |  |  |  |  | 3 | 9 | 0 | 0 |  |  |  |  |  |  |  |  |  | \* |  |  |  |  |  |  | 3 | 9 | 2 | 0 |  |  |  |  |  |  |  |  |  | \* |  |  |  |  |  |  |  |  |  |  |
| M | N | 9 | 5 | 6 | 5 | 2 | 0 |  | : |  | T | T | G | C | A | G | A | A | T | T | A | G | T | G | C | A | G | G | T | A | C | A | A | A | T | T | G | C | A | G | A | T | G | A | A | A | C | A | T | T | C | A | T | C | A | T | A | C | C | A | A | C | C | A | T | G | T | A | C | C | A | A | G | C | A | A | C | A | A | C | A | A | A | A | G | G | A | G | A | C | A | T | T | A | C | C | A | T | C | G | G | T | A | A | T | A | A | C | T | T | C | T | G | T | C | G | T | T | T | G | T | A | T | G | A | A | C | C | A | T | T | T | G | T | T | C | A | G | T | A | C | A | A | A | G | A | C | A | T | G | A | T | T | A | C | C | T | T | T | C | A | C | A | A | G | A | A | T | G | G | C | A | A | A | G | C | C | A | T | C | A |  | : |  | 3 | 9 | 3 | 0 |
| M | T | 0 | 1 | 2 | 7 | 3 | 2 |  | : |  | T | T | G | C | A | G | A | A | T | T | A | G | T | G | C | A | G | G | T | A | C | A | A | A | T | T | G | C | A | G | A | T | G | A | A | A | C | A | T | T | C | A | T | C | A | T | A | C | C | A | A | C | C | A | T | G | T | A | C | C | A | A | G | C | A | A | C | A | A | C | A | A | A | A | G | G | A | G | A | C | A | T | T | A | C | C | A | T | C | G | G | T | A | A | T | A | A | C | T | T | C | T | G | T | C | G | T | T | T | G | T | A | T | G | A | A | C | C | A | T | T | T | G | T | T | C | A | G | T | A | C | A | A | A | G | A | C | A | T | G | A | T | T | A | C | C | T | T | T | C | A | C | A | A | G | A | A | T | G | G | C | A | A | A | G | C | C | A | T | C | A |  | : |  | 3 | 9 | 3 | 0 |
| M | T | 0 | 1 | 2 | 7 | 3 | 4 |  | : |  | T | T | G | C | A | G | A | A | T | T | A | G | T | G | C | A | G | G | T | A | C | A | A | A | T | T | G | C | A | G | A | T | G | A | A | A | C | A | T | T | C | A | T | C | A | T | A | C | C | A | A | C | C | A | T | G | T | A | C | C | A | A | G | C | A | A | C | A | A | C | A | A | A | A | G | G | A | G | A | C | A | T | T | A | C | C | A | T | C | G | G | T | A | A | T | A | A | C | T | T | C | T | G | T | C | G | T | T | T | G | T | A | T | G | A | A | C | C | A | T | T | T | G | T | T | C | A | G | T | A | C | A | A | A | G | A | C | A | T | G | A | T | T | A | C | C | T | T | T | C | A | C | A | A | G | A | A | T | G | G | C | A | A | A | G | C | C | A | T | C | A |  | : |  | 3 | 9 | 3 | 0 |
| M | T | 0 | 2 | 7 | 0 | 0 | 6 |  | : |  | T | T | G | C | A | G | A | A | T | T | A | G | T | G | C | A | G | G | T | A | C | A | A | A | T | T | G | C | A | G | A | T | G | A | A | A | C | A | T | T | C | A | T | C | A | T | A | C | C | A | A | C | C | A | T | G | T | A | C | C | A | A | G | C | A | A | C | A | A | C | A | A | A | A | G | G | A | G | A | C | A | T | C | A | C | C | A | T | C | G | G | T | A | A | T | A | A | C | T | T | C | T | G | T | C | G | T | T | T | G | T | A | T | G | A | A | C | C | A | T | T | T | G | T | T | C | A | G | T | A | C | A | A | A | G | A | C | A | T | G | A | T | T | A | C | C | T | T | T | C | A | C | A | A | G | A | A | T | G | G | C | A | A | A | G | C | C | A | T | C | A |  | : |  | 3 | 9 | 3 | 0 |
| M | T | 0 | 2 | 7 | 0 | 0 | 7 |  | : |  | T | T | G | C | A | G | A | A | T | T | A | G | T | G | C | A | G | G | T | A | C | A | A | A | T | T | G | C | A | G | A | T | G | A | A | A | C | A | T | T | C | A | T | C | A | T | A | C | C | A | A | C | C | A | T | G | T | A | C | C | A | A | G | C | A | A | C | A | A | C | A | A | A | A | G | G | A | G | A | C | A | T | T | A | C | C | A | T | C | G | G | T | A | A | T | A | A | C | T | T | C | T | G | T | C | G | T | T | T | G | T | A | T | G | A | A | C | C | A | T | T | T | G | T | T | C | A | G | T | A | C | A | A | A | G | A | C | A | T | G | A | T | T | A | C | C | T | T | T | C | A | C | A | A | G | A | A | T | G | G | C | A | A | A | G | C | C | A | T | C | A |  | : |  | 3 | 9 | 3 | 3 |
| M | T | 0 | 3 | 6 | 0 | 5 | 3 |  | : |  | T | T | G | C | A | G | A | A | T | T | A | G | T | G | C | A | G | G | T | A | C | A | A | A | T | T | G | C | A | G | A | T | G | A | A | A | C | A | T | T | C | A | T | C | A | T | A | C | C | A | A | C | C | A | T | G | T | A | C | C | A | A | G | C | A | A | C | A | A | C | A | A | A | A | G | G | A | G | A | C | A | T | T | A | C | C | A | T | C | G | G | T | A | A | T | A | A | C | T | T | C | T | G | T | C | G | T | T | T | G | T | A | T | G | A | A | C | C | A | T | T | T | G | T | T | C | A | G | T | A | C | A | A | A | G | A | C | A | T | G | A | T | T | A | C | C | T | T | T | C | A | C | A | A | G | A | A | T | G | G | C | A | A | A | G | C | C | A | T | C | A |  | : |  | 3 | 9 | 3 | 3 |
| M | T | 0 | 3 | 6 | 0 | 5 | 4 |  | : |  | T | T | G | C | A | G | A | A | T | T | A | G | T | G | C | A | G | G | T | A | C | A | A | A | T | T | G | C | A | G | A | T | G | A | A | A | C | A | T | T | C | A | T | C | A | T | A | C | C | A | A | C | C | A | T | G | T | A | C | C | A | A | G | C | A | A | C | A | A | C | A | A | A | A | G | G | A | G | A | C | A | T | T | A | C | C | A | T | C | G | G | T | A | A | T | A | A | C | T | T | C | T | G | T | C | G | T | T | T | G | T | A | T | G | A | A | C | C | A | T | T | T | G | T | T | C | A | G | T | A | C | A | A | A | G | A | C | A | T | G | A | T | T | A | C | C | T | T | T | C | A | C | A | A | G | A | A | T | G | G | C | A | A | A | G | C | C | A | T | C | A |  | : |  | 3 | 9 | 3 | 3 |
| M | T | 0 | 3 | 6 | 0 | 5 | 5 |  | : |  | T | T | G | C | A | G | A | A | T | T | A | G | T | G | C | A | G | G | T | A | C | A | A | A | T | T | G | C | A | G | A | T | G | A | A | A | C | A | T | T | C | A | T | C | A | T | A | C | C | A | A | C | C | A | T | G | T | A | C | C | A | A | G | C | A | A | C | A | A | C | A | A | A | A | G | G | A | G | A | C | A | T | T | A | C | C | A | T | C | G | G | T | A | A | T | A | A | C | T | T | C | T | G | T | C | G | T | T | T | G | T | A | T | G | A | A | C | C | A | T | T | T | G | T | C | C | A | G | T | A | C | A | A | A | G | A | C | A | T | G | A | T | T | A | C | C | T | T | T | C | A | C | A | A | G | A | A | T | G | G | C | A | A | A | G | C | C | A | T | C | A |  | : |  | 3 | 9 | 3 | 3 |
| M | T | 0 | 3 | 6 | 0 | 5 | 6 |  | : |  | T | T | G | C | A | G | A | A | T | T | A | G | T | G | C | A | G | G | T | A | C | A | A | A | T | T | G | C | A | G | A | T | G | A | A | A | C | A | T | T | C | A | T | C | A | T | A | C | C | A | A | C | C | A | T | G | T | A | C | C | A | G | G | C | A | A | C | A | A | C | A | A | A | A | G | G | A | G | A | C | A | T | T | A | C | C | A | T | C | G | G | T | A | A | T | A | A | C | T | T | C | T | G | T | C | G | T | T | T | G | T | A | T | G | A | A | C | C | A | T | T | T | G | T | T | C | A | G | T | A | C | A | A | A | G | A | C | A | T | G | A | T | T | A | C | C | T | T | T | C | A | C | A | A | G | A | A | T | G | G | C | A | A | A | G | C | C | A | T | C | A |  | : |  | 3 | 9 | 3 | 0 |
| M | T | 0 | 3 | 6 | 0 | 5 | 7 |  | : |  | T | T | G | C | A | G | A | A | T | T | A | G | T | G | C | A | G | G | T | A | C | A | A | A | T | T | G | C | A | G | A | T | G | A | A | A | C | A | T | T | C | A | T | C | A | T | A | C | C | A | A | C | C | A | T | G | T | A | C | C | A | A | G | C | A | A | C | A | A | C | A | A | A | A | G | G | A | G | A | C | A | T | T | A | C | C | A | T | C | G | G | T | A | A | T | A | A | C | T | T | C | T | G | T | C | G | T | T | T | G | T | A | T | G | A | A | C | C | A | T | T | T | G | T | T | C | A | G | T | A | C | A | A | A | G | A | C | A | T | G | A | T | T | A | C | C | T | T | T | C | A | C | A | A | G | A | A | T | G | G | C | A | A | A | G | C | C | A | T | C | A |  | : |  | 3 | 9 | 3 | 0 |
| K | X | 2 | 4 | 9 | 7 | 3 | 8 |  | : |  | T | T | G | C | A | G | A | A | T | T | A | G | T | G | C | A | G | G | T | A | C | A | A | A | T | T | G | C | A | G | A | T | G | A | A | A | C | A | T | T | C | A | T | C | A | T | A | C | C | A | A | C | C | A | T | G | T | A | C | C | A | A | G | C | A | A | C | A | A | C | A | A | A | A | G | G | A | G | A | C | A | T | T | A | C | C | A | T | C | G | G | T | A | A | T | A | A | C | T | T | C | T | G | T | C | G | T | T | T | G | T | A | T | G | A | A | C | C | A | T | T | T | G | T | T | C | A | G | T | A | C | A | A | A | G | A | C | A | T | G | A | T | T | A | C | C | T | T | T | C | A | C | A | A | G | A | A | T | G | G | C | A | A | A | G | C | C | A | T | C | A |  | : |  | 3 | 9 | 3 | 0 |
| K | X | 2 | 4 | 9 | 7 | 3 | 7 |  | : |  | T | T | G | C | A | G | A | A | T | T | A | G | T | G | C | A | G | G | T | A | C | A | A | A | T | T | G | C | A | G | A | T | G | A | A | A | C | A | T | T | C | A | T | C | A | T | A | C | C | A | A | C | C | A | T | G | T | A | C | C | A | A | G | C | A | A | C | A | A | C | A | A | A | A | G | G | A | G | A | C | A | T | T | A | C | C | A | T | C | G | G | T | A | A | T | A | A | C | T | T | C | T | G | T | C | G | T | T | T | G | T | A | T | G | A | A | C | C | A | T | T | T | G | T | T | C | A | G | T | A | C | A | A | A | G | A | C | A | T | G | A | T | T | A | C | C | T | T | T | C | A | C | A | A | G | A | A | T | G | G | C | A | A | A | G | C | C | A | T | C | A |  | : |  | 3 | 9 | 3 | 0 |
| K | X | 2 | 4 | 9 | 7 | 3 | 6 |  | : |  | T | T | G | C | A | G | A | A | T | T | A | G | T | G | C | A | G | G | T | A | C | A | A | A | T | T | G | C | A | G | A | T | G | A | A | A | C | A | T | T | C | A | T | C | A | T | A | C | C | A | A | C | C | A | T | G | T | A | C | C | A | A | G | C | A | A | C | A | A | C | A | A | A | A | G | G | A | G | A | C | A | T | T | A | C | C | A | T | C | G | G | T | A | A | T | A | A | C | T | T | C | T | G | T | C | G | T | T | T | G | T | A | T | G | A | A | C | C | A | T | T | T | G | T | T | C | A | G | T | A | C | A | A | A | G | A | C | A | T | G | A | T | T | A | C | C | T | T | T | C | A | C | A | A | G | A | A | T | G | G | C | A | A | A | G | C | C | A | T | C | A |  | : |  | 3 | 9 | 3 | 0 |
| K | X | 2 | 4 | 9 | 7 | 3 | 5 |  | : |  | T | T | G | C | A | G | A | A | T | T | A | G | T | G | C | A | G | G | T | A | C | A | A | A | T | T | G | C | A | G | A | T | G | A | A | A | C | A | T | T | C | A | T | C | A | T | A | C | C | A | A | C | C | A | T | G | T | A | C | C | A | A | G | C | A | A | C | A | A | C | A | A | A | A | G | G | A | G | A | C | A | T | T | A | C | C | A | T | C | G | G | T | A | A | T | A | A | C | T | T | C | T | G | T | C | G | T | T | T | G | T | A | T | G | A | A | C | C | A | T | T | T | G | T | T | C | A | G | T | A | C | A | A | A | G | A | C | A | T | G | A | T | T | A | C | C | T | T | T | C | A | C | A | A | G | A | A | T | G | G | C | A | A | A | G | C | C | A | T | C | A |  | : |  | 3 | 9 | 3 | 0 |
| M | F | 1 | 9 | 7 | 9 | 1 | 6 |  | : |  | T | T | G | C | A | G | A | A | T | T | A | G | T | G | C | A | G | G | T | A | C | A | A | A | T | T | G | C | A | G | A | T | G | A | A | A | C | A | T | T | T | A | T | C | A | T | A | C | C | A | A | C | C | A | T | G | T | A | C | C | A | A | G | C | A | A | C | A | A | C | A | A | A | A | G | G | A | G | A | C | A | T | T | A | C | C | A | T | C | G | G | T | A | A | T | A | A | C | T | T | C | T | G | T | C | G | T | T | T | G | T | A | T | G | A | A | C | C | A | T | T | T | G | T | T | C | A | G | T | A | C | A | A | A | G | A | C | A | T | G | A | T | T | A | C | C | T | T | T | C | A | C | A | A | G | A | A | T | G | G | C | A | A | A | G | C | C | A | T | C | A |  | : |  | 3 | 9 | 3 | 3 |
| K | T | 2 | 5 | 0 | 6 | 3 | 2 |  | : |  | T | T | G | C | A | G | A | A | T | T | A | G | T | G | C | A | G | G | T | A | C | A | A | A | T | T | G | C | A | G | A | T | G | A | A | A | C | A | T | T | C | A | T | C | A | T | A | C | C | A | A | C | C | A | T | G | T | A | C | C | A | A | G | C | A | A | C | A | A | C | A | A | A | A | G | G | A | G | A | C | A | T | T | A | C | C | A | T | C | G | G | T | A | A | T | A | A | C | T | T | C | T | G | T | C | G | T | T | T | G | T | A | T | G | A | A | C | C | A | T | T | T | G | T | T | C | A | G | T | A | C | A | A | A | G | A | C | A | T | G | A | T | T | A | C | C | T | T | T | C | A | C | A | A | G | A | A | T | G | G | C | A | A | A | G | C | C | A | T | C | A |  | : |  | 3 | 9 | 3 | 3 |
|  |  |  |  |  |  |  |  |  |  |  | T | T | G | C | A | G | A | A | T | T | A | G | T | G | C | A | G | G | T | A | C | A | A | A | T | T | G | C | A | G | A | T | G | A | A | A | C | A | T | T | c | A | T | C | A | T | A | C | C | A | A | C | C | A | T | G | T | A | C | C | A | a | G | C | A | A | C | A | A | C | A | A | A | A | G | G | A | G | A | C | A | T | t | A | C | C | A | T | C | G | G | T | A | A | T | A | A | C | T | T | C | T | G | T | C | G | T | T | T | G | T | A | T | G | A | A | C | C | A | T | T | T | G | T | t | C | A | G | T | A | C | A | A | A | G | A | C | A | T | G | A | T | T | A | C | C | T | T | T | C | A | C | A | A | G | A | A | T | G | G | C | A | A | A | G | C | C | A | T | C | A |  |  |  |  |  |  |  |

|  |  |  |  |  |  |  |  |  |  |  |  |  |  |  |  |  |  |  |  |  |  |  |  |  |  |  |  |  |  |  |  |  |  |  |  |  |  |  |  |  |  |  |  |  |  |  |  |  |  |  |  |  |  |  |  |  |  |  |  |  |  |  |  |  |  |  |  |  |  |  |  |  |  |  |  |  |  |  |  |  |  |  |  |  |  |  |  |  |  |  |  |  |  |  |  |  |  |  |  |  |  |  |  |  |  |  |  |  |  |  |  |  |  |  |  |  |  |  |  |  |  |  |  |  |  |  |  |  |  |  |  |  |  |  |  |  |  |  |  |  |  |  |  |  |  |  |  |  |  |  |  |  |  |  |  |  |  |  |  |  |  |  |  |  |  |  |  |  |  |  |  |  |  |  |  |  |  |  |  |  |  |  |  |  |  |  |  |  |
| --- | --- | --- | --- | --- | --- | --- | --- | --- | --- | --- | --- | --- | --- | --- | --- | --- | --- | --- | --- | --- | --- | --- | --- | --- | --- | --- | --- | --- | --- | --- | --- | --- | --- | --- | --- | --- | --- | --- | --- | --- | --- | --- | --- | --- | --- | --- | --- | --- | --- | --- | --- | --- | --- | --- | --- | --- | --- | --- | --- | --- | --- | --- | --- | --- | --- | --- | --- | --- | --- | --- | --- | --- | --- | --- | --- | --- | --- | --- | --- | --- | --- | --- | --- | --- | --- | --- | --- | --- | --- | --- | --- | --- | --- | --- | --- | --- | --- | --- | --- | --- | --- | --- | --- | --- | --- | --- | --- | --- | --- | --- | --- | --- | --- | --- | --- | --- | --- | --- | --- | --- | --- | --- | --- | --- | --- | --- | --- | --- | --- | --- | --- | --- | --- | --- | --- | --- | --- | --- | --- | --- | --- | --- | --- | --- | --- | --- | --- | --- | --- | --- | --- | --- | --- | --- | --- | --- | --- | --- | --- | --- | --- | --- | --- | --- | --- | --- | --- | --- | --- | --- | --- | --- | --- | --- | --- | --- | --- | --- | --- | --- | --- | --- | --- | --- | --- | --- | --- | --- |
|  |  |  |  |  |  |  |  |  |  |  |  |  |  |  |  |  |  |  |  |  |  |  |  |  |  |  |  |  |  |  |  |  |  |  |  |  |  |  |  |  |  |  |  |  |  |  |  |  |  |  |  |  |  |  |  |  |  |  |  |  |  |  |  |  |  |  |  |  |  |  |  |  |  |  |  |  |  |  |  |  |  |  |  |  |  |  |  |  |  |  |  |  |  |  |  |  |  |  |  |  |  |  |  |  |  |  |  |  |  |  |  |  |  |  |  |  |  |  |  |  |  |  |  |  |  |  |  |  |  |  |  |  |  |  |  |  |  |  |  |  |  |  |  |  |  |  |  |  |  |  |  |  |  |  |  |  |  |  |  |  |  |  |  |  |  |  |  |  |  |  |  |  |  |  |  |  |  |  |  |  |  |  |  |  |  |  |  |  |
|  |  |  |  |  |  |  |  |  |  |  |  |  |  | 3 | 9 | 4 | 0 |  |  |  |  |  |  |  |  |  | \* |  |  |  |  |  |  | 3 | 9 | 6 | 0 |  |  |  |  |  |  |  |  |  | \* |  |  |  |  |  |  | 3 | 9 | 8 | 0 |  |  |  |  |  |  |  |  |  | \* |  |  |  |  |  |  | 4 | 0 | 0 | 0 |  |  |  |  |  |  |  |  |  | \* |  |  |  |  |  |  | 4 | 0 | 2 | 0 |  |  |  |  |  |  |  |  |  | \* |  |  |  |  |  |  | 4 | 0 | 4 | 0 |  |  |  |  |  |  |  |  |  | \* |  |  |  |  |  |  | 4 | 0 | 6 | 0 |  |  |  |  |  |  |  |  |  | \* |  |  |  |  |  |  | 4 | 0 | 8 | 0 |  |  |  |  |  |  |  |  |  | \* |  |  |  |  |  |  | 4 | 1 | 0 | 0 |  |  |  |  |  |  |  |  |  |  |  |
| M | N | 9 | 5 | 6 | 5 | 2 | 0 |  | : |  | G | C | A | C | C | A | A | A | A | A | G | G | T | A | A | C | A | A | A | A | G | C | T | T | A | T | T | T | T | C | A | T | G | G | A | C | A | T | C | C | A | G | G | A | T | T | C | C | T | T | G | A | A | A | G | T | A | A | G | A | A | A | A | T | A | G | G | A | A | G | T | A | A | C | A | T | T | C | C | T | A | C | A | C | C | A | A | A | C | C | C | T | G | A | A | A | A | T | A | T | T | A | C | C | C | C | A | G | T | G | A | C | C | A | T | T | C | A | C | C | A | G | G | A | T | A | A | T | A | T | T | T | C | C | A | C | A | A | T | T | T | T | T | A | A | G | G | G | G | G | A | G | G | A | G | A | T | T | G | G | G | G | A | A | A | C | A | C | T | T | T |  | : |  | 4 | 1 | 0 | 1 |
| M | T | 0 | 1 | 2 | 7 | 3 | 2 |  | : |  | G | C | A | C | C | A | A | A | A | A | G | G | T | A | A | C | A | A | A | A | G | C | T | T | A | T | T | T | T | C | A | T | G | G | A | C | A | T | C | C | A | G | G | A | T | T | C | C | T | T | G | A | A | A | G | T | A | A | G | A | A | A | A | T | A | G | G | A | A | G | T | A | A | C | A | T | T | C | C | T | A | C | A | C | C | A | A | A | C | C | C | T | G | A | A | A | A | T | A | T | T | A | C | C | C | C | A | G | T | G | A | C | C | A | T | T | C | A | C | C | A | G | G | A | T | A | A | T | A | T | T | T | C | C | A | C | A | A | T | T | T | T | T | A | A | G | G | G | G | G | A | G | G | A | G | A | T | T | G | G | G | G | A | A | A | C | A | C | T | T | T |  | : |  | 4 | 1 | 0 | 1 |
| M | T | 0 | 1 | 2 | 7 | 3 | 4 |  | : |  | G | C | A | C | C | A | A | A | A | A | G | G | T | A | A | C | A | A | A | A | G | C | T | T | A | T | T | T | T | C | A | T | G | G | A | C | A | T | C | C | A | G | G | A | T | T | C | C | T | T | G | A | A | A | G | T | A | A | G | A | A | A | A | T | A | G | G | A | A | G | T | A | A | C | A | T | T | C | C | T | A | C | A | C | C | A | A | A | C | C | C | T | G | A | A | A | A | T | A | T | T | A | C | C | C | C | A | G | T | G | A | C | C | A | T | T | C | A | C | C | A | G | G | A | T | A | A | T | A | T | T | T | C | C | A | C | A | A | T | T | T | T | T | A | A | G | G | G | G | G | A | G | G | A | G | A | T | T | G | G | G | G | A | A | A | C | A | C | T | T | T |  | : |  | 4 | 1 | 0 | 1 |
| M | T | 0 | 2 | 7 | 0 | 0 | 6 |  | : |  | G | C | A | C | C | A | A | A | A | A | G | G | T | A | A | C | A | A | A | A | G | C | T | T | A | T | T | T | T | C | A | T | G | G | A | C | A | T | C | C | A | G | G | A | T | T | C | C | T | T | G | A | A | A | G | T | A | A | G | A | A | A | A | T | A | G | G | A | A | G | T | A | A | C | A | T | T | C | C | T | A | C | A | C | C | A | A | A | C | C | C | T | G | A | A | A | A | T | A | T | T | G | C | C | C | C | A | G | T | G | A | C | C | A | T | T | C | A | C | C | A | G | G | A | T | A | A | T | A | T | T | T | C | C | A | C | A | A | T | T | T | T | T | A | A | G | G | G | G | G | A | G | G | A | G | A | T | T | G | G | G | G | A | A | A | C | A | C | T | T | T |  | : |  | 4 | 1 | 0 | 1 |
| M | T | 0 | 2 | 7 | 0 | 0 | 7 |  | : |  | G | C | A | C | C | A | A | A | A | A | G | G | T | A | A | C | A | A | A | A | G | C | T | T | A | T | T | T | T | C | A | T | G | G | A | C | A | T | C | C | A | G | G | A | T | T | C | C | T | T | G | A | A | A | G | T | A | A | G | A | A | A | A | T | A | G | G | A | A | G | T | A | A | C | A | T | T | C | C | T | A | C | A | C | C | A | A | A | C | C | C | T | G | A | A | A | A | T | A | T | T | A | C | C | C | C | A | G | T | G | A | C | C | A | T | T | C | A | C | C | A | G | G | A | T | A | A | T | A | T | T | T | C | C | A | C | A | A | T | T | T | T | T | A | A | G | G | G | G | G | A | G | G | A | G | A | T | T | G | G | G | G | A | A | A | C | A | C | T | T | T |  | : |  | 4 | 1 | 0 | 4 |
| M | T | 0 | 3 | 6 | 0 | 5 | 3 |  | : |  | G | C | A | C | C | A | A | A | A | A | G | G | T | A | A | C | A | A | A | A | G | C | T | T | A | T | T | T | T | C | A | T | G | G | A | T | T | T | C | C | A | G | G | A | T | T | C | C | T | T | G | A | A | A | G | T | A | A | G | A | A | A | A | T | A | G | G | A | A | G | T | A | A | T | A | T | T | C | C | T | A | C | A | C | C | A | A | A | C | C | C | T | G | A | A | A | A | T | A | T | T | A | C | C | C | C | A | G | T | G | A | C | C | A | T | T | C | A | C | C | A | G | G | A | T | A | A | T | A | T | T | T | C | C | A | C | A | A | T | T | T | T | T | A | A | G | G | G | G | G | A | G | G | A | G | A | T | T | G | G | G | G | A | A | A | C | A | C | T | T | T |  | : |  | 4 | 1 | 0 | 4 |
| M | T | 0 | 3 | 6 | 0 | 5 | 4 |  | : |  | G | C | A | C | C | A | A | A | A | A | G | G | T | A | A | C | A | A | A | A | G | C | T | T | A | T | T | T | T | C | A | T | G | G | A | T | T | T | C | C | A | G | G | A | T | T | C | C | T | T | G | A | A | A | G | T | A | A | G | A | A | A | A | T | A | G | G | A | A | G | T | A | A | T | A | T | T | C | C | T | A | C | A | C | C | A | A | A | C | C | C | T | G | A | A | A | A | T | A | T | T | A | C | C | C | C | A | G | T | G | A | C | C | A | T | T | C | A | C | C | A | G | G | A | T | A | A | T | A | T | T | T | C | C | A | C | A | A | T | T | T | T | T | A | A | G | G | G | G | G | A | G | G | A | G | A | T | T | G | G | G | G | A | A | A | C | A | C | T | T | T |  | : |  | 4 | 1 | 0 | 4 |
| M | T | 0 | 3 | 6 | 0 | 5 | 5 |  | : |  | G | C | A | C | C | A | A | A | A | A | G | G | T | A | A | C | A | A | A | A | G | C | T | T | A | T | T | T | T | C | A | T | G | G | A | T | T | T | C | C | A | G | G | A | T | T | C | C | T | T | G | A | A | A | G | T | A | A | G | A | A | A | A | T | A | G | G | A | A | G | T | A | A | T | A | T | T | C | C | T | A | C | A | C | C | A | A | A | C | C | C | T | G | A | A | A | A | T | A | T | T | A | C | C | C | C | A | G | T | G | A | C | C | A | T | T | C | A | C | C | A | G | G | A | T | A | A | T | A | T | T | T | C | C | A | C | A | A | T | T | T | T | T | A | C | G | G | G | G | G | A | G | G | A | G | A | T | T | G | G | G | G | A | A | A | C | A | C | T | T | T |  | : |  | 4 | 1 | 0 | 4 |
| M | T | 0 | 3 | 6 | 0 | 5 | 6 |  | : |  | G | C | A | C | C | A | A | A | A | A | G | G | T | A | A | C | A | A | A | A | G | C | T | T | A | T | T | T | T | C | A | T | G | G | A | C | A | T | C | C | A | G | G | A | T | T | C | C | T | T | G | A | A | A | G | T | A | A | G | A | A | A | A | T | A | G | G | A | A | G | T | A | A | C | A | T | T | C | C | T | A | C | A | C | C | A | A | A | C | C | C | T | G | A | A | A | A | T | A | T | T | A | C | C | C | C | A | G | T | G | A | C | C | A | T | T | C | A | C | C | A | G | G | A | T | A | A | T | A | T | T | T | C | C | A | C | A | A | T | T | T | T | T | A | A | G | G | G | G | G | A | G | G | A | G | A | T | T | G | G | G | G | A | A | A | C | A | C | T | T | T |  | : |  | 4 | 1 | 0 | 1 |
| M | T | 0 | 3 | 6 | 0 | 5 | 7 |  | : |  | G | C | A | C | C | A | A | A | A | A | G | G | T | A | A | C | A | A | A | A | G | C | T | T | A | T | T | T | T | C | A | T | G | G | A | C | A | T | C | C | A | G | G | A | T | T | C | C | T | T | G | A | A | A | G | T | A | A | G | A | A | A | A | T | A | G | G | A | A | G | T | A | A | C | A | T | T | C | C | T | A | C | A | C | C | A | A | A | C | C | C | T | G | A | A | A | A | T | A | T | T | A | C | C | C | C | A | G | T | G | A | C | C | A | T | T | C | A | C | C | A | G | G | A | T | A | A | T | A | T | T | T | C | C | A | C | A | A | T | T | T | T | T | A | A | G | G | G | G | G | A | G | G | A | G | A | T | T | G | G | G | G | A | A | A | C | A | C | T | T | T |  | : |  | 4 | 1 | 0 | 1 |
| K | X | 2 | 4 | 9 | 7 | 3 | 8 |  | : |  | G | C | A | C | C | A | A | A | A | A | G | G | T | A | A | C | A | A | A | A | G | C | T | T | A | T | T | T | T | C | A | T | G | G | A | C | A | T | C | C | A | G | G | A | T | T | C | C | T | T | G | A | A | A | G | T | A | A | G | A | A | A | A | T | A | G | G | A | A | G | T | A | A | C | A | T | T | C | C | T | A | C | A | C | C | A | A | A | C | C | C | T | G | A | A | A | A | T | A | T | T | A | C | T | C | C | A | G | T | G | A | C | C | A | T | T | C | A | C | C | A | G | G | A | T | A | A | T | A | T | T | T | C | C | A | C | A | A | T | T | T | T | T | A | A | G | G | G | G | G | A | G | G | A | G | A | T | T | G | G | G | G | A | A | A | C | A | C | T | T | T |  | : |  | 4 | 1 | 0 | 1 |
| K | X | 2 | 4 | 9 | 7 | 3 | 7 |  | : |  | G | C | A | C | C | A | A | A | A | A | G | G | T | A | A | C | A | A | A | A | G | C | T | T | A | T | T | T | T | C | A | T | G | G | A | C | A | T | C | C | A | G | G | A | T | T | C | C | T | T | G | A | A | A | G | T | A | A | G | A | A | A | A | T | A | G | G | A | A | G | T | A | A | C | A | T | T | C | C | T | A | C | A | C | C | A | A | A | C | C | C | T | G | A | A | A | A | T | A | T | T | A | C | T | C | C | A | G | T | G | A | C | C | A | T | T | C | A | C | C | A | G | G | A | T | A | A | T | A | T | T | T | C | C | A | C | A | A | T | T | T | T | T | A | A | G | G | G | G | G | A | G | G | A | G | A | T | T | G | G | G | G | A | A | A | C | A | C | T | T | T |  | : |  | 4 | 1 | 0 | 1 |
| K | X | 2 | 4 | 9 | 7 | 3 | 6 |  | : |  | G | C | A | C | C | A | A | A | A | A | G | G | T | A | A | C | A | A | A | A | G | C | T | T | A | T | T | T | T | C | A | T | G | G | A | C | A | T | C | C | A | G | G | A | T | T | C | C | T | T | G | A | A | A | G | T | A | A | G | A | A | A | A | T | A | G | G | A | A | G | T | A | A | C | A | T | T | C | C | T | A | C | A | C | C | A | A | A | C | C | C | T | G | A | A | A | A | T | A | T | T | A | C | T | C | C | A | G | T | G | A | C | C | A | T | T | C | A | C | C | A | G | G | A | T | A | A | T | A | T | T | T | C | C | A | C | A | A | T | T | T | T | T | A | A | G | G | G | G | G | A | G | G | A | G | A | T | T | G | G | G | G | A | A | A | C | A | C | T | T | T |  | : |  | 4 | 1 | 0 | 1 |
| K | X | 2 | 4 | 9 | 7 | 3 | 5 |  | : |  | G | C | A | C | C | A | A | A | A | A | G | G | T | A | A | C | A | A | A | A | G | C | T | T | A | T | T | T | T | C | A | T | G | G | A | C | A | T | C | C | A | G | G | A | T | T | C | C | T | T | G | A | A | A | G | T | A | A | G | A | A | A | A | T | A | G | G | A | A | G | T | A | A | C | A | T | T | C | C | T | A | C | A | C | C | A | A | A | C | C | C | T | G | A | A | A | A | T | A | T | T | A | C | T | C | C | A | G | T | G | A | C | C | A | T | T | C | A | C | C | A | G | G | A | T | A | A | T | A | T | T | T | C | C | A | C | A | A | T | T | T | T | T | A | A | G | G | G | G | G | A | G | G | A | G | A | T | T | G | G | G | G | A | A | A | C | A | C | T | T | T |  | : |  | 4 | 1 | 0 | 1 |
| M | F | 1 | 9 | 7 | 9 | 1 | 6 |  | : |  | G | C | A | C | C | A | A | A | A | A | G | G | T | A | A | C | A | A | A | A | G | C | T | T | A | T | T | T | T | C | A | T | G | G | A | C | T | T | C | C | A | G | G | A | T | T | C | C | T | T | G | A | A | A | G | T | A | A | G | A | A | A | A | T | C | G | G | A | A | G | T | A | A | C | A | T | T | C | C | T | A | C | A | C | C | A | A | A | C | C | C | T | G | A | A | A | A | T | A | T | T | A | C | C | C | C | A | G | T | G | A | C | C | A | T | T | C | A | C | C | A | G | G | A | T | A | A | T | A | T | T | T | C | C | A | C | C | A | T | T | T | T | T | A | A | G | G | G | G | G | A | G | G | A | G | A | T | T | G | G | G | G | A | A | A | C | A | C | T | T | T |  | : |  | 4 | 1 | 0 | 4 |
| K | T | 2 | 5 | 0 | 6 | 3 | 2 |  | : |  | G | C | A | C | C | A | A | A | A | A | G | G | T | A | A | C | A | A | A | A | G | C | T | T | A | T | T | T | T | C | A | T | G | G | A | T | T | T | C | C | G | G | G | A | T | T | C | C | T | T | G | A | A | A | G | T | A | A | G | A | G | A | A | T | A | G | G | A | A | G | T | A | A | T | A | T | T | C | C | T | A | C | A | C | C | A | A | A | C | C | C | T | G | A | A | A | A | T | A | T | T | A | C | C | C | C | A | G | T | G | A | C | C | A | T | T | C | A | C | C | A | G | G | A | T | A | A | T | A | T | T | T | C | C | A | C | A | A | T | T | T | T | T | A | A | G | G | G | G | G | A | G | G | A | G | A | T | T | G | G | G | G | A | A | A | C | A | C | T | T | T |  | : |  | 4 | 1 | 0 | 4 |
|  |  |  |  |  |  |  |  |  |  |  | G | C | A | C | C | A | A | A | A | A | G | G | T | A | A | C | A | A | A | A | G | C | T | T | A | T | T | T | T | C | A | T | G | G | A |  |  | T | C | C | a | G | G | A | T | T | C | C | T | T | G | A | A | A | G | T | A | A | G | A | a | A | A | T | a | G | G | A | A | G | T | A | A |  | A | T | T | C | C | T | A | C | A | C | C | A | A | A | C | C | C | T | G | A | A | A | A | T | A | T | T | a | C |  | C | C | A | G | T | G | A | C | C | A | T | T | C | A | C | C | A | G | G | A | T | A | A | T | A | T | T | T | C | C | A | C | a | A | T | T | T | T | T | A | a | G | G | G | G | G | A | G | G | A | G | A | T | T | G | G | G | G | A | A | A | C | A | C | T | T | T |  |  |  |  |  |  |  |

|  |  |  |  |  |  |  |  |  |  |  |  |  |  |  |  |  |  |  |  |  |  |  |  |  |  |  |  |  |  |  |  |  |  |  |  |  |  |  |  |  |  |  |  |  |  |  |  |  |  |  |  |  |  |  |  |  |  |  |  |  |  |  |  |  |  |  |  |  |  |  |  |  |  |  |  |  |  |  |  |  |  |  |  |  |  |  |  |  |  |  |  |  |  |  |  |  |  |  |  |  |  |  |  |  |  |  |  |  |  |  |  |  |  |  |  |  |  |  |  |  |  |  |  |  |  |  |  |  |  |  |  |  |  |  |  |  |  |  |  |  |  |  |  |  |  |  |  |  |  |  |  |  |  |  |  |  |  |  |  |  |  |  |  |  |  |  |  |  |  |  |  |  |  |  |  |  |  |  |  |  |  |  |  |  |  |  |  |  |
| --- | --- | --- | --- | --- | --- | --- | --- | --- | --- | --- | --- | --- | --- | --- | --- | --- | --- | --- | --- | --- | --- | --- | --- | --- | --- | --- | --- | --- | --- | --- | --- | --- | --- | --- | --- | --- | --- | --- | --- | --- | --- | --- | --- | --- | --- | --- | --- | --- | --- | --- | --- | --- | --- | --- | --- | --- | --- | --- | --- | --- | --- | --- | --- | --- | --- | --- | --- | --- | --- | --- | --- | --- | --- | --- | --- | --- | --- | --- | --- | --- | --- | --- | --- | --- | --- | --- | --- | --- | --- | --- | --- | --- | --- | --- | --- | --- | --- | --- | --- | --- | --- | --- | --- | --- | --- | --- | --- | --- | --- | --- | --- | --- | --- | --- | --- | --- | --- | --- | --- | --- | --- | --- | --- | --- | --- | --- | --- | --- | --- | --- | --- | --- | --- | --- | --- | --- | --- | --- | --- | --- | --- | --- | --- | --- | --- | --- | --- | --- | --- | --- | --- | --- | --- | --- | --- | --- | --- | --- | --- | --- | --- | --- | --- | --- | --- | --- | --- | --- | --- | --- | --- | --- | --- | --- | --- | --- | --- | --- | --- | --- | --- | --- | --- | --- | --- | --- | --- | --- |
|  |  |  |  |  |  |  |  |  |  |  |  |  |  |  |  |  |  |  |  |  |  |  |  |  |  |  |  |  |  |  |  |  |  |  |  |  |  |  |  |  |  |  |  |  |  |  |  |  |  |  |  |  |  |  |  |  |  |  |  |  |  |  |  |  |  |  |  |  |  |  |  |  |  |  |  |  |  |  |  |  |  |  |  |  |  |  |  |  |  |  |  |  |  |  |  |  |  |  |  |  |  |  |  |  |  |  |  |  |  |  |  |  |  |  |  |  |  |  |  |  |  |  |  |  |  |  |  |  |  |  |  |  |  |  |  |  |  |  |  |  |  |  |  |  |  |  |  |  |  |  |  |  |  |  |  |  |  |  |  |  |  |  |  |  |  |  |  |  |  |  |  |  |  |  |  |  |  |  |  |  |  |  |  |  |  |  |  |  |
|  |  |  |  |  |  |  |  |  |  |  |  |  |  |  |  | \* |  |  |  |  |  |  | 4 | 1 | 2 | 0 |  |  |  |  |  |  |  |  |  | \* |  |  |  |  |  |  | 4 | 1 | 4 | 0 |  |  |  |  |  |  |  |  |  | \* |  |  |  |  |  |  | 4 | 1 | 6 | 0 |  |  |  |  |  |  |  |  |  | \* |  |  |  |  |  |  | 4 | 1 | 8 | 0 |  |  |  |  |  |  |  |  |  | \* |  |  |  |  |  |  | 4 | 2 | 0 | 0 |  |  |  |  |  |  |  |  |  | \* |  |  |  |  |  |  | 4 | 2 | 2 | 0 |  |  |  |  |  |  |  |  |  | \* |  |  |  |  |  |  | 4 | 2 | 4 | 0 |  |  |  |  |  |  |  |  |  | \* |  |  |  |  |  |  | 4 | 2 | 6 | 0 |  |  |  |  |  |  |  |  |  | \* |  |  |  |  |  |  |  |  |  |  |  |  |
| M | N | 9 | 5 | 6 | 5 | 2 | 0 |  | : |  | T | C | T | C | A | G | T | C | A | T | T | T | C | A | G | C | C | T | T | C | A | C | A | G | A | A | G | T | T | G | A | A | A | A | A | T | T | A | C | T | T | G | A | T | T | C | T | A | T | T | T | G | T | T | C | A | G | A | A | C | A | C | C | C | A | C | T | T | G | A | C | G | A | A | A | G | A | A | T | C | A | A | C | A | A | A | G | G | T | A | A | A | T | T | T | G | A | A | G | C | A | C | A | A | A | T | T | A | C | C | T | T | A | T | T | A | G | A | T | C | C | T | A | A | C | A | A | A | G | T | G | A | T | T | A | A | G | T | G | C | A | A | A | C | C | T | A | T | G | C | A | G | T | A | T | T | C | A | C | C | A | C | A | G | G | A | C | A | G | G | G |  | : |  | 4 | 2 | 7 | 2 |
| M | T | 0 | 1 | 2 | 7 | 3 | 2 |  | : |  | T | C | T | C | A | G | T | C | A | T | T | T | C | A | G | C | T | T | T | C | A | C | A | G | A | A | G | T | T | G | A | A | A | A | A | T | T | A | C | T | T | G | A | T | T | C | T | A | T | T | T | G | T | T | C | A | G | A | A | C | A | C | C | C | A | C | T | T | G | A | C | G | A | A | A | G | A | A | T | C | A | A | C | A | A | A | G | G | T | A | A | A | T | T | T | G | A | A | G | C | A | C | A | A | A | T | T | A | C | C | T | T | A | T | T | A | G | A | T | C | C | T | A | A | C | A | A | A | G | T | G | A | T | T | A | A | G | T | G | C | A | A | A | C | C | T | A | T | G | C | A | G | T | A | T | T | C | A | C | C | A | C | A | G | G | A | C | A | G | G | G |  | : |  | 4 | 2 | 7 | 2 |
| M | T | 0 | 1 | 2 | 7 | 3 | 4 |  | : |  | T | C | T | C | A | G | T | C | A | T | T | T | C | A | G | C | T | T | T | C | A | C | A | G | A | A | G | T | T | G | A | A | A | A | A | T | T | A | C | T | T | G | A | T | T | C | T | A | T | T | T | G | T | T | C | A | G | A | A | C | A | C | C | C | A | C | T | T | G | A | C | G | A | A | A | G | A | A | T | C | A | A | C | A | A | A | G | G | T | A | A | A | T | T | T | G | A | A | G | C | A | C | A | A | A | T | T | A | C | C | T | T | A | T | T | A | G | A | T | C | C | T | A | A | C | A | A | A | G | T | G | A | T | T | A | A | G | T | G | C | A | A | A | C | C | T | A | T | G | C | A | G | T | A | T | T | C | A | C | C | A | C | A | G | G | A | C | A | G | G | G |  | : |  | 4 | 2 | 7 | 2 |
| M | T | 0 | 2 | 7 | 0 | 0 | 6 |  | : |  | T | C | T | C | A | G | T | C | A | T | T | T | C | A | G | C | T | T | T | C | A | C | A | G | A | A | G | T | T | G | A | A | A | A | A | T | T | A | C | T | T | G | A | T | T | C | T | A | T | T | T | G | T | T | C | A | G | A | A | C | A | C | C | C | A | C | T | T | G | A | C | G | A | A | A | G | A | A | T | C | A | A | C | A | A | A | G | G | T | A | A | A | T | T | T | G | A | A | G | C | A | C | A | A | A | T | T | A | C | C | T | T | A | T | T | A | G | A | T | C | C | T | A | A | C | A | A | A | G | T | G | A | T | T | A | A | G | T | G | C | A | A | A | C | C | T | A | T | G | C | A | G | T | A | T | T | C | A | C | C | A | C | A | G | G | A | C | A | G | G | G |  | : |  | 4 | 2 | 7 | 2 |
| M | T | 0 | 2 | 7 | 0 | 0 | 7 |  | : |  | T | C | T | C | A | G | T | C | A | T | T | T | C | A | G | C | T | T | T | C | A | C | A | G | A | A | G | T | T | G | A | A | A | A | A | T | T | A | C | T | T | G | A | T | T | C | T | A | T | T | T | G | T | T | C | A | G | A | A | C | A | C | C | C | A | C | T | T | G | A | C | A | A | A | A | G | A | A | T | C | A | A | C | A | A | A | G | G | A | A | A | A | T | T | T | G | A | A | G | C | A | C | A | A | A | T | T | A | C | C | T | T | A | T | T | A | G | A | T | C | C | T | A | A | C | A | A | A | G | T | G | A | T | T | A | A | G | T | G | C | A | A | A | C | C | T | A | T | G | C | A | G | T | A | T | T | C | A | C | C | A | C | A | G | G | A | C | A | G | G | G |  | : |  | 4 | 2 | 7 | 5 |
| M | T | 0 | 3 | 6 | 0 | 5 | 3 |  | : |  | T | C | T | C | A | G | T | C | A | T | T | T | C | A | G | C | T | T | T | C | A | C | A | G | A | A | G | T | T | G | A | A | A | A | A | T | T | A | C | T | T | G | A | T | T | C | T | A | T | T | T | G | T | T | C | A | G | A | A | C | A | C | C | C | A | C | T | T | G | A | C | G | A | A | A | G | A | A | T | C | A | A | C | A | A | A | G | G | T | A | A | A | T | T | T | G | A | A | G | C | A | C | A | A | A | T | T | A | C | C | T | T | A | T | T | A | G | A | T | C | C | T | A | A | C | A | A | A | G | T | G | A | T | T | A | A | G | T | G | C | A | A | A | C | C | T | A | T | G | C | A | G | T | A | T | T | C | A | C | C | A | C | A | G | G | A | C | A | G | G | G |  | : |  | 4 | 2 | 7 | 5 |
| M | T | 0 | 3 | 6 | 0 | 5 | 4 |  | : |  | T | C | T | C | A | G | T | C | A | T | T | T | C | A | G | C | T | T | T | C | A | C | A | G | A | A | G | T | T | G | A | A | A | A | A | T | T | A | C | T | T | G | A | T | T | C | T | A | T | T | T | G | T | T | C | A | G | A | A | C | A | C | C | C | A | C | T | T | G | A | C | G | A | A | A | G | A | A | T | C | A | A | C | A | A | A | G | G | T | A | A | A | T | T | T | G | A | A | G | C | A | C | A | A | A | T | T | A | C | C | T | T | A | T | T | A | G | A | T | C | C | T | A | A | C | A | A | A | G | T | G | A | T | T | A | A | G | T | G | C | A | A | A | C | C | T | A | T | G | C | A | G | T | A | T | T | C | A | C | C | A | C | A | G | G | A | C | A | G | G | G |  | : |  | 4 | 2 | 7 | 5 |
| M | T | 0 | 3 | 6 | 0 | 5 | 5 |  | : |  | T | C | T | C | A | G | T | C | A | T | T | T | C | A | G | C | T | T | T | C | A | C | A | G | A | A | G | T | T | G | A | A | A | A | A | T | T | A | C | T | T | G | A | T | T | C | T | A | T | T | T | G | T | T | C | A | G | A | A | C | A | C | C | C | A | C | T | T | G | A | C | G | A | A | A | G | A | A | T | C | A | A | C | A | A | A | G | G | T | A | A | A | T | T | T | G | A | A | G | C | A | C | A | A | A | T | T | A | C | C | T | T | A | T | T | A | G | A | T | C | C | T | A | A | C | A | A | A | G | T | G | A | T | T | A | A | G | T | G | C | A | A | A | C | C | T | A | T | G | C | A | A | T | A | T | T | C | A | C | C | A | C | A | G | G | A | C | A | G | G | G |  | : |  | 4 | 2 | 7 | 5 |
| M | T | 0 | 3 | 6 | 0 | 5 | 6 |  | : |  | T | C | T | C | A | G | T | C | A | T | T | T | C | A | G | C | T | T | T | C | A | C | A | G | A | A | G | T | T | G | A | A | A | A | A | T | T | A | C | T | T | G | A | T | T | C | T | A | T | T | T | G | T | T | C | A | G | A | A | C | A | C | C | C | A | C | T | T | G | A | C | G | A | A | A | G | A | A | T | C | A | A | C | A | A | A | G | G | T | A | A | A | T | T | T | G | A | A | G | C | A | C | A | A | A | T | T | A | C | C | T | T | A | T | T | A | G | A | T | C | C | T | A | A | C | A | A | A | G | T | G | A | T | T | A | A | G | T | G | C | A | A | A | C | C | T | A | T | G | C | A | G | T | A | T | T | C | A | C | C | A | C | A | G | G | A | C | A | G | G | G |  | : |  | 4 | 2 | 7 | 2 |
| M | T | 0 | 3 | 6 | 0 | 5 | 7 |  | : |  | T | C | T | C | A | G | T | C | A | T | T | T | C | A | G | C | T | T | T | C | A | C | A | G | A | A | G | T | T | G | A | A | A | A | A | T | T | A | C | T | T | G | A | T | T | C | T | A | T | T | T | G | T | T | C | A | G | A | A | C | A | C | C | C | A | C | T | T | G | A | C | G | A | A | A | G | A | A | T | C | A | A | C | A | A | A | G | G | T | A | A | A | T | T | T | G | A | A | G | C | A | C | A | A | A | T | T | A | C | C | T | T | A | T | T | A | G | A | T | C | C | T | A | A | C | A | A | A | G | T | G | A | T | T | A | A | G | T | G | C | A | A | A | C | C | T | A | T | G | C | A | G | T | A | T | T | C | A | C | C | A | C | A | G | G | A | C | A | G | G | G |  | : |  | 4 | 2 | 7 | 2 |
| K | X | 2 | 4 | 9 | 7 | 3 | 8 |  | : |  | T | C | T | C | A | G | T | C | A | T | T | T | C | A | G | C | T | T | T | C | A | C | A | G | A | A | G | T | T | G | A | A | A | A | A | T | T | A | C | T | T | G | A | T | T | C | T | A | T | T | T | G | T | T | C | A | G | A | A | C | A | C | C | C | A | C | T | T | G | A | T | G | A | A | A | G | A | A | T | C | A | A | C | A | A | A | G | G | A | A | A | A | T | T | T | G | A | A | G | C | A | C | A | A | A | T | T | A | C | C | T | T | A | T | T | A | G | A | T | C | C | T | A | A | C | A | A | A | G | T | G | A | T | T | A | A | G | T | G | C | A | A | A | C | C | T | A | T | G | C | A | G | T | A | T | T | C | A | C | C | A | C | A | G | G | A | C | A | G | G | G |  | : |  | 4 | 2 | 7 | 2 |
| K | X | 2 | 4 | 9 | 7 | 3 | 7 |  | : |  | T | C | T | C | A | G | T | C | A | T | T | T | C | A | G | C | T | T | T | C | A | C | A | G | A | A | G | T | T | G | A | A | A | A | A | T | T | A | C | T | T | G | A | T | T | C | T | A | T | T | T | G | T | T | C | A | G | A | A | C | A | C | C | C | A | C | T | T | G | A | T | G | A | A | A | G | A | A | T | C | A | A | C | A | A | A | G | G | A | A | A | A | T | T | T | G | A | A | G | C | A | C | A | A | A | T | T | A | C | C | T | T | A | T | T | A | G | A | T | C | C | T | A | A | C | A | A | A | G | T | G | A | T | T | A | A | G | T | G | C | A | A | A | C | C | T | A | T | G | C | A | G | T | A | T | T | C | A | C | C | A | C | A | G | G | A | C | A | G | G | G |  | : |  | 4 | 2 | 7 | 2 |
| K | X | 2 | 4 | 9 | 7 | 3 | 6 |  | : |  | T | C | T | C | A | G | T | C | A | T | T | T | C | A | G | C | T | T | T | C | A | C | A | G | A | A | G | T | T | G | A | A | A | A | A | T | T | A | C | T | T | G | A | T | T | C | T | A | T | T | T | G | T | T | C | A | G | A | A | C | A | C | C | C | A | C | T | T | G | A | T | G | A | A | A | G | A | A | T | C | A | A | C | A | A | A | G | G | A | A | A | A | T | T | T | G | A | A | G | C | A | C | A | A | A | T | T | A | C | C | T | T | A | T | T | A | G | A | T | C | C | T | A | A | C | A | A | A | G | T | G | A | T | T | A | A | G | T | G | C | A | A | A | C | C | T | A | T | G | C | A | G | T | A | T | T | C | A | C | C | A | C | A | G | G | A | C | A | G | G | G |  | : |  | 4 | 2 | 7 | 2 |
| K | X | 2 | 4 | 9 | 7 | 3 | 5 |  | : |  | T | C | T | C | A | G | T | C | A | T | T | T | C | A | G | C | T | T | T | C | A | C | A | G | A | A | G | T | T | G | A | A | A | A | A | T | T | A | C | T | T | G | A | T | T | C | T | A | T | T | T | G | T | T | C | A | G | A | A | C | A | C | C | C | A | C | T | T | G | A | C | G | A | A | A | G | A | A | T | C | A | A | C | A | A | A | G | G | T | A | A | A | T | T | T | G | A | A | G | C | A | C | A | A | A | T | T | A | C | C | T | T | A | T | T | A | G | A | T | C | C | T | A | A | C | A | A | A | G | T | G | A | T | T | A | A | G | T | G | C | A | A | A | C | C | T | A | T | G | C | A | G | T | A | T | T | C | A | C | C | A | C | A | G | G | A | C | A | G | G | G |  | : |  | 4 | 2 | 7 | 2 |
| M | F | 1 | 9 | 7 | 9 | 1 | 6 |  | : |  | T | C | T | C | A | G | T | C | A | T | T | T | C | A | G | C | T | T | T | C | A | C | A | G | A | A | G | T | T | G | A | A | A | A | A | T | T | A | C | T | T | G | A | T | T | C | T | A | T | T | T | G | T | T | C | A | G | A | A | C | A | T | C | C | A | C | T | T | G | A | C | G | A | A | A | G | A | A | T | C | A | A | C | A | A | A | G | G | A | A | A | A | T | T | T | G | A | A | G | C | A | C | A | A | A | T | T | A | C | C | T | T | A | T | T | A | G | A | T | C | C | T | A | A | C | A | A | A | G | T | G | A | T | T | A | A | G | T | G | C | A | A | A | C | C | C | A | T | G | C | A | G | T | A | T | T | C | A | C | C | A | C | A | G | G | A | C | A | G | G | G |  | : |  | 4 | 2 | 7 | 5 |
| K | T | 2 | 5 | 0 | 6 | 3 | 2 |  | : |  | T | C | T | C | A | G | T | C | A | T | T | T | C | A | G | C | T | T | T | C | A | C | A | G | A | A | G | T | T | G | A | A | A | A | A | T | T | A | C | T | T | G | A | T | T | C | T | A | T | T | T | G | T | T | C | A | G | A | A | C | A | C | C | C | A | C | T | T | G | A | C | G | A | A | A | G | A | A | T | C | A | A | C | A | A | A | G | G | T | A | A | A | T | T | T | G | A | A | G | C | A | C | A | A | A | T | T | A | C | C | T | T | A | T | T | A | G | A | T | C | C | T | A | A | C | A | A | A | G | T | G | A | T | T | A | A | G | T | G | C | A | A | A | C | C | T | A | T | G | C | A | G | T | A | T | T | C | A | C | C | A | C | A | G | G | A | C | A | G | G | G |  | : |  | 4 | 2 | 7 | 5 |
|  |  |  |  |  |  |  |  |  |  |  | T | C | T | C | A | G | T | C | A | T | T | T | C | A | G | C | t | T | T | C | A | C | A | G | A | A | G | T | T | G | A | A | A | A | A | T | T | A | C | T | T | G | A | T | T | C | T | A | T | T | T | G | T | T | C | A | G | A | A | C | A | c | C | C | A | C | T | T | G | A | c | g | A | A | A | G | A | A | T | C | A | A | C | A | A | A | G | G |  | A | A | A | T | T | T | G | A | A | G | C | A | C | A | A | A | T | T | A | C | C | T | T | A | T | T | A | G | A | T | C | C | T | A | A | C | A | A | A | G | T | G | A | T | T | A | A | G | T | G | C | A | A | A | C | C | t | A | T | G | C | A | g | T | A | T | T | C | A | C | C | A | C | A | G | G | A | C | A | G | G | G |  |  |  |  |  |  |  |

|  |  |  |  |  |  |  |  |  |  |  |  |  |  |  |  |  |  |  |  |  |  |  |  |  |  |  |  |  |  |  |  |  |  |  |  |  |  |  |  |  |  |  |  |  |  |  |  |  |  |  |  |  |  |  |  |  |  |  |  |  |  |  |  |  |  |  |  |  |  |  |  |  |  |  |  |  |  |  |  |  |  |  |  |  |  |  |  |  |  |  |  |  |  |  |  |  |  |  |  |  |  |  |  |  |  |  |  |  |  |  |  |  |  |  |  |  |  |  |  |  |  |  |  |  |  |  |  |  |  |  |  |  |  |  |  |  |  |  |  |  |  |  |  |  |  |  |  |  |  |  |  |  |  |  |  |  |  |  |  |  |  |  |  |  |  |  |  |  |  |  |  |  |  |  |  |  |  |  |  |  |  |  |  |  |  |  |  |  |
| --- | --- | --- | --- | --- | --- | --- | --- | --- | --- | --- | --- | --- | --- | --- | --- | --- | --- | --- | --- | --- | --- | --- | --- | --- | --- | --- | --- | --- | --- | --- | --- | --- | --- | --- | --- | --- | --- | --- | --- | --- | --- | --- | --- | --- | --- | --- | --- | --- | --- | --- | --- | --- | --- | --- | --- | --- | --- | --- | --- | --- | --- | --- | --- | --- | --- | --- | --- | --- | --- | --- | --- | --- | --- | --- | --- | --- | --- | --- | --- | --- | --- | --- | --- | --- | --- | --- | --- | --- | --- | --- | --- | --- | --- | --- | --- | --- | --- | --- | --- | --- | --- | --- | --- | --- | --- | --- | --- | --- | --- | --- | --- | --- | --- | --- | --- | --- | --- | --- | --- | --- | --- | --- | --- | --- | --- | --- | --- | --- | --- | --- | --- | --- | --- | --- | --- | --- | --- | --- | --- | --- | --- | --- | --- | --- | --- | --- | --- | --- | --- | --- | --- | --- | --- | --- | --- | --- | --- | --- | --- | --- | --- | --- | --- | --- | --- | --- | --- | --- | --- | --- | --- | --- | --- | --- | --- | --- | --- | --- | --- | --- | --- | --- | --- | --- | --- | --- | --- | --- |
|  |  |  |  |  |  |  |  |  |  |  |  |  |  |  |  |  |  |  |  |  |  |  |  |  |  |  |  |  |  |  |  |  |  |  |  |  |  |  |  |  |  |  |  |  |  |  |  |  |  |  |  |  |  |  |  |  |  |  |  |  |  |  |  |  |  |  |  |  |  |  |  |  |  |  |  |  |  |  |  |  |  |  |  |  |  |  |  |  |  |  |  |  |  |  |  |  |  |  |  |  |  |  |  |  |  |  |  |  |  |  |  |  |  |  |  |  |  |  |  |  |  |  |  |  |  |  |  |  |  |  |  |  |  |  |  |  |  |  |  |  |  |  |  |  |  |  |  |  |  |  |  |  |  |  |  |  |  |  |  |  |  |  |  |  |  |  |  |  |  |  |  |  |  |  |  |  |  |  |  |  |  |  |  |  |  |  |  |  |
|  |  |  |  |  |  |  |  |  |  |  |  | 4 | 2 | 8 | 0 |  |  |  |  |  |  |  |  |  | \* |  |  |  |  |  |  | 4 | 3 | 0 | 0 |  |  |  |  |  |  |  |  |  | \* |  |  |  |  |  |  | 4 | 3 | 2 | 0 |  |  |  |  |  |  |  |  |  | \* |  |  |  |  |  |  | 4 | 3 | 4 | 0 |  |  |  |  |  |  |  |  |  | \* |  |  |  |  |  |  | 4 | 3 | 6 | 0 |  |  |  |  |  |  |  |  |  | \* |  |  |  |  |  |  | 4 | 3 | 8 | 0 |  |  |  |  |  |  |  |  |  | \* |  |  |  |  |  |  | 4 | 4 | 0 | 0 |  |  |  |  |  |  |  |  |  | \* |  |  |  |  |  |  | 4 | 4 | 2 | 0 |  |  |  |  |  |  |  |  |  | \* |  |  |  |  |  |  | 4 | 4 | 4 | 0 |  |  |  |  |  |  |  |  |  |  |  |  |  |
| M | N | 9 | 5 | 6 | 5 | 2 | 0 |  | : |  | A | A | G | A | G | T | T | T | A | A | A | A | C | T | C | A | G | A | T | C | G | A | A | G | A | G | C | T | A | C | T | C | A | A | G | C | T | A | G | G | G | A | T | C | A | T | C | A | G | A | C | C | C | A | G | C | A | A | A | A | G | T | C | C | T | C | A | C | T | C | A | T | C | A | C | C | A | G | C | C | T | T | C | A | T | G | G | T | T | A | G | G | A | A | T | C | A | T | G | C | C | G | A | G | A | T | C | A | A | A | C | G | C | G | G | C | A | A | A | G | C | A | C | G | T | A | T | G | G | T | C | A | T | T | A | A | C | T | A | T | A | A | A | A | A | G | T | T | A | A | A | T | G | A | C | A | A | T | A | C | C | A | A | G | G | G | A | G | A | C | G |  | : |  | 4 | 4 | 4 | 3 |
| M | T | 0 | 1 | 2 | 7 | 3 | 2 |  | : |  | A | A | G | A | G | T | T | T | A | A | A | A | C | T | C | A | G | A | T | C | G | A | A | G | A | G | C | T | A | C | T | C | A | A | G | C | T | A | G | G | G | A | T | C | A | T | C | A | G | A | C | C | C | A | G | C | A | A | A | A | G | T | C | C | T | C | A | C | T | C | A | T | C | A | C | C | A | G | C | C | T | T | C | A | T | G | G | T | T | A | G | G | A | A | T | C | A | T | G | C | C | G | A | G | A | T | C | A | A | A | C | G | C | G | G | C | A | A | A | G | C | A | C | G | T | A | T | G | G | T | C | A | T | T | A | A | C | T | A | T | A | A | A | A | A | G | T | T | A | A | A | T | G | A | C | A | A | T | A | C | C | A | A | G | G | G | A | G | A | C | G |  | : |  | 4 | 4 | 4 | 3 |
| M | T | 0 | 1 | 2 | 7 | 3 | 4 |  | : |  | A | A | G | A | G | T | T | C | A | A | A | A | C | T | C | A | G | A | T | C | G | A | A | G | A | G | C | T | G | C | T | C | A | A | A | C | T | A | G | G | G | A | T | C | A | T | C | A | G | A | C | C | C | A | G | C | A | A | G | A | G | C | C | C | T | C | A | C | T | C | A | T | C | T | C | C | A | G | C | C | T | T | T | A | T | G | G | T | T | A | G | G | A | A | T | C | A | T | G | C | C | G | A | G | A | T | C | A | A | A | C | G | C | G | G | C | A | A | A | G | C | A | C | G | T | A | T | G | G | T | C | A | T | T | A | A | C | T | A | T | A | A | A | A | A | G | T | C | A | A | A | T | G | A | C | A | A | T | A | C | C | A | A | A | G | G | A | G | A | C | G |  | : |  | 4 | 4 | 4 | 3 |
| M | T | 0 | 2 | 7 | 0 | 0 | 6 |  | : |  | A | A | G | A | G | T | T | T | A | A | A | A | C | T | C | A | G | A | T | C | G | A | A | G | A | G | C | T | A | C | T | C | A | A | G | C | T | A | G | G | G | A | T | C | A | T | C | A | G | A | C | C | C | A | G | C | A | A | A | A | G | T | C | C | T | C | A | C | T | C | A | T | C | A | C | C | A | G | C | C | T | T | C | A | T | G | G | T | T | A | G | G | A | A | T | C | A | T | G | C | C | G | A | G | A | T | C | A | A | A | C | G | C | G | G | C | A | A | A | G | C | A | C | G | T | A | T | G | G | T | C | A | T | T | A | A | C | T | A | T | A | A | A | A | A | G | T | T | A | A | A | T | G | A | C | A | A | T | A | C | C | A | A | G | G | G | A | G | A | C | G |  | : |  | 4 | 4 | 4 | 3 |
| M | T | 0 | 2 | 7 | 0 | 0 | 7 |  | : |  | A | A | G | A | G | T | T | T | A | A | G | A | C | T | C | A | G | A | T | C | G | A | A | G | A | G | C | T | A | C | T | C | A | A | G | C | T | A | G | G | G | A | T | C | A | T | C | A | G | A | C | C | C | A | G | C | A | A | A | A | G | T | C | C | T | C | A | C | T | C | A | T | C | A | C | C | A | G | C | C | T | T | C | A | T | G | G | T | T | A | G | G | A | A | T | C | A | T | G | C | C | G | A | G | A | T | C | A | A | A | C | G | C | G | G | C | A | A | A | G | C | A | C | G | T | A | T | G | G | T | C | A | T | T | A | A | C | T | A | T | A | A | A | A | A | G | T | T | A | A | A | T | G | A | C | A | A | T | A | C | C | A | A | G | G | G | A | G | A | C | G |  | : |  | 4 | 4 | 4 | 6 |
| M | T | 0 | 3 | 6 | 0 | 5 | 3 |  | : |  | A | A | G | A | G | T | T | C | A | A | A | A | C | T | C | A | G | A | T | C | G | A | A | G | A | G | C | T | G | C | T | C | A | A | A | C | T | A | G | G | G | A | T | C | A | T | C | A | G | A | C | C | C | A | G | C | A | A | G | A | G | C | C | C | T | C | A | C | T | C | G | T | C | T | C | C | A | G | C | C | T | T | T | A | T | G | G | T | T | A | G | G | A | A | T | C | A | T | G | C | C | G | A | G | A | T | C | A | A | A | C | G | C | G | G | C | A | A | A | G | C | A | C | G | T | A | T | G | G | T | C | A | T | T | A | A | C | T | A | T | A | A | A | A | A | G | T | T | A | A | A | T | G | A | C | A | A | T | A | C | C | A | A | A | G | G | A | G | A | T | G |  | : |  | 4 | 4 | 4 | 6 |
[truncated: 334,178 more chars]
