# Supplementary material for: Molecular characterization of strawberry vein banding virus from China and the development of loop‑mediated isothermal amplification assays for their detection
Source: Sci Rep. 2022 Mar 22;12:4912. doi: 10.1038/s41598-022-08981-9 (PMC8940885; doi:10.1038/s41598-022-08981-9)

**Supplementary Figure S4a. The effect of temperature in the optimization experiment.**


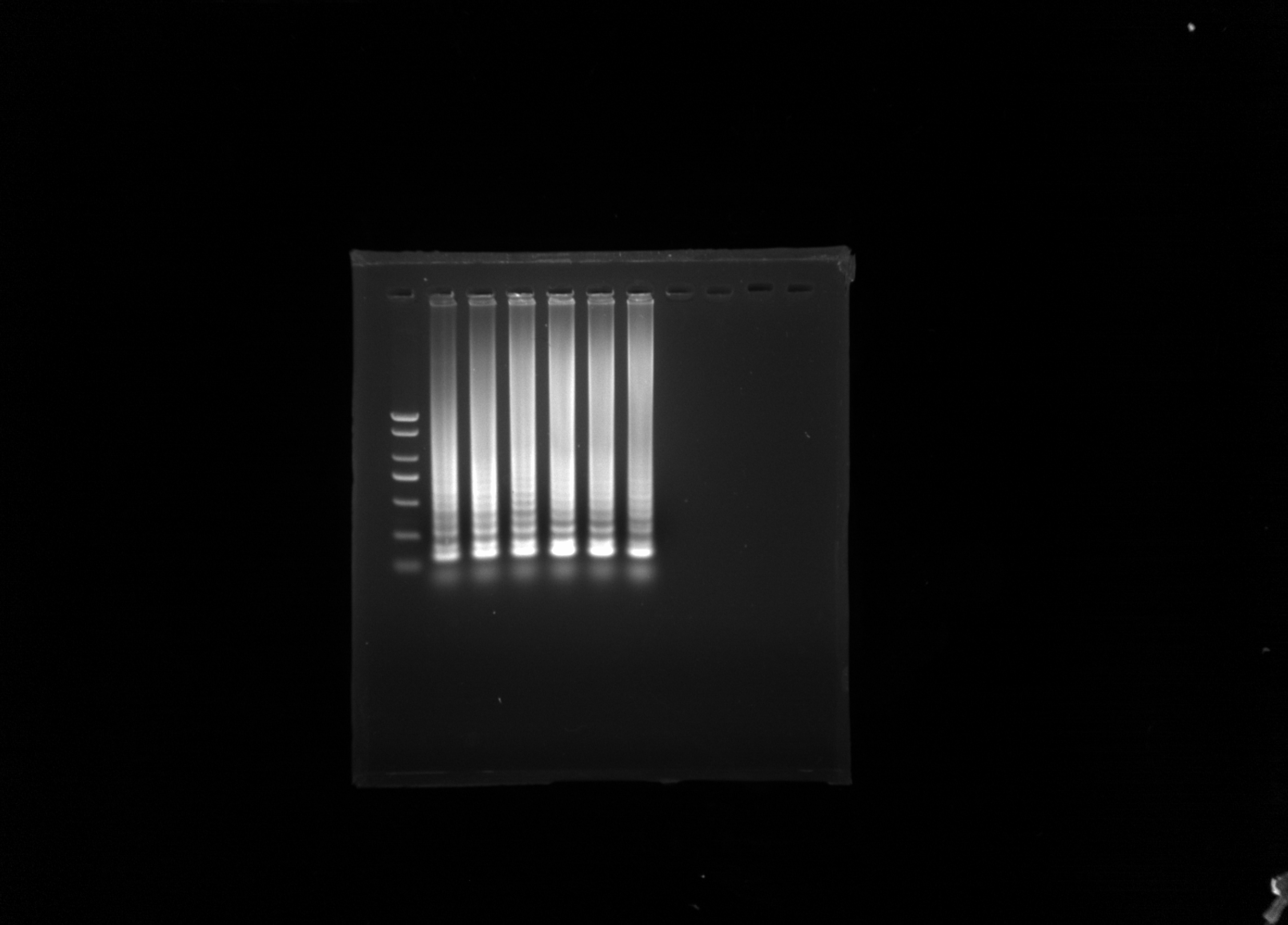


**Supplementary Figure S4b. The effect of time in the optimization experiment.**


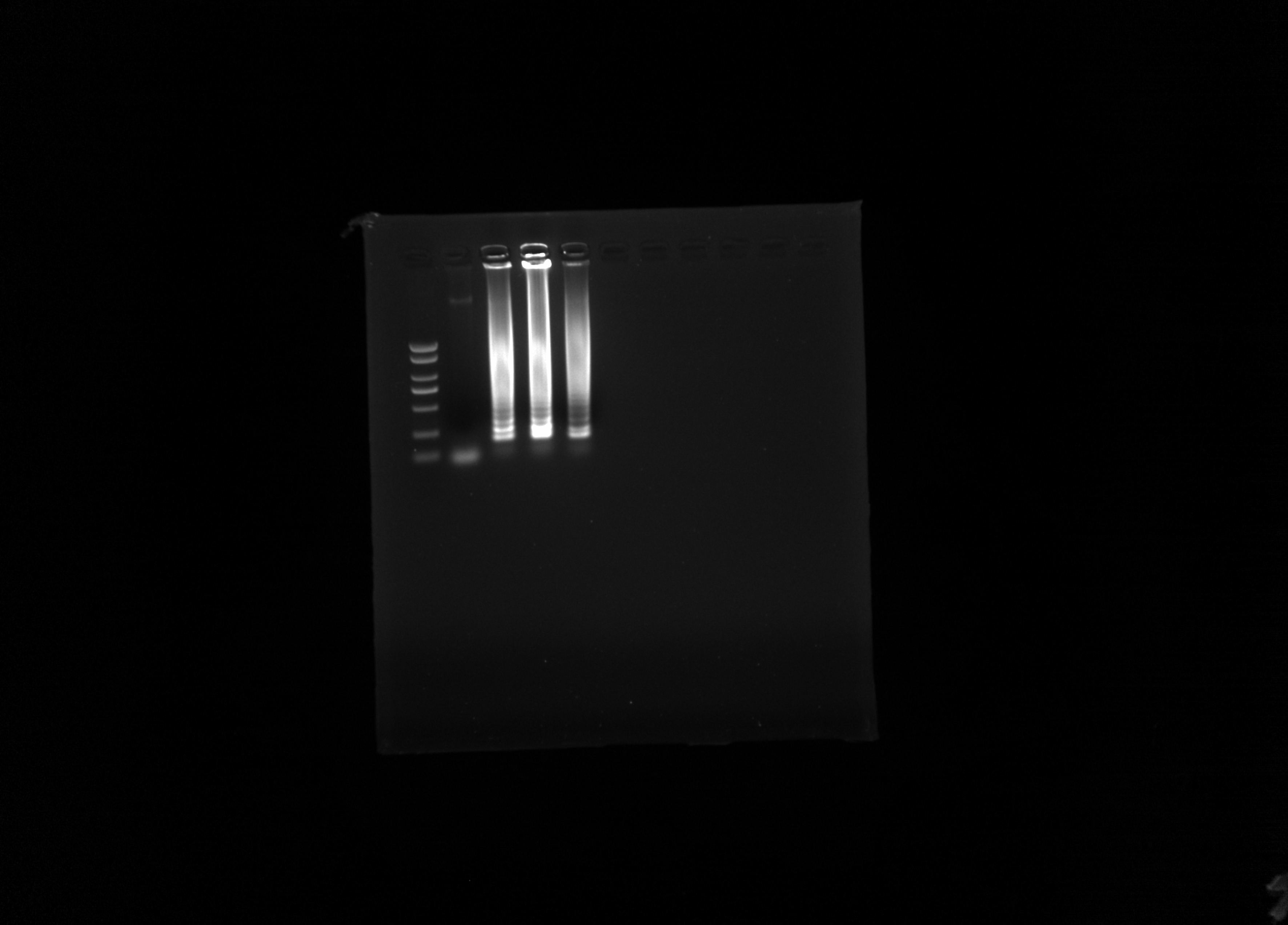


**Supplementary Figure S4c. The effect of primers FIP/BIP in the optimization experiment.**


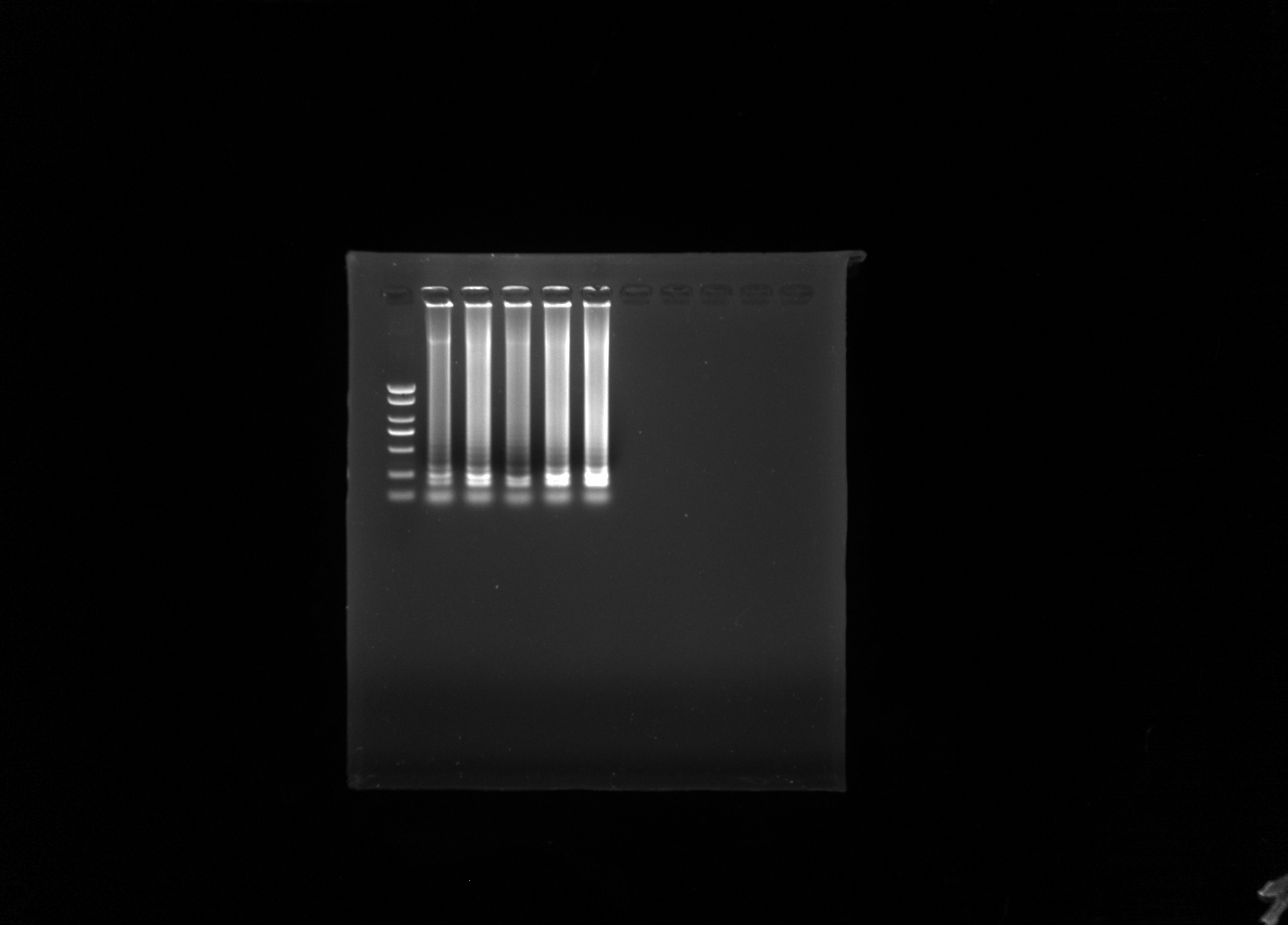


**Supplementary Figure S4d. The effect of primers F3/B3 in the optimization experiment.**


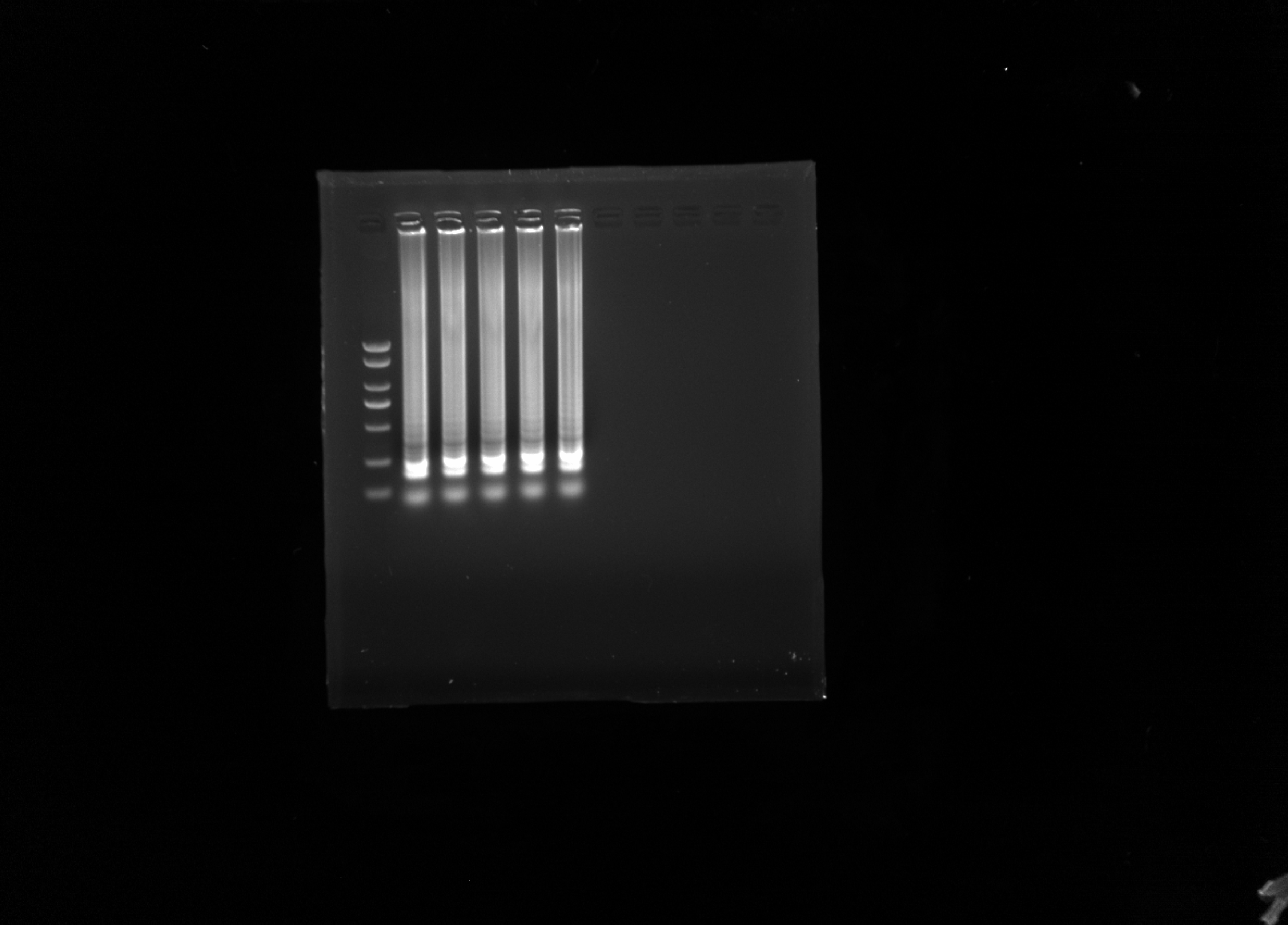


**Supplementary Figure S4e. The effect of Mg^2+^ in the optimization experiment.**


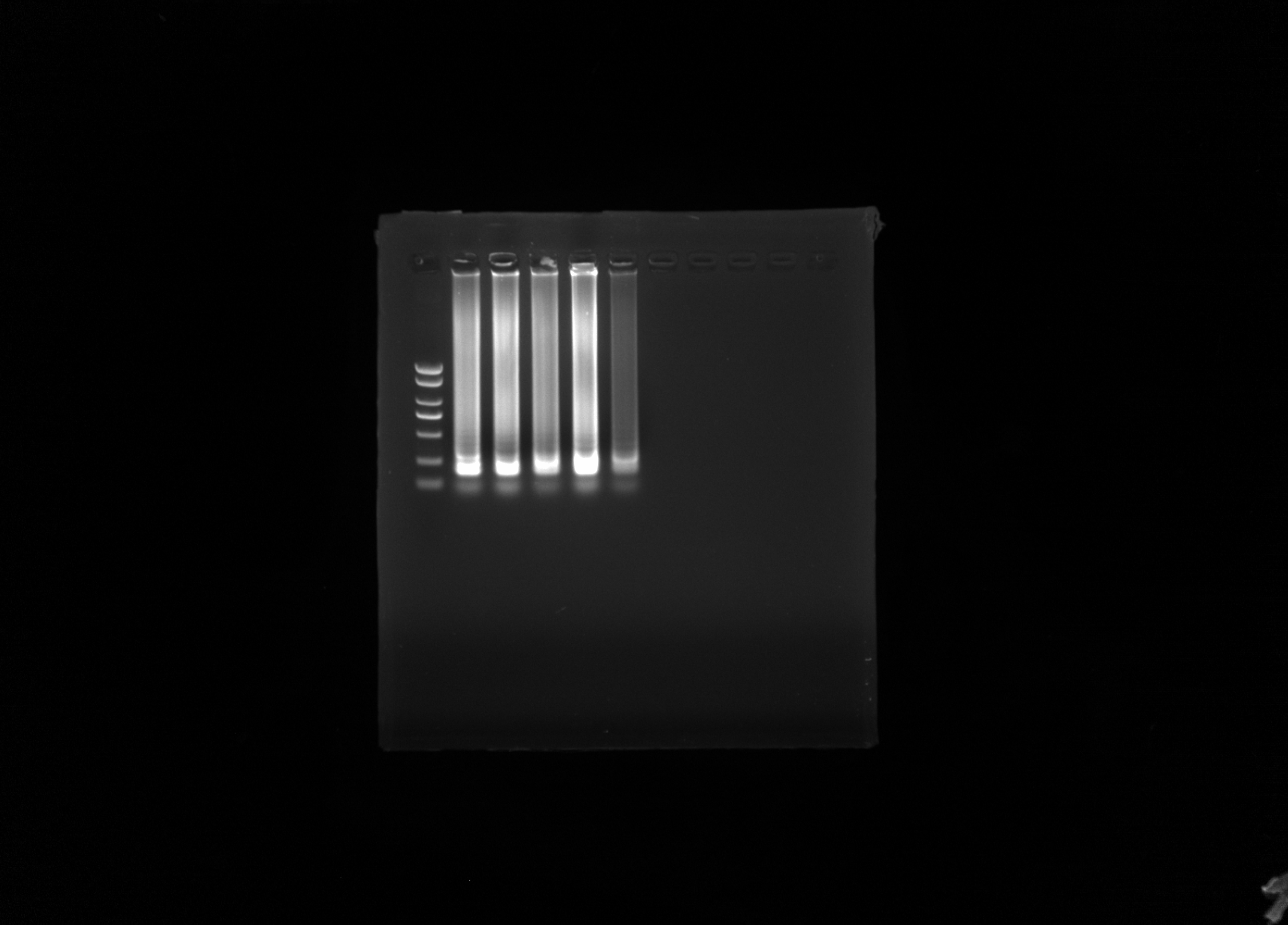


**Supplementary Figure S4f. The effect of dNTPs in the optimization experiment.**


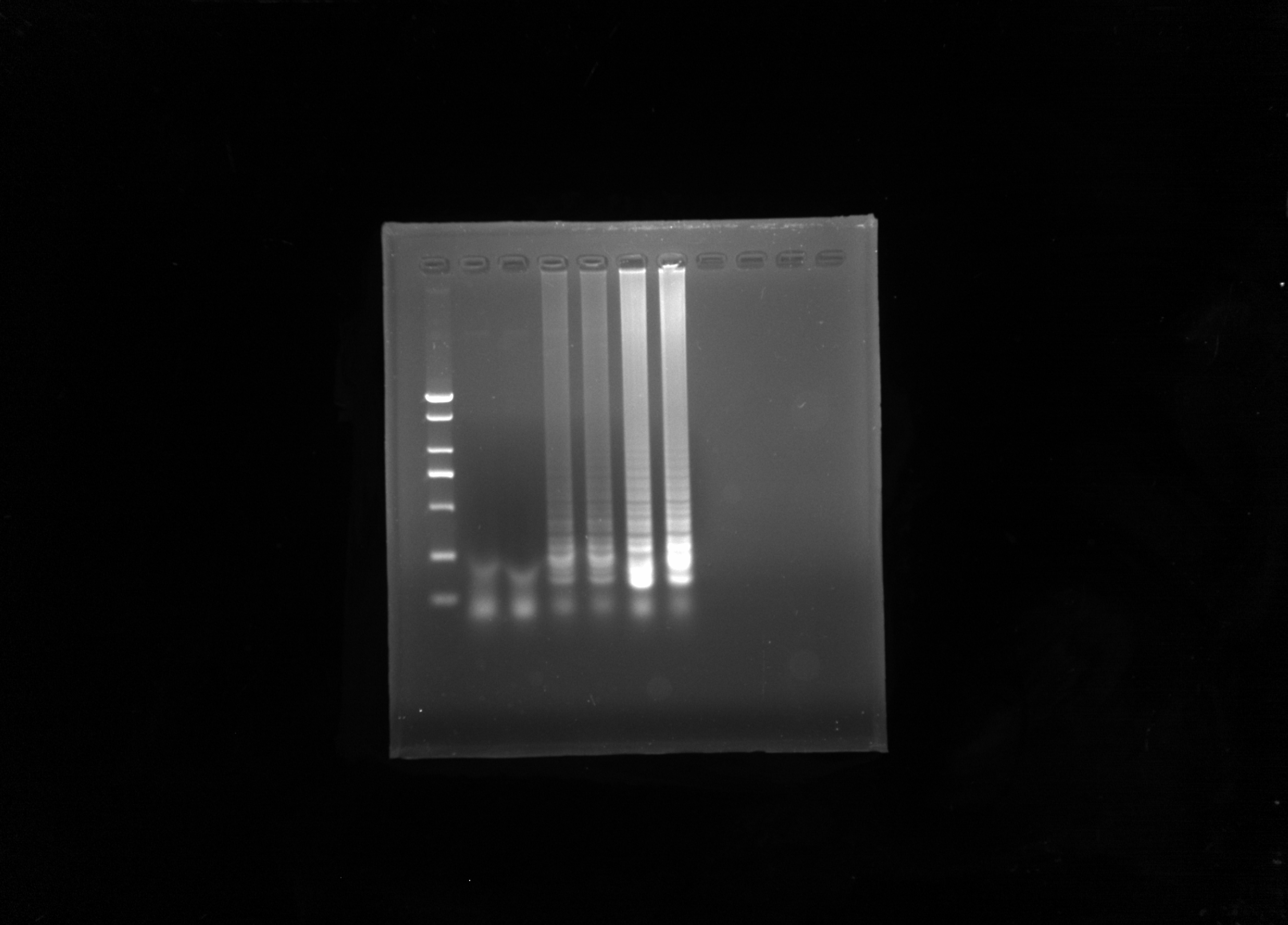


**Supplementary Figure S4g. The effect of Betaine in the optimization experiment.**


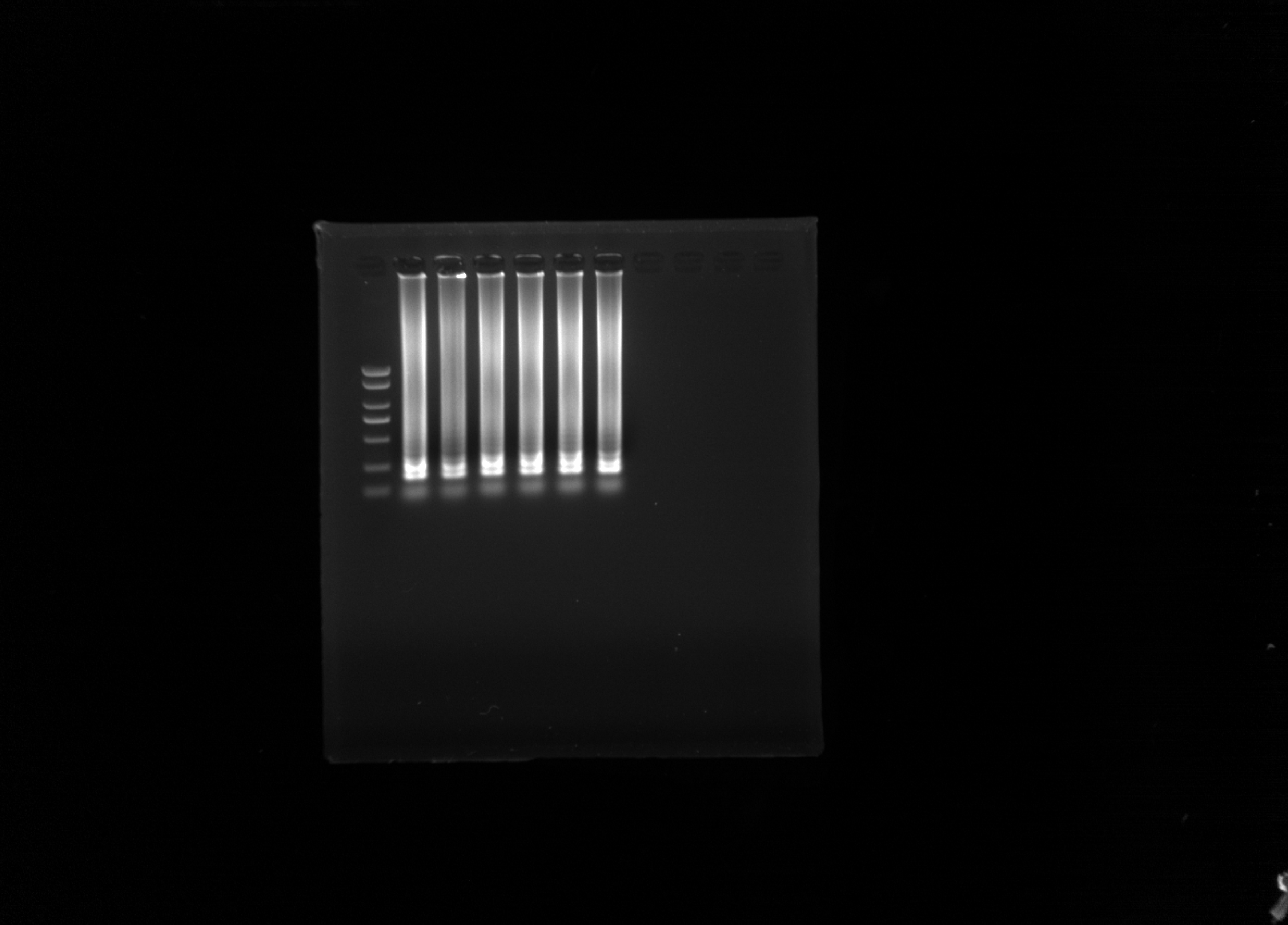


**Supplementary Figure S5a**


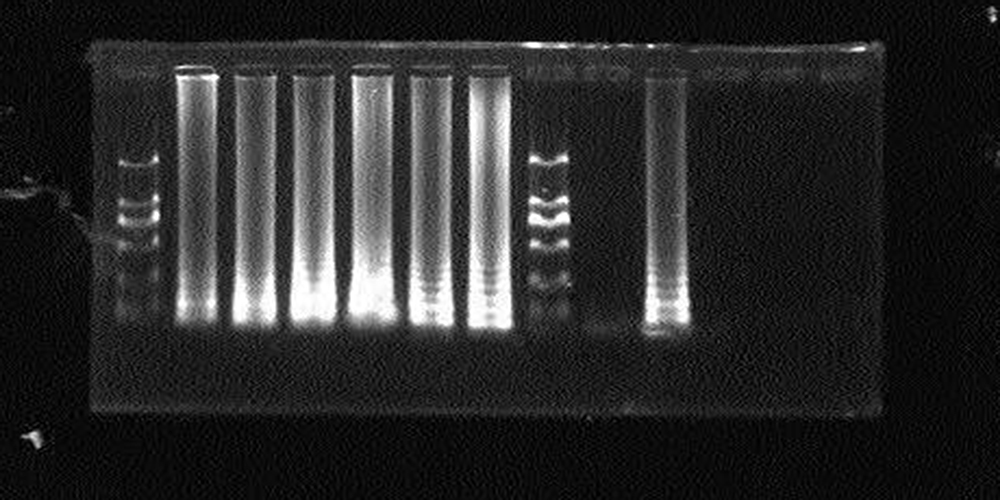


**Supplementary Figure S6a**


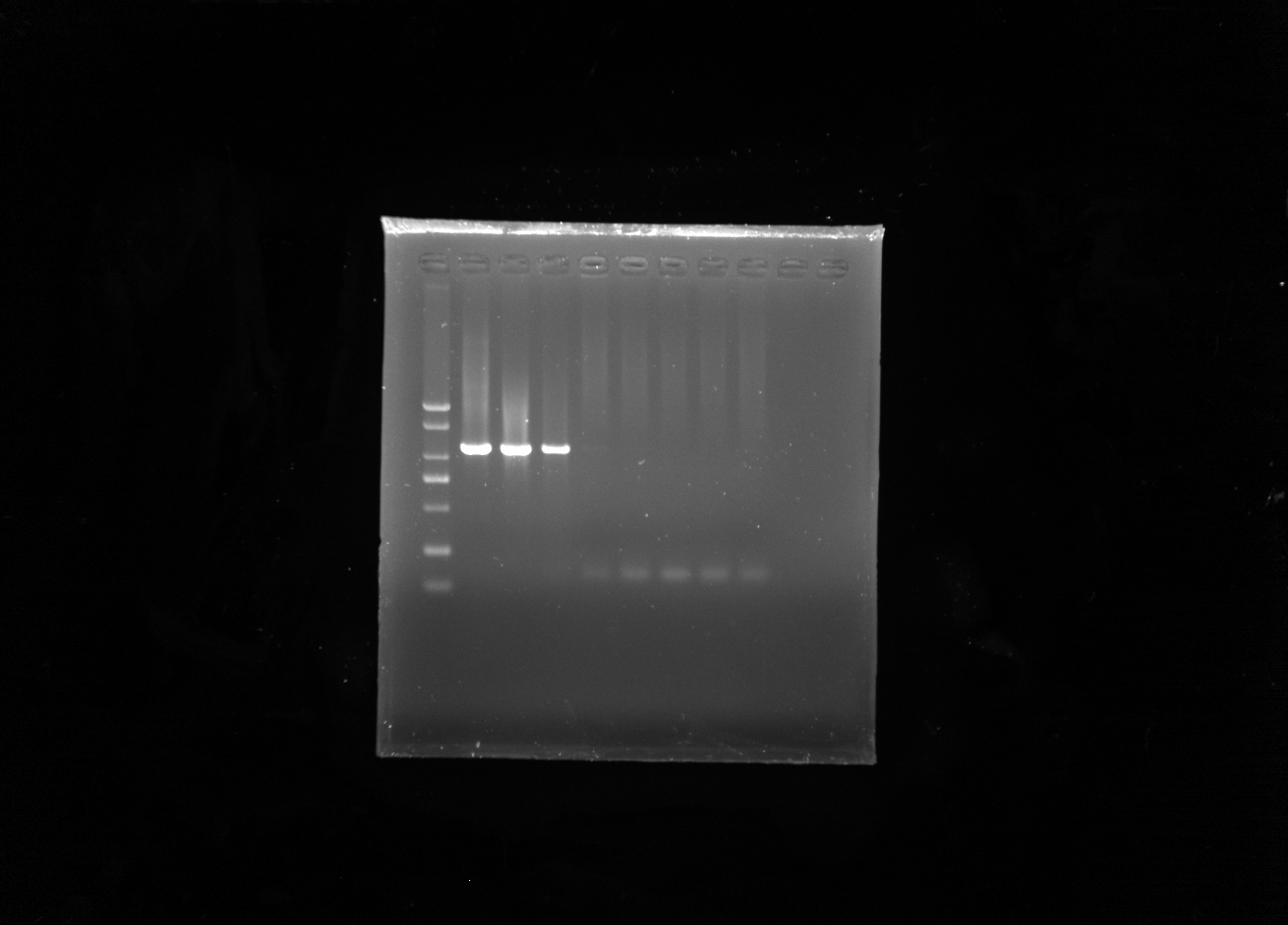


**Supplementary Figure S6b**


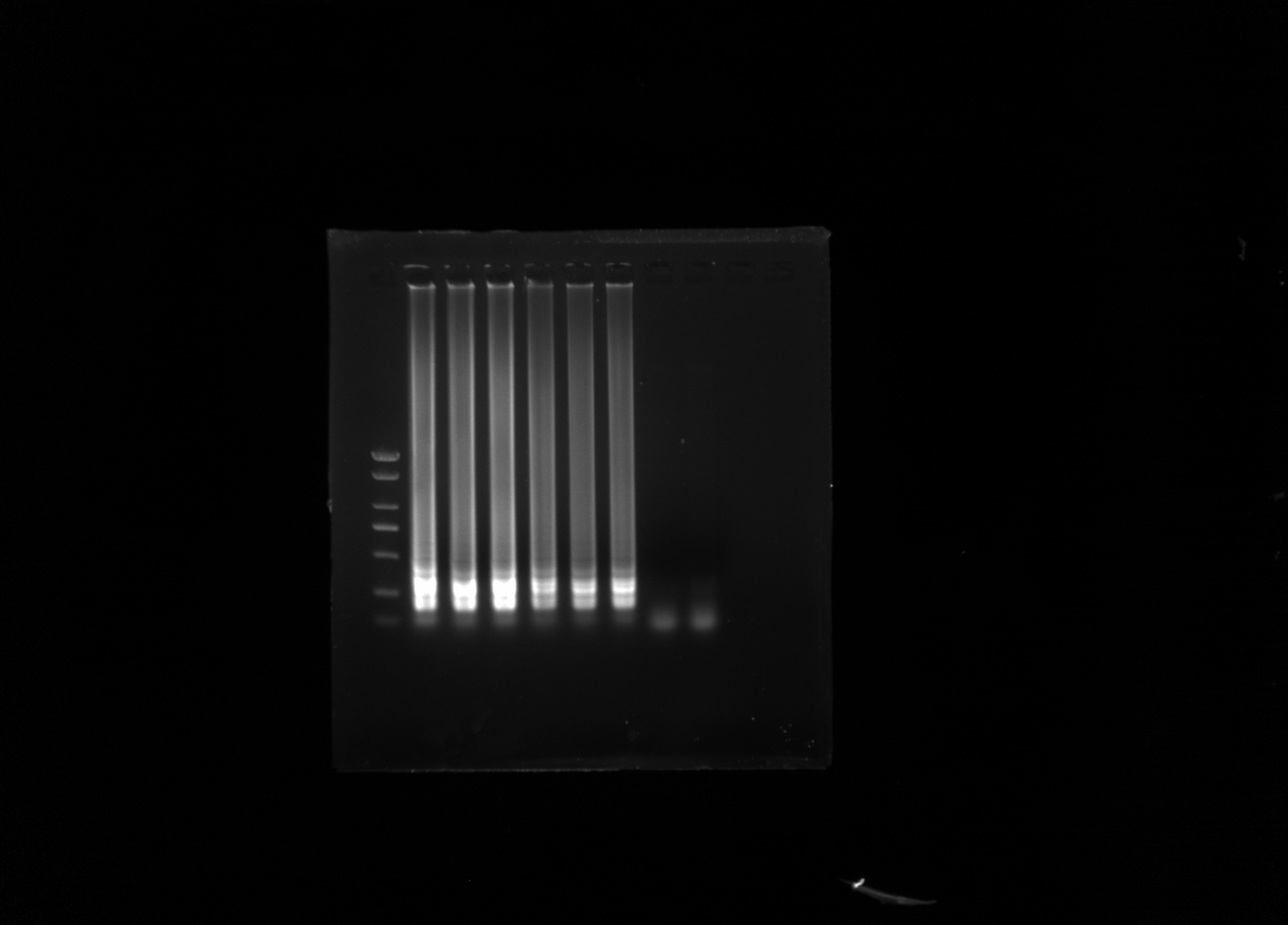


**Supplementary Figure S7a**


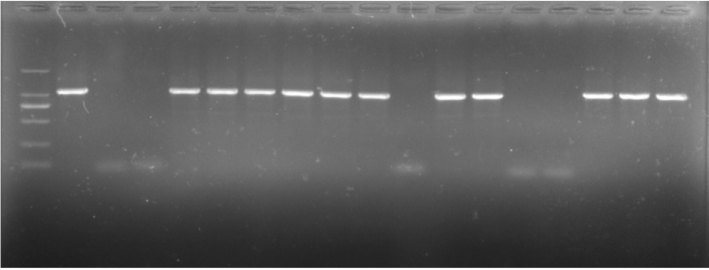


**Supplementary Figure S7b**


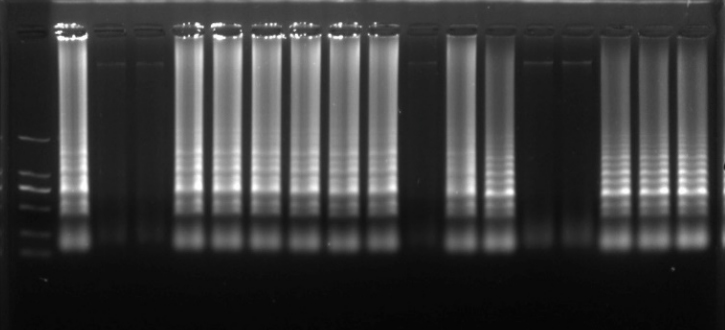

Supplement: Supplementary file 3 — Supplementary Figures. [file 41598_2022_8981_MOESM3_ESM.doc]
