# Supplementary material for: Molecular characterization of strawberry vein banding virus from China and the development of loop‑mediated isothermal amplification assays for their detection
Source: Sci Rep. 2022 Mar 22;12:4912. doi: 10.1038/s41598-022-08981-9 (PMC8940885; doi:10.1038/s41598-022-08981-9)
Supplement: Supplementary file 4 — Supplementary Tables. [file 41598_2022_8981_MOESM4_ESM.docx]

**Supplementary Table 1 Recombination analysis of SVBV**

| recombination event | major parent | minor parent | Programs detected the recombination and the corresponding *P*-value | | | | | | | Beginning breakpoint | Ending breakpoint |
| --- | --- | --- | --- | --- | --- | --- | --- | --- | --- | --- | --- |
|  |  |  | RDP | GENECONV | BootScan | MaxChi | Chimaera | Siscan | 3Seq |  |  |
| HE681085  (Anhui, China) | KX950836  (Canada) | MF197916 (China, Beijing) | 4.615×10^-68^ | 1.069×10^-61^ | 1.055×10^-61^ | 3.381×10^-35^ | 4.034×10^-35^ | 1.013×10^-35^ | 2.472×10^-115^ | 2277 | 5774 |
| MT027006  (Beijing, China) | MT012732  (Beijing, China) | KX950836  (Canada) | 1.402×10^-57^ | 2.745×10^-55^ | ------ | 1.759×10^-19^ | 8.983×10^-20^ | 3.995×10^-21^ | 7.775×10^-49^ | 2648 | 3240 |
| KX787430  (Anhui, China) | HE681085  (Anhui, China) | X97304  (USA) | 2.359×10^-28^ | 3.511×10^-19^ | 9.408×10^-22^ | 6.527×10^-28^ | 1.924×10^-25^ | 1.436×10^-24^ | 1.223×10^-44^ | 76 | 1736 |
| MT036056  (Xinjiang, China) | MT036057  (Xinjiang, China) | KX950836  (Canada) | 8.003×10^-22^ | 2.345×10^-20^ | ------ | 2.235×10^-15^ | 5.346×10^-14^ | 3.625×10^-11^ | 9.590×10^-31^ | 2610 | 3158 |
| KX950836  (Canada) | KP311681  (Liaoning, China) | X97304  (USA) | 1.206×10^-25^ | ------ | 1.329×10^-21^ | 2.951×10^-13^ | 1.847×10^-16^ | ------ | 4.821×10^-27^ | 2744 | 3582 |
| KX950836  (Canada) | MN956520  (Shanxi, China) | X97304  (USA) | 1.799×10^-17^ | ------ | 7.412×10^-16^ | 1.914×10^-09^ | 5.385×10^-07^ | ------ | 1.196×10^-11^ | 5384 | 5930 |
| MT731326 (China,Shandong) | KX249735  (Beijing, China) | MT036054  (Beijing, China) | 2.796×10^-12^ | 5.353×10^-12^ | 5.387×10^-11^ | 4.031×10^-08^ | 2.763×10^-08^ | 6.918×10^-10^ | 8.382×10^-17^ | 4043 | 4862 |
| KR080547  (Beijing, China) | MT012734  (Liaoning, China) | KX787430  (Anhui, China) | 1.263×10^-11^ | 1.432×10^-09^ | 8.578×10^-05^ | 6.817×10^-09^ | 2.758×10^-09^ | 5.386×10^-12^ | 3.075×10^-09^ | 5022 | 5870 |
| KT250632  (Beijing, China) | MT036054  (Beijing, China) | MT012732  (Beijing, China) | 1.352×10^-07^ | 8.449×10^-05^ | 3.041×10^-02^ | 2.589×10^-08^ | 1.305×10^-07^ | 2.591×10^-06^ | 2.530×10^-11^ | 5376 | 7158 |
| MT036055  (Beijing, China) | LC315804  (Japan) | MF197916 (China, Beijing) | 6.420×10^-04^ | 7.742×10^-07^ | ------ | 1.383×10^-09^ | 2.189×10^-10^ | 6.644×10^-11^ | 2.162×10^-02^ | 4337 | 5492 |
| MT027006  (Beijing, China) | MT731326 (China,Shandong) | MF197916 (China, Beijing) | 4.832×10^-05^ | 3.446×10^-04^ | ------ | 1.840×10^-06^ | 5.059×10^-07^ | 3.693×10^-05^ | 4.286×10^-09^ | 5108 | 6102 |
| KX787430  (Anhui, China) | MT012732  (Beijing, China) | HE681085  (Anhui, China) | ------ | 1.676×10^-02^ | 2.699×10^-02^ | 3.411×10^-05^ | 2.058×10^-02^ | ------ | 7.015×10^-09^ | 2349 | 4624 |
| KX787430  (Anhui, China) | MF197916 (China, Beijing) | KP311681  (Liaoning, China) | 3.273×10^-08^ | 7.523×10^-07^ | ------ | 1.060×10^-06^ | 7.687×10^-06^ | ------ | 7.513×10^-09^ | 2220 | 5586 |
| MT027007  (Beijing, China) | LC315804  (Japan) | HE681085  (Anhui, China) | ------ | 3.917×10^-04^ | ------ | 2.489×10^-08^ | 3.232×10^-08^ | 9.001×10^-08^ | 1.858×10^-08^ | 6580 | 8052 |
| MT027007  (Beijing, China) | KT250632  (Beijing, China) | MT036057  (Xinjiang, China) | 1.741×10^-03^ | 1.901×10^-03^ | ------ | 4.078×10^-07^ | 1.762×10^-07^ | 5.595×10^-08^ | 1.769×10^-10^ | 3350 | 4786 |
| HE681085  (Anhui, China) | MT027007  (Beijing, China) | MF197916 (China, Beijing) | ------ | 1.858×10^-03^ | ------ | 1.571×10^-04^ | 1.513×10^-04^ | 1.163×10^-04^ | 1.995×10^-08^ | 5775 | 5949 |
| MT036055  (Beijing, China) | LC315804  (Japan) | KX950836  (Canada) | ------ | 1.290×10^-06^ | 3.407×10^-02^ | 6.343×10^-06^ | 1.946×10^-06^ | 6.136×10^-05^ | 8.356×10^-07^ | 6885 | 8052 |
| MT012734  (Liaoning, China) | KX249735  (Beijing, China) | KP311681  (Liaoning, China) | 6.019×10^-04^ | 4.218×10^-02^ | 3.617×10^-02^ | 1.697×10^-02^ | 8.279×10^-03^ | ------ | 2.629×10^-02^ | 4043 | 4610 |
| KP311681  (Liaoning, China) | MT027007  (Beijing, China) | KX950836  (Canada) | 7.908×10^-05^ | 1.446×10^-02^ | ------ | 4.668×10^-05^ | 1.598×10^-03^ | ------ | 1.780×10^-03^ | 5274 | 6467 |
| KT250632  (Beijing, China) | HE681085  (Anhui, China) | KX950836  (Canada) | ------ | 2.459×10^-03^ | 1.130×10^-02^ | 1.082×10^-02^ | 1.916×10^-02^ | 4.582×10^-03^ | ------ | 4624 | 5026 |
| LC315804  (Japan) | MT027006  (Beijing, China) | KX950836  (Canada) | 3.075×10^-03^ | ------ | ------ | 3.013×10^-05^ | 1.617×10^-04^ | 3.019×10^-05^ | 3.838×10^-04^ | 4492 | 4955 |

| Parameters | Settings | | | | | |
| --- | --- | --- | --- | --- | --- | --- |
| Temperature (℃) | 60 | 61 | 62 | 63 | 64 | 65 |
| Time (min) | 30 | 45 | 60 | 75 |  |  |
| Primers FIP/BIP (μM) | 1.0 | 1.2 | 1.4 | 1.6 | 1.8 |  |
| Primers F3/B3 (μM) | 0.1 | 0.15 | 0.2 | 0.25 | 0.3 |  |
| Mg^2+^ (mM) | 2.0 | 4.0 | 6.0 | 8.0 | 10.0 |  |
| dNTPs (mM) | 0 | 0.4 | 0.8 | 1.2 | 1.6 | 2.0 |
| Betaine (M) | 0 | 0.6 | 0.8 | 1.0 | 1.2 | 1.4 |

**Supplementary Table 2 Matrix of parameters to indicate the tested sets of parameters**
